# Light‐Driven Ratchet Mechanism Accelerates Regioselective Metal‐Cation Exchange in a Heterobimetallic Helicate

**DOI:** 10.1002/anie.202508952

**Published:** 2025-06-30

**Authors:** Maximilian J. Notheis, Gregor Schnakenburg, Larissa K. S. von Krbek

**Affiliations:** ^1^ Kekulé‐Institut für Organische Chemie and Biochemie Rheinische Friedrich‐Wilhelms‐Universität Bonn Gerhard‐Domagk‐Str. 1 53121 Bonn Germany; ^2^ Institut für Anorganische Chemie Rheinische Friedrich‐Wilhelms‐Universität Bonn Gerhard‐Domagk‐Str. 1 53121 Bonn Germany

**Keywords:** Heterometallic complexes, Molecular ratchet, Out‐of‐equilibrium, Self‐assembly, Self‐sorting

## Abstract

Molecular machines rely on their capacity to exploit non‐equilibrium processes to perform work. However, the development of these non‐equilibrium processes, such as molecular ratchets, is still in its early stages. Here, we report a diazocine‐containing ligand (**L**) harbouring two distinct chelating coordination sites that can self‐sort into dinuclear homo‐ and heterobimetallic helicates ([Fe^II^_2_ **L**](OTf)_4_, [Co^II^_2_ **L**](OTf)_4_, [Zn^II^_2_ **L**](OTf)_4_, [Zn^II^Fe^II^ **L**](OTf)_4_, [Zn^II^Co^II^ **L**](OTf)_4_) with precisely controlled metal cation distribution. The photoisomerisation of the helicates operates via a molecular ratchet mechanism, resulting in metastable diastereomers that shift the system from thermodynamic equilibrium. Continuous white‐light irradiation autonomously drives this ratchet process, selectively enriching an out‐of‐equilibrium pseudo‐mesocate structure. Crucially, the ratchet mechanism can significantly accelerate metal‐cation exchange from the [Zn^II^_2_ **L**](OTf)_4_ helicate to the [Zn^II^Fe^II^ **L**](OTf)_4_ helicate. Thus, the system operates in a manner reminiscent of a “claw machine”, selectively seizing Fe^II^ ions when subjected to a precisely controllable external stimulus. These findings lay the foundation for creating adaptive and reconfigurable supramolecular structures that use non‐equilibrium phenomena on a molecular level.

## Graphical abstract

Photoswitchable homo‐ and heterobimetallic helicates form with high diastereo‐ and regioselectivity via single‐step self‐assembly or self‐sorting. White‐light exposure autonomously operates one helicate as a molecular ratchet, promoting the selective formation of an out‐of‐equilibrium structure. This ratchet mechanism furthermore accelerates regioselective metal‐cation exchange from a homometallic to a heterometallic helicate.

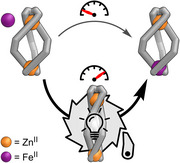

## Introduction

Harnessing endergonic reactions is crucial for operating molecular machinery and creating adaptive materials.^[^ ^1^ ^]^ A prime example of harnessing endergonic reactions is photosynthesis, which captures light energy to create a proton gradient.^[^ ^2^ ^]^ This gradient powers ATP synthase,^[^ ^3^ ^]^ converting ADP into ATP through an endergonic reaction. In essence, photosynthesis acts as a molecular ratchet,^[^ ^1^, ^4^ ^]^ coupling an exergonic proton transport process with an endergonic reaction within a multi‐step chemical reaction cycle.

Molecular ratchets function unidirectionally and bypass microscopic reversibility,^[^ ^5^ ^]^ allowing them to access out‐of‐equilibrium states. Chemical stimuli or light can activate molecular ratchets,^[^ ^6^, ^7^ ^]^ with light being a notably appealing option due to its unique benefits: light generates no chemical waste and allows for precise spatial and temporal control.^[^ ^8^ ^]^ To effectively harness light in artificial molecular ratchets, precisely aligning the photoresponsive building blocks, such as azobenzenes,^[^ ^9^, ^10^, ^11^, ^12^, ^13^ ^]^ diazocines,^[^ ^14^, ^15^, ^16^ ^]^ crowded alkenes^[^ ^17^, ^18^ ^]^ or dithienylethanes (DTE),^[^ ^19^, ^20^ ^]^ can enhance the switching effect. A key method for organising several photoresponsive building blocks within one structure is through the self‐assembly of supramolecular organic or metal‐organic capsules.^[^ ^8^, ^21^, ^22^ ^]^ Recently, Feringa, Kathan and colleagues^[^ ^11^ ^]^ reported a supramolecular organic capsule that functions as a molecular ratchet, using photoisomerisation and imine exchange to form a kinetically trapped, out‐of‐equilibrium open capsule. In another study, Clever, Herges and co‐workers^[^ ^14^ ^]^ introduced a diazocine‐containing ligand that preferentially formed an out‐of‐equilibrium Pd^II^_2_L_4_ lantern when exposed to light in the presence of Pd^II^ cations and disassembled in darkness.

Improving the structural complexity of metallo‐supramolecular capsules by integrating various ligands or metal cations into heteroleptic^[^ ^23^, ^24^ ^]^ or heterometallic^[^ ^25^ ^]^ structures, or by incorporating asymmetric ligands,^[^ ^26^, ^27^, ^28^ ^]^ may broaden their utility in guest binding and supramolecular catalysis, thus achieving the molecular recognition and catalytic regulation akin to that seen in biological host–guest systems.^[^ ^29^ ^]^ Heterometallic structures are known for their improved magnetic^[^ ^30^ ^]^ and photophysical properties,^[^ ^31^ ^]^ along with enhanced redox^[^ ^32^ ^]^ and catalytic activity^[^ ^33^ ^]^ compared to their homometallic counterparts. However, accurately self‐sorting various metal cations and ligands into heterobimetallic structures presents a substantial challenge, particularly when using metal cations that prefer similar ligand environments. A stepwise synthesis is commonly used,^[^ ^34^, ^35^, ^36^, ^37^, ^38^, ^39^, ^40^, ^41^, ^42^, ^43^ ^]^ starting with the assembly of one kinetically inert metal–ligand corner, followed by forming the rest of the structure using the pre‐assembled building block or metal cation exchange. While this approach tends to be effective, it involves two steps, requiring more synthetic effort than homometallic metallo‐supramolecular one‐pot self‐assembly. A small number of one‐pot self‐assembly examples of heterobimetallic cages have been reported,^[^ ^25^, ^35^, ^44^, ^45^, ^46^, ^47^ ^]^ employing the two distinct coordination geometries of two different metal cations.

Here, we present a diazocine‐based ligand (**L**) that self‐assembles into various metallo‐supramolecular M^II^_2_ **L** helicates in the presence of different metal(II) cations (Zn^II^, Fe^II^, and Co^II^). The ligand features two chemically distinct coordination sites, allowing the formation of both homo‐ and heterobimetallic helicates (Fe^II^_2_ **L**, Co^II^_2_ **L**, Zn^II^_2_ **L**, Zn^II^Fe^II^ **L**, Fe^II^Zn^II^ **L**, and Zn^II^Co^II^ **L**) with precise metal distribution achieved through one‐pot self‐sorting, sequential metal addition or metal exchange. Crucially, the reversible photoisomerisation of diazocines in **L** induces structural changes in the M^II^_2_ **L** helicate, thereby shifting the assemblies away from the thermodynamic minimum, akin to a molecular ratchet. Continuous exposure to white light autonomously drives this molecular ratchet, leading to the formation of a single high‐energy isomer with notable selectivity. Furthermore, we used this light‐driven molecular ratchet to facilitate the dynamic and selective exchange of metal cations, transforming the homometallic Zn_2_ **L** helicate into a heterobimetallic ZnFe**L** helicate.

## Results and Discussion

### Synthesis and Photoswitching of Ligand **L**

Photoresponsive subcomponent **1** was synthesised from 2‐bromo‐8‐iodo‐11,12‐dihydrodibenzo[c,g][1,2]diazocine^[^ ^48^ ^]^ (Scheme S1 and Section S2) via two successive, selective, and high‐yielding cross‐coupling reactions with ethyl pinacol boronic acid esters^[^ ^49^ ^]^ 5‐(4,4,5,5‐tetraethyl‐1,3,2‐dioxaborolan‐2‐yl)‐2,2′‐bipyridine (**S3**) and 2‐(1,3‐dioxolan‐2‐yl)‐5‐(4,4,5,5‐tetraethyl‐1,3,2‐dioxaborolan‐2‐yl)pyridine (**S6**).

**Scheme 1:**
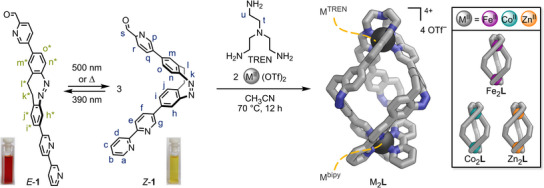
Left: Photoswitching between the two isomers of **1** and their photochromism in acetonitrile. Right: Subcomponent self‐assembly of *Z*‐**1**, TREN and M(OTf)_2_ yielding M_2_ **L** (GFN2‐xTB optimised structure: hydrogens and counterions omitted for clarity; C: grey, N: blue, M^II^ (dark grey) = Fe^II^, Co^II^, Zn^II^). Insert: Helicate structures obtained by self‐assembly (Fe_2_ **L**, Co_2_ **L**, Zn_2_ **L**).

At ambient conditions, subcomponent **1** primarily exists as its thermodynamically stable *Z*‐isomer (99% *Z*‐**1**). When exposed to 390 nm light, a second set of signals associated with *E*‐**1** emerged in the ^1^H NMR spectrum, reaching a photostationary state of 72% *E*‐**1** and resulting in a colour change of the solution from yellow to red (Scheme 1, left; Section S7.4). *E→Z* isomerisation (>99% *Z*‐**1**) took place under white light or 500 nm light irradiation and through thermal relaxation, exhibiting a half‐life of 165 min at 25 °C in acetonitrile (20 µM; Section S7.3.1). The photoswitching of subcomponent **1** is rapid and completely reversible, usually taking less than a minute of irradiation in either direction (20 µM–2 mM in CH_3_CN, 0.1–0.2 mW light power). The asymmetric 2,8‐substitution pattern at the central diazocine unit in subcomponent **1** enables a significant structural change from a 90° to a 180° angle between the two metal‐binding moieties upon photoswitching (Scheme 1, left; Figure S67). Employing two distinct metal‐binding moieties generates directionality within asymmetric subcomponent **1**. Dynamic covalent tethering of **1**’s pyridine carboxaldehydes into TREN tri‐pyridylimines fixes subcomponent **1**’s orientation, leading to regioselective helicate self‐assembly, as previously demonstrated^[^ ^26^ ^]^ in related systems.

### Synthesis of Homometallic Helicates

Homometallic, triple‐stranded helicates Fe_2_ **L**, Co_2_ **L**, and Zn_2_ **L** were obtained from the self‐assembly of pyridine carboxaldehyde **1** (1 equiv.), tris(2‐aminoethyl)amine (TREN, 1 equiv.), and the corresponding metal(II) trifluoromethanesulfonates (triflate or ^−^OTf, 2 equiv.), Fe(OTf)_2_, Co(OTf)_2_, and Zn(OTf)_2_, respectively (Scheme 1, right). Fe_2_ **L**, Co_2_ **L**, and Zn_2_ **L** were characterised by mass spectrometry, NMR spectroscopy, and UV–vis spectroscopy (Sections S3 and S7.3).

**Figure 1:**
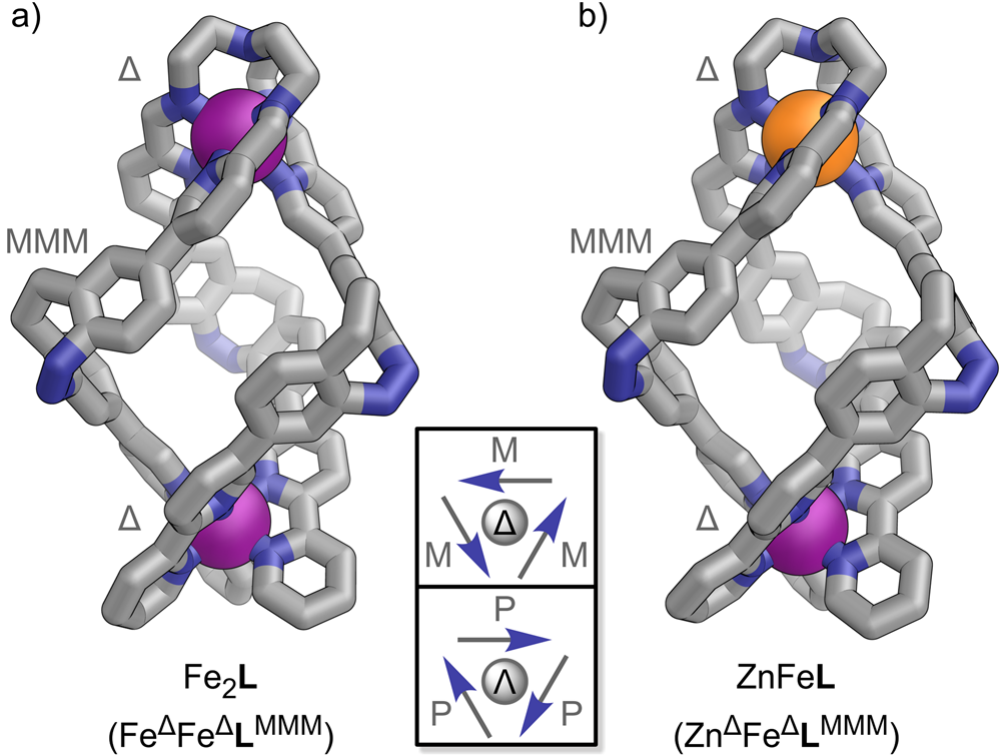
Crystal structures of a) homometallic helicate Fe_2_ **L** and b) heterobimetallic helicate ZnFe**L** (C: grey, N: blue, Fe: purple, Zn: orange). Disorder, solvent of crystallisation, and anions are omitted for clarity. Insert: Schematic representation of the helicates clarifying *nomenclature* (looking along the M^TREN–^M^bipy^ axis; metal cations: grey circles; diazocine units: arrows): Δ/Λ: right‐/lefthanded helicity of the metal cation ligand environment; P/M: diazocine azo‐nitrogens pointing clockwise/anti‐clockwise (see also Section S6.1).

Fe_2_ **L** crystallised in the triclinic space group P1 (Figure 1a; Section S6.2). Triple‐stranded ligand **L** connects two homochiral metal centres, forming a dinuclear helicate structure with distorted *C*_3_ symmetry. All *Z*‐diazocine units maintain the same orientation within ligand **L**, with their helical configuration (**L** ^MMM^) opposing the helicity of the metal centres (Fe^ΔΔ^, Figure 1a; Sections S6.1 and S6.2). The self‐assembly of dinucelar homometallic helicate Fe_2_ **L** exhibits remarkable diastereoselectivity, resulting in the formation of only one pair of enantiomers: Fe^Δ^Fe^Δ^ **L** ^MMM^ and Fe^Λ^Fe^Λ^ **L** ^PPP^.

**Figure 2:**
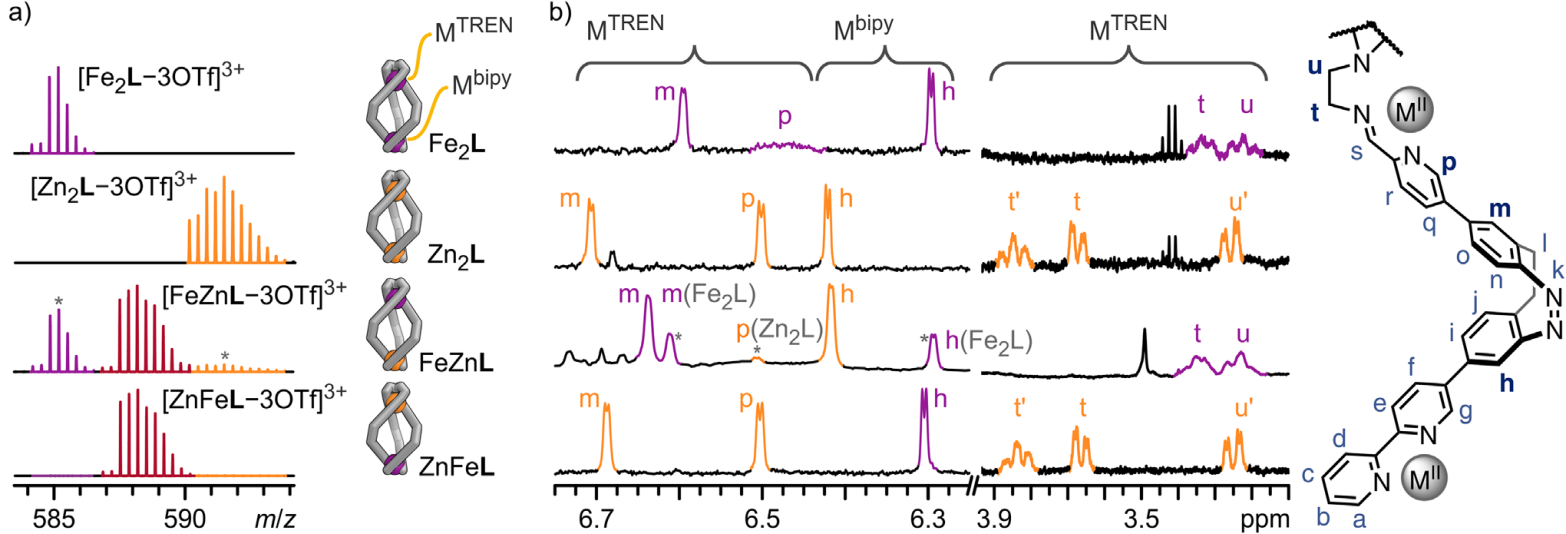
Analytical data of Fe_2_ **L**, Zn_2_ **L**, FeZn**L**, and ZnFe**L** (top to bottom). a) Partial high‐resolution ESI^+^ mass spectra (CH_3_CN) showing the respective [MM'**L–**3OTf]^3+^ isotope patterns. b) Partial ^1^H NMR spectra (500 MHz, CH_3_CN, 298 K) showing characteristic proton signals of the M^TREN^ and M^bipy^ coordination sites, respectively. Asterisks denote signals from Zn_2_ **L** and Fe_2_ **L**, respectively, indicating reduced selectivity in the stepwise formation of FeZn**L**. The spectra of Fe_2_ **L** and Zn_2_ **L** exhibit residual diethyl ether signals, as completely drying the sample under high vacuum led to decomposition. Right: Partial chemical structure of ligand **L**.

**Figure 3:**
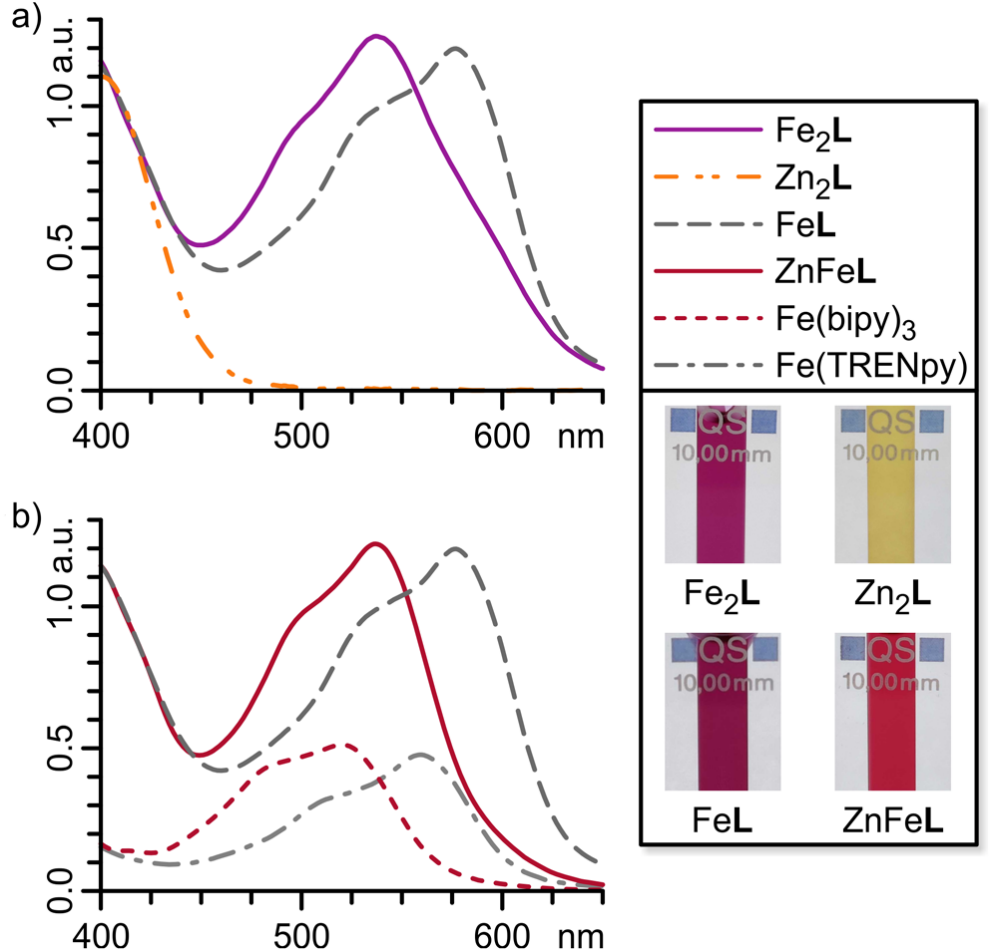
UV–vis spectra (0.03 mM, CH_3_CN) of a) Fe_2_ **L**, Zn_2_ **L**, and Fe^TREN^ **L** as well as b) Fe^TREN^ **L**, ZnFe**L**, Fe(bipy)_3_, and Fe(TRENpy). Insert: Photocromism of Fe_2_ **L**, Zn_2_ **L**, Fe^TREN^ **L**, and ZnFe**L**.

The high‐resolution electrospray ionisation (ESI) mass spectrum of Fe_2_ **L** showed a sequence of signals corresponding to the species [Fe_2_ **L **− *n*OTf]*^n^* ^+^ (*n* = 1, 2, 3, and 4), confirming the stoichiometry of Fe_2_ **L** in the gas phase (Figures 2a and S24). ^1^H NMR spectroscopy indicated a *C*_3_ symmetric structure with diasterotopic signals for the TREN ethylene groups, as expected for the chiral coordination pocket (Figures 2b and S19). Proton signals of H‐p, H‐s, H‐u′, and H‐t′ close to the TREN tri‐pyridylimine metal corner (Fe^TREN^) are significantly broadened with half‐widths of 30 to 50 Hz and are only visible in highly concentrated samples (Figure S19). ^1^H,^1^H ROESY spectroscopy revealed close interproton contacts between diazocine protons H‐h and H‐m with bipyridine proton H‐g and pyridylimine proton H‐p, respectively, confirming that the same racemic diastereomer of Fe_2_ **L**, namely Fe^Δ^Fe^Δ^ **L** ^MMM^, together with its enantiomer Fe^Λ^Fe^Λ^ **L** ^PPP^, is present in both solution and the solid state (Figure S56 and Section S6.4.1). The UV–vis spectrum of Fe_2_ **L** displays a diazocine n–π* absorption band at 405 nm, along with three bands in the visible range that are characteristic of Fe^II^ MLCT transitions (*λ* = 497, 539, and 599 nm; Figure 3a). By synthesising model compounds for the two unique metal binding sites in Fe_2_ **L**—Fe(bipy)_3_(OTf)_2_ and Fe(TREN tri‐pyridylimine)(OTf)_2_ (short: Fe(bipy)_3_ and Fe(TRENpy), respectively)—and comparing their UV–vis spectra to that of Fe_2_ **L** (Figure 3b), we assigned the three MLCT transition bands to the two distinct coordination sites in Fe_2_ **L**. The two lower‐wavelength bands are attributed to the MLCT transition of one Fe^II^ coordinated by three bipyridines (Fe^bipy^, *λ*_bipy_ = 497 and 539 nm). The second Fe^II^ coordinated by the TREN tri‐pyridylimine coordination pocket (Fe^TREN^) causes the much weaker higher‐wavelength band at *λ*_TREN_ = 599 nm. These findings align well with comparable Fe^II^ complexes documented in the literature.^[^ ^50^, ^51^ ^]^

Characterisation of homometallic helicates Co_2_ **L** and Zn_2_ **L** by mass spectrometry and NMR spectroscopy indicated the formation of similar structures to Fe_2_ **L** (Figures 2 and 3; Sections S3.3, S3.4 and S6.4.2).

The ^1^H NMR spectrum of Co_2_ **L** is characteristically dispersed over a range of nearly 200 ppm (Figure S26), due to the paramagnetism of Co^II^, which precludes further assignments of the signals. The UV–vis spectrum of Co_2_ **L** shows an MLCT absorption band at *λ* ≈ 350 nm and an almost unchanged diazocine n–π* absorption band (405 nm; Figure S72).

The ^1^H NMR spectrum of Zn_2_ **L** showed signal shifts similar to but clearly distinct from Fe_2_ **L** (Figure 2b). UV–vis spectroscopy of Zn_2_ **L** revealed an almost unchanged diazocine n–π* absorption band around 405 nm compared to subcomponent **1**, with no MLCT transition observed in the visible range (Figures 3a and S70).

The characteristic proton shifts of H‐h, H‐m, H‐p, H‐t, and H‐u of Fe_2_ **L** and Zn_2_ **L** in ^1^H NMR (Figure 2b), as well as the three characteristic MLCT bands of Fe^bipy^ and Fe^TREN^ in UV–vis (Figure 3a), allow for detailed elucidation of Fe^II^ and Zn^II^ distribution between the two coordination sites of ligand **L**, enabling us to analyse heterobimetallic helicates precisely.

### Synthesis of Heterobimetallic Helicates

Ligand **L** features two coordination sites that are chemically, kinetically, and thermodynamically distinct: a TREN tri‐pyridylimine and a bipyridine. The sixfold chelation of the TREN tri‐pyridylimine coordination pocket (M^TREN^) is kinetically more inert and potentially thermodynamically preferred over the three twofold chelating bipyridines (M^bipy^) due to an enhanced chelate effect similar to that of a cryptand. The greater stability of TREN tri‐pyridylimine coordination sites, compared to three individual pyridylimines, has been used in previous applications for subcomponent exchange and cage stabilisation.^[^ ^52^, ^53^ ^]^ Considering kinetics, the bipyridine binding site (M^bipy^) should be accessible first, as the TREN tris‐pyridylimine coordination pocket (M^TREN^) has not yet formed via metal‐mediated imine‐bond formation. Thus, employing ligand **L** alongside two different metal cations, with one forming thermodynamically more stable M─N bonds^[^ ^54^ ^]^ and exhibiting slower ligand exchange kinetics,^[^ ^55^, ^56^, ^57^ ^]^ should result in the selective assembly of heterobimetallic helicates. Potential combinations of metal cations that favour an octahedral coordination sphere include Fe^II^ and Zn^II^ or Co^II^ and Zn^II^.

**Figure 4:**
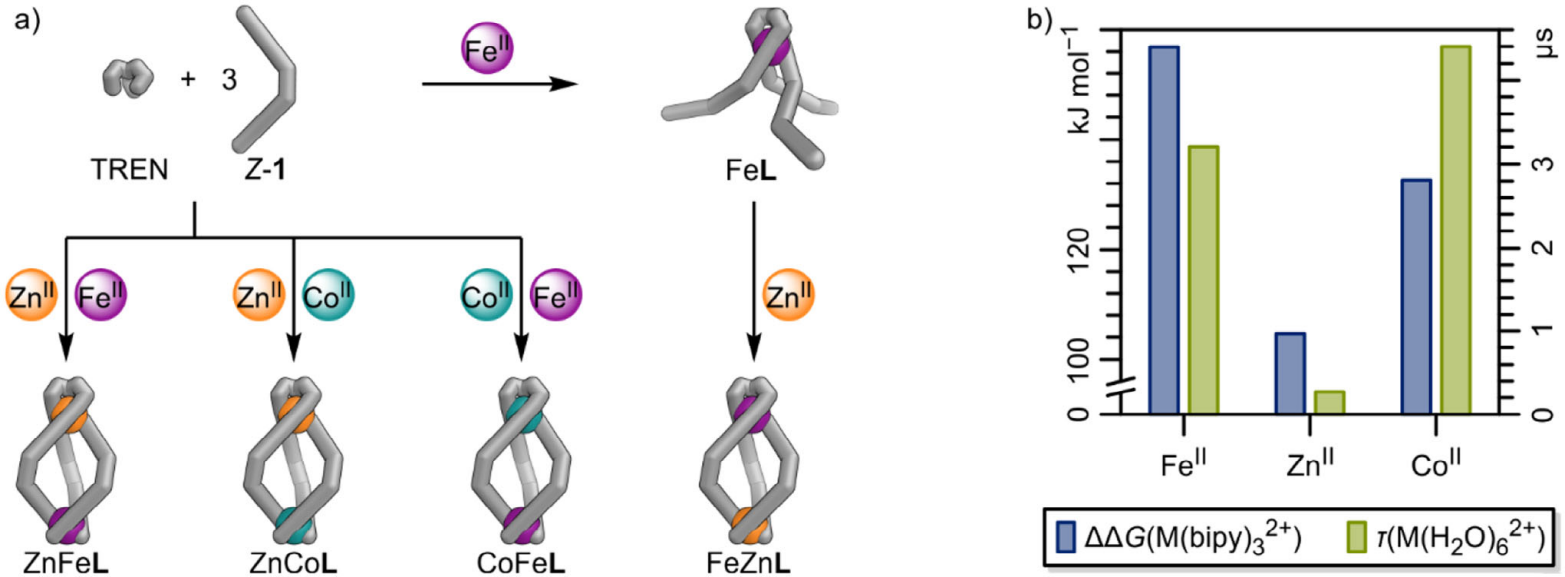
a) Stepwise self‐assembly of heterobimetallic helicate FeZn**L** from TREN and *Z*‐**1** and metal‐cation self‐sorting upon self‐assembly of ZnFe**L**, ZnCo**L**, and CoFe**L**. Reaction conditions: CH_3_CN, 70 °C, 12 h. b) Overview of thermodynamic and kinetic differences of Fe^II^, Zn^II^ and Co^II^ complexes. Blue: Gibbs free energies^[^ ^54^ ^]^ of the dissociation of one bipyridine ligand in M(bipy)_3_. Green: mean lifetimes of coordinated water in M(H_2_O)_6_^2+^ in water.^[^ ^55^, ^56^, ^57^ ^]^

Using the greater stability and kinetic inertness of the TREN tri‐pyridylimine binding pocket (M^TREN^), we attempted the consecutive assembly of a heterobimetallic helicate by mixing pyridine carboxaldehyde **1** (3 equiv.), TREN (1 equiv.), and Fe(OTf)_2_ (1 equiv.) to form mononuclear complex Fe**L** with Fe^II^ in the TREN tris‐pyridylimine binding pocket (Fe^TREN^ **L**, Figure 4a). Subsequently, Zn(OTf)_2_ (1 equiv.) was added to Fe**L** to allow formation of a heterobimetallic helicate with Fe^II^ remaining in the TREN tris‐pyridylimine binding pocket (Fe^TREN^) and Zn^II^ coordinating to the three bipyridine units (Zn^bipy^), which we refer to as FeZn**L** (short for Fe^TREN^Zn^bipy^ **L**). High‐resolution ESI mass spectrometry confirmed the formation of an [Fe + **L**] species following the first reaction step, with minor amounts of Fe_2_ **L** also detected. After the second reaction step, an [Fe + Zn + **L**] species was generated, with some Fe_2_ **L** still present alongside newly formed Zn_2_ **L** (Figures 2a and S31). The presence of both Fe_2_ **L** and Zn_2_ **L**, alongside the desired FeZn**L**, suggests that the formation of Fe_2_ **L** is thermodynamically more favourable than the formation of Fe**L**, which results in unreacted or partially reacted TREN and **1** remaining in the reaction mixture. After the addition of Zn(OTf)_2_, the unreacted or partially reacted ligand subcomponents, TREN and **1**, form Zn_2_ **L**.

The ^1^H NMR spectrum of FeZn**L** confirmed the formation of a heterobimetallic helicate after the second reaction step (Figures 2b and S30). The chemical shifts of protons near the TREN tris‐pyridylimine binding pocket, specifically diazocine proton H‐m, pyridylimine proton H‐p, and ethylene protons H‐t and H‐u, are comparable to the chemical shifts of their Fe_2_ **L** counterparts. In contrast, the chemical shift of diazocine proton H‐h, which is close to the bipy unit, is nearly identical to that of its Zn_2_ **L** counterpart, indicating an Fe^TREN^Zn^bipy^ **L** structure. As indicated by the ESI mass spectrum, ^1^H NMR spectroscopy also suggested the formation of dinuclear homometallic Fe_2_ **L** and Zn_2_ **L** (25% and 10%, respectively, Figure 2b; Section S3.7). The higher proportion of Fe_2_ **L** compared to Zn_2_ **L** is likely due to an excess of Fe(OTf)_2_ (approximately 10%) present during the formation of Fe**L**. As described above, distinct MLCT absorption bands for Fe^II^, coordinated by three bipyridine units (Fe^bipy^) or a TREN tris‐pyridylimine binding pocket (Fe^TREN^), facilitated further structural conformation of Fe**L** and FeZn**L** using UV‐vis spectroscopy (Figures 3 and S32). The UV–vis spectra of Fe^TREN^ **L** and the reference compound Fe(TRENpy) are nearly identical, suggesting the presence of an Fe^TREN^ coordination sphere in Fe^TREN^ **L**. The UV–vis spectrum of FeZn**L** reveals a secondary band associated with the additional MLCT transition observed in the contaminant Fe_2_ **L**. Still, it displays a major band that is identical to the one found in Fe^TREN^ **L**. The difference in absorption bands between Fe^bipy^ and Fe^TREN^ is readily visible to the naked eye, as the bipyridine‐containing Fe^II^ complexes exhibit a vibrant red colour, whereas their pyridylimine counterparts present a deep purple hue (Figure 3, insert). The kinetics of Fe^TREN^ **L** complex formation were further investigated using UV–vis spectroscopy (Section S5), which indicated the nearly immediate formation of an Fe^bipy^ **1**_3_ complex, in which Fe^II^ is coordinated by three bipy units, as evidenced by the appearance of the characteristic Fe^bipy^‐MLCT absorption band. Over a few hours, the Fe^bipy^‐MLCT absorption band transformed into an Fe^TREN^‐MLCT absorption band, thus indicating the successful formation of Fe^TREN^ **L**. The progress of the reaction can also be observed with the naked eye: the reaction solution turns a distinctive Fe^bipy^‐red colour within minutes and then gradually changes to a deep violet shade, characteristic of Fe^TREN^, indicating the sequential formation of Fe^bipy^ **1**_3_ and Fe^TREN^ **L**. Therefore, we conclude that Fe^bipy^ **1**_3_ is kinetically favoured, whereas Fe^TREN^ **L** is potentially thermodynamically more stable but also kinetically more inert, thus confirming our assumption that the TREN tris‐pyridylimine coordination pocket should be the more stable binding site.

After confirming our assumption regarding the kinetic and thermodynamic coordination behaviours of aldehyde **1** and ligand **L**, we aimed to harness these properties to achieve heterobimetallic social self‐sorting using Fe^II^, Zn^II^, and Co^II^. Given that Fe^II^ and Zn^II^ show the most significant difference in thermodynamic and kinetic stabilities (Figure 4b), we initially sought to form a self‐sorted ZnFe**L** heterobimetallic helicate (short for Zn^TREN^Fe^bipy^ **L**) from a mixture of pyridine carboxaldehyde **1** (3 equiv.), TREN (1 equiv.), Fe(OTf)_2_ (1 equiv.), and Zn(OTf)_2_ (1 equiv.) in acetonitrile (Figure 4a). The self‐assembly of Fe^TREN^ **L** indicated that the M^bipy^ coordination site would form before the M^TREN^ binding site because it is readily accessible. In contrast, the M^TREN^ coordination site must be formed through three metal‐mediated imine condensation reactions, which occur on a significantly slower timescale. Consequently, we anticipated that both Fe^II^ and Zn^II^ would first coordinate to the bipy units of **1**, with Fe^II^ gradually replacing the thermodynamically less stable Zn^II^ (ΔΔ*G* ^Fe,Zn^(M(bipy)_3_^2+^) = 52.2 kJ mol^−1^)^[^ ^54^ ^]^ at all coordination sites, resulting in the formation of Fe^bipy^ **1**_3_. Once all Fe^II^ is coordinated to bipy (Fe^bipy^), only Zn^II^ should remain to facilitate the formation of TREN tri‐pyridylimine, thereby selectively installing Zn^II^ in the M^TREN^ coordination pocket to produce ZnFe**L** (short for Zn^TREN^Fe^bipy^ **L**). ZnFe**L** crystallised in the triclinic space group P1 (Figure 1b; Section S6.2). The solid‐state structure confirmed the expected Zn^TREN^‐Fe^bipy^ metal distribution in a triple‐stranded helicate structure almost identical to that of Fe_2_ **L** (Figure 1a; Section S6.2). High‐resolution ESI mass spectrometry confirmed the formation of a [Zn + Fe + **L**] heterobimetallic helicate (Figures 2a and S38). ^1^H NMR spectroscopy indicated the expected regioselectivity of the self‐sorted helicate ZnFe**L** (Zn^TREN^Fe^bipy^ **L**), as evidenced by the characteristic proton signal shifts of protons H‐h, H‐m, H‐p, H‐t, and H‐u corresponding to Zn^TREN^ and Fe^bipy^ coordination (Figure 2b). Furthermore, ^1^H,^1^H ROESY NMR spectroscopy confirmed the retention of the solid‐state structure in solution (Section S6.4.1). UV–vis spectroscopy revealed a characteristic Fe^bipy^‐MLCT absorption band (*λ*_bipy_ = 537 nm), whereas no Fe^TREN^‐MLCT absorption band (*λ*_TREN_ ≈ 580 nm) was detected, further supporting the successful self‐sorting within ZnFe**L** (Figures 3b and S76).

The investigation of the kinetics of ZnFe**L** complex formation via UV–vis spectroscopy further corroborated our proposed assembly mechanism for ZnFe**L** (Section S5). An Fe^bipy^‐MLCT absorption band (*λ*_bipy_ = 540 nm) quickly appeared, with no further changes to the spectra, indicating that Fe^II^ remained in the bipy binding site, thereby enabling the selective formation of TREN tri‐pyridylimine around Zn^II^. These findings suggest that this self‐sorting into ZnFe**L** is, indeed, a delicate interplay between ligand association and formation kinetics as well as metal‐coordination thermodynamics. Prolonged heating of ZnFe**L** (65 °C, 2 months) in the presence of 3.0 equiv. Fe^II^ led to the formation of 6% Fe_2_ **L**, demonstrating the kinetic inertness of the ZnFe**L** structure and the Zn^TREN^ coordination site while indicating that Fe_2_ **L** might be thermodynamically favoured over ZnFe**L** (Section S9.4).

To support our hypothesis that heterobimetallic helicate self‐sorting occurs due to a delicate interplay between metal–ligand stability and exchange kinetics, we attempted the synthesis of two additional heterobimetallic helicates using Zn^II^/Co^II^ and Co^II^/Fe^II^ mixtures. In these mixtures, both the differences in thermodynamic metal–ligand stability and in metal–ligand exchange kinetics are gradually reduced. While the difference in metal–ligand exchange kinetics between Zn^II^ and Co^II^ is comparable to that of the Zn^II^/Fe^II^ pair,^[^ ^55^, ^56^, ^57^ ^]^ the difference in M^bipy^‐association energy between Co(bipy)_3_^2+^ and Zn(bipy)_3_^2+^ is approximately half as high as that between Fe(bipy)_3_^2+^ and Zn(bipy)_3_^2+^ (ΔΔ*G* ^Fe,Zn^(M(bipy)_3_^2+^) = 52.2 kJ mol^−1^ and ΔΔ*G* ^Co,Zn^(M(bipy)_3_^2+^) = 28.0 kJ mol^−1^, respectively; Figure 4b).^[^ ^54^ ^]^ Therefore, helicate self‐assembly from a mixture of Zn^II^ and Co^II^ should indicate whether the difference in thermodynamics or kinetics of the metal cations has a more significant impact on successful self‐sorting. For Co^II^ and Fe^II^, both the difference in M^bipy^‐association energy between Co(bipy)_3_^2+^ and Fe(bipy)_3_^2+^ and the difference in metal–ligand exchange kinetics are small.^[^ ^54^, ^55^, ^56^, ^57^ ^]^ Consequently, if our hypothesis was correct, we expected poor self‐sorting, if any.

In the case of the one‐pot heterobimetallic helicate assembly involving Zn^II^, Co^II^, **1**, and TREN (Figure 4a), both ^1^H NMR spectroscopy and ESI mass spectrometry indicated selective self‐sorting into the ZnCo**L** (Zn^TREN^Co^bipy^ **L**) helicate (SI,Section S4.1.2). ESI mass spectrometry revealed a singular set of peaks for [Zn + Co + **L **+ *n*OTf]^(4−^ *^n^* ^)+^ (*n* = 0, 1, 2), and the wide‐sweep ^1^H NMR spectrum displayed only signals reminiscent of those in Co(bipy)_3_(OTf)_2_, with none reminiscent of a Co^II^‐TREN tri‐pyridylimine coordination.

Helicate assembly in the presence of Co^II^ and Fe^II^, however, resulted in a mixture of CoFe**L** (Co^TREN^Fe^bipy^ **L**, approx. 60%–70%), Fe_2_ **L** (approx. 15%) and Co_2_ **L** helicates (approx. 15%). The composition of the helicate mixture was tentatively estimated using ESI mass spectrometry, NMR, and UV–vis spectroscopy (Sections S4.3 and S4.4). The paramagnetic nature of Co^II^ hindered the determination of accurate values.

The attempted heterobimetallic self‐sorting experiments with Zn^II^/Co^II^ and Co^II^/Fe^II^ indicate that self‐sorting still occurs with a reduced difference in metal–ligand bond strength. However, self‐sorting is significantly less efficient if both the binding energy difference and the difference in ligand exchange kinetics are diminished, as seen in the Co^II^/Fe^II^ pair. Remarkably, Co^II^ can occupy both ligand **L**’s coordination sites, depending on the other metal cation present in the self‐sorting process. These results further support our proposed self‐sorting mechanism into heterobimetallic helicates (Scheme S54):

- Both metal cations form M^bipy^‐complexes with three equivalents of subcomponent **1**, respectively.
- Because of the disparity in metal–ligand binding energies (Fe^II^ > Co^II^ > Zn^II^), the more stable metal cation replaces the weaker in the M^bipy^ complexes and gets sequestered by the M^bipy^ coordination sphere.
- “Trapping” the more stable cation in the M^bipy^ coordination sphere allows the thermodynamically less stable metal cation to facilitate the formation of the TREN tri‐pyridylimine binding pocket M^TREN^, thereby completing the self‐sorted heterobimetallic helicate.

### Photoswitching of Zn_2_ **L**

After demonstrating the ability of ligand **L** to form either dinuclear homometallic helicates or even heterobimetallic ones via the effective self‐sorting of two different metal cations, we investigated the effects of switching the photochromic diazocine units within the helicates.

**Figure 5:**
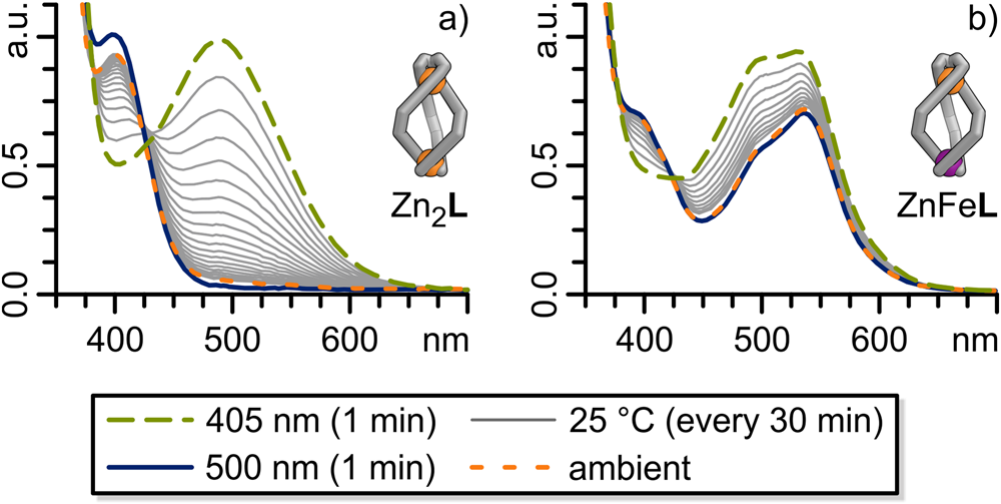
UV–vis spectra (0.03 mM, CH_3_CN, 25 °C) under ambient light and after 405 and 500 nm light irradiation, respectively, as well as thermal relaxation after 405 nm light irradiation for a) Zn_2_ **L** and b) ZnFe**L**.

**Figure 6:**
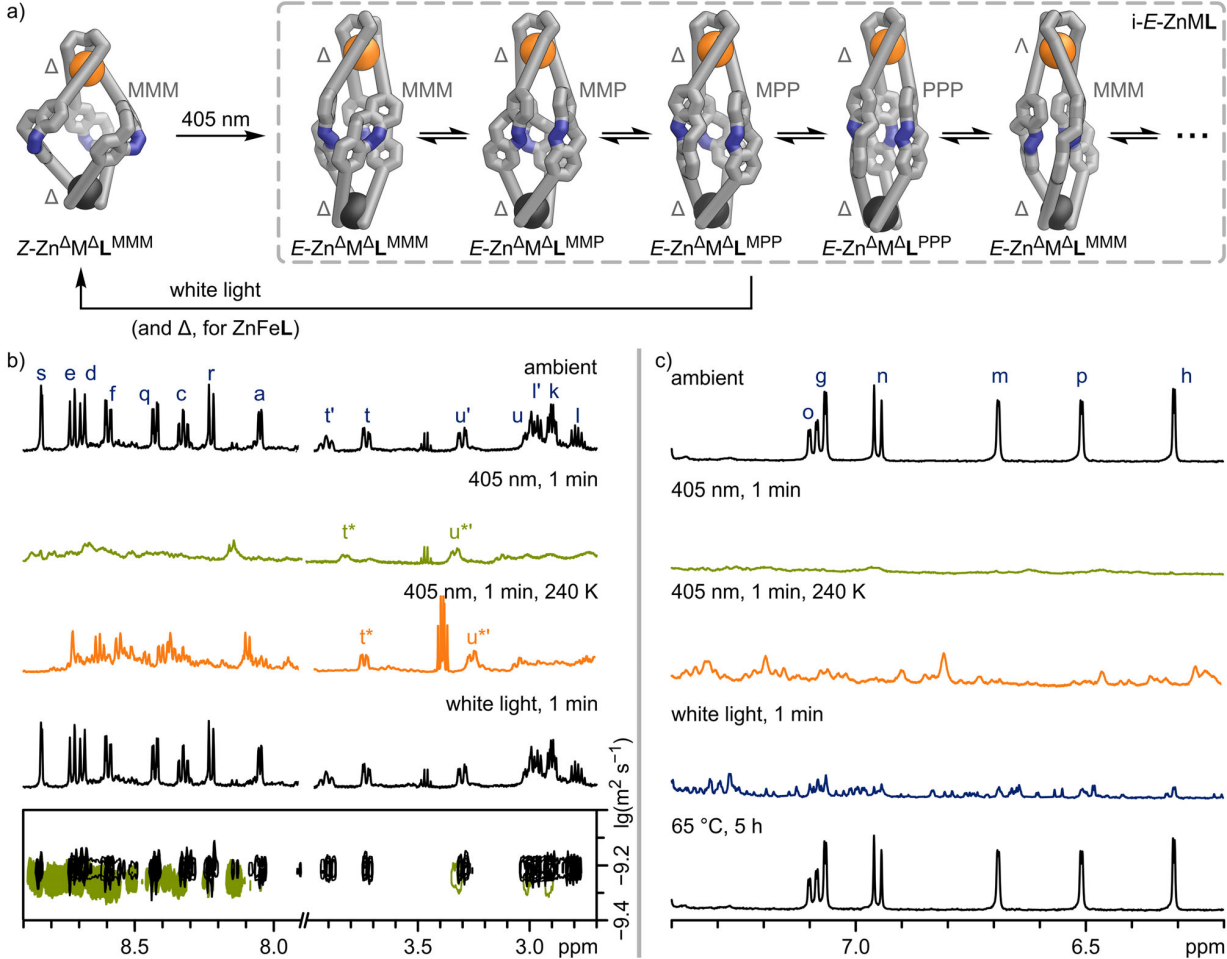
Photoswitching of Zn_2_ **L** and ZnFe**L** (ZnM**L**; stylised representations of GFN2‐xTB optimised structures). a) Photoisomerisation of *Z*‐ZnM**L** to a mixture of possible *E*‐ZnM**L** diastereomers (i‐*E*‐ZnM**L**). *Nomenclature* (looking along the M^TREN^–M^bipy^ axis) Δ/Λ: right‐/lefthanded helicity of the metal cation ligand environment; P/M: diazocine nitrogens pointing clockwise/anti‐clockwise. i: mixture of isomers of the same composition and ligand configuration (*E*/*Z*), i.e., i‐*E*‐ZnM**L** represents all states in the grey dashed box. b) ^1^H NMR spectra (500 MHz, CD_3_CN) of Zn_2_ **L** under ambient conditions (298 K, black), after 405 nm light irradiation (1 min; 298 K (green) and 240 K (orange), respectively), after white light irradiation (1 min, 298 K, black), and ^1^H DOSY NMR spectrum before and after 405 nm light irradiation (black and green, respectively, 298 K; top to bottom). c) ^1^H NMR spectra (500 MHz, CD_3_CN) of ZnFe**L** under ambient conditions (298 K, black), after 405 nm light irradiation (1 min; 298 K (green) and 240 K (orange), respectively), after white light irradiation (1 min, 298 K, blue), and after heating to 65 °C (5 h, 298 K, black; top to bottom).

Irradiation of an acetonitrile solution of Zn_2_ **L** with visible light (405 nm, 25 °C, 1 mM) resulted in a colour change from yellow to red, along with the disappearance of the 405 nm absorption band and the emergence of an intense absorption band at 490 nm in the UV–vis spectrum (Figure 5a). The changes in the UV–vis spectrum are nearly identical to those observed for aldehyde **1** (Figure S80), suggesting an equally efficient *Z→E* conversion in Zn_2_ **L** helicate as in free aldehyde **1** upon irradiation with 405 nm light. High‐resolution ESI mass spectrometry demonstrates that the Zn_2_ **L** composition remains intact following irradiation with 405 nm light (Figure S99). Additionally, ^1^H DOSY NMR spectroscopy indicated that the diffusion coefficients—and, therefore, the solvodynamic diameters—of the structures before and after 405 nm light irradiation differ only slightly (Figure 6b, bottom). In the ^1^H NMR spectrum, irradiation of Zn_2_ **L** with 405 nm light results in the disappearance of nearly all previously sharp and well‐defined signals, leaving only ill‐defined and broad signals, apart from the signals of the ethylene groups H‐t and H‐u. Moreover, no signals for the free ligand **L** or free aldehyde **1** were detected, indicating that at least the Zn^TREN^ binding pocket remained intact following irradiation (Figure 6b, green, 2^nd^ from top). The intact Zn^TREN^ binding pocket, along with high‐resolution ESI mass spectrometry confirming the retention of the Zn_2_ **L** composition, ^1^H DOSY NMR spectroscopy suggesting a structure of a similar solvodynamic diameter, and UV–vis spectroscopy demonstrating successful switching of the diazocine units, might indicate the formation of an *E*,*E*,*E*‐Zn_2_ **L** structure (abbreviated as *E*‐Zn_2_ **L**, Figure 6a). Although switching should occur in a stepwise manner, we tentatively assume structures in which only some of the diazocines are switched, namely *E*,*E*,*Z*‐Zn_2_ **L** and *E*,*Z*,*Z*‐Zn_2_ **L**, to be unlikely, as literature precedence^[^ ^14^, ^18^, ^58^, ^59^ ^]^ of cages containing photochromic units in their ligand backbone suggests that switching is a highly cooperative process, with intermediates only observed at extremely low temperatures.^[^ ^60^ ^]^ The first switching event causes the most significant structural change, potentially accelerating the subsequent switching of other photochromic units within the same structure to relieve strains caused by previous isomerisation events.

The observed broad ^1^H NMR signals after 405 nm irradiation of Zn_2_ **L** (Figure 6b, green, 2^nd^ from top) indicate structural transformations, which are on intermediate exchange on the NMR timescale. We tentatively assume these to be hindered rotations of the diazocine moieties within the arms of the ligands or isomerisation of the helicate to a pseudo‐mesocate. In *E*,*E*,*E*‐Zn_2_ **L**, the three “arms” of ligand **L** adopt a pseudo‐linear configuration, enabling rotation of the diazocine moieties (i‐*E*‐Zn_2_ **L**, Figure 6a, right; Figure S102).

Rotation of the diazocine moieties would give rise to four potential constantly interconverting rotamers with the diazocine units pointing with their azo‐nitrogens either in a clockwise (**L** ^PPP^) or counterclockwise direction (**L** ^MMM^) in the helicate or even mixtures of both (**L** ^PPM^ or **L** ^MMP^; Figure 6a). Additionally, due to steric strains within *E*‐Zn_2_ **L**, we anticipate that both Zn^II^ corners in *E*‐Zn_2_ **L** will be significantly weaker than those in *Z*‐Zn_2_ **L**, potentially promoting isomerisation between helicate and pseudo‐mesocate or even facilitating metal exchange.

To test our hypothesis regarding the interconversion of rotamers and helicate‐to‐pseudo‐mesocate leading to the broad signals in the ^1^H NMR spectrum after 405 nm irradiation of *Z*‐Zn_2_ **L**, we performed low‐temperature ^1^H NMR spectroscopy (240 K) on the same sample (Figure 6b, orange, 3^rd^ from top; Figure S97). The resulting spectrum reveals a multitude of sharp signals, indicating a plethora of chemical environments and isomeric structures, which do not interconvert at this temperature. This observation supports our hypothesis that multiple interconverting diastereomers (i‐*E*‐Zn_2_ **L**) are responsible for the broad signals observed at 298 K. Irradiation of i‐*E*‐Zn_2_ **L** with either 500 nm or white light reverses the process almost instantly, indicating that Zn_2_ **L** switching is completely reversible at ambient temperature (Figure 6b, black, 4^th^ from top). However, at 240 K, irradiation of i‐*E*‐Zn_2_ **L** with white light does not lead to the clean formation of *Z*‐Zn_2_ **L** but rather a multitude of sharp signals that indicate the formation of multiple isomers (Figure S97). Therefore, we tentatively assume that irradiation with white light causes all diazocine moieties to switch back into their *Z* states, with each i‐*E*‐Zn_2_ **L** helicate or pseudo‐mesocate rotamer isomerising to its corresponding i‐*Z*‐Zn_2_ **L** helicate or pseudo‐mesocate atropisomer. These atropisomers cannot interconvert without breaking at least one pair of Zn^II^‐bipy coordinative bonds. At 240 K, the abundance of atropisomers results in numerous sharp ^1^H NMR signals (Figure S97). At room temperature, the Zn^II^‐bipy coordinative bond readily dissociates, allowing the atropisomers to converge to the most stable *Z*‐Zn_2_ **L** structure (Figure S97). Thus, the isomerisation process *Z*‐Zn_2_ **L**→*E*‐Zn_2_ **L**→*Z*‐Zn_2_ **L** is influenced by the kinetic lability of the metal cation within the bipy pocket of **L**. To further explore the intricate *Z*‐Zn_2_ **L**→*E*‐Zn_2_ **L**→*Z*‐Zn_2_ **L** switching mechanism and support our hypothesis, we investigated switching in ZnFe**L**, which contains a kinetically more stable Fe^II^ in the bipy binding site of **L** (Fe^bipy^) and should, thus, hinder isomerisation of the atropisomers (i‐*Z*‐ZnFe**L**) to the thermodynamically preferred structure (*Z*‐Zn^Δ^Fe^Δ^ **L** ^MMM^).

### Photoswitching of ZnFe**L**: A Molecular Energy Ratchet

Similar to Zn_2_ **L**, the irradiation of ZnFe**L** with 405 nm light resulted in a ^1^H NMR spectrum featuring broad signals, while ^1^H DOSY NMR spectroscopy and high‐resolution ESI mass spectrometry indicated the formation of a species with the same composition and a similar size to *Z*‐ZnFe**L**, which we denote as i‐*E*‐ZnFe**L** (Figure 6; Sections S7.4 and S7.5). The UV–vis spectra of *Z*‐ZnFe**L** and *E*‐ZnFe**L** showed nearly the same differences as those between *Z*‐Zn_2_ **L** and *E*‐Zn_2_ **L**, as well as *Z*‐**1** and *E*‐**1**, with the MLCT absorption band remaining unchanged (Figure 5b; Figure S80). This suggests that photoswitching remains efficient, even in the presence of the Fe^bipy^ MLCT absorption band, and that both Fe^TREN^ and Fe^bipy^ coordination are preserved. As with i‐*E*‐Zn_2_ **L**, the observed broad ^1^H NMR signals for i‐*E*‐ZnFe**L** sharpened when cooled to 240 K (Figure 6c, orange, 3^rd^ from top), indicating that diazocine rotation and helicate→pseudo‐mesocate isomerisation caused the broadened signals at room temperature.

**Scheme 2:**
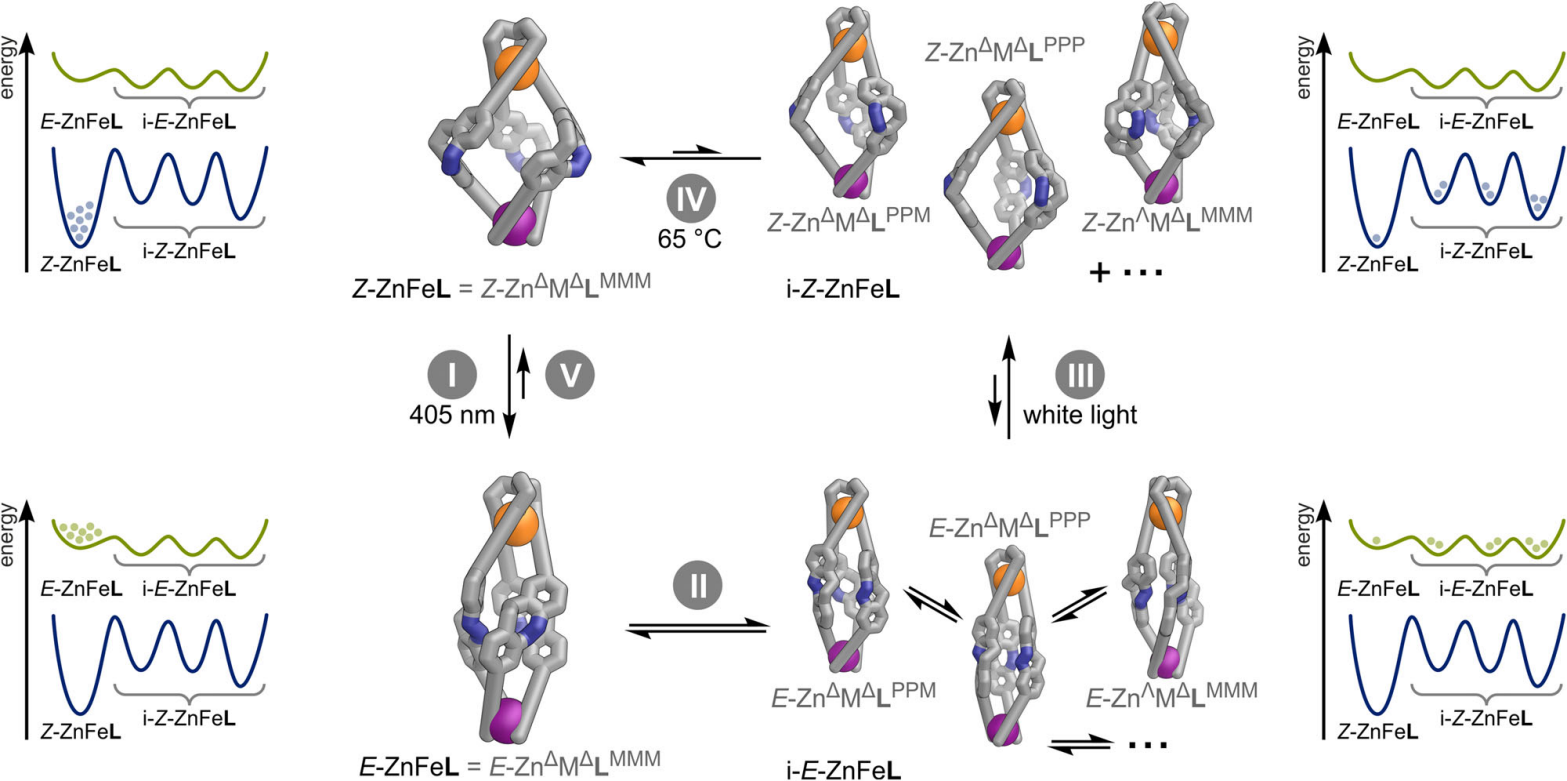
Chemical reaction network and schematic potential energy surfaces related to the formation of high‐energy diastereomers i‐*Z*‐ZnFe**L**, facilitated by a molecular ratchet mechanism driven by consecutive 405 nm and white light irradiation (stylised representations of GFN2‐xTB optimised structures). (I) *Z*→*E* photoisomerisation of *Z*‐ZnFe**L** to *E*‐ZnFe**L**. (II) Isomerisation of *E*‐ZnFe**L** via rotation of the diazocine moieties or helicity flipping of a metal vertex. (III) *E*→*Z* photoisomerization of different i‐*E*‐ZnFe**L** diastereomers, which are now kinetically trapped in their high‐energy states. (IV) Thermal relaxation from i‐*Z*‐ZnFe**L** isomers to *Z*‐ZnFe**L**.

As envisioned, irradiating i‐*E*‐ZnFe**L** with white light resulted in a complex ^1^H NMR spectrum featuring multiple signal sets (Figure 6c, blue, 4^th^ from top). This signal pattern is similar to that of i‐*E*‐Zn_2_ **L** following 405 nm and white light irradiation at 240 K, prompting us to label it i‐*Z*‐ZnFe**L** (Scheme 2; Figure S98). We can reform the thermodynamically stable *Z*‐ZnFe**L** by heating i‐*Z*‐ZnFe**L** at 65 °C for five hours (Figure 6c, black, 5^th^ from top). The formation of kinetically trapped states after 405 nm and white light irradiation at room temperature was also observed for Fe_2_ **L** (Figure S84), supporting our hypothesis that the dissociation of bipyridine units in the M^bipy^ coordination site is the rate‐determining step for the kinetically trapped states to revert to the thermodynamically favoured *Z*‐M^Δ^M^Δ^ **L** ^MMM^ (*Z*‐Zn^Δ^Zn^Δ^ **L** ^MMM^, *Z*‐Zn^Δ^Fe^Δ^ **L** ^MMM^, and *Z*‐Fe^Δ^Fe^Δ^ **L** ^MMM^, respectively). The stronger metal–ligand bond of Fe^II^ compared to Zn^II^ leads to a higher barrier for the dissociation of metal–bipyridine coordination, creating a more effective kinetic trap. This elevated barrier is evident in the temperature needed for the various helicate and pseudo‐mesocate atropisomers (i‐*Z*‐Zn_2_ **L**, i‐*Z*‐ZnFe**L**, and i‐*Z*‐Fe_2_ **L**) to return to their initial thermodynamically favoured states *Z*‐M^Δ^M^Δ^ **L** ^MMM^ (*Z*‐Zn^Δ^Zn^Δ^ **L** ^MMM^, *Z*‐Zn^Δ^Fe^Δ^ **L** ^MMM^, and *Z*‐Fe^Δ^Fe^Δ^ **L** ^MMM^, respectively), which rises from room temperature for i‐*Z*‐Zn_2_ **L** to 65 °C for i‐*Z*‐ZnFe**L** and i‐*Z*‐Fe_2_ **L**.

We examined the kinetically trapped intermediates during the switching cycle of ZnFe**L** through in situ illuminated ^1^H NMR spectroscopy (Section S7.4.3). Although the variety of signals hindered a thorough kinetic analysis, our findings indicate that irradiating ZnFe**L** with 405 nm light produces a mixture of at least two distinct species. This implies that the structural alterations induced by 405 nm light irradiation result in a distribution of conformational and possibly isomeric (helicate and pseudo‐mesocate) states. The notable asymmetry in these i‐*E*‐ZnFe**L** states is evident in the intricate ^1^H NMR spectrum.

From these findings, we conclude that photoswitchable helicate ZnFe**L** functions as a light‐driven molecular energy ratchet (Scheme 2):

- Irradiation of ZnFe**L** with 405 nm light causes all diazocine units within the structure to isomerise to their *E*‐configurations, forming symmetric *E*‐ZnFe**L** (*E*‐Zn^Δ^Fe^Δ^ **L** ^MMM^).
- In *E*‐ZnFe**L**, the diazocines can now rotate, albeit slowly, resulting in four rotamers (*E*‐Zn^Δ^Fe^Δ^ **L** ^MMM^, *E*‐Zn^Δ^Fe^Δ^ **L** ^PMM^, *E*‐Zn^Δ^Fe^Δ^ **L** ^PPM^, *E*‐Zn^Δ^Fe^Δ^ **L** ^PPP^; Figure 6a; Section S7.6). *E*‐ZnFe**L** is sterically strained with weakened coordinative bonds around the Zn^TREN^ and Fe^bipy^ corners, which allows for helicate→pseudo‐mesocate isomerisation, giving rise to four pseudo‐mesocate rotamers (*E*‐Zn^Λ^Fe^Δ^ **L** ^MMM^ etc.; Sections S6.3 and S8). Rotamer and pseudo‐mesocate formation from symmetric *E*‐Zn^Δ^Fe^Δ^ **L** ^MMM^ to i‐*E*‐ZnFe**L** occurs rapidly, indicating low energy differences and activation barriers between the states.
- Irradiating the isomeric mixture i‐*E*‐ZnFe**L** with 500 nm or white light causes all diazocine units within the supramolecular structures to isomerise to their *Z*‐configurations, trapping the respective helicates, pseudo‐mesocates, and their rotamers (i‐*Z*‐ZnFe**L**).
- Due to the kinetic inertness of Fe^II^, i‐*Z*‐ZnFe**L** represent kinetically trapped states that can revert to the thermodynamically favoured *Z*‐Zn^Δ^Fe^Δ^ **L** ^MMM^ by partial Fe^bipy^ coordination site dissociation at 65 °C, thus closing the reaction cycle.

Trapping the respective i‐*E*‐ZnFe**L** in their current isomeric forms through photoswitching provides a potential direct pathway from symmetric *E*‐Zn^Δ^Fe^Δ^ **L** ^MMM^ to ground state *Z*‐Zn^Δ^Fe^Δ^ **L** ^MMM^ (Scheme 2, process V).

In the case of Zn_2_ **L**, i‐*Z*‐Zn_2_ **L** are kinetically trapped at low temperatures (Figure 6b), demonstrating that the same processes operate during the irradiation of Zn_2_ **L**.

Since most molecules follow the reaction cycle unidirectionally (Scheme 2, processes I–IV), the photoswitching of ZnFe**L** acts as a light‐driven molecular energy ratchet: 405 nm light irradiation drives the thermodynamically stable *Z*‐ZnFe**L** helicate into metastable *E*‐ZnFe**L** helicates, which can readily access various conformers and stereoisomers (i‐*E*‐ZnFe**L**). Under white light irradiation, these i‐*E*‐ZnFe**L** states relax swiftly into their corresponding kinetically trapped i‐*Z*‐ZnFe**L** isomers. These higher‐energy kinetically trapped isomers (i‐*Z*‐ZnFe**L**) cannot readily return to the initial thermodynamically stable state (*Z*‐ZnFe**L**) due to the required rotation of the diazocine units being only possible through partial Fe^bipy^ coordination‐site dissociation. Thus, the system captures light energy to convert the more stable isomer (*Z*‐ZnFe**L**) into less stable, kinetically trapped isomers (i‐*Z*‐ZnFe**L**). This transformation leads to the occupancy of higher‐energy states, storing some of the absorbed light energy as strained or unfavourable molecular conformations.

### An Autonomously Operating Molecular Ratchet Under White Light Irradiation

**Figure 7:**
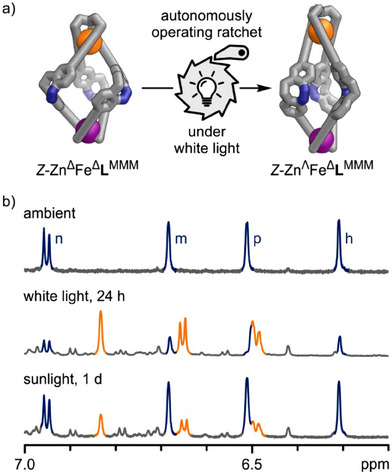
a) Continuous exposure to white light powers the ratchet autonomously, leading to the formation of a particular mesocate structure. (*Z*‐Zn^Λ^Fe^Δ^ **L** ^MMM^, as a racemate; stylised representations of GFN2‐xTB optimised structures). b) ^1^H NMR spectra (500 MHz, CD_3_CN, 298 K) of parent structure *Z*‐Zn^Δ^Fe^Δ^ **L** ^MMM^ under ambient conditions, after white light irradiation for 24 h, and after exposing the parent structure *Z*‐Zn^Δ^Fe^Δ^ **L** ^MMM^ to sunlight for one winter day (top to bottom). In all spectra post‐irradiation, parent helicate *Z*‐Zn^Δ^Fe^Δ^ **L** ^MMM^ (blue) and mesocate *Z*‐Zn^Λ^Fe^Δ^ **L** ^MMM^ (orange) could be identified.

Remarkably, this molecular energy ratchet can operate autonomously when exposed to white light, even though the photostationary state under such light is less than 1% *E*‐diazocine. Constant white light irradiation accumulates one specific structure (70% after 24 h; Figure 7). Using ^1^H NMR and ^1^H,^1^H ROESY NMR spectroscopy, we unambiguously determined the structure as the *Z*‐Zn^Λ^Fe^Δ^ **L** ^MMM^ pseudo‐mesocate (and its enantiomer). The observed proton–proton contacts between ligand **L**’s pyridine and diazocine phenyl rings (H‐f↔H‐h, H‐g↔H‐i, H‐m↔H‐p, H‐o↔H‐q) correspond to the ligand conformation within the *Z*‐Zn^Λ^Fe^Δ^ **L** ^MMM^ pseudo‐mesocate (for further details see Section S8). Although a photostationary state below 1% *E*‐diazocine enables efficient *E*→*Z* isomerisation on a macroscopic level, diazocine moieties and, consequently, ZnFe**L**, continually isomerise at a microscopic level. Given the properties of the molecular ratchet, a minimal quantity of *E*‐ZnFe**L** effectively propels the reaction cycle. Similar to a mechanical ratchet, each light‐triggered cycle advances the system by selectively confining it in energetically less favourable but kinetically stable states (i‐*Z*‐ZnFe**L**). Under continuous irradiation, the system converges into the most stable high‐energy configuration, identified as *Z*‐Zn^Λ^Fe^Δ^ **L** ^MMM^. This conversion into *Z*‐Zn^Λ^Fe^Δ^ **L** ^MMM^ could occur via two pathways:

- Photochemical *Z*→*E* isomerisation of a *Z*‐ZnFe**L** to an *E*‐ZnFe**L** isomer, which subsequently isomerises into the corresponding *E*‐Zn^Λ^Fe^Δ^ **L** ^MMM^ pseudo‐mesocate. *E*→*Z* isomerisation then yields *Z*‐Zn^Λ^Fe^Δ^ **L** ^MMM^.
- Slow thermal relaxation of less favourable high‐energy i‐*Z*‐ZnFe**L** isomers to relatively more favourable pseudo‐mesocate *Z*‐Zn^Λ^Fe^Δ^ **L** ^MMM^.

Kinetic studies on the thermal relaxation of the i‐*Z*‐ZnFe**L** mixture, obtained via subsequent 405 nm and white light irradiation, support the thermal relaxation pathway (ii). The time‐dependent ^1^H NMR spectra revealed that various components of this mixture were interconverting, with none of the involved species returning to the initial ground state of *Z*‐ZnFe**L** (*Z*‐Zn^Δ^Fe^Δ^ **L** ^MMM^; Section S7.4.4). Thermal conversion of pseudo‐mesocate *Z*‐Zn^Λ^Fe^Δ^ **L** ^MMM^ into the overall thermodynamically more stable *Z*‐Zn^Δ^Fe^Δ^ **L** ^MMM^ helicate occurred at room temperature, exhibiting a half‐life of several days (Figure S115).

Curiously, the molecular ratchet functions in sunlight as well. When a sample of *Z*‐Zn^Δ^Fe^Δ^ **L** ^MMM^ helicate is placed by a window for one winter day, it transforms into a mixture enriched with pseudo‐mesocate *Z*‐Zn^Λ^Fe^Δ^ **L** ^MMM^ (Figure 7b, bottom). However, this process is less efficient compared to using a white LED. Therefore, the molecular ratchet is capable of converting light into chemical energy and can even retain that energy for a limited duration.

### Metal Exchange

**Figure 8:**
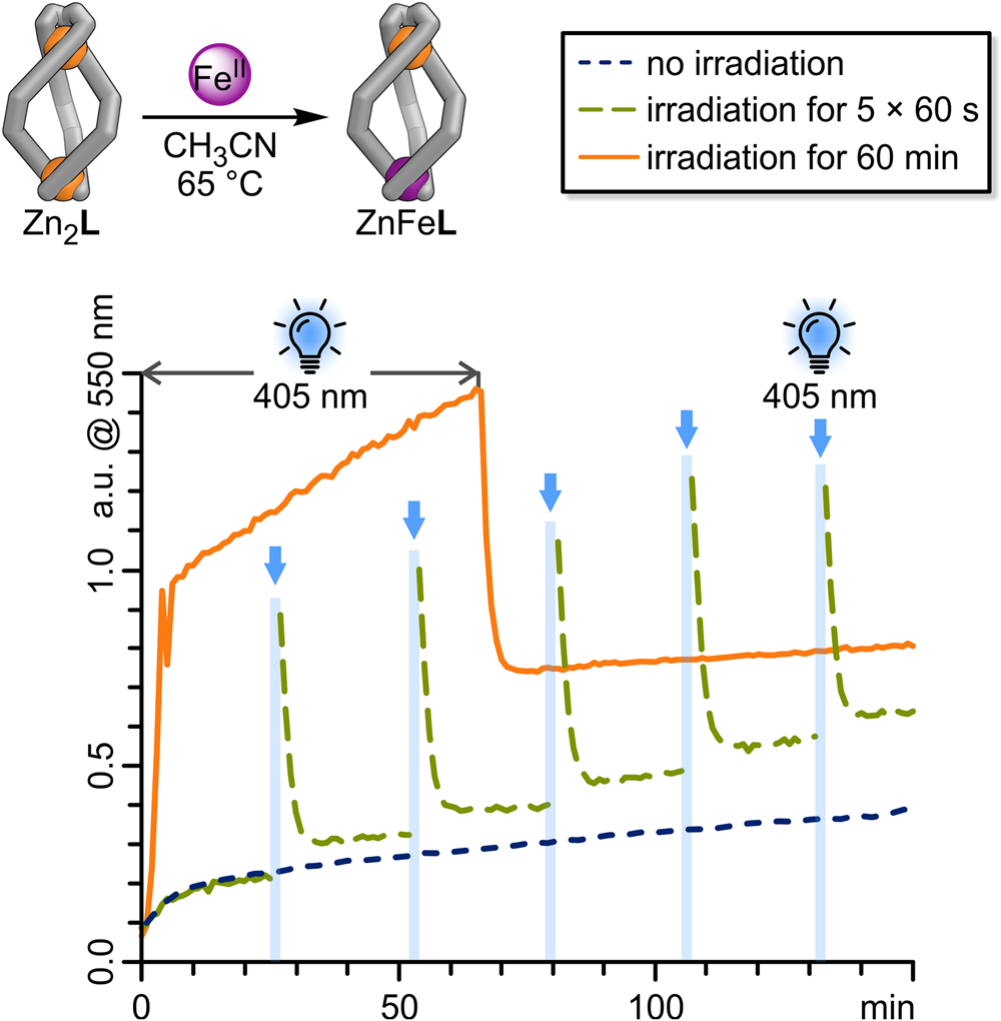
Zn^II^→Fe^II^ exchange transforming Zn_2_ **L** into ZnFe**L** by mixing Fe(OTf)_2_ (1.5 equiv.) and Zn_2_L at 65 °C. The reaction progress was monitored by following the Fe^bipy^ MLCT absorption band at 550 nm via UV–vis spectroscopy (CH_3_CN, 0.06 mM, 65 °C). Blue dotted line: background reaction without irradiation. Green dashed line: The sample was irradiated with 405 nm light for 60 s five times, as indicated by the blue arrows. Orange solid line: The sample was irradiated for the first 60 min of the measurement as indicated by grey arrows.

As mentioned earlier, ligand **L** contains two coordination sites with distinct kinetic stabilities, which we have shown to be essential for self‐sorting of heterobimetallic helicates. To take advantage of the differences in kinetic inertness between the two binding sites, we performed a selective metal‐cation exchange in the M^bipy^‐coordination site, transforming Zn_2_ **L** into ZnFe**L** via Zn^II^→Fe^II^ exchange (Figure 8, top). Specifically, treating an acetonitrile solution of Zn_2_ **L** with Fe(OTf)_2_ (1.5 equiv.) at 65 °C led to the formation of ZnFe**L**, confirmed by ^1^H NMR, UV‐vis spectroscopy, and ESI mass spectrometry (Section S9). Kinetic studies using ^1^H NMR spectroscopy (Section S9.3) indicated that the Zn^II^→Fe^II^ exchange does not follow apparent first‐order kinetics. Rather, Zn_2_ **L** is rapidly consumed, resulting in both the intended product ZnFe**L** and a non‐productive intermediate. This resting state is subsequently converted into the product ZnFe**L** via Zn_2_ **L** as an intermediate.

Given that the helicate molecular ratchet is accumulating high‐energy structures (i‐*E*‐ZnFe**L**), in which the M^bipy^ coordination site is likely weakened, we aimed to examine whether we could use this unidirectional reaction cycle (Scheme 2) to expedite the Zn^II^→Fe^II^ cation exchange within Zn_2_ **L**, resulting in the formation of ZnFe**L**. To evaluate this hypothesis, Fe(OTf)_2_ (1.5 equiv.) was added to an acetonitrile solution of Zn_2_ **L** at 65 °C, and the mixture was illuminated with 405 nm light for 60 seconds every 25 min. Since ZnFe**L** shows a distinct Fe^bipy^ MLCT transition band at 550 nm, which correlates with the number of Fe─N bonds formed and which Zn_2_ **L** entirely lacks (Figure 5), this reaction can also be monitored in situ using UV–vis spectroscopy (Figure 8). Note that *E*‐diazocines exhibit strong absorption at 550 nm (Figure S68), and this will influence the absorption band at 550 nm during irradiation, continuing until the diazocine thermally returns to its *Z*‐form. The thermal relaxation of the *E*‐diazocine moieties to their *Z*‐configurations occurs rapidly at 65 °C (*τ*_½_(65 °C) = 72 s), achieving complete conversion within less than 10 min. In the dark, the Zn_2_ **L**→ZnFe**L** background reaction (Figure 8, blue dotted line) shows a rapid initial rise in conversion before stabilising to a nearly linear reaction rate after about 12 min. Irradiating the Zn_2_ **L**→ZnFe**L** reaction mixture with 405 nm light outside the UV–vis spectrometer results in a significant increase in absorption at 550 nm (Figure 8, green dashed line), primarily attributed to the absorption of the *E*‐diazocine units. However, the observed increase in 550 nm absorption approximately 10 min after irradiation can be solely attributed to an increased Fe^bipy^ absorption, which reflects the Zn_2_ **L**→ZnFe**L** conversion. Four subsequent irradiations of the reaction mixture with 405 nm light further accelerate the conversion of Zn_2_ **L** to ZnFe**L**, resulting in nearly double the amount of ZnFe**L** produced compared to the background reaction after 150 min. The increased conversion of Zn^bipy^ to Fe^bipy^ after five 60‐s light irradiations of the ZnFe**L**‐Fe(OTf)_2_ reaction mixture reinforces our hypothesis that the molecular ratchet can help promote this exchange.

Notably, the Zn^bipy^→Fe^bipy^ exchange could also be accelerated under constant irradiation (60 min; Figure 8, orange solid line). Once the *E*‐diazocine moieties have reached their photostationary states, their absorption remains constant under continuous irradiation. Consequently, the significant fivefold steeper increase in absorption observed between minutes 10 and 60, compared to the background reaction, can be solely ascribed to an accelerated exchange from Zn^bipy^ to Fe^bipy^.

We believe that the molecular energy ratchet mechanism is vital for enabling the metal‐cation exchange process. By populating higher‐energy states and enriching non‐ideal conformers, the photoswitching process effectively destabilises the coordination sphere around the metal centres, thereby accelerating the Zn^bipy^→Fe^bipy^ exchange process. The ability to control this process through light irradiation offers a powerful tool for modulating kinetics in complex systems.

## Conclusion

To summarise, we have created a unique photoswitchable diazocine‐based ligand **L**, which self‐assembles into a range of photo‐responsive metallo‐supramolecular bimetallic helicates. The subtle balance between bond strength and kinetic lability at both the ligand coordination sites and the metal cations enabled us to form dinuclear heterobimetallic Zn^II^‐Fe^II^ and Zn^II^‐Co^II^ helicates with precise metal distribution through one‐pot self‐sorting reactions. We believe that this self‐sorting primarily involves a kinetic selection process regarding ligand **L**’s binding sites, meaning that the M^bipy^ coordination site is formed first. Additionally, it entails a thermodynamic selection for the metal cations, where the more thermodynamically stable Fe**1**_3_ (or Co**1**_3_) complex is favoured over the less stable Zn**1**_3_.

Importantly, we discovered that *Z*→*E* photoisomerisation of the diazo units in the metallo‐supramolecular Zn_2_ **L** and ZnFe**L** helicates initiates various conformational and stereochemical changes, all while preserving the overall constitutional integrity of the complexes. These conformers become trapped in higher energy isomers and atropisomers following *E*→*Z* isomerisation. We interpret the system as a molecular energy ratchet, capable of temporarily storing light energy by populating these energy‐rich isomers. The molecular ratchet could autonomously function under continuous white light exposure, efficiently converting light energy into chemical energy within a high‐energy *Z*‐ZnFe**L** pseudo‐mesocate.

The higher‐energy *E*‐isomers formed under light irradiation exhibit a more labile M^bipy^ coordination sphere, allowing the molecular ratchet to operate as a light‐controlled accelerator for the highly selective metal‐cation exchange process from homobimetallic Zn_2_ **L** to heterobimetallic ZnFe**L** helicate.

Our work enhances the structural complexity and functionality of photoswitchable complexes, highlighting the potential of using light to drive supramolecular systems out of equilibrium. This could open up new paths for developing light‐controlled molecular machines, stimuli‐responsive catalysis, and photo‐responsive building blocks for smart materials, potentially with customised energy storage and release characteristics.

## Supporting Information

The authors have cited additional references within the Supporting Information.^[^ ^61^, ^62^, ^63^, ^64^, ^65^, ^66^, ^67^, ^68^, ^69^, ^70^, ^71^, ^72^, ^73^, ^74^, ^75^, ^76^, ^77^, ^78^, ^79^, ^80^, ^81^, ^82^ ^]^

## Author Contributions

M. J. N.: Synthesis; characterisation; photoswitching studies; analysis of kinetics; calculations of molecular models; and project conception. G. S.: X‐ray crystallography. L. K. S. v. K.: Project conception and supervision.

## Conflict of Interests

The authors declare no conflict of interest.

## Supporting information



Supporting Information

Supporting Information

## Data Availability

The data that support the findings of this study are available in the supplementary material of this article. Deposition numbers 2442413 (for Fe_2_ **L**) and 2442414 (for ZnFe**L**) contain the supplementary crystallographic data for this paper. These data are provided free of charge by the joint Cambridge Crystallographic Data Centre and Fachinformationszentrum Karlsruhe Access Structures service (http://www.ccdc.cam.ac.uk/structures).
