# Supplementary material for: Light‐Driven Ratchet Mechanism Accelerates Regioselective Metal‐Cation Exchange in a Heterobimetallic Helicate
Source: Angew Chem Int Ed Engl. 2025 Jun 30;64(35):e202508952. doi: 10.1002/anie.202508952 (PMC12377437; doi:10.1002/anie.202508952)
Supplement: Supplementary file 1 — Supporting Information [file ANIE-64-e202508952-s002.pdf]

Supporting Information

for

Light-driven ratchet mechanism accelerates regioselective metal-cation exchange in a heterobimetallic helicate

Maximilian J. Notheis,[a] Gregor Schnakenburg,[b] and Larissa K. S. von Krbek*,[a]

a. Kekulé-Institut für Organische Chemie and Biochemie,
Rheinische Friedrich-Wilhelms-Universität Bonn,
Gerhard-Domagk-Str. 1, 53121 Bonn, Germany.
E-mail: larissa.vonkrbek@uni-bonn.de.

b. Institut für Anorganische Chemie,
Rheinische Friedrich-Wilhelms-Universität Bonn,
Gerhard-Domagk-Str. 1, 53121 Bonn, Germany.

Content

S1 General procedures **4**
S2 Ligand Synthesis **6**
S2.1 5-(4,4,5,5-tetraethyl-1,3,2-dioxaborolan-2-yl)-2,2'-bipyridine (**S3**) 7
S2.2 5-bromo-2-(1,3-dioxolan-2-yl)pyridine (**S5**) 10
S2.3 2-(1,3-dioxolan-2-yl)-5-(4,4,5,5-tetraethyl-1,3,2-dioxaborolan-2-yl)pyridine (**S6**) 11
S2.4 (*Z*)-8-([2,2'-bipyridin]-5-yl)-2-bromo-11,12-dihydrodibenzo[*c*,*g*][1,2] diazocine (**S7**) 14
S2.5 (*Z*)-2-(6-(1,3-dioxolan-2-yl)pyridin-3-yl)-8-([2,2'-bipyridin]-5-yl)-11,12-dihydrodibenzo[*c*,*g*][1,2]diazocine (**S8**) 17
S2.6 (*Z*)-5-(8-([2,2'-bipyridin]-5-yl)-11,12-dihydrodibenzo[*c*,*g*][1,2]diazocin-2-yl)picolinaldehyde (**1**) 20
S3 Synthesis of the bimetallic helicates **23**
S3.1 Use of stock solutions 23
S3.2 General procedure 23
S3.3 Zn_2**L** 25
S3.4 Fe_2**L** 30
S3.5 Co_2**L** 36
S3.6 Fe**L** 38
S3.7 FeZn**L** 40
S3.8 ZnFe**L** via Zn→Fe exchange 43
S4 Heterobimetallic self-sorting **48**
S4.1 Experimental procedures 48
S4.1.1 ZnFe**L** 48
S4.1.2 ZnCo**L** 50
S4.1.3 CoFe**L** 52
S4.2 Overview of one-pot self-sorting results 54
S4.3 CoFe**L** – UV-vis 55
S4.4 ZnCo**L** and CoFe**L** – ^{1}H NMR 56
S5 UV-vis kinetics of the complexation reactions **57**
S6 Structural characterization of the complexes **61**
S6.1 A note on the helicate nomenclature used 61
S6.2 X-ray crystallography 63
S6.3 Quantum chemical structure optimizations 67
S6.3.1 Structures of Zn_2**L** with unidirectional diazocine orientation within the ligand arms in the *Z* state 67
S6.3.2 Structures of Zn_2**L** with other ligand configurations 68
S6.3.3 Structures of complexes with metals other than zinc 70
S6.3.4 Comment on energy calculations on metal-organic helicates 71

S6.4 Determination of solution stereochemistry of the helicates 72
S6.4.1 1D ROESY NMR of ZnFe**L** 74
S6.4.1 2D ROESY NMR of redissolved Fe_2**L** single crystals 76
S6.4.2 2D ROESY NMR of Zn_2**L** 77
S7 Photochemical characterisation 78
S7.1 General procedures for the illumination of different types of samples 78
S7.2 Geometry changes of aldehyde **1** during photoswitching 81
S7.3 Photoswitching and thermal relaxation (UV-vis) 81
S7.3.1 Aldehyde subcomponent **1** 82
S7.3.2 Zn_2**L** 83
S7.3.3 Co_2**L** 84
S7.3.4 Fe_2**L** 85
S7.3.5 ZnFe**L** 86
S7.3.6 Photochemical fatigue of Zn_2**L** 87
S7.3.7 Difference in UV-vis spectra before and after photoswitching 88
S7.4 Photoswitching (^{1}H NMR) 89
S7.4.1 Photostationary states (*ex-situ* illumination) 89
S7.4.2 DOSY NMR of switched states 92
S7.4.3 Photoswitching of ZnFe**L** followed by *in-situ* illumination NMR 96
S7.4.4 NMR kinetics of reforming ZnFe**L** from kinetically trapped state i-*Z*-ZnFe**L** 98
S7.4.5 Low-temperature NMR of the switched states 101
S7.5 Photoswitching of helicates investigated by ESI$^+$ MS 103
S7.6 Possible isomerisations observed during photoswitching 105
S8 Irradiation of ZnFeL with full spectrum light for long periods 106
S9 Metal Exchange 115
S9.1 Thermal relaxation of ZnFe**L** at 65 °C for reference 115
S9.2 Metal-exchange kinetics by UV-Vis 115
S9.3 Metal-exchange kinetics by NMR 117
S9.4 Stability of ZnFe**L** and metal scrambling 120
S10 Dilution experiment for Zn_2L 121
S11 Literature 122

S1 General procedures

Reagents and materials. Commercial solvents and reagents were obtained from the following suppliers and used without further purification unless specified otherwise: Sigma Aldrich, Alfa Aesar, abcr, Acros Organics, BLD-Pharm, Fluorochem, Merck, TCI, Carbolution, Thermo Fisher Scientific.

Dry solvents (acetonitrile, tetrahydrofuran, methanol) were dried using an MP-SPS 800 (MBraun) drying apparatus. Solvents used for column chromatography were distilled at atmospheric pressure prior to use.

Schlenk techniques. All reactions using chemicals sensitive to air were carried out under argon using established Schlenk techniques. If chemicals were also sensitive to moisture, glassware was flame-dried prior to use.

Column chromatography. Column chromatography was carried out either using a puriFlash 5.020 (Interchim) flash chromatography machine with PuriFlash 15 µm Si HP (Interchim) flash cartridges or by hand using Silica gel ultra pure (Thermo scientific, 60 µm).

NMR spectroscopy. All NMR spectroscopic measurements were carried out using 300 MHz, 400 MHz, 500 MHz or 700 MHz spectrometers (Bruker Avance I 300, Bruker Avance I 400, Bruker Avance I 500, Bruker Avance III HD Prodigy 500, Bruker Avance III HD Ascend 700). ^{1}H and ^{13}C NMR spectra are referenced to the residual solvent peak for CD_3CN (^{1}H: 1.94 ppm, ^{13}C: 1.32 ppm), CD_2Cl_2 (^{1}H: 5.32 ppm, ^{13}C: 53.5 ppm) or $CDCl_3$ (^{1}H: 7.26 ppm, ^{13}C: 77.16 ppm). NMR signals are reported in terms of chemical shift (δ) in ppm, relative integral, multiplicity, coupling constants (in Hz) and assignment, in that order. The following abbreviations for multiplicity are used: s, singlet; d, doublet; t, triplet; qu, quartet; qn, quintet; m, multiplet; br, broad. Spectra were digitally processed (phase and baseline corrections, integration, peak analysis) using MestReNova 14.2.1 (Mestrelab) and TopSpin 4.05 (BrukerBioSpin). All processing operations were manually checked to ensure that the processed spectra accurately represented the raw data.

Mass spectrometry. Mass spectra were acquired using an Orbitrap XL (Thermo Fischer Scientific) and evaluated and plotted using XCalibur 4.2 (Thermo Fischer Scientific).

UV-vis spectroscopy. UV-vis spectroscopy was carried out using a Cary 60 (Agilent) utilizing a flash lamp style spectrometer with a temperature-controlled sample holder.

FTIR spectroscopy. FTIR spectroscopy was carried out using a Nicolet Summit (Thermo Fisher). A droplet of the sample solution was placed on the measurement window (ATR crystal) and left to dry on air and a spectrum of the resulting thin film was measured.

Elemental analysis. Elemental analysis was carried out using a Vario Micro Cube (Elementar). Approximately 2 mg of compound were used, and all measurements were carried out in duplicate.

HPLC analysis. HPLC chromatography was carried out using a PLATINblue (Knauer) system using a Nucleodur 100-3 Gravity C18 column (3 µm; 2,0 x 100 mm, Machery Nagel).

Light sources. Illumination experiments (NMR and MS) were carried out using portable built-in-house light sources incorporating commercial LED chips (Table S1, for further details see Section S7.1). *In-situ* illumination experiments (NMR and UV-vis) were carried out using *Prizmatrix* fibre collimated LEDs (Table S2, for further details see Section S7.1).

Table S1. Light output of non-collimated LEDs.

**λ** [nm]	**385**	**405**	**515**	**white**
**Manufacturer**	Nichia	Nichia	Roithner LaserTechnik GmbH	Nichia
**Type**	NVSU233B(T) – U385	NVSU233B(T) - U405	SMB1N-515V-02	NVSWE21AT
**Light output** (as specified by manufacturer)	1.7 W	370 mW	250 mW	297 lm

Table S2. Light output of fibre-collimated LEDs.

**λ** [nm]	**365**	**390**	**405**	**430**	**450**	**500**	**590**	**660**	**white**
**Output power** [mW] (1 m optical fibre)	170	220	260	160	440	165	230	140	74

S2 Ligand Synthesis

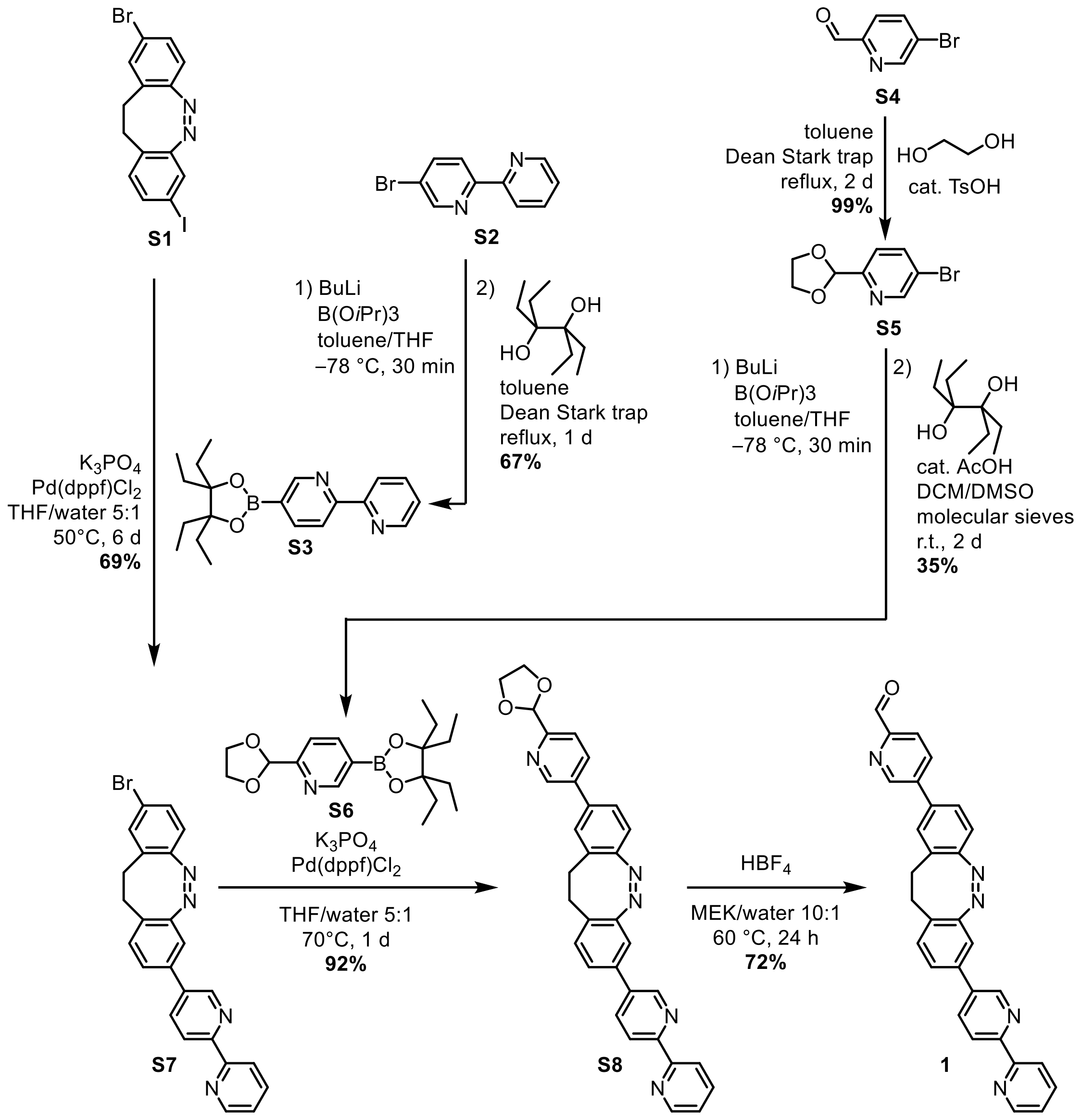

Scheme S1. Synthesis of aldehyde **1** from asymmetric diazocine **S1**, which was previously reported by our group.[1]

S2.1 5-(4,4,5,5-tetraethyl-1,3,2-dioxaborolan-2-yl)-2,2'-bipyridine (**S3**)

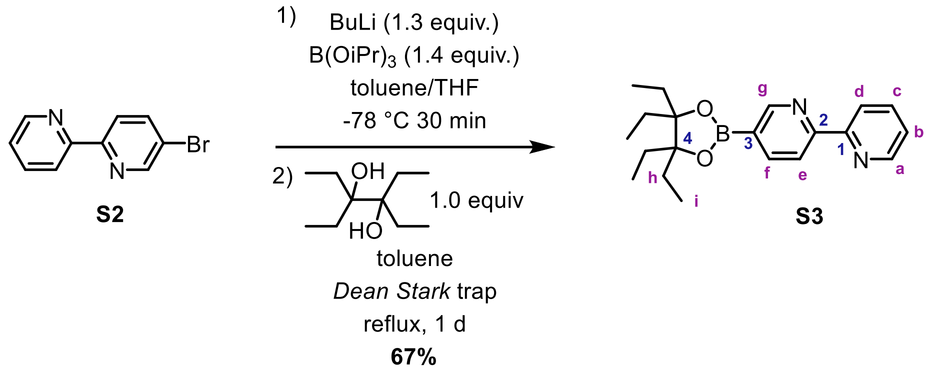

The borylation was carried out following a modified procedure for the borylation of a pyridine acetal.[2]

Under an argon atmosphere, in a heat-gun dried round bottom flask, dry toluene (20 mL) was cooled to −78 °C in an acetone/dry ice bath and *n*-Butyl lithium (2.5 M in hexanes, 5.53 mL, 13.82 mmol, 1.3 equiv.) was added to the cod toluene. A solution of 5-bromo-2,2′-bipyridine (**S2**, 2.50 g, 10.63 mmol, 1.0 equiv.) in 9 mL of dry toluene was added dropwise to the *n*-butyl lithium solution at −78 °C and the mixture was stirred for 15 minutes. Then dry THF (7 mL) was added and the mixture was stirred at −78 °C for 10 minutes. Triisopropylborate (3.41 mL, 14.89 mmol,1.4 equiv.) was added dropwise to the mixture andhe reaction mixture was stirred for an additional 15 minutes at −78 °C. The cooling bath was removed and the reaction was allowed to warm up under stirring for 15 minutes. Then NaOH (40 mL, 3.5 M in water) were added to the reaction mixture, which turned red upon addition of the base. It was allowed to warm up to room temperature and the aqueous phase was acidified to pH = 4 by the addition of HCl (4 M in water) and then extracted with a mixture of DCM/MeOH (10:1 (v/v), 3 × 200 mL). The combined organic layers were dried over magnesium sulfate and the solids were filtered off. The solvent was removed on a rotary evaporator to afford [2,2′-bipyridin]-5-ylboronic acid, which was subjected to the next reaction step without further purification.

The crude [2,2′-bipyridin]-5-ylboronic acid (2.13 g, 10.63 mmol, 1.0 equiv.) and 3,4-diethylhexane-3,4-diol (2.01 mL, 10.63 mmol, 1.0 equiv.) were added to a round bottom flask equipped with a *Dean-Stark* apparatus. The solids were suspended in of

toluene (30 mL) and the reaction was heated to reflux for 24 hours. Approximately 1 mL of water was collected in the trap over the course of the reaction. All volatiles were removed on a rotary evaporator and the oily residue was purified via column chromatography (silica gel, dichloromethane/ethyl acetate 100:0→50:50 (v/v) over 15 column volumes). Product **S3** was obtained as a brown oil with an overall yield of 67% (2.40 g,7.09 mmol).

R_F value (dichloromethane/ethyl acetate; 1:1 (v/v)) = 0.68

^{1}HNMR(500 MHz, CD_2Cl_2, 298 K): δ [ppm] = 0.99 (t, *J* = 7.5 Hz, 12H, H-i), 1.86 - 1.73 (m, 8H, H-h), 7.33 (ddd, *J* = 7.5, 4.8, 1.2 Hz, 1H, H-b), 7.83 (ddd, *J* = 8.0, 7.5, 1.8 Hz, 1H, H-c), 8.17 (dd, *J* = 7.9, 1.8 Hz, 1H, H-f), 8.41 (dd, *J* = 7.9, 1.0 Hz, 1H, H-g), 8.47 (dt, *J* = 8.0, 1.1 Hz, 1H, H-d), 8.67 (ddd, *J* = 4.8, 1.8, 0.9 Hz, 1H, H-a), 8.97 (dd, *J* = 1.8, 1.0 Hz, 1H, H-e).

^{13}C NMR (126 MHz, CD_2Cl_2, 298 K): δ [ppm] = 9.01 (C-i), 26.83 (C-h), 89.71 (C-4), 120.33 (C-g), 121.58 (C-d), 124.35 (C-b), 137.21 (C-c), 143.41 (C-f), 149.60 (C-a), 155.33 (C-e), 156.47 (C-1), 158.29 (C-2).

Due to the proximity to the boron atom the signal for C-3 is not visible.

HRMS (ESI$^+$ Orbitrap): *m*/*z* (relative intensity) = 339.2239 (100%, $[M+H]^+$, calcd. 339.2242), 361.205 (5%, $[M+Na]^+$, calcd. 361.206).

FTIR (ATR, thin film from CD_2Cl_2: $\tilde{\nu}$ [cm^{-1}] = 751.8 (m), 800.8 (vw), 826.2 (vw), 861.3 (vw), 918.4 (s), 954.5 (w), 1023.2 (w), 1102.7 (vs), 1292.2 (m), 1365.7 (vs), 1459.8 (m), 1546.4 (w), 1595.2 (s), 2883.0 (vw), 2942.7 (w), 2973.2 (m).

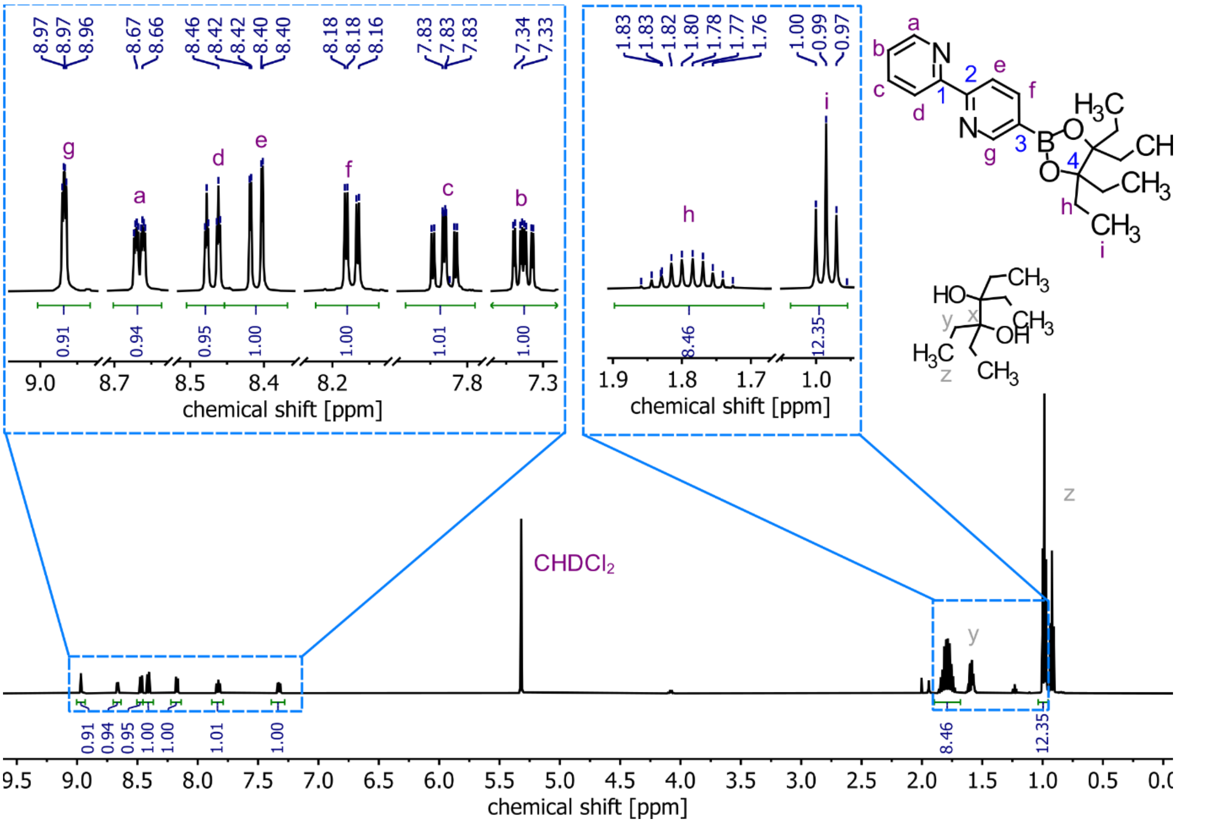

Figure S1. ^{1}H NMR spectrum (500 MHz, CD_2Cl_2, 298 K) of EPin ester **S3**. Signals denoted with y and z correspond to 3,4-diethylhexane-3,4-diol, which could be successfully removed after the next reaction step.

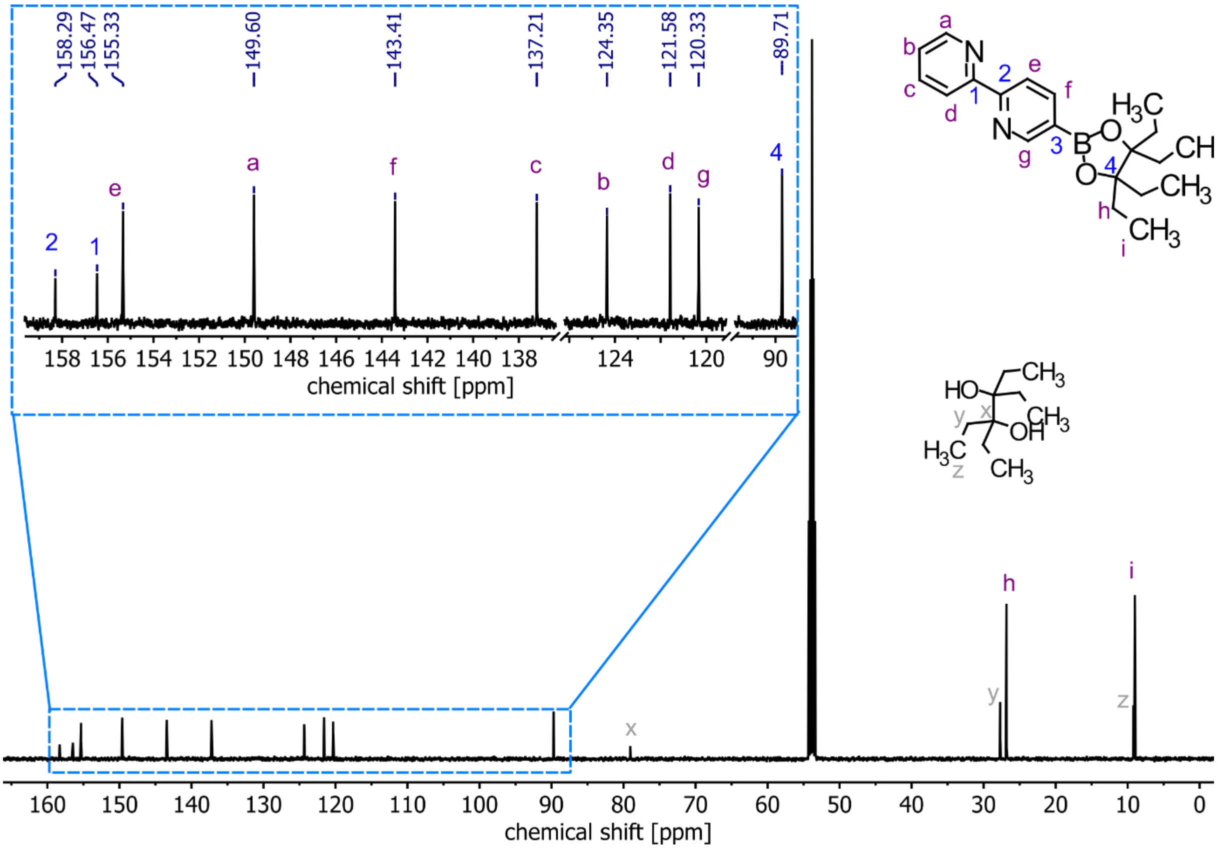

Figure S2. ^{13}C NMR spectrum (126 MHz, CD_2Cl_2, 298 K) of EPin ester **S3**. Signals denoted with x, y, and z correspond to 3,4-diethylhexane-3,4-diol, which could be successfully removed after the next reaction step.

S2.2 5-bromo-2-(1,3-dioxolan-2-yl)pyridine (**S5**)

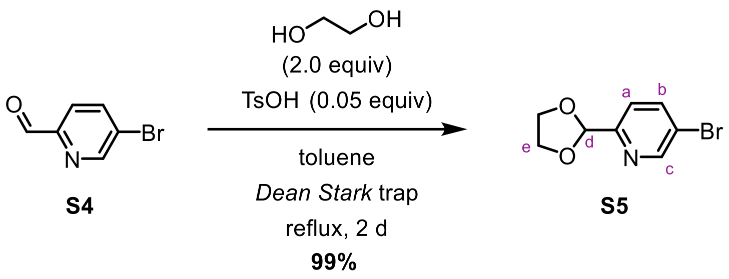

The acetal protection was carried out following a literature procedure.[3]

5-Bromopicolinaldehyde (**S4**, 5.00 g, 26.9 mmol, 1.00 equiv.), ethylene glycol (3.00 mL, 3.34 g, 54.8 mmol, 2.00 equiv.), and *p*-toluenesulfonic acid monohydrate (0.26 g, 1.3 mmol, 0.05 equiv.) were added to a round bottom flask equipped with a *Dean-Stark* apparatus. The solids were suspended in toluene (100 mL) and the reaction was heated to reflux for 2 days. Approximately 2 mL of water were collected in the trap over the course of the reaction. The reaction mixture was extracted with saturated aqueous sodium bicarbonate solution (2 × 50 mL). The organic phase was dried with magnesium sulfate, and all volatiles were removed under reduced pressure to afford product **S5** as a pale-yellow oil in 99% yield (6.10 g, 26.9 mmol).

^{1}H NMR (400 MHz, $CDCl_3$) δ [ppm] = 4.02 – 4.21 (m, 4H, H-e), 5.83 (s, 1H, H-d), 7.44 (dd, J = 8.3, 0.7 Hz, 1H, H-a), 7.87 (dd, J = 8.3, 2.3 Hz, 1H, H-b), 8.68 (dd, J = 2.3, 0.8 Hz, 1H, H-c).

^{1}H NMR data is in agreement with literature reported data.[3]

S2.3 2-(1,3-dioxolan-2-yl)-5-(4,4,5,5-tetraethyl-1,3,2-dioxaborolan-2-yl)pyridine (**S6**)

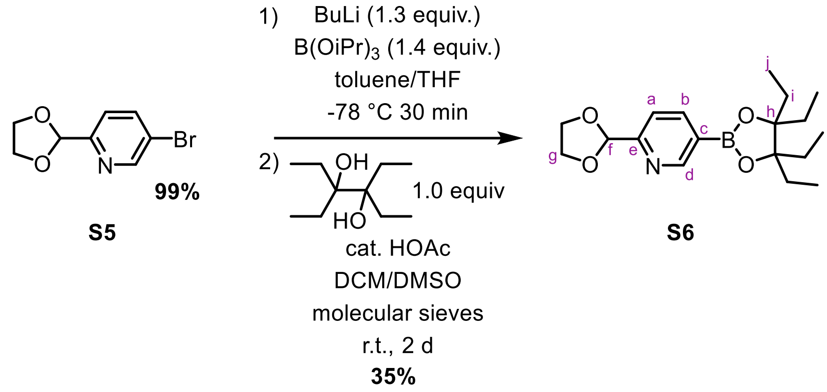

The borylation was carried out following a literature procedure[2] and the esterification following a modified procedure for the esterification of pyridyl boronic acids.[4]

Under an argon atmosphere, in a heat-gun dried round bottom flask, dry toluene (40 mL) was cooled to −78 °C in an acetone/dry ice bath and *n*-butyl lithium (2.5 M in hexanes, 11.2 mL,1.79 g, 28.0 mmol, 1.30 equiv.) was added to the cold toluene. A solution of protected bromo-pyridine (**S5**, 4.96 g, 21.6 mmol, 1.00 equiv.) in of dry toluene (10 mL) was added dropwise to the *n*-butyl lithium solution at −78 °C and the mixture was subsequently stirred for 15 minutes. Dry THF (15 mL) was added to the reaction mixture and the mixture was stirred for 10 minutes at −78 °C. Triisopropylborate (7.17 mL, 31.2 mmol,1.45 equiv.) was added dropwise to the reaction mixture and the reaction mixture was stirred for an additional 15 minutes at −78 °C. The cooling bath was removed, and the reaction was allowed to warm up under stirring for 15 minutes. Then aqueous NaOH solution (40 mL, 3.5 M) was added to the reaction mixture, upon which the reaction mixture turned red. After the addition was completed, the still-cold reaction mixture was brought to room temperature. The aqueous phase was acidified to pH = 4 by the addition of HCl (4 M in water) and then extracted with a mixture of DCM/MeOH (10:1 (v/v), 3 × 200 mL). The combined organic layers were dried over magnesium sulfate and the solids were filtered off. The solvent was removed on a rotary evaporator to afford the crude boronic acid (2.96 g, approx. 15.2 mmol). This was used without further purification in the next step.

The crude boronic acid (2.96 g, approx. 15.2 mmol, approx. 1.00 equiv.), 3,4-diethylhexane-3,4-diol (2.87 mL, 15.18 mmol, 1.00 equiv.), and acetic acid (0.09 g,

1.5 mmol, 0.10 equiv.) dissolved in a mixture of dry dimethylsulfoxide and dry dichloromethane (1:2 (v:v), 150 mL). 3 Å Molecular sieves (approx. 10 g) were added, the reaction mixture was purged with argon for 5 minutes, and the mixture was stirred at room temperature for 2 days. The reaction mixture was extracted with a mixture of saturated aqueous NaCl solution and water (1:3 (v/v), 3 × 200 mL). The organic phase was dried over magnesium sulfate. All volatiles were removed on a rotary evaporator and the residue was purified via column chromatography (silica gel, dichloromethane/ethyl acetate 100:0→70:30 (v/v) over 15 column volumes). Boronic ester **S6** was obtained as a brown oil with an overall yield of 35% (2.39 g, 7.16 mmol).

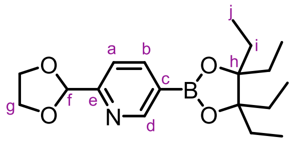

R_F value (dichloromethane/ethyl acetate 4:1 (v/v)) = 0.52.

^{1}H NMR (499 MHz, CD_2Cl_2) δ [ppm] = 0.96 (t, J = 7.5 Hz, 12H, H-j), 1.69 – 1.85 (m, 8H, H-i), 3.99 – 4.18 (m, 4H, H-g), 5.79 (s, 1H, H-6), 7.50 (dd, J = 7.7, 1.0 Hz, 1H, H-a), 8.09 (dd, J = 7.8, 1.7 Hz, 1H, H-b), 8.87 (dd, J = 1.7, 1.0 Hz, 1H, H-d).

^{13}C NMR* (126 MHz, CD_2Cl_2) δ [ppm] = 9.0 (C-j), 26.8(C-i), 66.0 (C-h), 89.8 (C-g), 104.3 (C -f), 120.1 (C-a), 143.3 (C-b), 155.2 (C-d), 159.9 (C-e).

HRMS (ESI$^+$ Orbitrap): m/z (relative intensity) = 333.2217 (100%, $[M+H]^+$, calcd. 333.2220).

FTIR (ATR, thin film from CD_2Cl_2): $\tilde{\nu}$ [cm^{-1}] = 401.9 (vw), 632.8 (vw), 665.2 (w), 771.4 (w), 811.6 (w), 843.3 (w), 917.2 (s), 1024.5 (s), 1099.5 (vs), 1290.3 (m), 1366.8 (vs), 1458.6 (m), 1561.1 (vw), 1599.8 (m), 2883.7 (m), 2974.5 (w).

* Due to the proximity to the boron atom, the signal for C-c is not resolved.

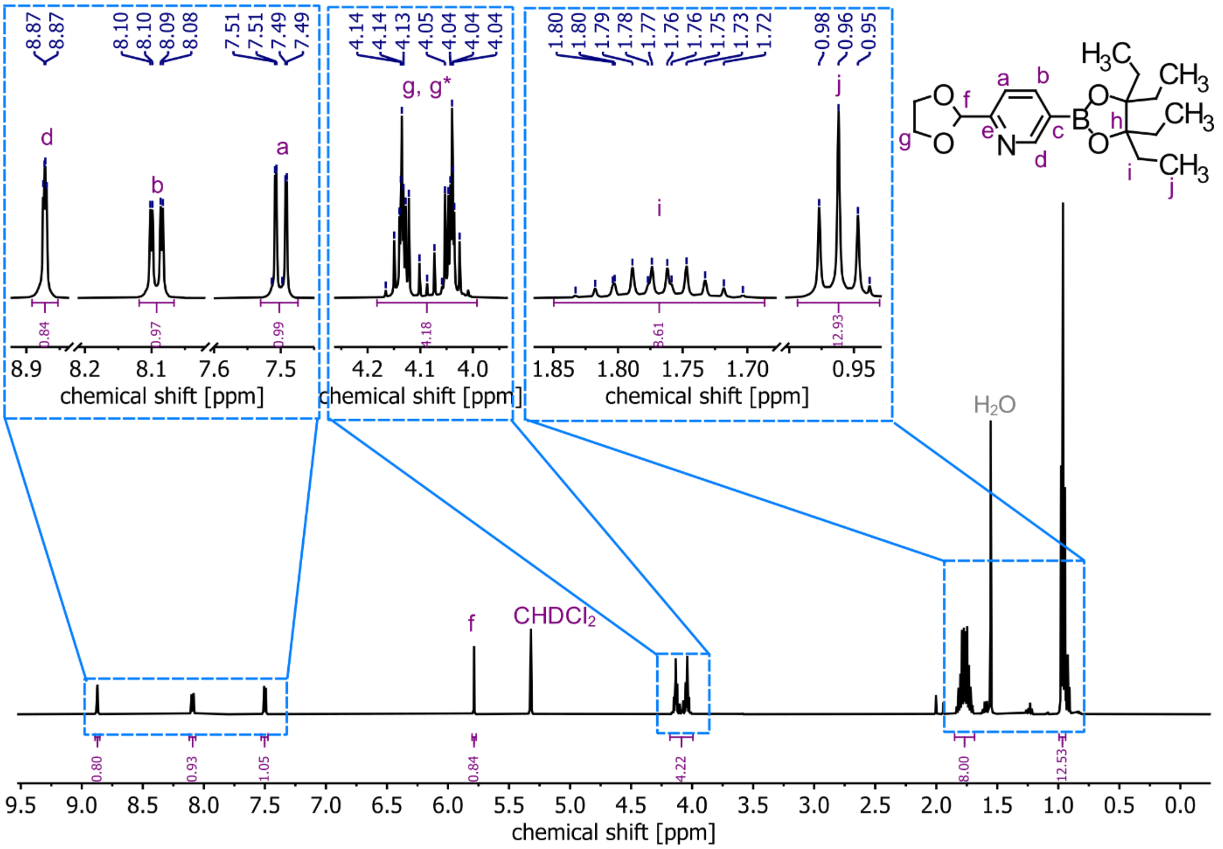

Figure S3. ^{1}H NMR spectrum (500 MHz, CD_2Cl_2, 298 K) of EPin ester **S6**.

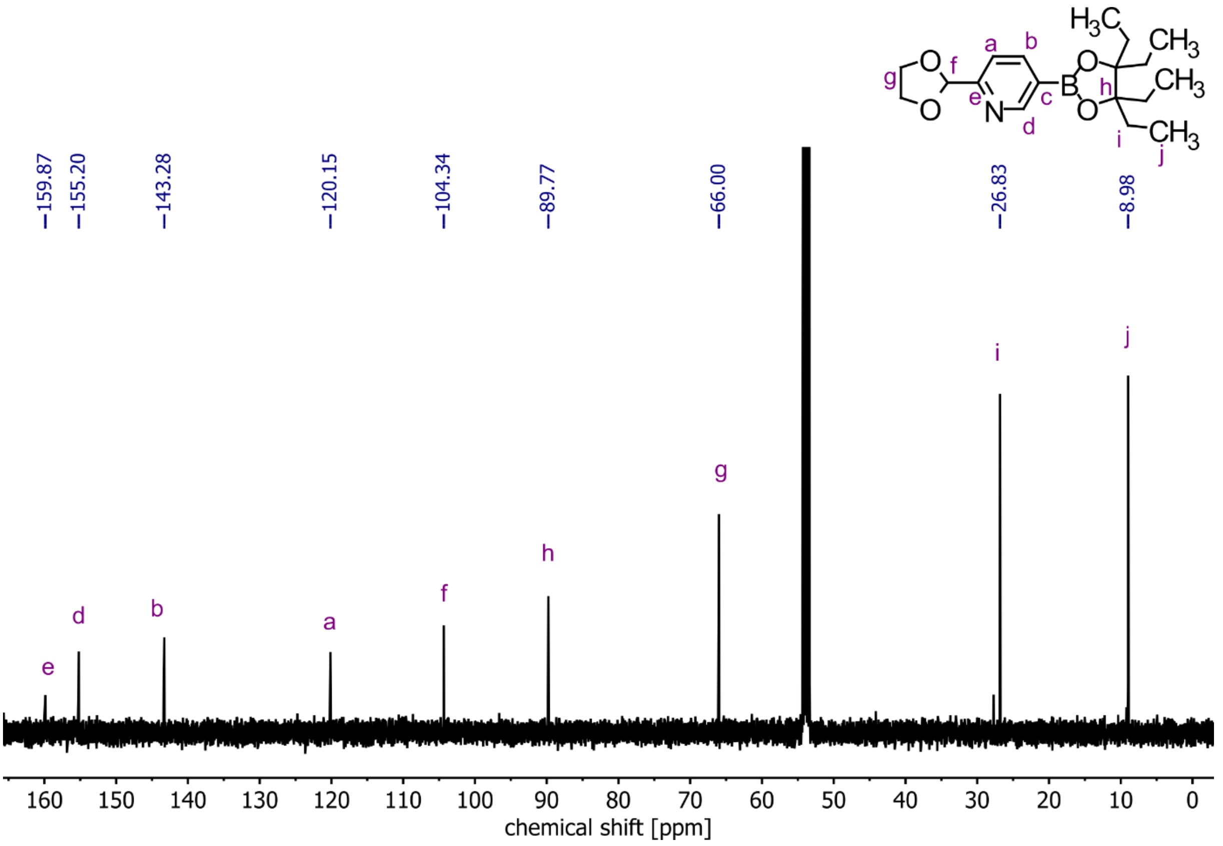

Figure S4. ^{13}C NMR spectrum (126 MHz, CD_2Cl_2, 298 K) of EPin ester **S6**.

S2.4 (*Z*)-8-([2,2'-bipyridin]-5-yl)-2-bromo-11,12-dihydrodibenzo[*c*,*g*][1,2] diazocine (**S7**)

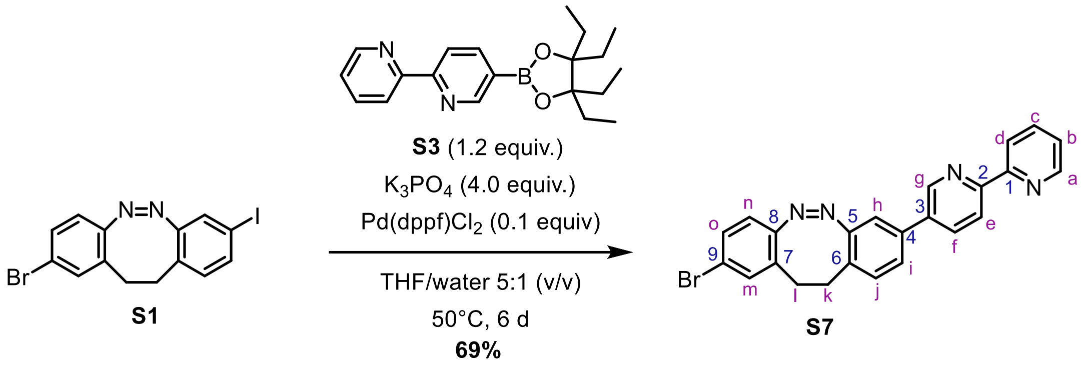

Diazocine **S1**[1] (200 mg, 484 µmol, 1.0 equiv.), boronic ester **S3** (197 mg, 581 µmol, 1.2 equiv.), potassium phosphate (257 mg, 1.21 mmol, 4.0 equiv.), Pd(dppf)Cl_2, (35 mg, 48 µmol, 0.1 equiv.), and a magnetic stirring bar were added into a screw cap vial. The open vial was transferred into an argon-filled oxygen-free glovebox and a deoxygenated tetrahydrofuran/water mixture (5:1 (v/v), 3 mL) was added. The vial containing the reaction mixture was closed and removed from the glovebox. The reaction mixture was stirred at 50 °C for 6 days under the vial's remaining Argon atmosphere. After cooling to room temperature, water (10 mL) and ethyl acetate (20 mL) were added to the reaction mixture and the phases were separated. The organic phase was washed with water (1 × 15 mL) and saturated aqueous NaCl solution (1 × 15 mL) and dried over magnesium sulfate. After all volatiles were removed by rotary evaporation, the residue was subjected to column chromatography (silica gel, cyclohexane/ethyl acetate 100:0 → 0:100 (v/v) over 18 column volumes) to afford product **S7** as a yellow solid in 69% yield (146 mg, 331 µmol).

R_F value (dichloromethane/ethyl acetate 1:1 (v/v)) = 0.61.

^{1}H NMR (500 MHz, CD_2Cl_2) δ [ppm] = 2.78 – 3.12 (m, 3H, H-k,l), 6.82 (d, J = 8.3 Hz, 1H, H-n), 7.18 – 7.23 (m, 2H, H-h,j), 7.26 (d, J = 2.1 Hz, 1H, H-m), 7.33 (dd, J = 8.4, 2.1 Hz, 1H, H-o), 7.36 (ddd, J = 7.5, 4.8, 1.2 Hz, 1H, H-b), 7.44 (dd, J = 7.9, 2.0 Hz, 1H, H-i), 7.87 (td, J = 7.7, 1.8 Hz, 1H, H-c), 8.00 (dd, J = 8.3, 2.4 Hz, 1H, H-f), 8.48 (dt, J = 8.0, 1.1 Hz, 1H, H-d), 8.51 (dd, J = 8.3, 0.9 Hz, 1H, H-e), 8.70 (ddd, J = 4.8, 1.9, 1.0 Hz, 1H, H-a), 8.87 (dd, J = 2.4, 0.9 Hz, 1H, H-g).

^{13}C NMR (126 MHz, CD_2Cl_2) δ [ppm] = 31.3 (C-k), 31.3 (C-l), 117.0 (C-h), 120.2 (C-9), 120.7 (C-n), 120.7 (C-e), 120.8 (C-d), 123.8 (C-b), 125.7 (C-i), 127.9 (C-6), 129.8 (C-o), 130.6 (C-7), 130.8 (C-j), 132.4 (C-m), 134.9 (C-f), 134.9 (C-3), 136.4 (C-4), 136.8 (C-c), 147.3 (C-g), 149.2 (C-a), 154.4 (C-8), 155.2 (C-2), 155.6 (C-1), 155.9 (C-5).

HRMS (ESI^+ Orbitrap): m/z (relative intensity) = 441.0706 (75%, $[M+H]^+$, calcd. 441.0709).

FTIR (ATR, thin film from CD_2Cl_2): $\tilde{\nu}$ [cm^{-1}] = 558.2 (vw), 628.6 (vw), 711.0 (w), 749.5 (m), 795.4 (s), 833.2 (m), 899.1 (vw), 924.4 (vw), 953.3 (w), 992.1 (vw), 1016.5 (vw), 1096.0 (w), 1243.7 (vw), 1361.7 (w), 1397.3 (vw), 1435.1 (m), 1457.9 (vs), 1563.4 (w), 1573.6 (w), 1588.5 (m), 2853.1 (vw), 2948.1 (vw), 3008.1 (vw), 3050.3 (vw).

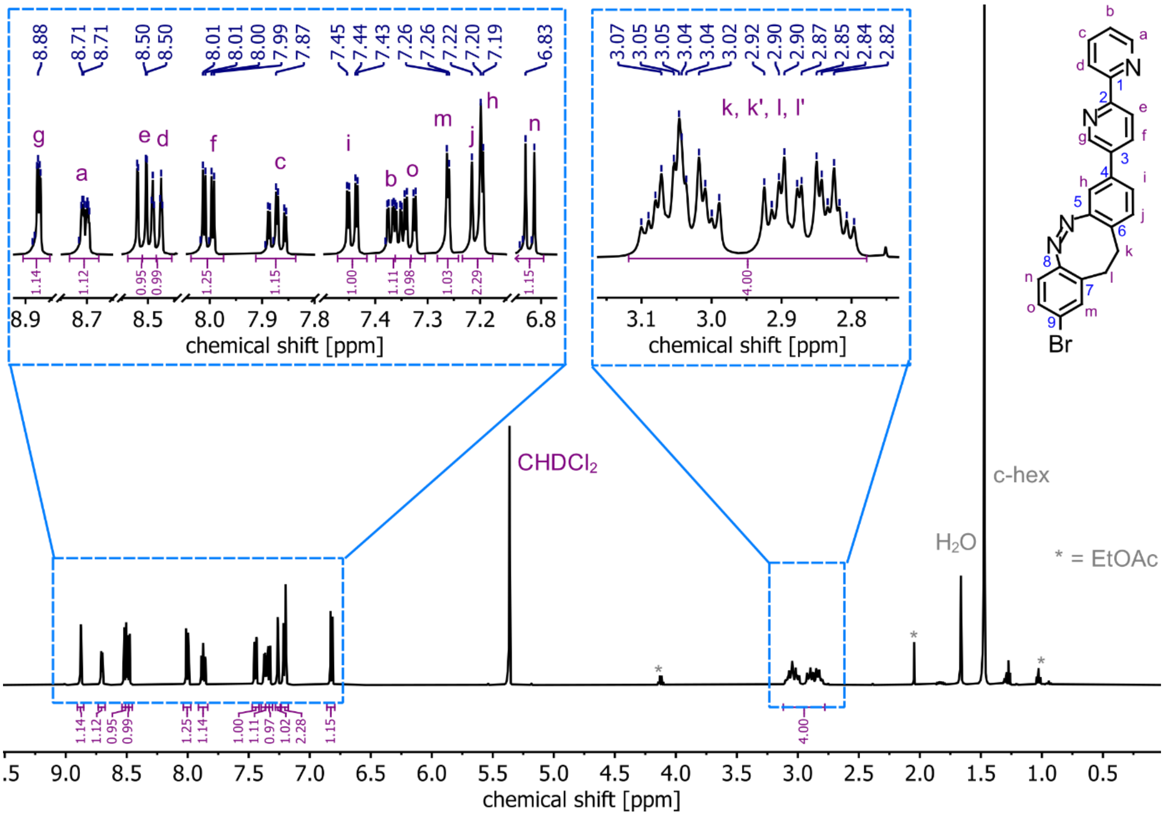

Figure S5. ^{1}H NMR spectrum (500 MHz, CD_2Cl_2, 298 K) of diazocine **S7**.

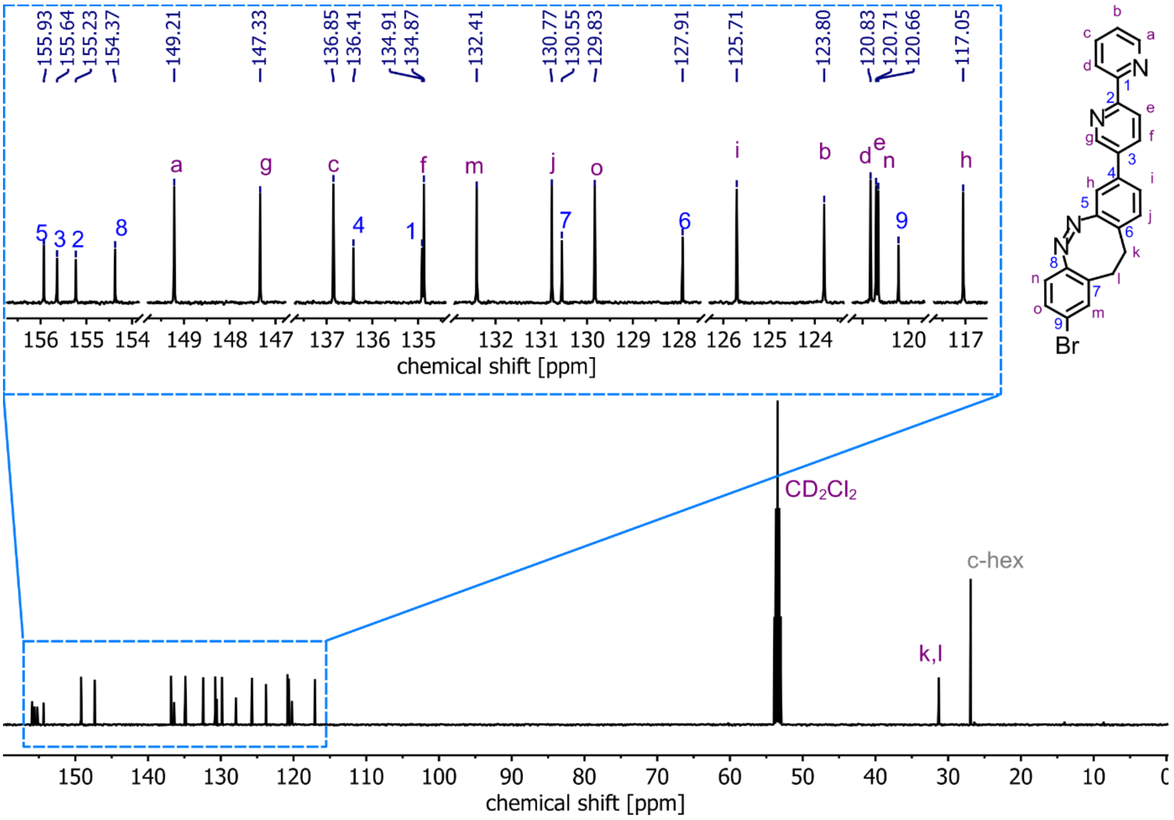

Figure S6. ^{13}C NMR spectrum (126 MHz, CD_2Cl_2, 298 K) of diazocine **S7**.

S2.5 (*Z*)-2-(6-(1,3-dioxolan-2-yl)pyridin-3-yl)-8-([2,2'-bipyridin]-5-yl)-11,12-dihydrodibenzo[*c*,*g*][1,2]diazocine (S8)

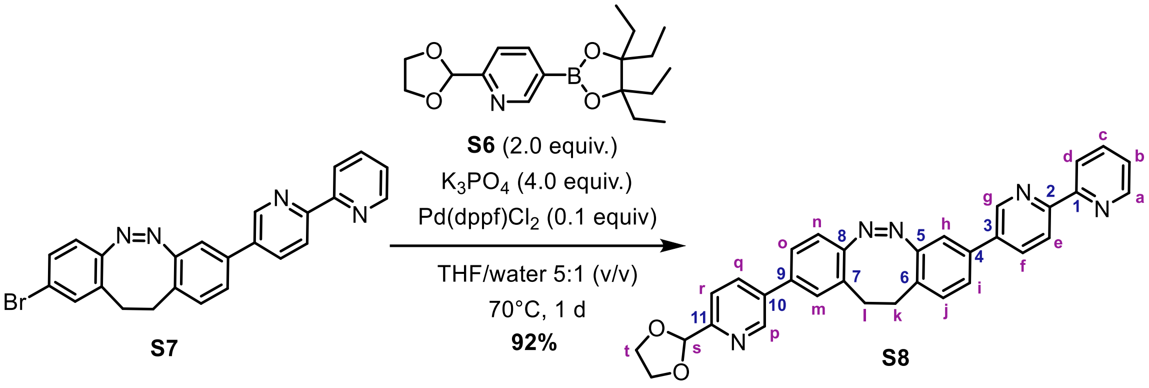

Diazocine **S7** (64.0 mg, 145 µmol, 1.0 equiv.), boronic ester **S6** (96.7 mg, 290 µmol, 2.0 equiv.), potassium phosphate (123 mg, 580 µmol, 4.0 equiv.), $Pd(dppf)Cl_2$ (11 mg, 15 µmol, 0.1 equiv.), and a magnetic stirring bar were added into a screw cap vial. The open vial was transferred into an argon-filled oxygen-free glovebox and a deoxygenated tetrahydrofuran/water mixture (5:1 (v/v), 3 mL) was added. The vial containing the reaction mixture was closed and removed from the glovebox. The reaction mixture was stirred at 70 °C for 1 day under the vial's remaining Argon atmosphere. After cooling to room temperature, water (10 mL) and ethyl acetate (20 mL) were added to the reaction mixture and the phases were separated. The organic phase was washed with water (1 × 15 mL) and saturated aqueous NaCl solution (1 × 15 mL) and dried over magnesium sulfate. After all volatiles were removed by rotary evaporation, the residue was subjected to column chromatography (silica gel, dichloromethane/*iso*-propanol 100:0 → 80:20 (v/v) over 15 column volumes) to afford product **S8** as a yellow solid in 92% yield (68 mg, 133 µmol).

R_F value (dichloromethane/ethyl acetate 1:1 (v/v)) = 0.19.

^{1}H NMR (500 MHz, CD_2Cl_2) δ [ppm] = 2.83 – 3.11 (m, 4H, H-k,l), 4.00 – 4.19 (m, 4H, H-t), 5.80 (s, 1H, H-s), 7.02 (d, *J* = 8.1 Hz, 1H, H-q), 7.19 (d, *J* = 8.0 Hz, 1H, H-j), 7.21 (d, *J* = 1.9 Hz, 1H, H-h), 7.29 – 7.34 (m, 2H, H-b,m), 7.40 (dd, *J* = 7.9, 2.0 Hz, 1H, H-i), 7.43 (dd, *J* = 8.2, 1.9 Hz, 1H, H-o), 7.55 (dd, *J* = 8.1, 0.9 Hz, 1H, H-r), 7.83 (td, *J* = 7.7, 1.8 Hz, 1H, H-c), 7.86 (dd, *J* = 8.2, 2.3 Hz, 1H, H-q), 7.97 (dd, *J* = 8.3, 2.4 Hz, 1H, H-f), 8.43 (dt, *J* = 8.0 Hz, 1.1, 1H, H-d), 8.46 (dd, *J* = 8.3, 0.9 Hz, 1H, H-e), 8.66 (ddd, *J* = 4.8, 1.8, 1.0 Hz, 1H, H-a), 8.73 (dd, *J* = 2.4, 0.9 Hz, 1H, H-p), 8.84 (dd, *J* = 2.4, 0.9 Hz, 1H, H-g).

^{13}C NMR (126 MHz, CD_2Cl_2) δ [ppm] = 31.4 (C-k), 31.7 (C-l), 65.6 (C-t), 103.7 (C-s), 117.2 (C-h), 119.8 (C-n), 120.4 (C-r), 120.7 (C-e), 120.8 (C-d), 123.7 (C-b), 125.6 (C-o), 125.6 (C-i), 128.1 (C-6), 128.4 (C-m), 128.9 (C-), 130.8 (C-j), 134.8 (C-q), 134.8 (C-f), 134.9 (C-3), 135.6 (C-10), 136.2 (C-4), 136.3 (C-9), 136.8 (C-c), 147.3 (C-g,p), 149.1 (C-a), 155.1 (C-2), 155.3 (C-8), 155.6 (C-1), 156.0 (C-5), 156.3 (C-11).

HRMS (ESI$^+$ Orbitrap): m/z (relative intensity) = 512.2080 (100%, $[M+H]^+$, calcd. 512.2080), 534.1895 (30%, $[M+Na]^+$, calcd. 534.1900).

FTIR (ATR, thin film from CD_2Cl_2): $\tilde{\nu}$ [cm^{-1}] = 668.2 (m), 750.2 (m), 796.4 (s), 841.4 (m), 943.5 (w), 1021.5 (m), 1092.9 (vs), 1242.9 (w), 1363.1 (w), 1387.6 (w), 1457.9 (vs), 1521.0 (vw), 1588.9 (m), 2851.4 (w), 2893.1 (w), 2922.0 (m), 3049.6 (w).

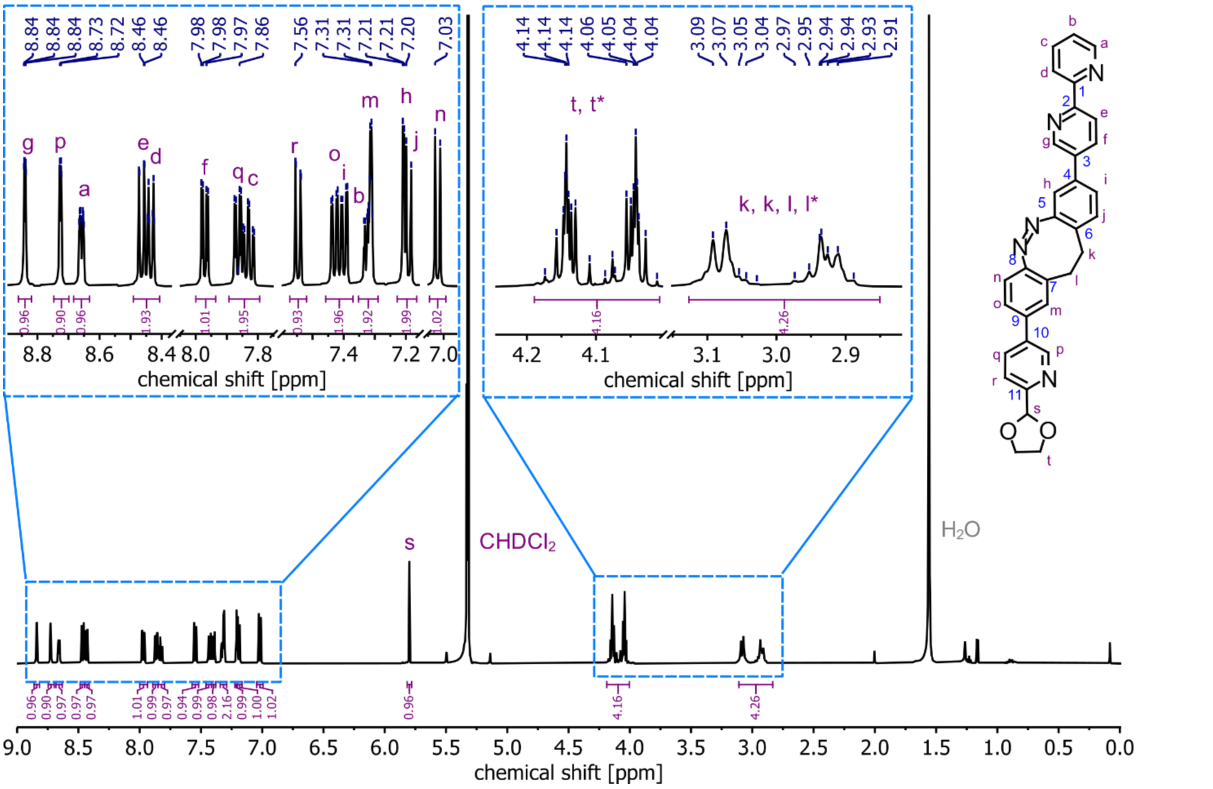

Figure S7. ^{1}H NMR spectrum (500 MHz, CD_2Cl_2, 298 K) of diazocine **S8**.

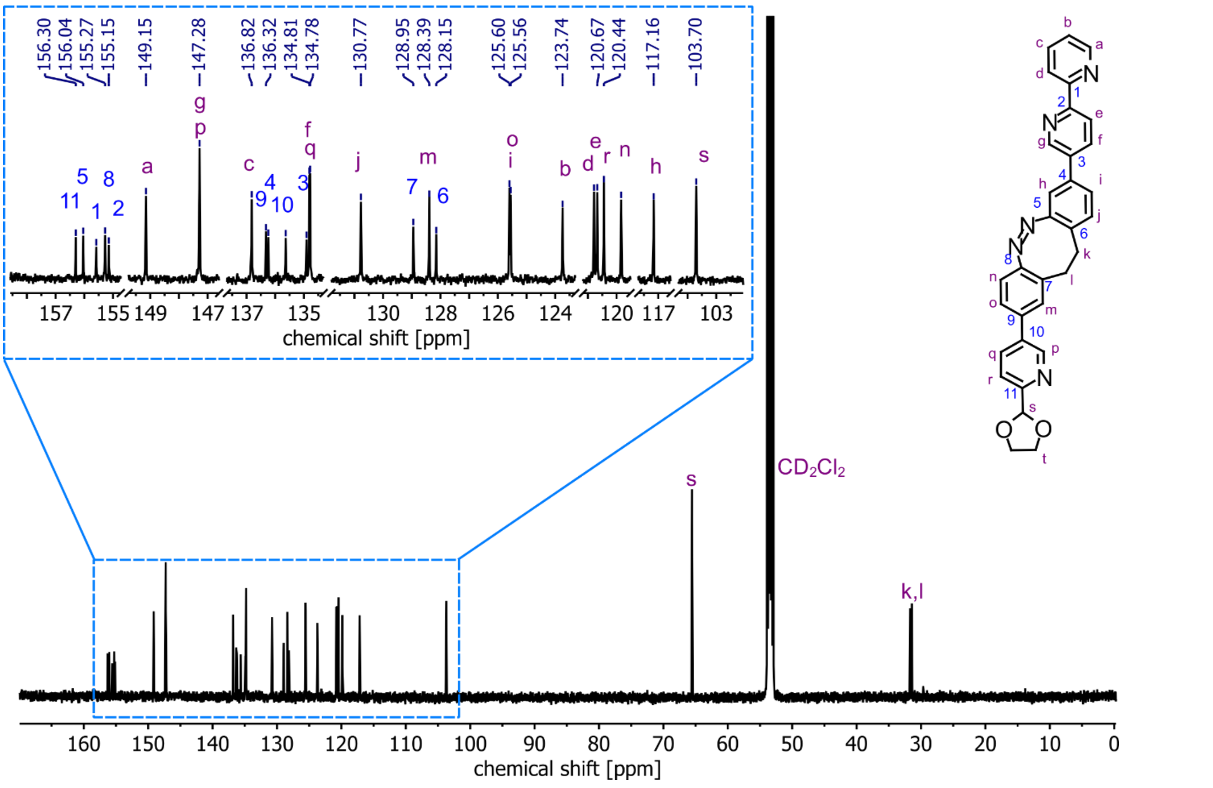

Figure S8. ^{13}C NMR spectrum (126 MHz, CD_2Cl_2, 298 K) of diazocine **S8**.

S2.6 (*Z*)-5-(8-([2,2'-bipyridin]-5-yl)-11,12-dihydrodibenzo[*c*,*g*][1,2]diazocin-2-yl)picolinaldehyde (**1**)

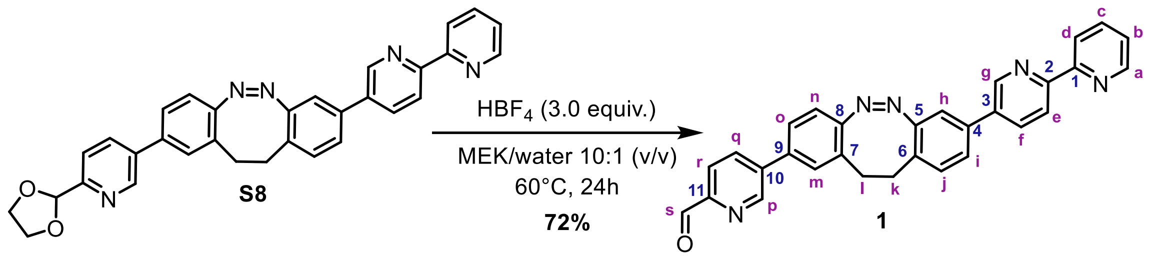

Diazocine **S8** (37.5 mg, 73 µmol, 1.0 equiv.) was dissolved in 10 mL of a mixture of methyl ethyl ketone and water (10:1, v/v) and HBF_4 (32.5 µL, 50 wt% in water, 19.3 mg, 220 µmol, 3.0 equiv.) was added. The reaction vessel was purged with argon for 5 minutes and the reaction mixture stirred at 60°C for 24 hours. After cooling to room temperature, water (10 mL) and ethyl acetate (20 mL) were added to the reaction mixture, and the phases were separated. The organic phase was washed with water (1 × 15 mL) and saturated aqueous NaCl solution (1 × 15 mL) and dried with magnesium sulfate. All volatiles were removed by rotary evaporation, and the residue was subjected to column chromatography (silica gel, dichloromethane/*iso*-propanol 100:0 → 80:20 (v/v) over 15 column volumes) to afford product **1** as a yellow solid in 72% yield (24.6 mg, 53 µmol).

R_F value (dichloromethane/ethyl acetate 1:1 (v/v)) = 0.54.

^{1}H NMR (500 MHz, CD_2Cl_2) δ [ppm] = 2.87 – 3.15 (m, 4H, H-k,l), 7.06 (d, J = 8.2 Hz, 1H, H-n), 7.19 (d, J = 8.0 Hz, 1H, H-j), 7.21 (d, J = 1.9 Hz, 1H, H-h), 7.32 (ddd, J = 7.5, 4.8, 1.2 Hz, 1H, H-b), 7.38 (d, J = 2.0 Hz, 1H, H-m), 7.40 (dd, J = 7.9, 2.0 Hz, 1H, H-i), 7.50 (dd, J = 8.2, 2.0 Hz, 1H, H-o), 7.83 (ddd, J = 8.1, 7.5, 1.8 Hz, 1H, H-c), 7.94 – 7.98 (m, 2H, H-f,q), 8.00 (ddd, J = 8.1, 2.2, 0.8 Hz, 1H, H-r), 8.43 (dt, J = 8.0, 1.1 Hz, 1H, H-d), 8.47 (dd, J = 8.3, 0.8 Hz, 1H, H-e), 8.66 (ddd, J = 4.8, 1.8, 0.9 Hz, 1H, H-a), 8.84 (dd, J = 2.4, 0.9 Hz, 1H, H-g), 8.94 (dd, J = 2.2, 0.9 Hz, 1H, H-p), 10.05 (d, J = 0.7 Hz, 1H, H-s).

^{13}C NMR (126 MHz, CD_2Cl_2) δ [ppm] = 31.4 (C-k), 31.7 (C-l), 117.1 (C-h), 120.0 (C-n), 120.7 (C-e), 120.8 (C-d), 121.5 (C-r), 123.8 (C-b), 125.6 (C-i), 125.9 (C-o), 128.0 (C-6), 128.7 (C-m), 129.3 (C-9), 130.8 (C-j), 134.8 (C-f), 134.8 (C-3), 134.9 (C-q), 135.4

(C-7), 136.3 (C-4), 136.8 (C-c), 139.3 (C-10), 147.3 (C-g), 148.4 (C-p), 149.2 (C-a), 151.7 (C-11), 155.2 (C-2), 155.6 (C-1), 155.9 (C-8), 156.0 (C-5), 192.9 (C-s).

HRMS (ESI⁺ Orbitrap): m/z (relative intensity) = 468.1818 (100%, $[M+H]^+$, calcd. 468.1819).

FTIR (ATR, thin film from CD_2Cl_2): $\tilde{\nu}$ [cm^{-1}] = 750.5 (w), 796.5 (m), 1017.9 (w), 1095.3 (vw), 1211.2 (w), 1363.1 (w), 1458.1 (vs), 1585.9 (m), 1708.8 (vs) (C=O), 2851.0 (w), 2922.9 (m), 3051.5 (vw).

Elemental analysis ($C_{30}H_{21}N_5O_1$):

Calcd. (%)	C: 77.07	H: 4.53	N: 14.98
Found (%)	C. 77.39	H: 4.70	N: 14.77

HPLC chromatography (reverse phase (C18), water / acetonitrile 65:35 (v/v) + 1% Et_2NH):

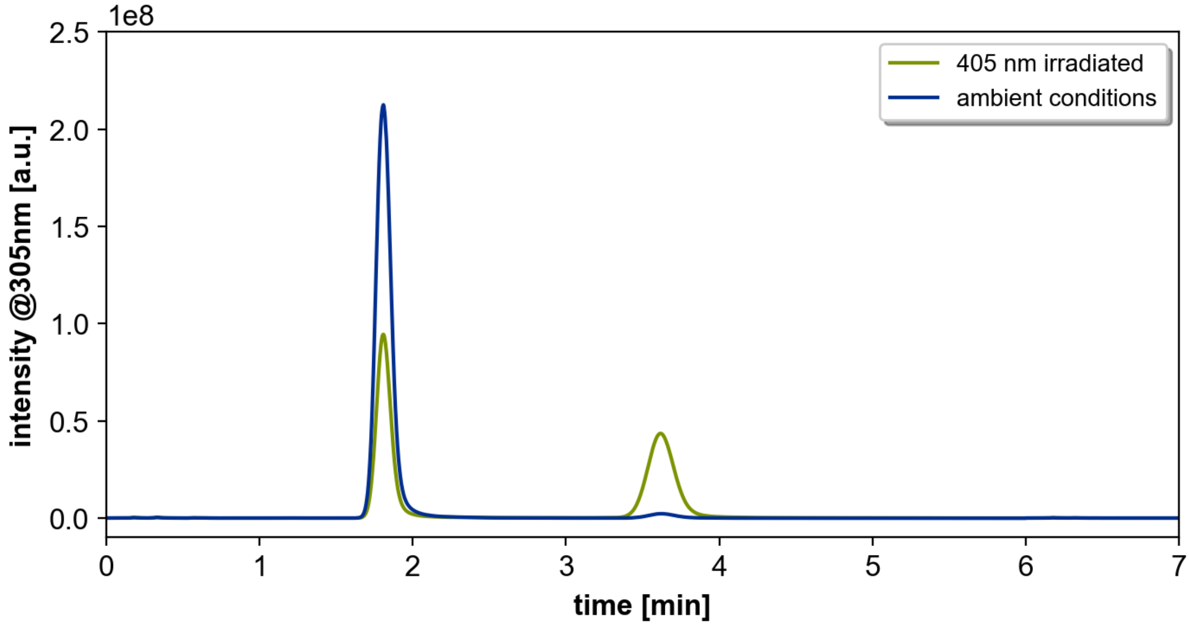

Figure S9. HPLC chromatogram showing the absorbance at 305 nm of aldehyde **1** before and after irradiation with 405 nm light. The sample shows a major peak corresponding to *Z*-**1** (retention time approx. 1.8 min) and a minor peak corresponding to the *E*-**1** (retention time approx. 3.6 min). The increased intensity of the second peak after irradiating the sample with 405 nm light confirms peak assignment.

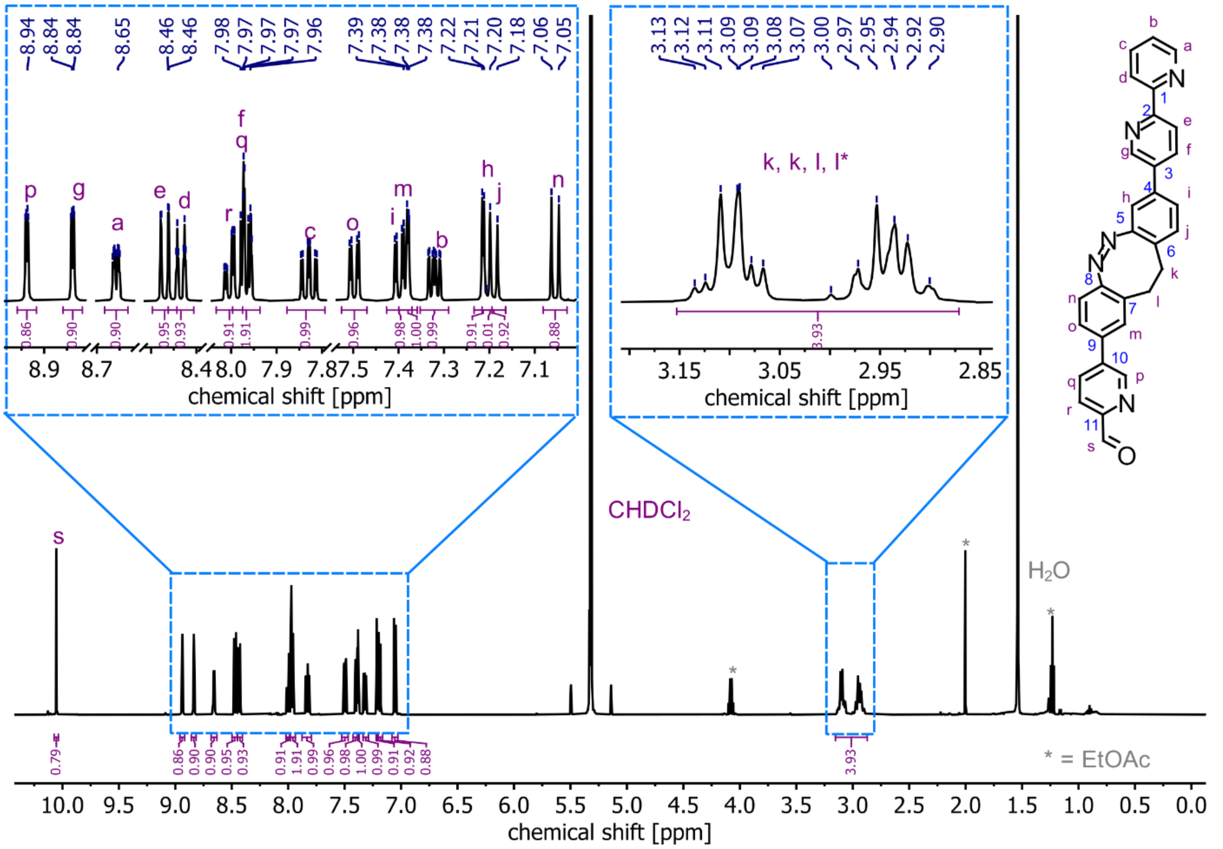

Figure S10. ^{1}H NMR spectrum (500 MHz, CD_2Cl_2, 298 K) of diazocine **1**.

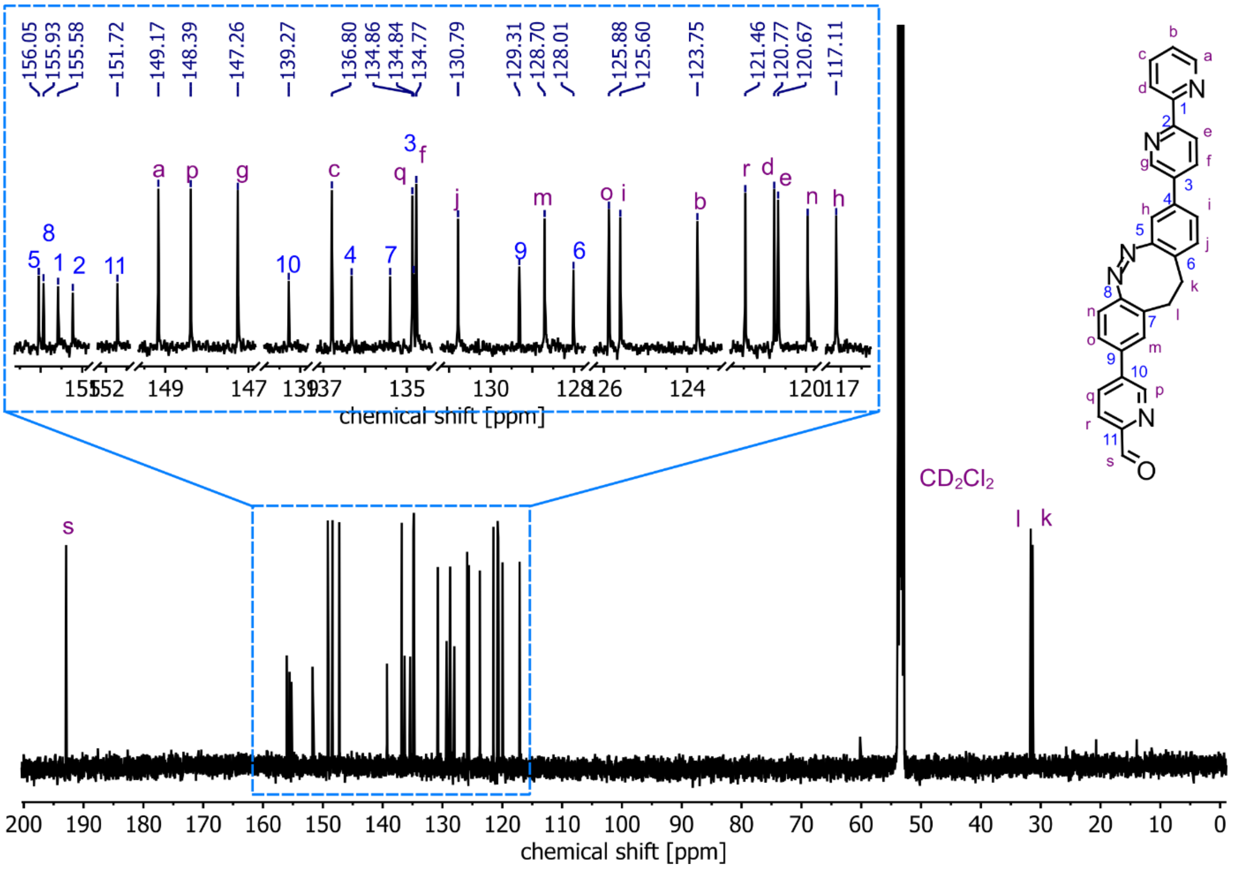

Figure S11. ^{13}C NMR spectrum (126 MHz, CD_2Cl_2, 298 K) of diazocine **1**.

S3 Synthesis of the bimetallic helicates

S3.1 Use of stock solutions

The use of stock solutions was essential for the synthesis of the bimetallic helicates presented herein. Some key aspects include:

1) The stoichiometry must be accurate for successful helicate formation and especially for the self-sorting experiments. The weight disparity between the ligand and the amine of 10:1 made this impossible at small scale.
2) The compounds must be fully dissolved in or miscible with the chosen solvent.
3) Solvents other than acetonitrile should be kept at a minimum, as the presence of excess protic solvents such as water or methanol hinder the formation of the complexes.
4) All fluid transfers were carried out using *Gilson* pipettes.
5) Stock solutions remained usable for months when stored in closed screw cap vials in a fridge at 6 °C.

S3.2 General procedure

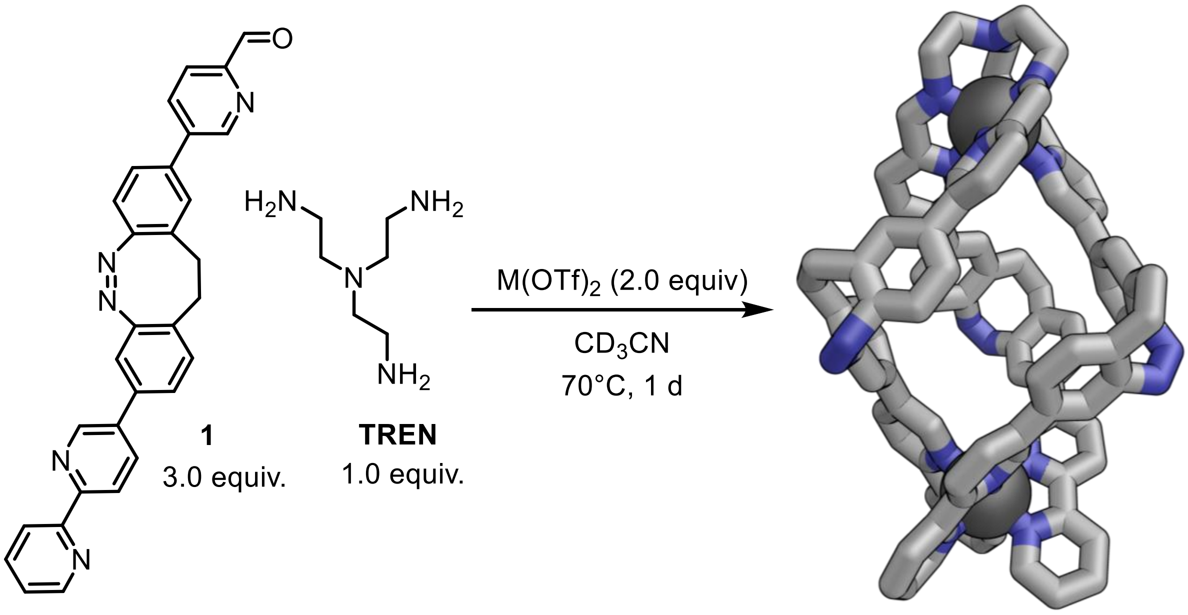

Aldehyde **1** (approx. 1–5 mg, 3.0 equiv.) was weighed into a screw-cap vial as a solid and a highly concentrated stock solution of tris(2-aminoethyl)amine (1.0 equiv.) in deuterated methanol (>50 mg mL^{-1}) was added to the bottom of the vial. Subsequently, a stock solution of metal salt in deuterated acetonitrile (2.0–2.2 equiv., 2–40 mg/mL, depending on the solubility of metal salt) was added. If the vial was not

filled with at least 0.5 mL of solvent, more deuterated acetonitrile was added. Finally, a magnetic stir bar was added, and the vial was closed and heated (to between 45 °C and 65 °C as specified) overnight. A ^{1}H NMR spectrum was measured to confirm successful complex formation. The complexes reported herein could be synthesized in a highly reproducible manner, if reaction time (>12 h) and temperature (>45° C) were sufficiently long and high, respectively, and the targeted stoichiometry was hit accurately.

If the spectrum was not sufficiently clean and impurities were present, the complex was purified by precipitation from diethyl ether. For this, the crude reaction mixture was filtered through a pipette stuffed with an extra fine glass fibre filter (pore size 1.6 µm). The filtrate was added in portions not larger than 0.2 mL (for triflates, perchlorates, and tetrafluoroborates) or no larger than 0.1 mL (for triflimides) to a 2 mL *Eppendorf* centrifuge vial filled with 1.7 mL of diethyl ether. The vial was shaken vigorously and centrifuged (14000 rpm, 1 minute). The supernatant was carefully discarded into a clean beaker to catch any solid in case the precipitate plug fell out. Another portion of 1.7 mL of diethyl ether was added to the residue and a second portion of the filtrate was added. This process was repeated until the filtrate was used up completely. If this was the case, the residue was suspended in 0.1 mL of acetonitrile and 1.9 mL of diethyl ether were added. The suspension was centrifuged (14000 rpm, 1 minute), the supernatant carefully decanted, and the residue suspended in 2.0 mL of diethyl ether. The centrifuge vial was sonicated for 1 minute and centrifuged (14000 rpm, 1 minute). This was repeated twice. The final residue was left open to air to dry for a few hours. Decomposition on air was not observed, but drying under reduced pressure resulted in decomposition. Drying on air resulted in mostly dry samples, but the last traces of solvent and/or water proved difficult to remove for the helicates investigated herein.

Complex formation is typically quantitative, lower yields can be attributed to losses during extraction/precipitation cycles.

S3.3 Zn_2**L**

Zn_2**L** was synthesized according to the general procedure (Section S3.2) using $Zn(OTf)_2$ (1.19 mg, 3.28 µmol, 2.00 equiv.), aldehyde **1** (2.30 mg, 4.92 µmol, 3.00 equiv.), and TREN (0.24 mg, 1.64 µmol, 1.00 equiv.) with a reaction time of 1 day at a temperature of 70 °C. After precipitation the product was obtained as a yellow powder in 83% yield (3.27 mg, 1.39 µmol).

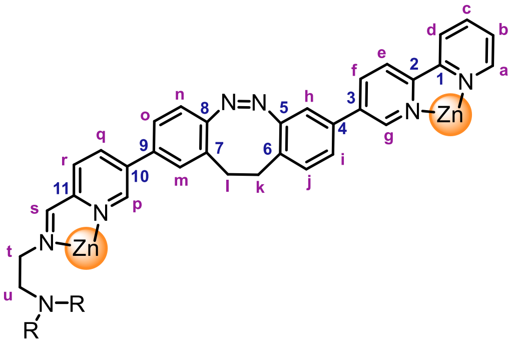

$[C_{96}H_{75}N_{19}Zn_2](CF_3O_3S)_4$

^{1}H NMR (700 MHz, CD_3CN) δ [ppm] = 2.74 (dt, J = 15.1, 7.6 Hz, 3H, H-l), 2.86 (q, J = 7.2 Hz, 6H, H-k), 2.91 – 3.00 (m, 6H, H-l′,u), 3.26 (dd, J = 13.7, 3.9 Hz, 3H, H-u′), 3.68 (dd, J = 11.9, 3.6 Hz, 3H, H-t), 3.86 (t, J = 12.8 Hz, 3H, H-t′), 6.42 (d, J = 1.9 Hz, 3H, H-h), 6.50 (d, J = 2.2 Hz, 3H, H-p), 6.71 (d, J = 1.9 Hz, 3H, H-m), 6.90 (d, J = 8.1 Hz, 3H, H-n), 7.03 (dd, J = 8.2, 1.9 Hz, 3H, H-o), 7.49 – 7.54 (m, 6H, H-g,j), 7.60 – 7.64 (m, 6H, H-b,i), 8.02 (ddd, J = 5.2, 1.7, 0.8 Hz, 3H, H-a), 8.20 (d, J = 8.0 Hz, 3H, H-r), 8.29 (td, J = 7.9, 1.7 Hz, 3H, H-c), 8.39 (dd, J = 8.0, 2.2 Hz, 3H, H-q), 8.56 (dd, J = 8.5, 2.3 Hz, 3H, H-f), 8.65 (dt, J = 8.3, 1.1 Hz, 3H, H-d), 8.69 (d, J = 8.6 Hz, 3H, H-e), 8.79 (d, J = 1.9 Hz, 3H, H-s).

^{13}C NMR (176 MHz, CD_3CN) δ [ppm] = 31.6 (C-l), 32.6 (C-k), 54.9 (C-u), 56.6 (C-t), 119.0 (C-h), 121.9 (C-n), 124.4 (C-e), 125.1 (C-d), 126.0 (C-o), 126.8 (C-b), 128.6 (C-i), 129.4 (C-r), 129.7 (C-m), 130.1 (C-7), 130.2 (C-6), 132.0 (C-j), 134.9 (C-4,9), 138.8 (C-3), 139.3 (C-q), 140.5 (C-f), 140.9 (C-10), 142.9 (C-c), 146.3 (C-p), 146.4 (C-g), 147.2 (C-11), 149.2 (C-2,a), 149.6 (C-1), 156.1 (C-8), 156.3 (C-5), 163.4 (C-s).

HRMS (ESI$^+$ Orbitrap): m/z (relative intensity) = 406.3756 (77%, $[Zn_2\mathbf{L}]^{4+}$, calcd. 406.3751), 591.4843 (100%, $[Zn_2\mathbf{L} + OTf]^{3+}$, calcd. 591.4843), 961.7037 (45%, $[Zn_2\mathbf{L} + 2OTf]^{2+}$, calcd. 961.7027).

FTIR (ATR, thin film from CD_3CN): $\tilde{\nu}$ [cm^{-1}] = 417.3 (vw), 517.6 (w), 574.0 (w), 638.0 (vs) (δ_s *F₃C**SO₃***), 756.1 (w), 797.5 (w), 843.8 (w), 901.5 (vw), 931.6 (vw), 1029.6 (vs) (ν_s *F₃C**SO₃***), 1161.0 (s) (ν_{as} **F₃C**SO₃), 1256.8 (vs) (ν_{as} F₃C**SO₃**), 1369.4 (vw), 1441.0 (w),

1473.1 (w), 1569.7 (w), 1599.2 (w), 1655.8 (w), 2857.1 (vw), 2926.8 (vw), 2947.5 (vw), 3069.3 (vw).

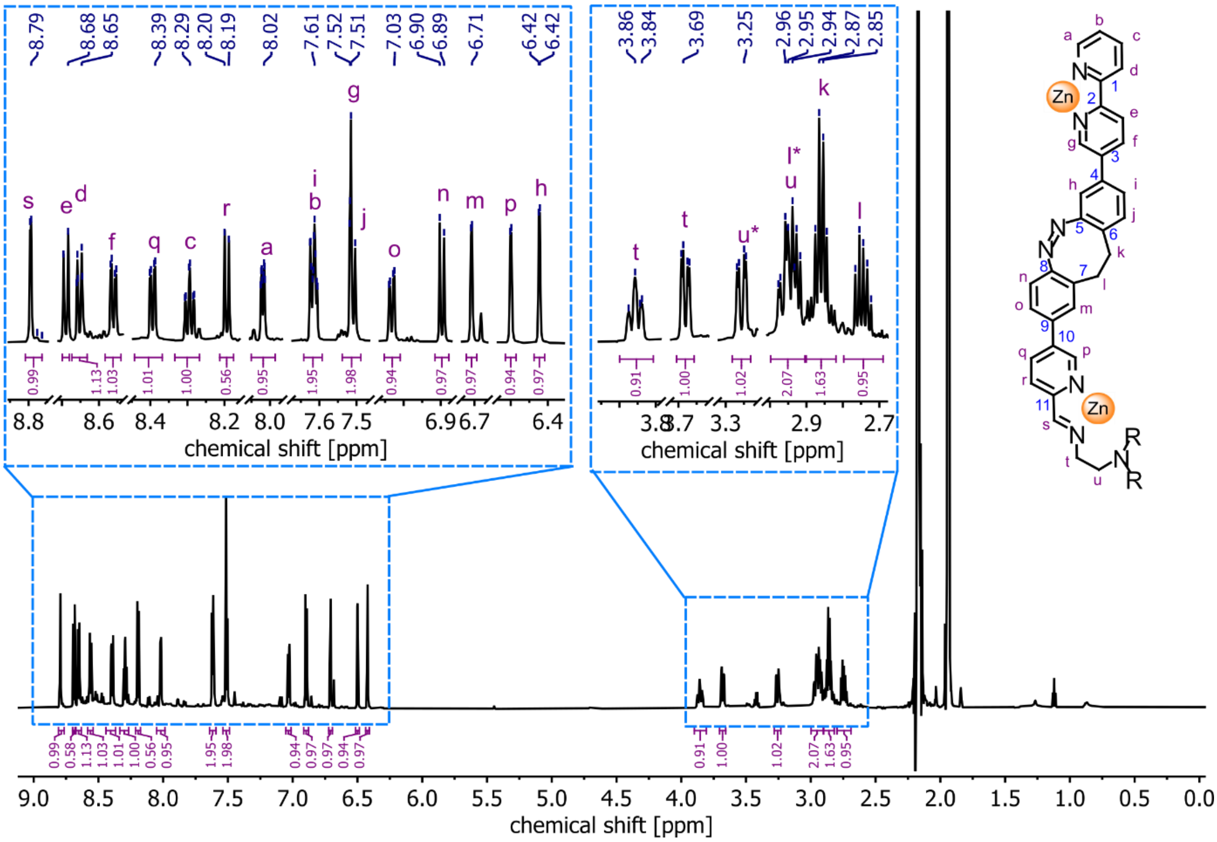

Figure S12. ¹H NMR spectrum (700 MHz, CD_3CN, 298 K) of Zn_2**L**.

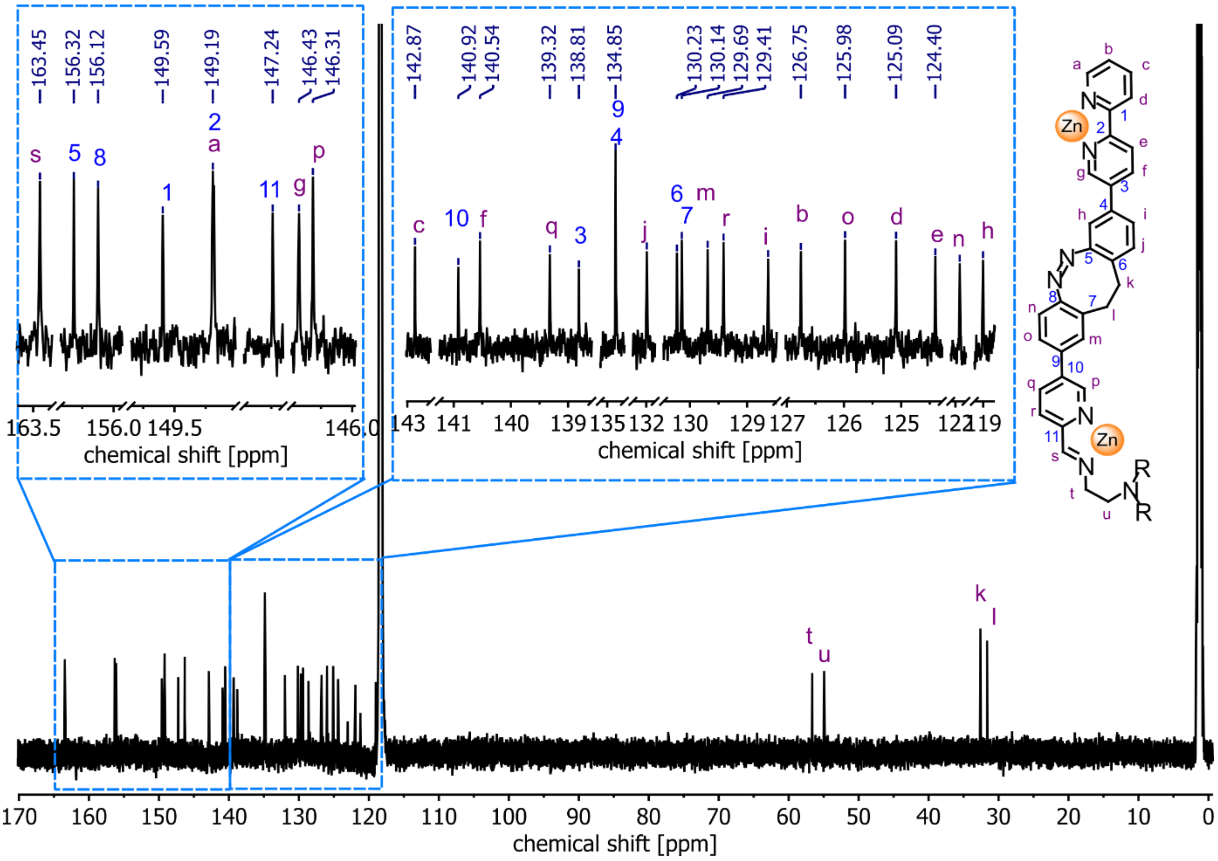

Figure S13. ¹³C NMR spectrum (176 MHz, CD_3CN, 298 K) of Zn_2**L**.

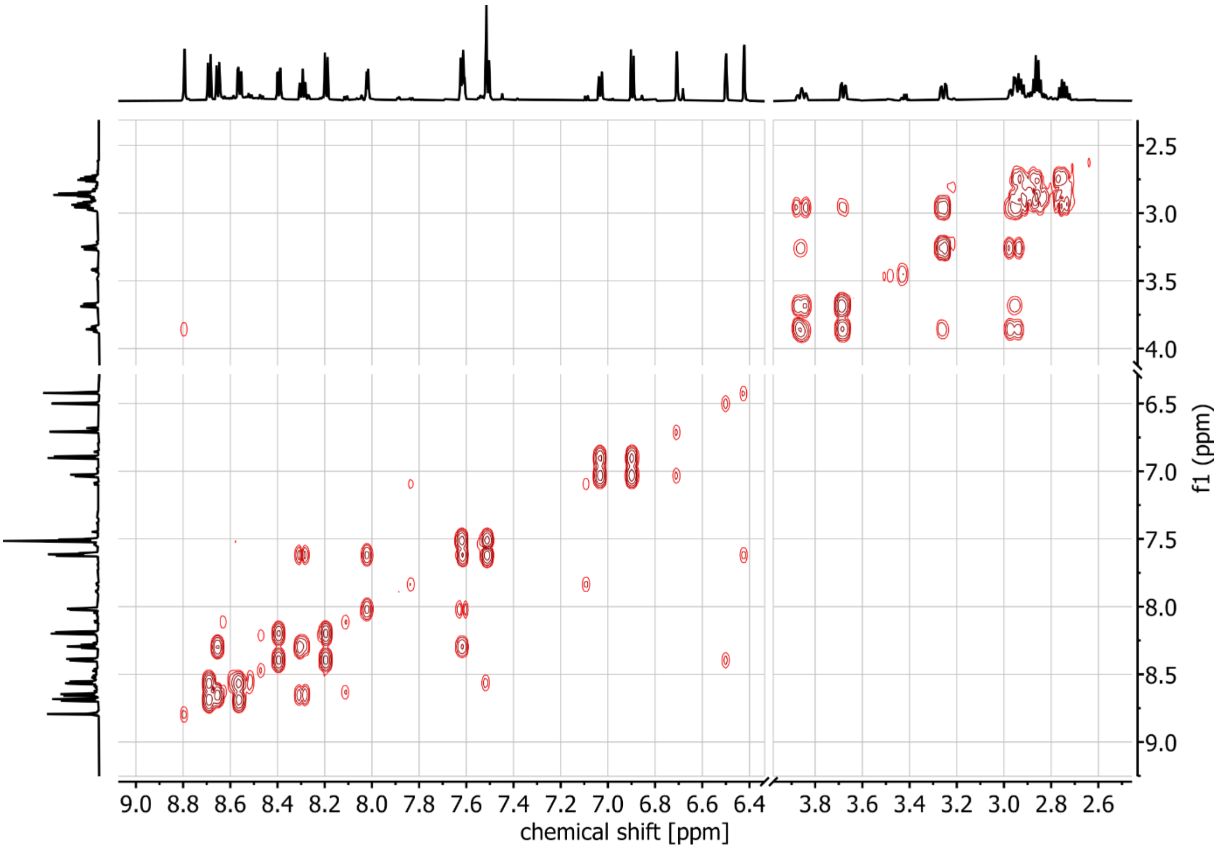

Figure S14. ^{1}H,^{1}H COSY spectrum (700 MHz, CD_3CN, 298 K) of Zn_2**L**.

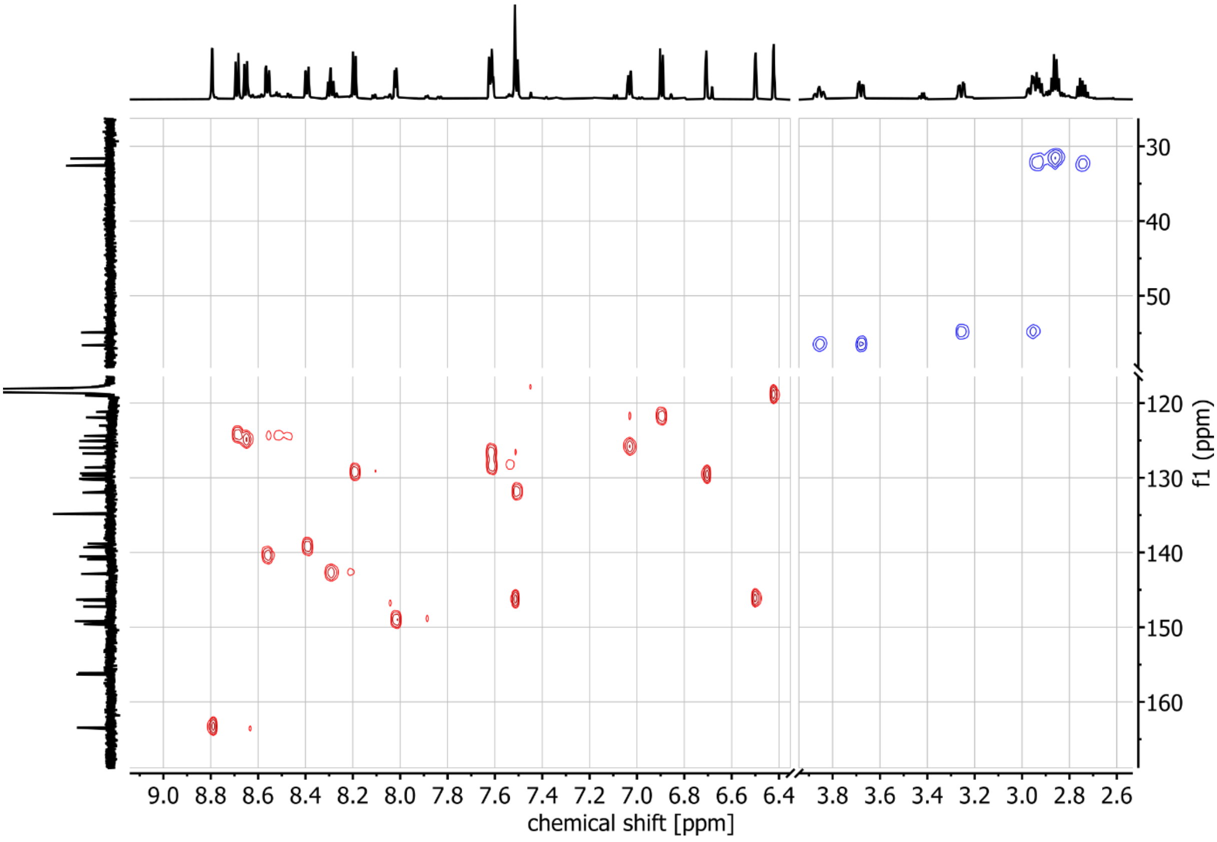

Figure S15. ^{1}H,^{13}C HSQC spectrum (700/176 MHz, CD_3CN, 298 K) of Zn_2**L**.

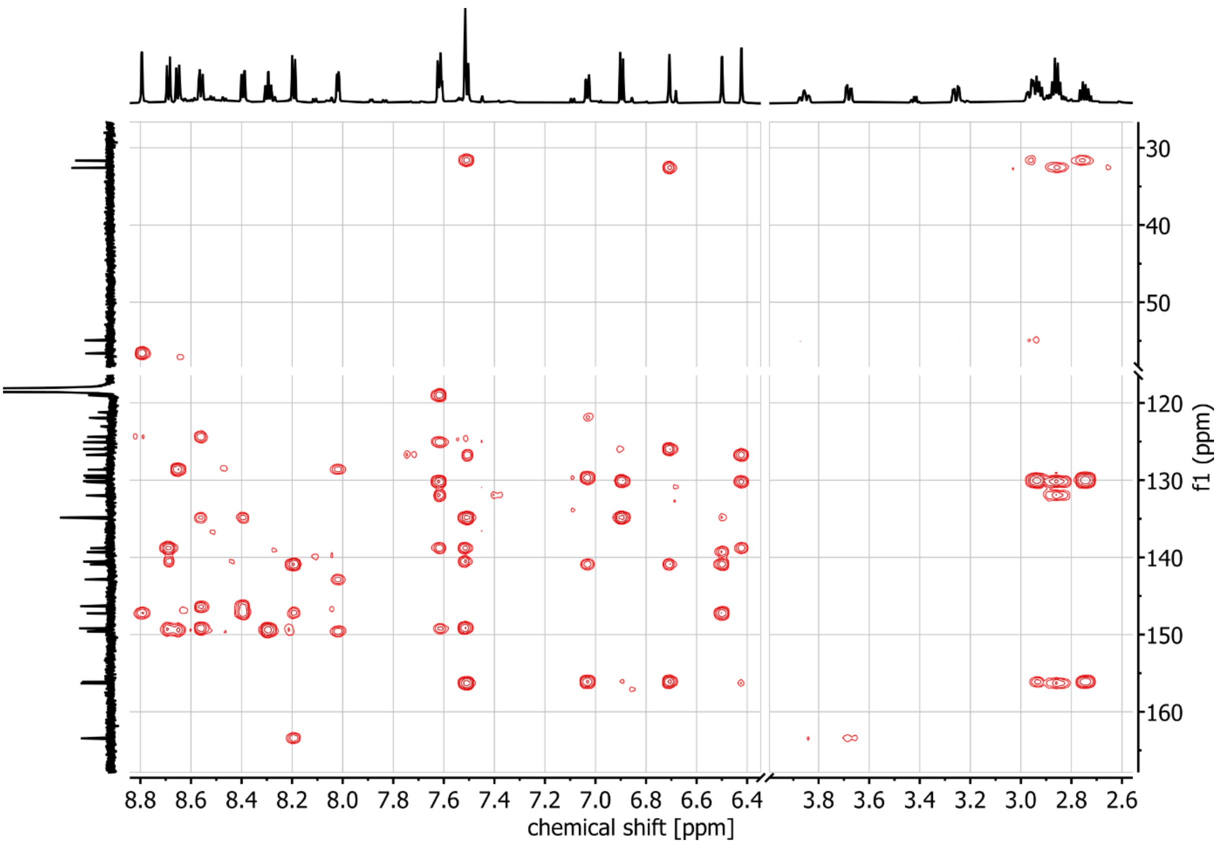

Figure S16. $^{1}H,^{13}C$ HMBC spectrum (700/176 MHz, CD_3CN, 298 K) of Zn_2**L**.

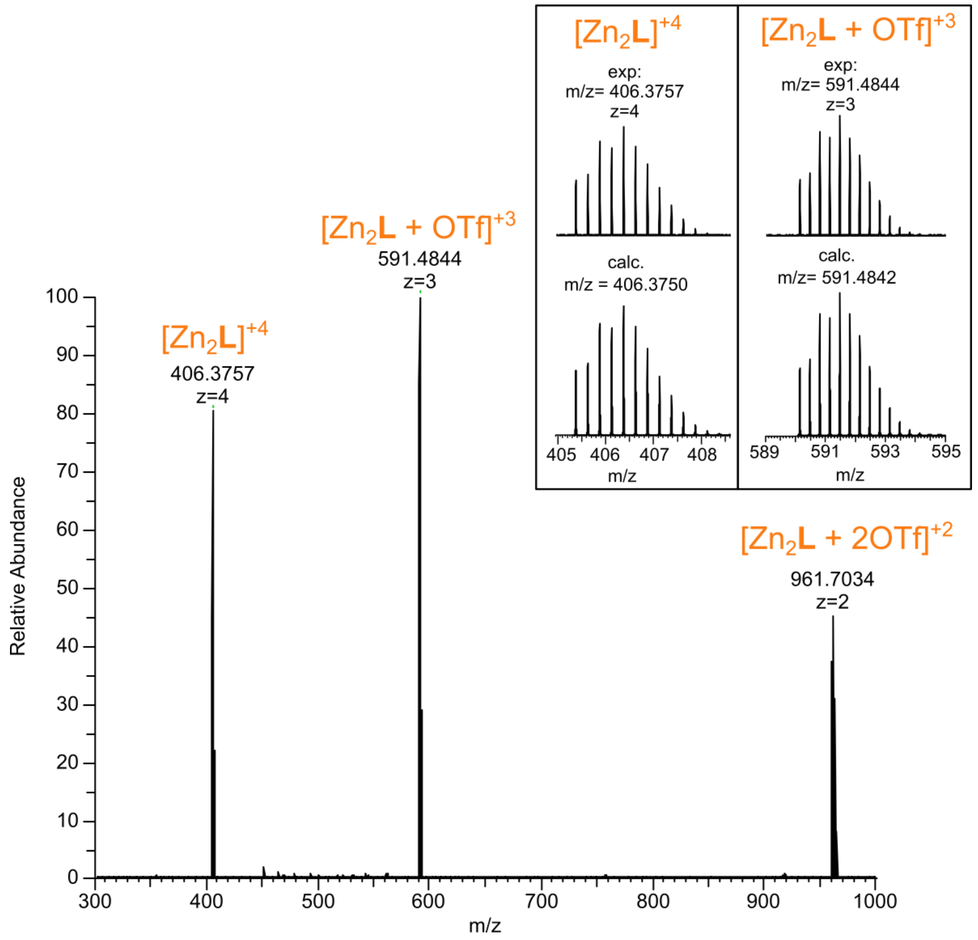

Figure S17. ESI^{+} mass spectrum (CH_3CN) of Zn_2**L**.

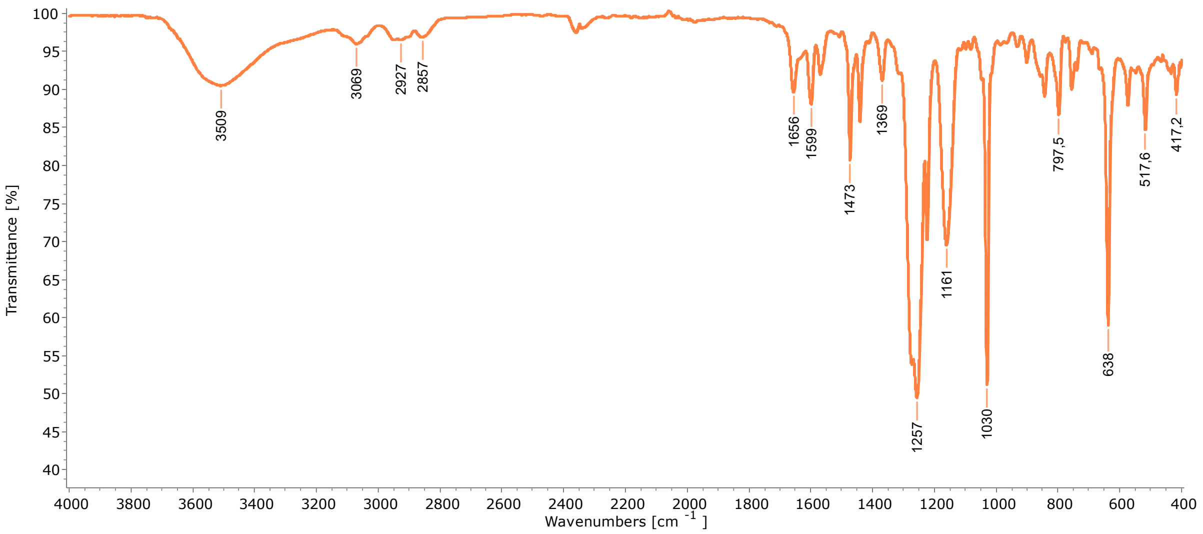

Figure S18. FTIR spectrum (ATR, thin film from evaporation of a CD_3CN solution) of Zn_2**L**.

S3.4 Fe_2**L**

Fe_2**L** was synthesized according to the general procedure (Section S3.2) using $Fe(OTf)_2$ (0.63 mg, 1.78 µmol, 2.00 equiv.), aldehyde **1** (1.25 mg, 2.67 µmol, 3.00 equiv.), and TREN (0.13 mg, 0.89 µmol, 1.00 equiv.) with a reaction time of 1 day at a temperature of 70 °C. After precipitation the product was obtained as a black powder in 90% yield (1.92 mg, 0.81 µmol).

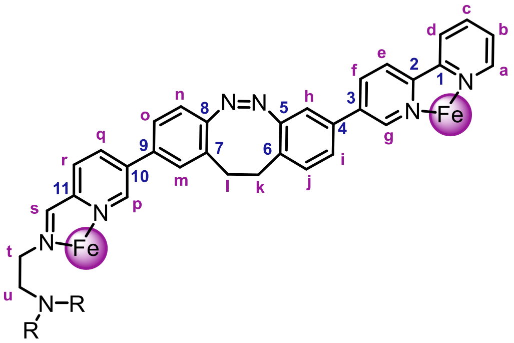

$[C_{96}H_{75}N_{19}Fe_2](CF_3O_3S)_4$

^{1}H NMR (700 MHz, CD_3CN) δ [ppm] = 2.67 – 2.77 (m, 3H, H-l), 2.77 – 2.89 (m, 6H, H-k), 2.96 (ddd, J = 14.8, 8.7, 5.6 Hz, 3H, H- l'), 3.22 (t, J = 13.8 Hz, 3H, H-u), 3.33 (t, J = 12.0 Hz, 3H, H-t), 3.90 (s(br), 3H, H-u'†), 4.03 (s(br), 3H, H-t'*), 6.30 (d, J = 1.9 Hz, 3H, H-h), 6.45 (s(br), 3H, H-p*), 6.60 (d, J = 1.9 Hz, 3H, H-m), 6.97 (d, J = 2.1 Hz, 3H, H-g), 7.00 (d, J = 8.1 Hz, 3H, H-n), 7.11 (dd, J = 8.1, 1.9 Hz, 3H, H-o), 7.41 (dt, J = 5.7, 1.4 Hz, 3H, H-a), 7.44 – 7.46 (m, 3H, H-b), 7.47 (d, J = 7.8 Hz, 3H, H-j), 7.53 (dd, J = 8.0, 2.0 Hz, 3H, H-i), 8.17 (td, J = 7.8, 1.5 Hz, 3H, H-c), 8.36 (dd, J = 8.2, 1.6 Hz, 3H, H-q), 8.44 (dd, J = 8.6, 2.1 Hz, 3H, H-f), 8.51 (d, J = 8.1 Hz, 3H, H-r), 8.65 (dt, J = 8.1, 1.1 Hz, 3H, H-d), 8.70 (d, J = 8.5 Hz, 3H, H-e), 9.59 (s(br), 3H, H-s*).

^{13}C NMR (176 MHz, CD_3CN) δ [ppm] = 31.3 (C-k), 32.6 (C-l), *53.9‡ (C-u), 60.5† (C-t), 117.8§ (C-h),* 121.6 (C-n), 125.0 (C-e), 125.9 (C-d), 126.4 (C-o), 126.7 (C-i), 128.7 (C-b), 129.8 (C-m,r), 130.5 (C-7), 131.0 (C-6), 132.0 (C-j), 134.3 (C-4), 135.2 (C-9), 136.9 (C-q), 137.7 (C-f), 138.9 (C-3), 139.9 (C-c), *141.6† (C-10),* 151.6 (C-g), 152.5 (C-11), 155.2 (C-a), 156.3 (C-8), 156.5 (C-5), 159.3 (C-1), 159.4 (C-2).

No signals or cross peaks could be observed for C-s and C-p as the corresponding ^{1}H NMR signals are already strongly broadened.

† The signals corresponding to H-p, H-s, H-u' and H-t' are strongly broadened.
‡ The signals corresponding to C-10, C-u, and C-t are not visible in the ^{13}C NMR spectrum and could only be found by their cross-peaks in ^{1}H,^{13}C HSQC and HMBC NMR.
§ The signal for C-h is underneath the solvent peak and could only be found by its cross-peak in ^{1}H,^{13}C HSQC NMR.

HRMS (ESI$^+$ Orbitrap): *m/z* (relative intensity) = 401.6297 (100%, [Fe_2**L**]$^{+4}$, calcd. 401.6290).

MS (ESI$^+$ Orbitrap): *m/z* (relative intensity) = 401.631 (100%, [Fe_2**L**]$^{+4}$, calcd. 401.629), 585.160 (34%, [Fe_2**L** + OTf]$^{+3}$, calcd. 585.154), 952.217 (4%, [Fe_2**L** + 2OTf]$^{+2}$, calcd. 952.208).

FTIR (ATR, thin film from CD_3CN): $\tilde{\nu}$ [cm^{-1}] = 518.6 (w), 576.7 (vw), 638.6 (s) (δ_s $F_3C\mathbf{SO_3}$), 756.7 (vw), 792.4 (w), 894.5 (w), 1030.1 (vs) (ν_s $F_3C\mathbf{SO_3}$), 1163.0 (m) (ν_{as} $\mathbf{F_3C}SO_3$), 1258.1 (vs) (ν_{as} $F_3C\mathbf{SO_3}$), 1367.0 (w), 1466.9 (m), 2851.8 (vw), 2928.1 (vw), 3080.1 (vw).

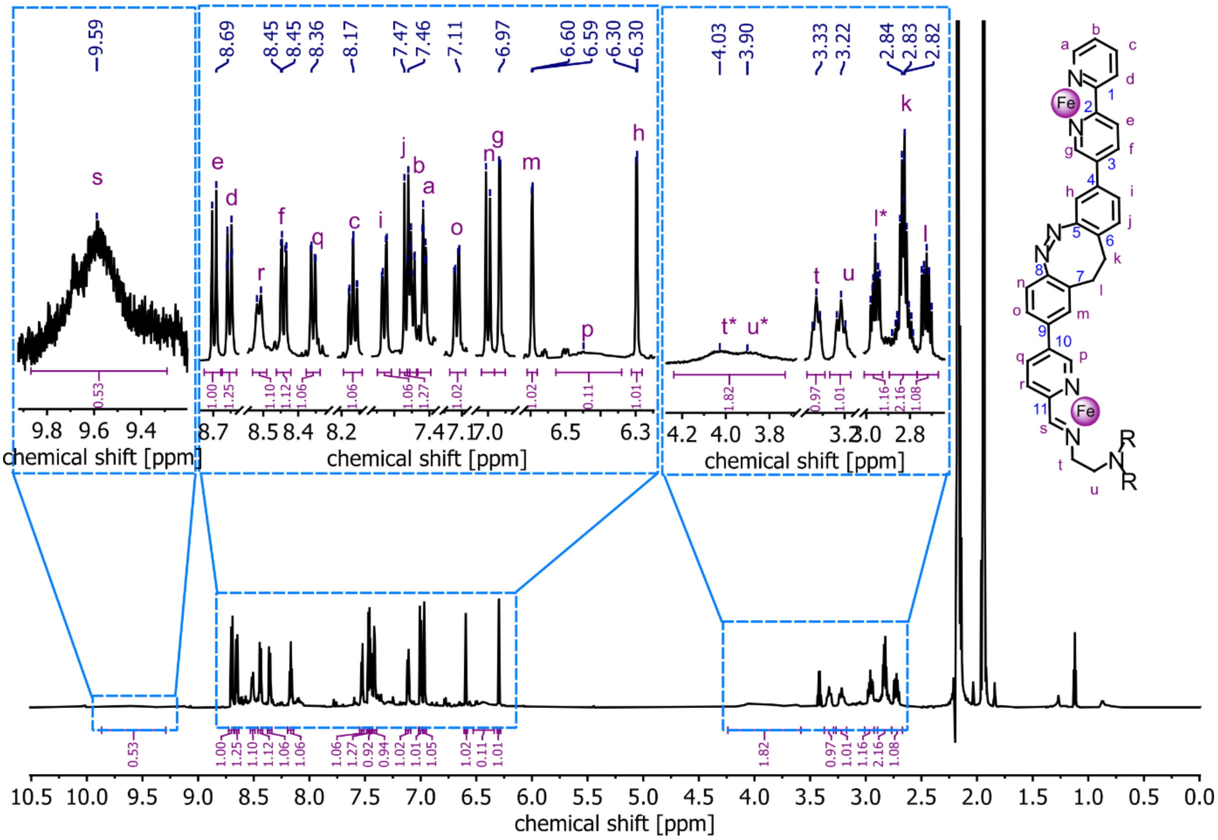

Figure S19. ^{1}H NMR spectrum (700 MHz, CD_3CN, 298 K) of Fe_2**L**.

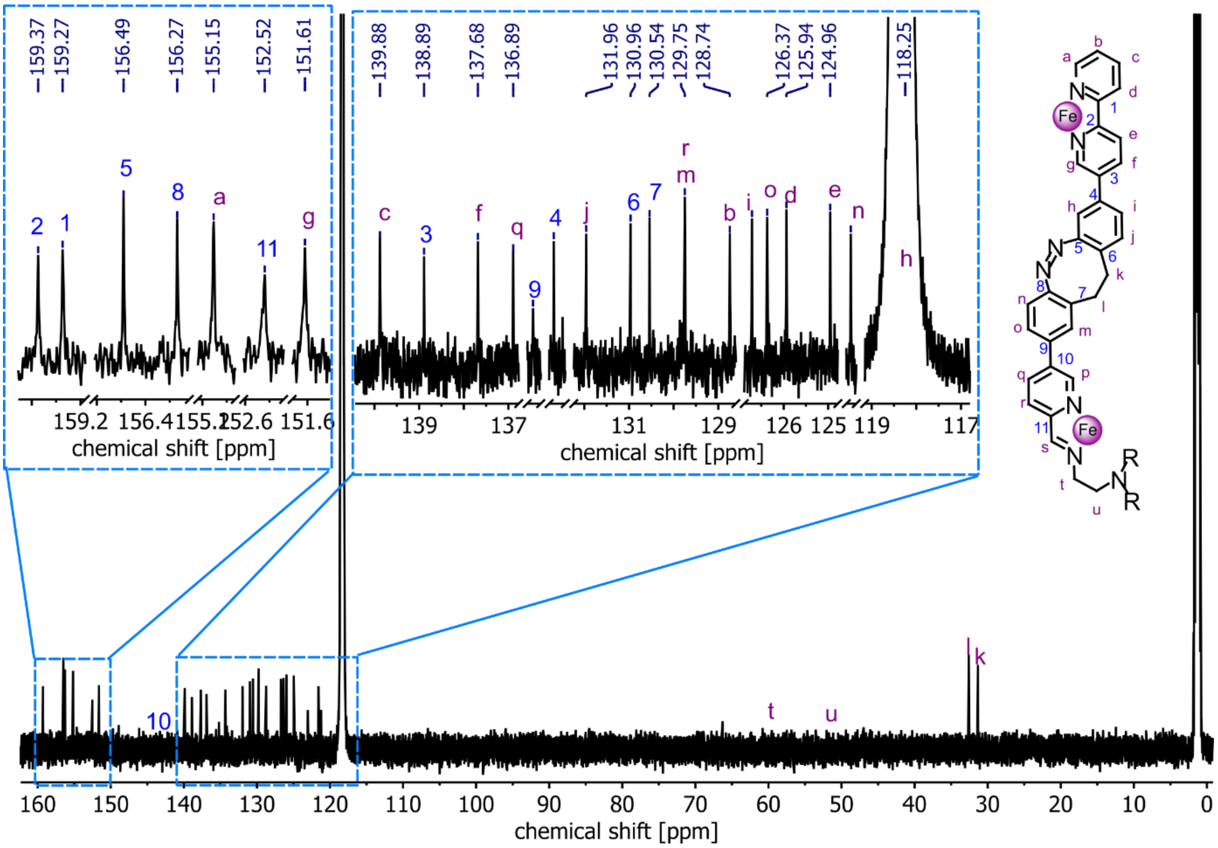

Figure S20. ^{13}C NMR spectrum (176 MHz, CD_3CN, 298 K) of Fe_2**L**.

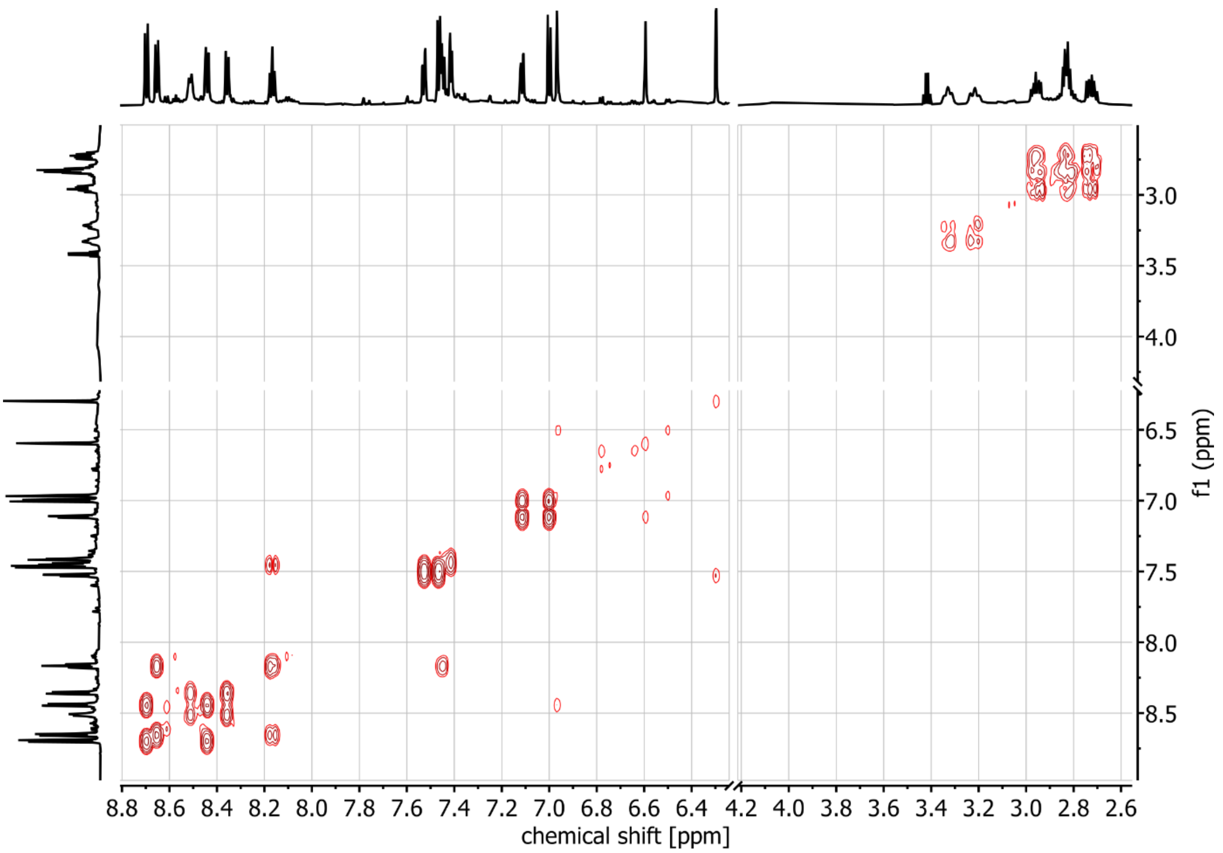

Figure S21. $^{1}H,^{1}H$ COSY spectrum (700 MHz, CD_3CN, 298 K) of Fe_2**L**.

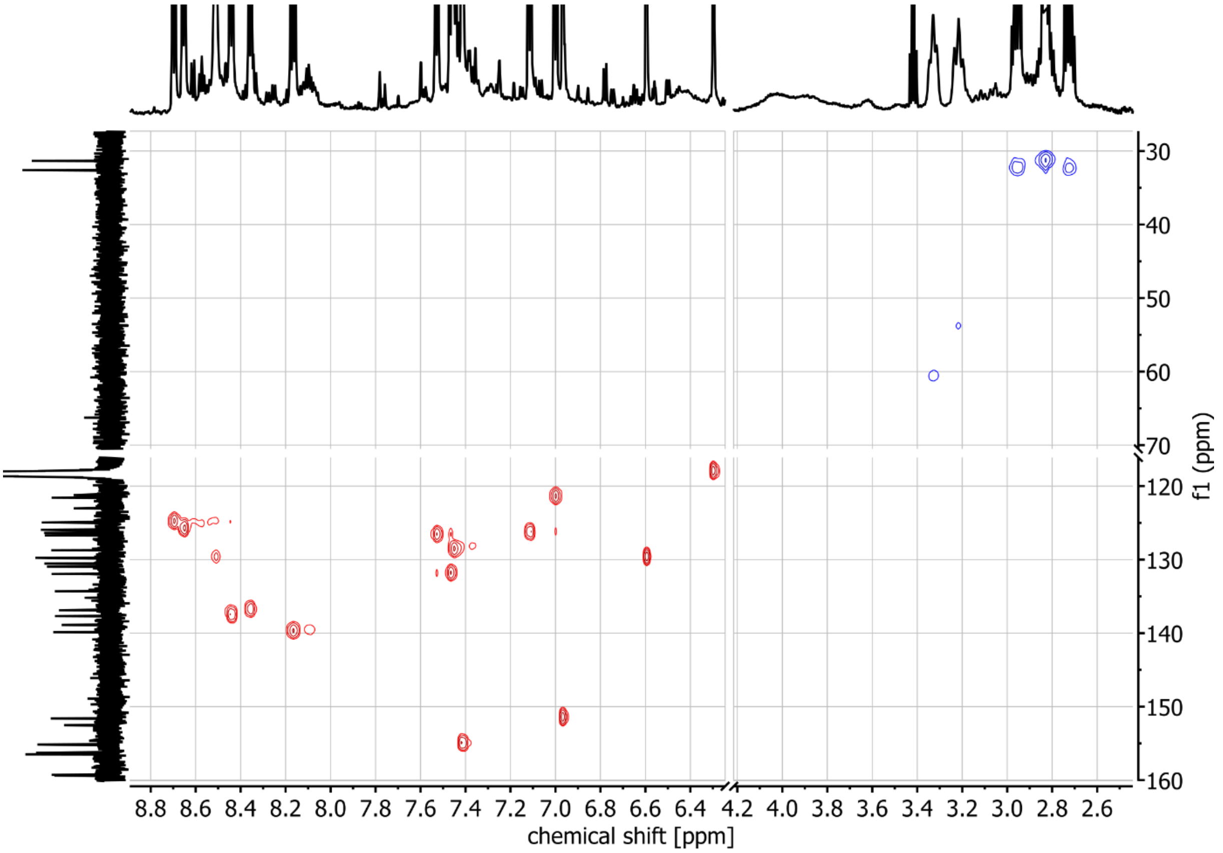

Figure S22. $^{1}H,^{13}C$ HSQC spectrum (700/176 MHz, CD_3CN, 298 K) of Fe_2**L**.

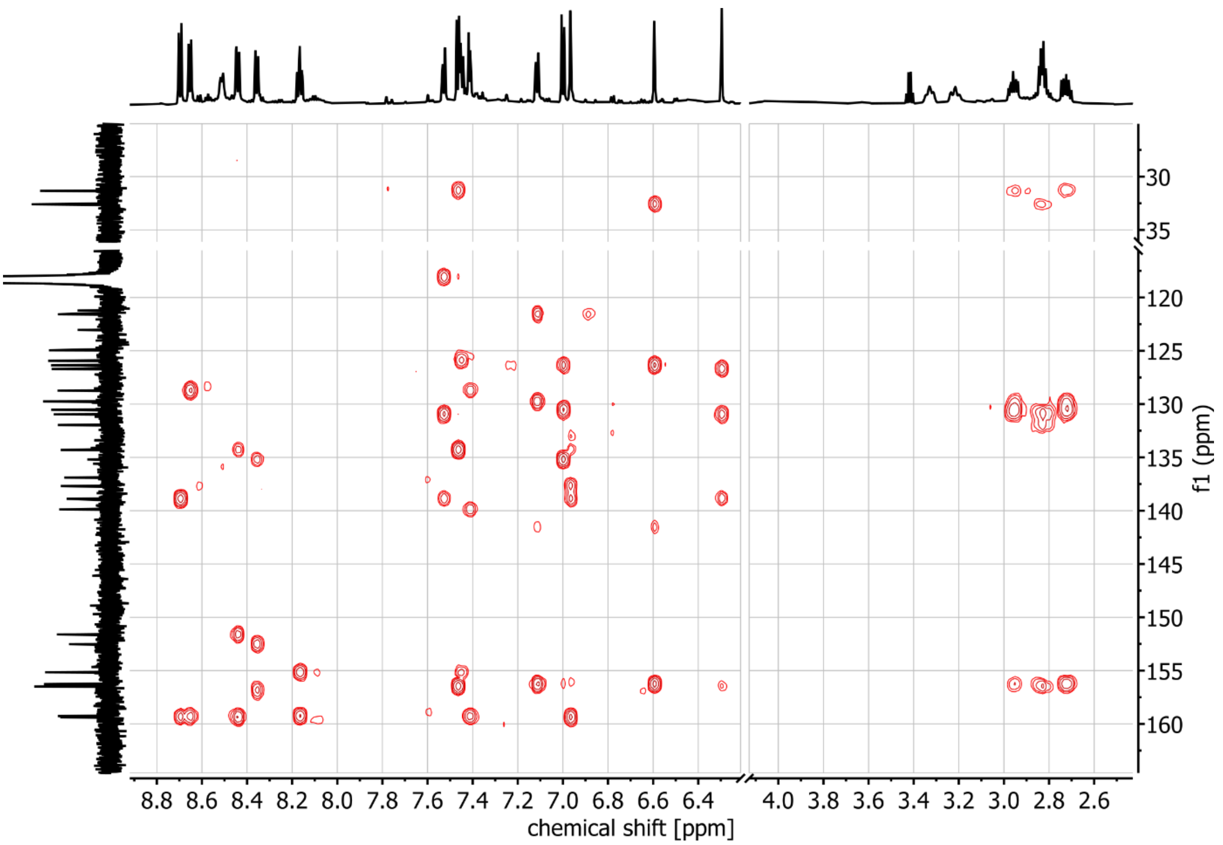

Figure S23. ^{1}H –^{13}C HMBC spectrum (700/176 MHz, CD_3CN, 298 K) of Fe_2**L**.

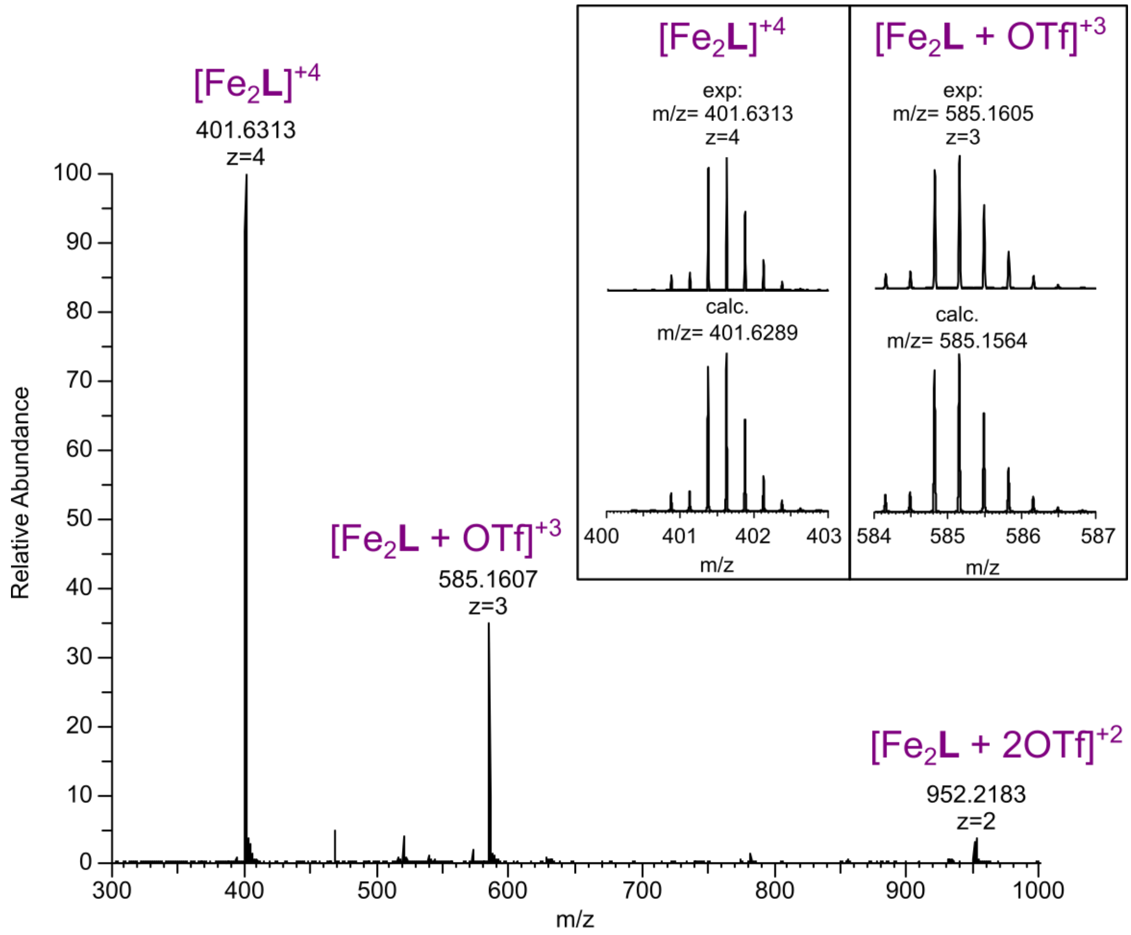

Figure S24. ESI^{+} mass spectrum (CH_3CN) of Fe_2**L**.

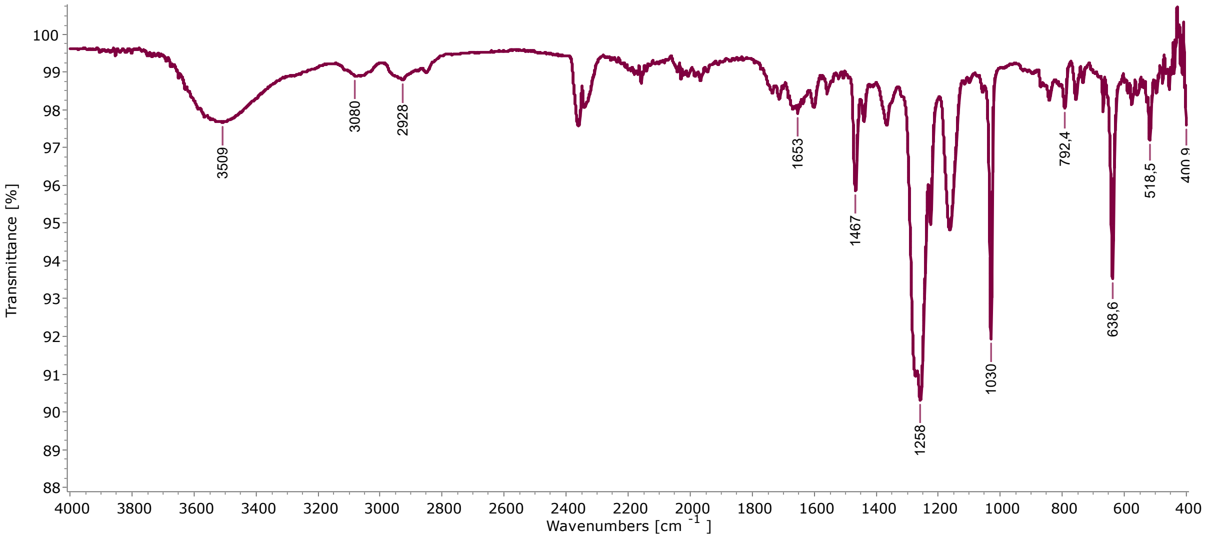

Figure S25. FTIR spectrum (ATR, thin film from evaporation of a CD_3CN solution) of Fe_2**L**.

S3.5 Co_2L

Co_2L 2as synthesized according to the general procedure (Section S3.2) using $Co(OTf)_2$ (1.20 mg, 3.4 µmol, 2.20 equiv.), aldehyde **1** (2.14 mg, 4.6 µmol, 3.00 equiv.), and TREN (0.22 mg, 1.5 µmol, 1.00 equiv.) with a reaction time of 1 day at a temperature of 70 °C. After precipitation the product was obtained as a yellow powder in 90% yield (1.92 mg, 0.81 µmol).

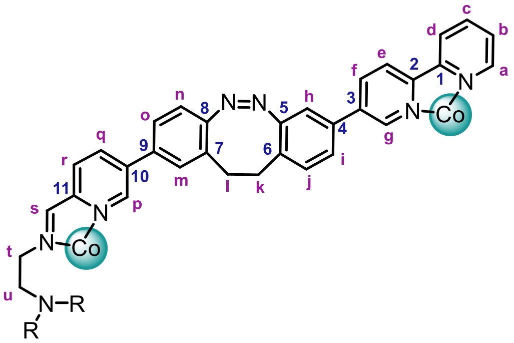

$[C_{96}H_{75}N_{19}Co_2](CF_3O_3S)_4$

^{1}H NMR (400 MHz, CD_3CN) δ [ppm] = 13.79 (H-c/f), 14.99 (H-c/f), 44.83 (H-b), 48.10 (H-q), 82.78 (H-d/e), 83.79 (H-d/e), 85.56 (H-a/g), 90.44 (H-a/g), 112.03 (H-r), 171.59 (H-s), 184.16 (H-p).

Due to the strong paramagnetism of the compound not all assignments could be made and not all signals could be observed.

HRMS (ESI$^+$ Orbitrap): *m/z* (relative intensity) = 402.8771 (100%, $[Co_2\mathbf{L}]^{+4}$, calcd. 402.8774), 586.8212 (15%, $[Co_2\mathbf{L} + OTf]^{+3}$, calcd. 586.8207).

MS (ESI$^+$Orbitrap): *m/z* (relative intensity) = 403.131 (100%, $[Co_2\mathbf{L}]^{+4}$, calcd. 403.8772), 519.927 (9%, $[Co_2\mathbf{L} + \mathbf{1}]^{+4}$, calcd. 519.930), 587.161 (58%, $[Co_2\mathbf{L} + OTf]^{+3}$, calcd. 587.163), 955.218 (2%, $[Co_2\mathbf{L}+ 2OTf]^{+2}$, calcd. 955.206).

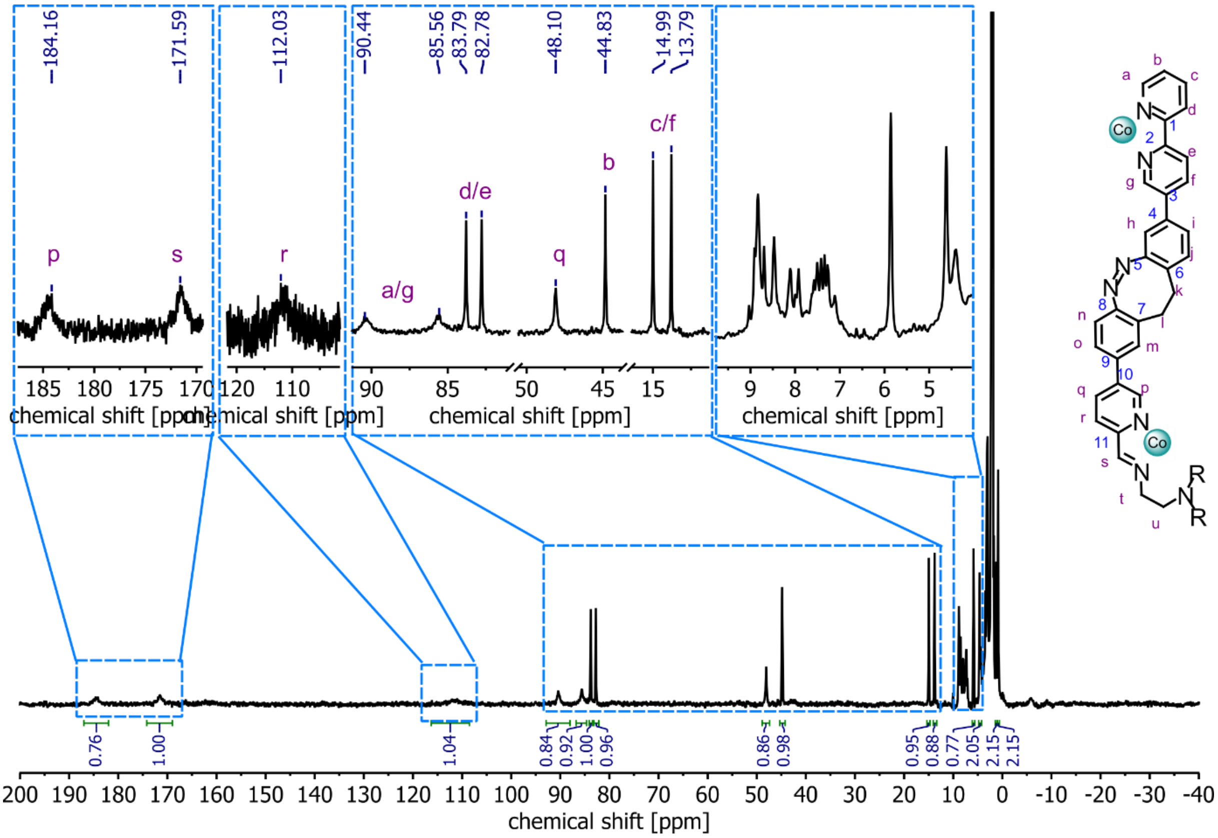

Figure S26. ^{1}H NMR spectrum (500 MHz, CD_3CN, 298 K) of Co_2**L**.

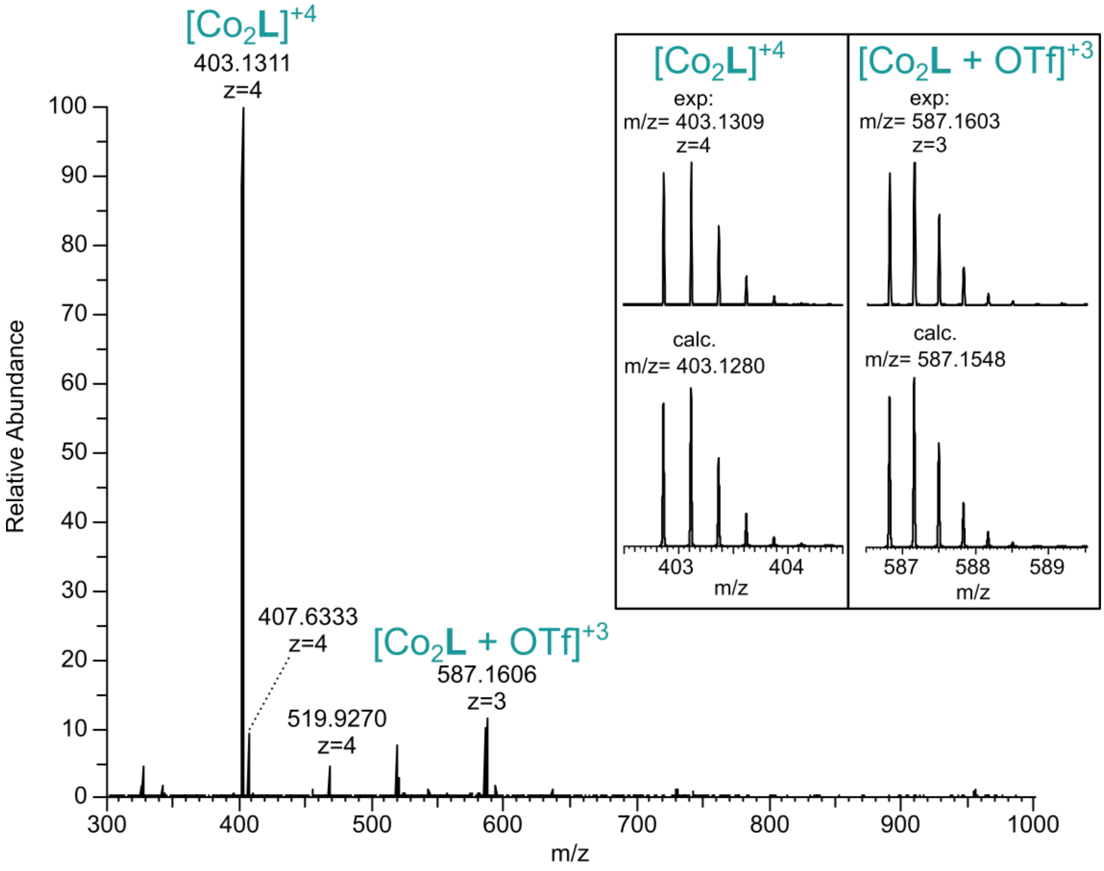

Figure S27. ESI^+ mass spectrum (CH_3CN) of Co_2**L**.

S3.6 FeL

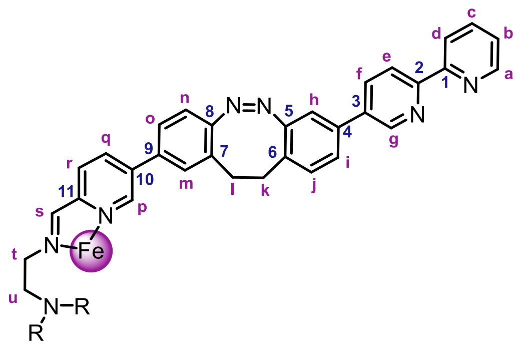

Fe**L** was synthesized according to the general procedure (Section S3.2) using $Fe(OTf)_2$ (0.34 mg, 0.96 µmol, 1.00 equiv.), aldehyde **1** (1.34 mg, 2.87 µmol, 3.00 equiv.), and TREN (0.14 mg, 0.96 µmol, 1.00 equiv.) with a reaction time of 1 day at a temperature of 70 °C. The crude product was used in the next step without workup.

Fe**L** exists as a mixture of conformational isomers with ^{1}H NMR and ESI(+) MS showing the formation of approximately 25% of the Fe_2**L** helicate as a side product.

HRMS (ESI$^+$ Orbitrap): *m*/*z* (relative intensity) = 774.7901 (100%, [Fe**L**]$^{+2}$, calcd. 774.7898).

MS (ESI$^+$ Orbitrap): *m*/*z* (relative intensity) = 401.628 (57%, [Fe_2**L**]$^{+4}$, calcd. 401.629), 585.156 (18%, [Fe_2**L** + OTf]$^{+3}$, calcd. 585.154), 775.289 (100%, [Fe**L**]$^{+2}$, calcd. 775.288), 952.210 (5%, [Fe_2**L** + 2OTf]$^{+2}$, calcd. 952.208), 1699.535 (5%, [Fe**L** + OTf]$^+$, calcd. 1699.528).

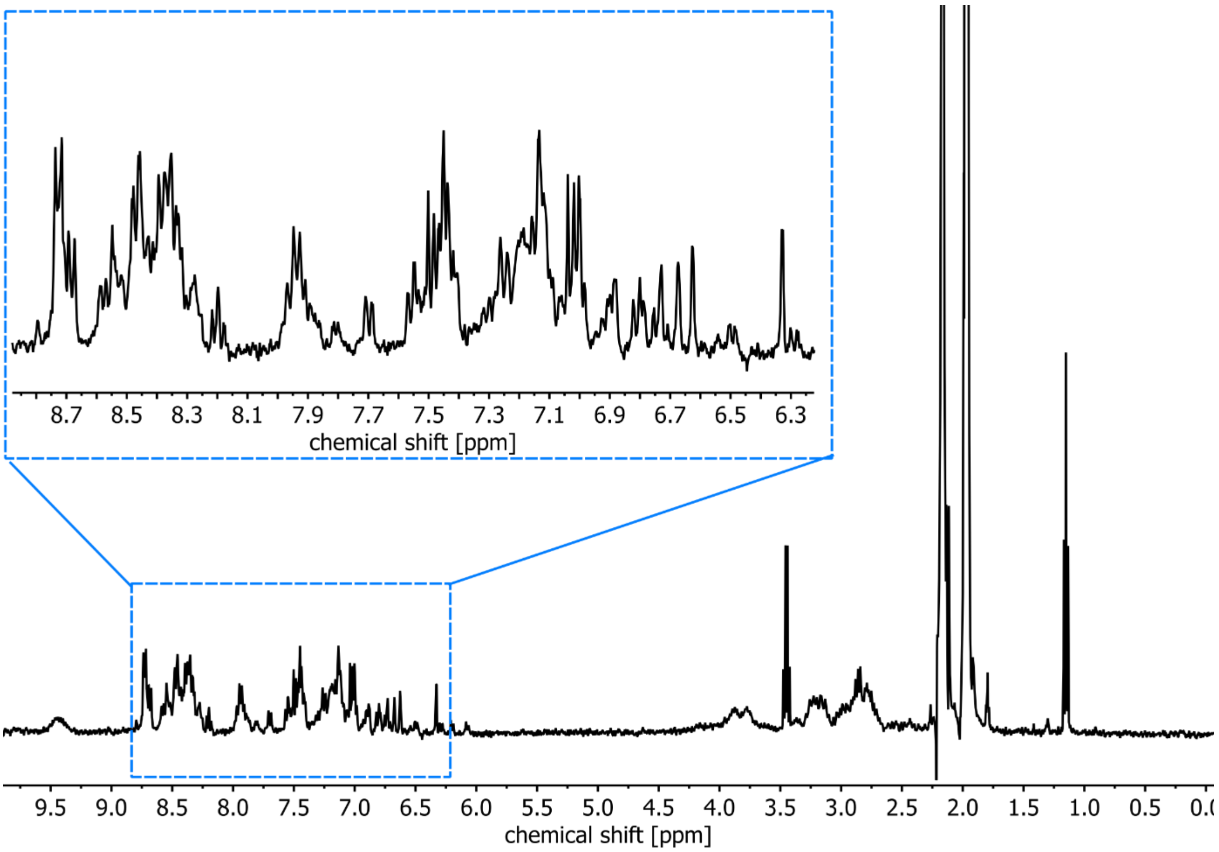

Figure S28. ^{1}H NMR spectrum (400 MHz, CD_3CN, 298 K) of Fe**L**.

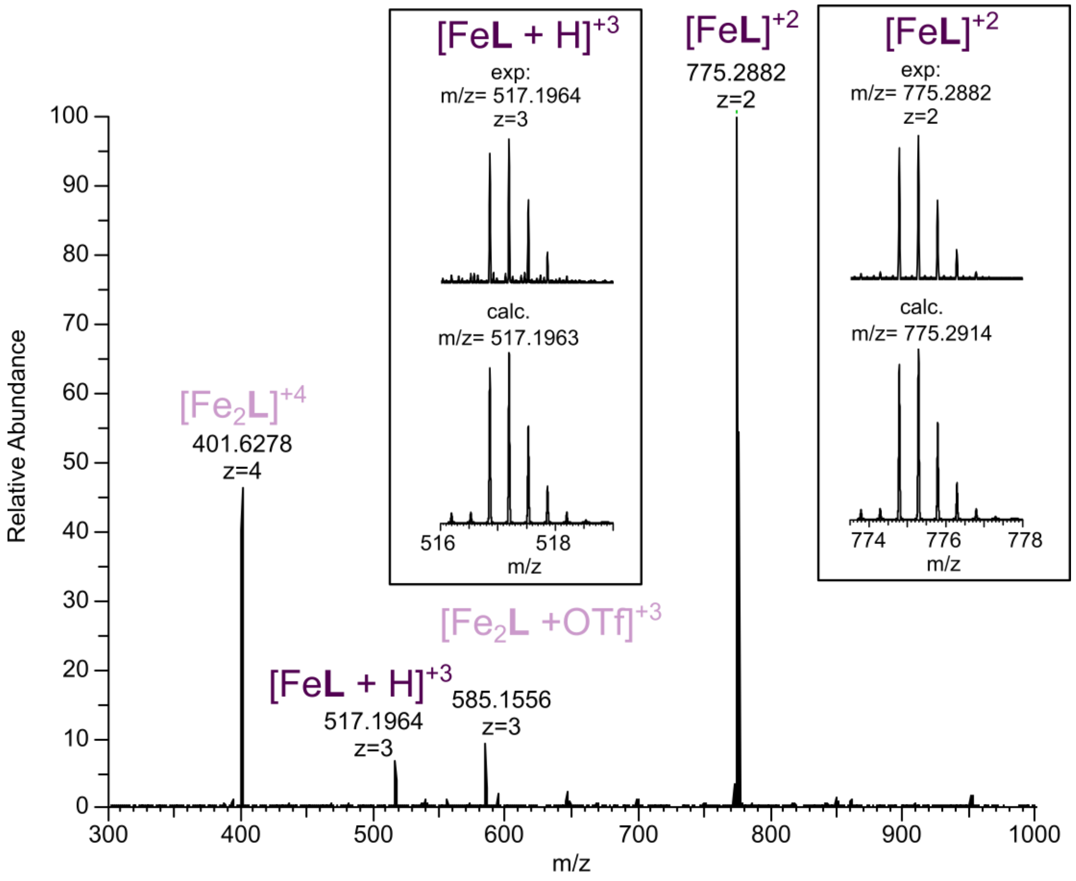

Figure S29. ESI⁺ mass spectrum (CH_3CN) of Fe**L**.

S3.7 FeZn**L**

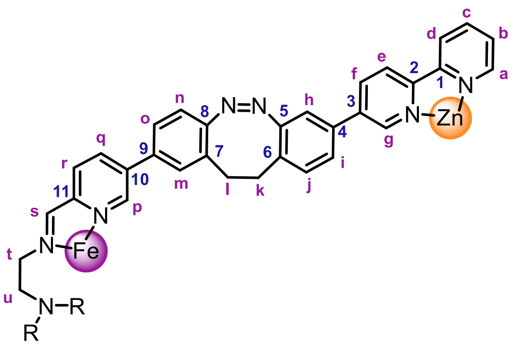

[$C_{96}H_{75}N_{19}ZnFe$](CF_3O_3S)$_4$

FeZn**L** was synthesized according to the general procedure (Section S3.2) using crude Fe**L** (approx. 0.96 µmol, 1.00 equiv.) and Zn(OTf)$_2$ (0.35 mg, 0.96 µmol, 1.00 equiv.) with a reaction time of 1 hour at a temperature of 30 °C. Even after precipitation no pure product could be obtained, only a mixture containing 65% of the desired FeZn**L** with 25% Fe$_2$**L** and 10% Zn$_2$**L** present.

^{1}H NMR (400 MHz, CD_3CN) δ [ppm] = 2.76 (dd, J = 15.4, 6.7 Hz, 3H), 2.80 – 2.88 (m, 6H), 2.94 (dd, J = 10.5, 5.6 Hz, 6H), 3.23 (s, 3H), 3.35 (s, 3H), 4.09 (s, 6H), 6.42 (s, 3H), 6.5 (s (br), 3H), 6.64 (s, 3H), 6.95 (d, J = 8.1 Hz, 3H), 7.08 (d, J = 8.3 Hz, 3H), 7.42 (s, 3H), 7.50 (d, J = 8.5 Hz, 3H), 7.55 (t, J = 9.2 Hz, 3H), 7.59 – 7.65 (m, 3H), 8.03 (d, J = 4.4 Hz, 3H), 8.30 (t, J = 7.1 Hz, 3H), 8.37 (t, J = 6.9 Hz, 3H), 8.54 (d, J = 7.0 Hz, 6H), 8.66 (d, J = 8.2 Hz, 3H), 8.70 (d, J = 8.5 Hz, 3H), 9.7 (s (br), 3H).

Since the helicates could not be separated and a significant signal overlap was observed, no assignments were made in the ^{1}H NMR spectrum.

HRMS (ESI$^+$ Orbitrap): m/z (relative intensity) = 591.4826 (100%, [FeZn**L** + OTf]$^{+3}$, calcd. 591.4843).

MS (ESI$^+$ Orbitrap): m/z (relative intensity) = 401.631 (80%, [Fe$_2$**L**]$^{+4}$, calcd. 401.629), 403.878 (100%, [FeZn**L**]$^{+4}$, calcd. 403.877), 406.374 (10%, [Zn$_2$**L**]$^{+4}$, calcd. 405.375), 585.159 (44%, [Fe$_2$**L** + OTf]$^{+3}$, calcd. 585.156), 588.155 (68%, [FeZn**L** + OTf]$^{+3}$, calcd. 588.154), 591.485 (10%, [Zn$_2$**L** + OTf]$^{+3}$, calcd. 591.484), 952.214 (6%, [Fe$_2$**L** + 2OTf]$^{+2}$, calcd. 952.215), 956.710 (9%, [FeZn**L** + 2OTf]$^{+2}$, calcd. 956.705), 961.705 (1%, [Zn$_2$**L** + 2OTf]$^{+2}$, calcd. 961.700).

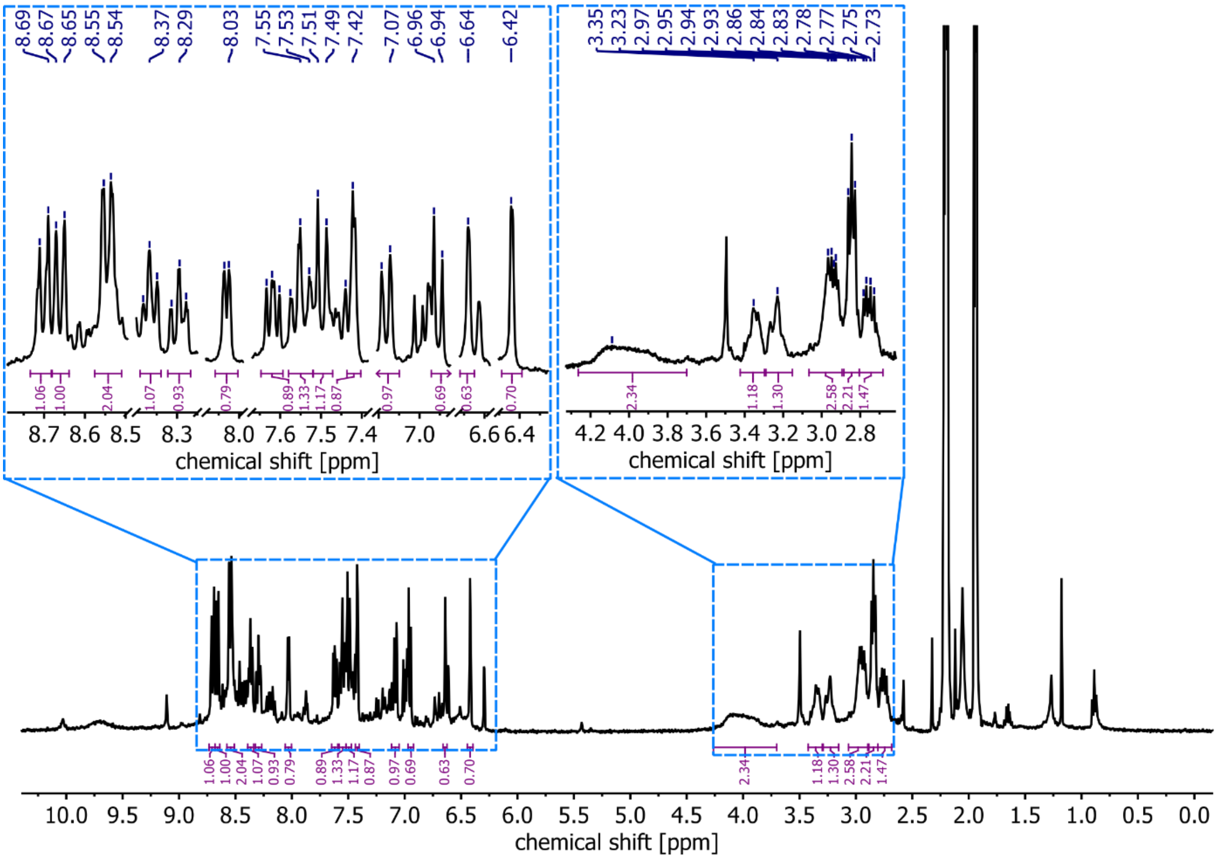

Figure S30. ^{1}H NMR spectrum (400 MHz, CD_3CN, 298 K) of FeZn**L**.

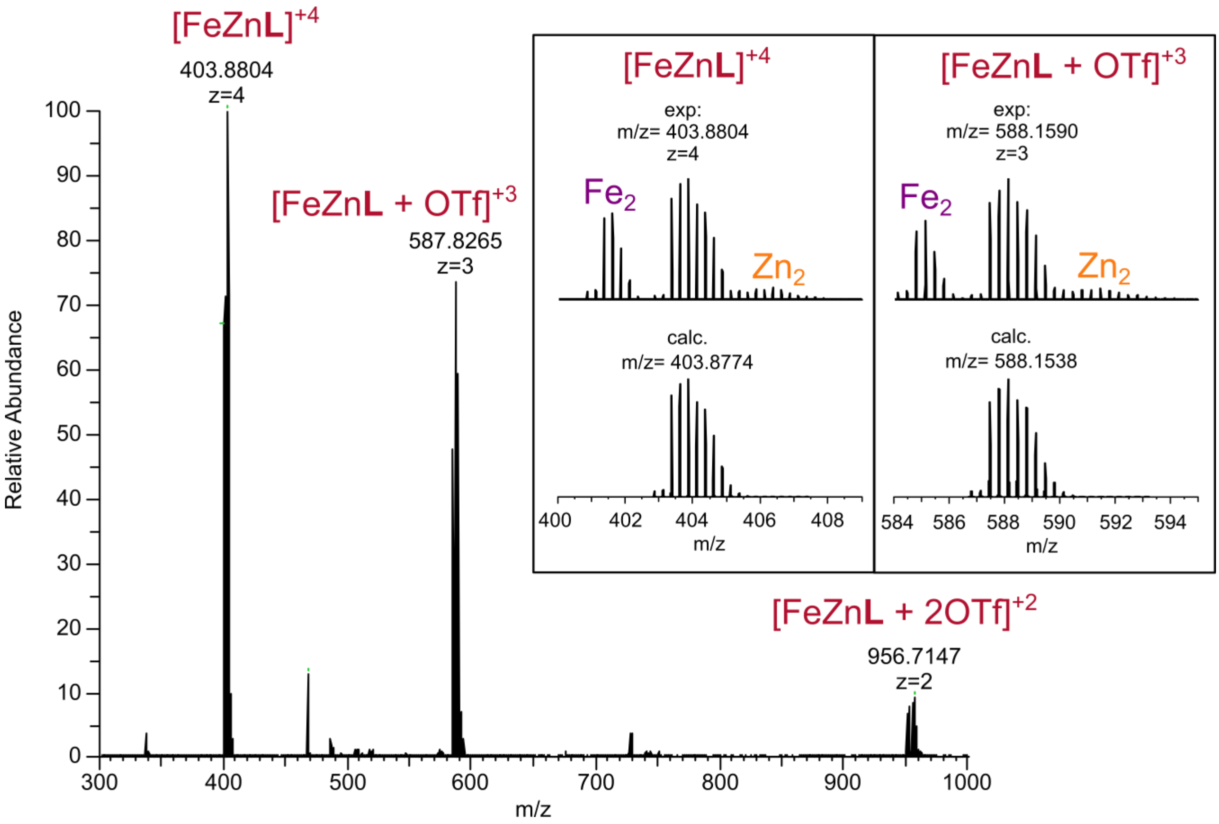

Figure S31. ESI(+) mass spectrum of FeZn**L** (CH_3CN).

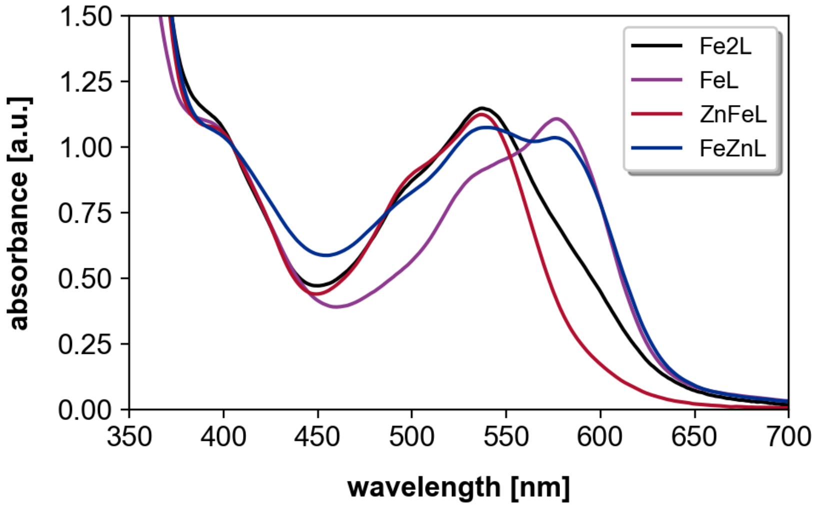

Figure S32. UV-vis spectra (CH_3CN, 0.06 mM) of Fe_2**L**, Fe**L**, ZnFe**L**, and FeZn**L**.

S3.8 ZnFe**L** via Zn→Fe exchange

ZnFe**L** was synthesized by adding $Fe(OTf)_2$ (0.44 mg, 1.23 µmol, 1.50 equiv.) to a solution of Zn_2**L** (1.98 mg, 0.82 µmol, 1.00 equiv.) in CD_3CN and heating the mixture to 65 °C for 2 days. The transformation occurred quantitatively.

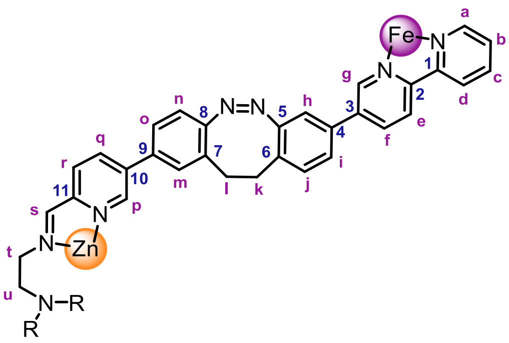

$[C_{96}H_{75}N_{19}ZnFe](CF_3O_3S)_4$

^{1}H NMR (700 MHz, CD_3CN) δ [ppm] = 2.70 – 2.77 (m, 3H, H-l), 2.80 – 2.89 (m, 6H, H-k), 2.91 – 2.99 (m, 6H, H-l',u), 3.25 (dd, J = 13.7, 3.9 Hz, 3H, H-u'), 3.67 (dd, J = 12.0, 3.6 Hz, 3H, H-t), 3.85 (t, J = 11.6 Hz, 3H, H-t'), 6.31 (d, J = 2.0 Hz, 3H, H-h), 6.50 (d, J = 2.2 Hz, 3H, H-p), 6.69 (d, J = 2.0 Hz, 3H, H-m), 6.95 (d, J = 8.1 Hz, 3H, H-n), 7.06 (d, J = 2.0 Hz, 3H, H-g), 7.09 (dd, J = 8.1, 2.0 Hz, 3H, H-o), 7.41 (dd, J = 5.7, 1.5 Hz, 3H, H-a), 7.45 (ddd, J = 7.4, 5.7, 1.3 Hz, 3H, H-b), 7.48 (d, J = 8.0 Hz, 3H, H-j), 7.58 (dd, J = 8.0, 2.0 Hz, 3H, H-i), 8.16 (td, J = 7.8, 1.5 Hz, 3H, H-c), 8.19 (d, J = 8.0 Hz, 3H, H-r), 8.42 (dd, J = 8.1, 2.3 Hz, 3H, H-q), 8.46 (dd, J = 8.5, 2.1 Hz, 3H, H-f), 8.65 (dt, J = 8.2, 1.0 Hz, 3H, H-d), 8.69 (d, J = 8.5 Hz, 3H, H-e), 8.79 (d, J = 1.9 Hz, 3H, H-s).

^{13}C NMR (176 MHz, CD_3CN) δ [ppm] = 31.0 (C-k), 32.3 (C-l), 54.6 (C-u), 56.3 (C-t), *118.2*[**] *(C-h),* 121.4 (C-n), 124.4 (C-e), 125.5 (C-d), 125.6 (C-o), 126.3 (C-i), 128.3 (C-b), 129.1 (C-r), 129.3 (C-m), 129.9 (C-7), 130.3 (C-6), 131.5 (C-j), 134.0 (C-4), 134.4 (C-9), 137.0 (C-f), 138.3 (C-3), 138.9 (C-q), 139.4 (C-c), 140.5 (C-10), 145.8 (C-p), 146.9 (C-11), 152.0 (C-g), 154.7 (C-a), 155.8 (C-8), 156.0 (C-5), 158.8 (C-2), 159.0 (C-1), 163.1 (C-s).

HRMS (ESI$^+$ Orbitrap): m/z (relative intensity) = 403.8763 (100%, $[ZnFe\mathbf{L}]^{4+}$, calcd. 403.8772).

[**] The signal for C-h is underneath the solvent peak and could only be found by its cross-peak in ^{1}H,^{13}C HSQC NMR.

MS (ESI^{+} Orbitrap): m/z (relative intensity) = 403.880 (100%, [ZnFe**L**]$^{+4}$, calcd. 403.877), 588.159 (58%, [ZnFe**L** + OTf]$^{3+}$, calcd. 588.152), 956.718 (9%, [ZnFe**L** + 2OTf]$^{2+}$, calcd. 956.705).

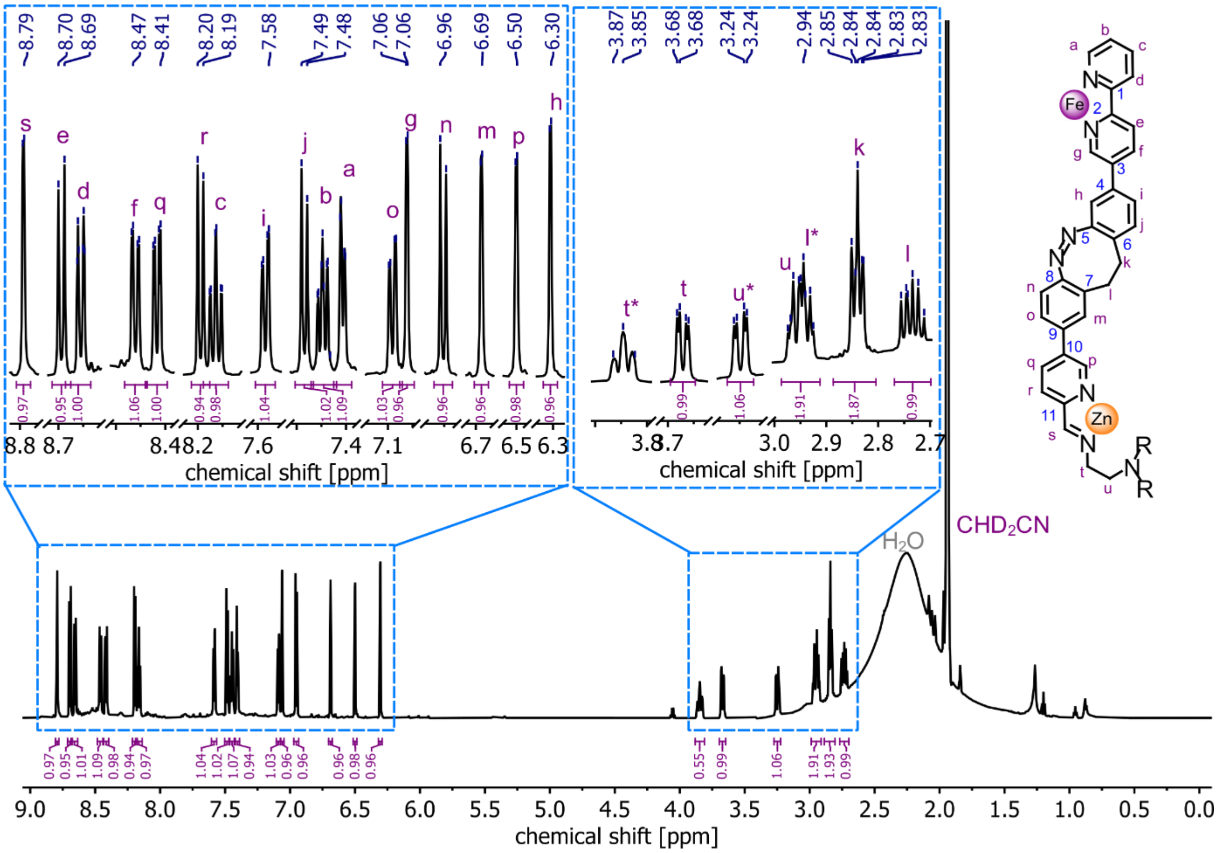

Figure S33. ^{1}H NMR spectrum (700 MHz, CD_3CN, 298 K) of ZnFe**L**.

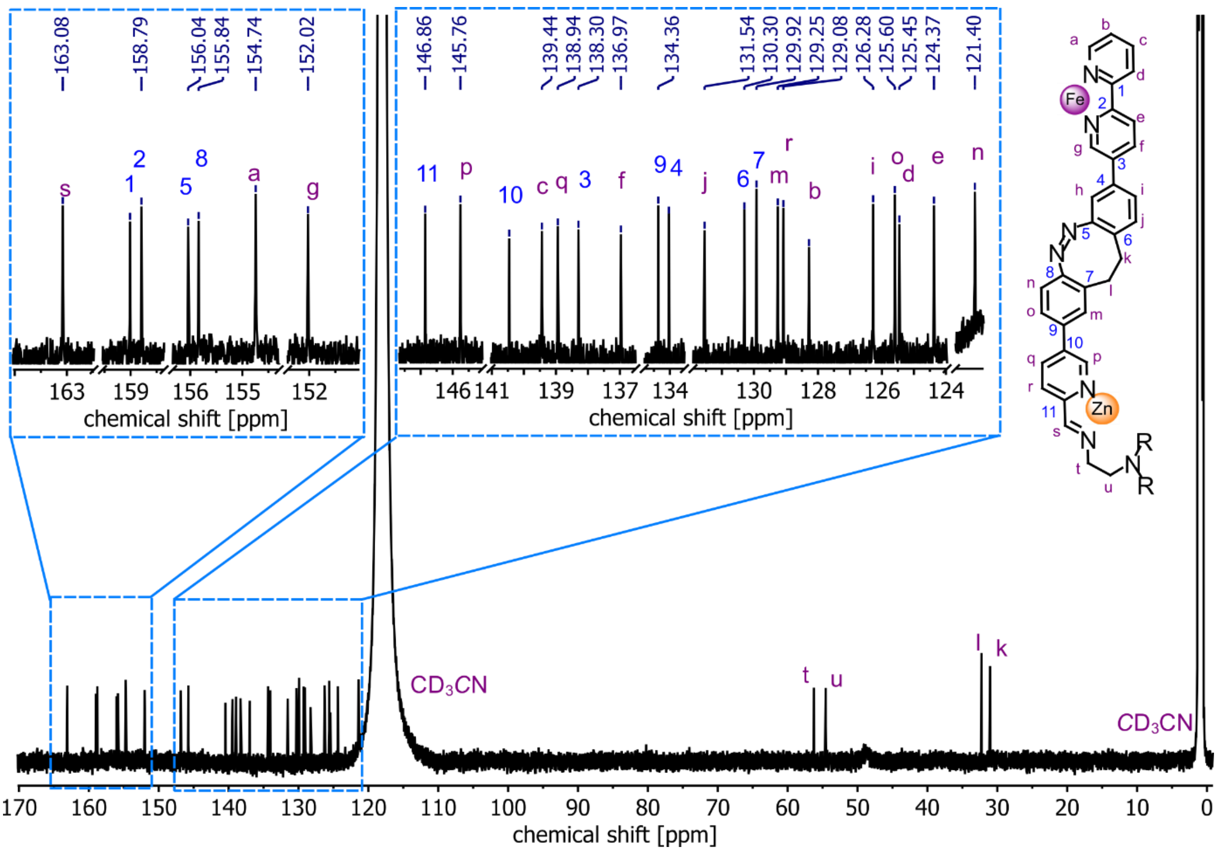

Figure S34. ^{13}C NMR spectrum (176 MHz, CD_3CN, 298 K) of ZnFe**L**.

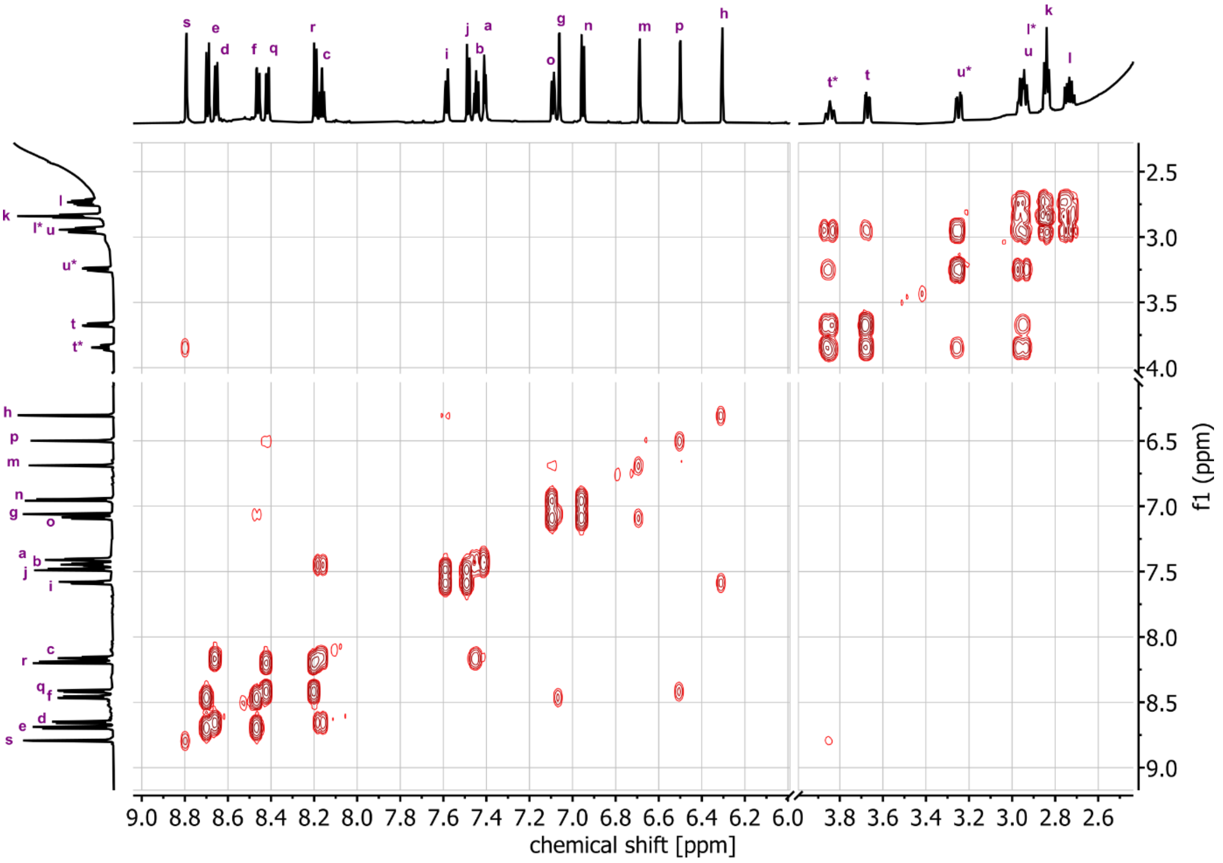

Figure S35. $^{1}H,^{1}H$ COSY spectrum (700 MHz, CD_3CN, 298 K) of ZnFe**L**.

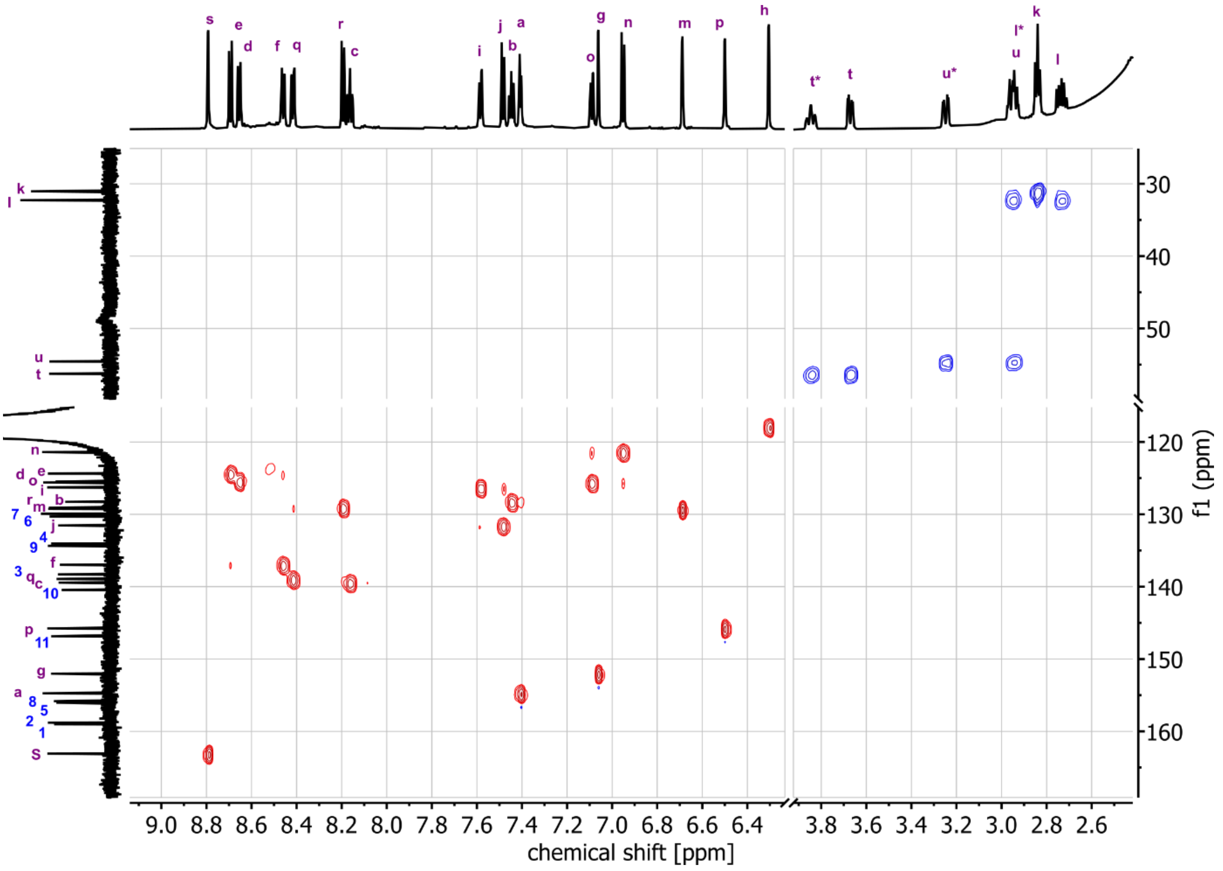

Figure S36. $^{1}H,^{13}C$ HSQC spectrum (700/176 MHz, CD_3CN, 298 K) of ZnFe**L**.

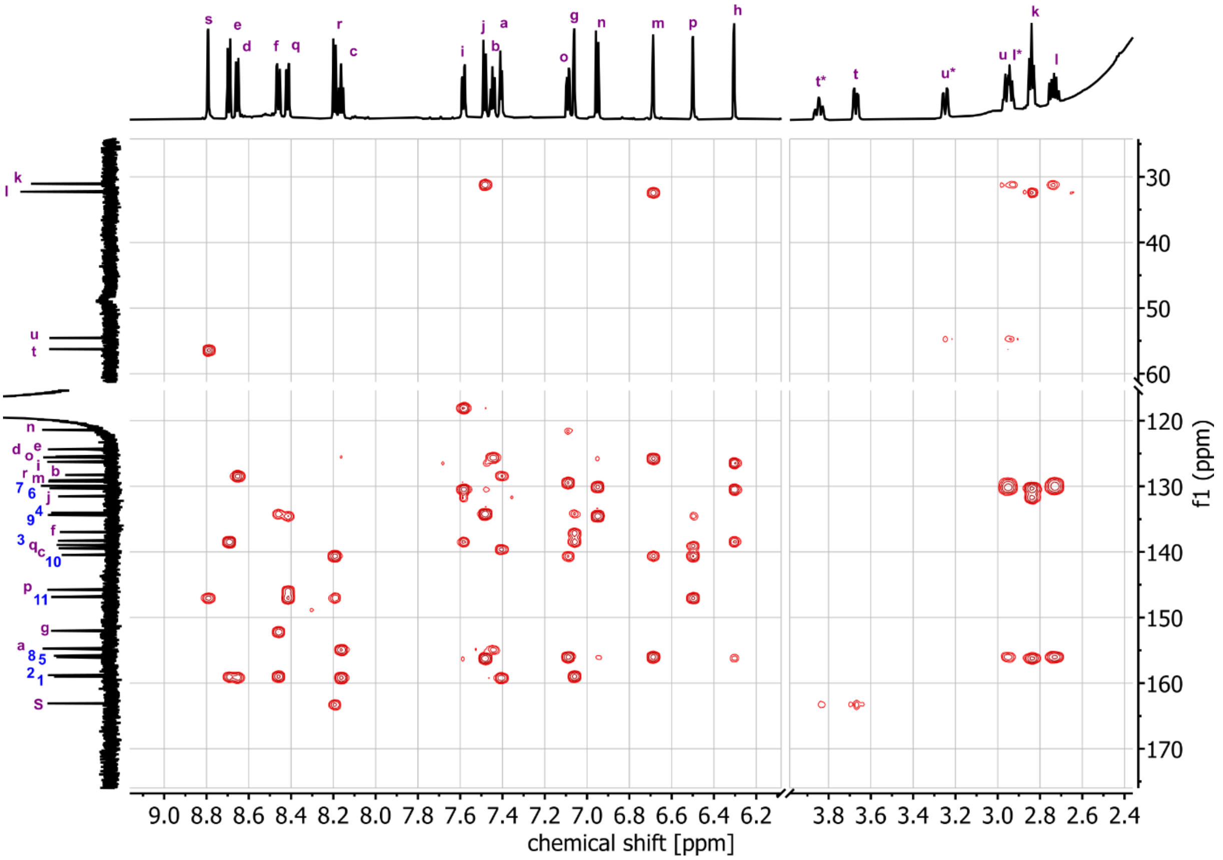

Figure S37. $^{1}H,^{13}C$ HMBC spectrum (700/176 MHz, CD_3CN, 298 K) of ZnFe**L**.

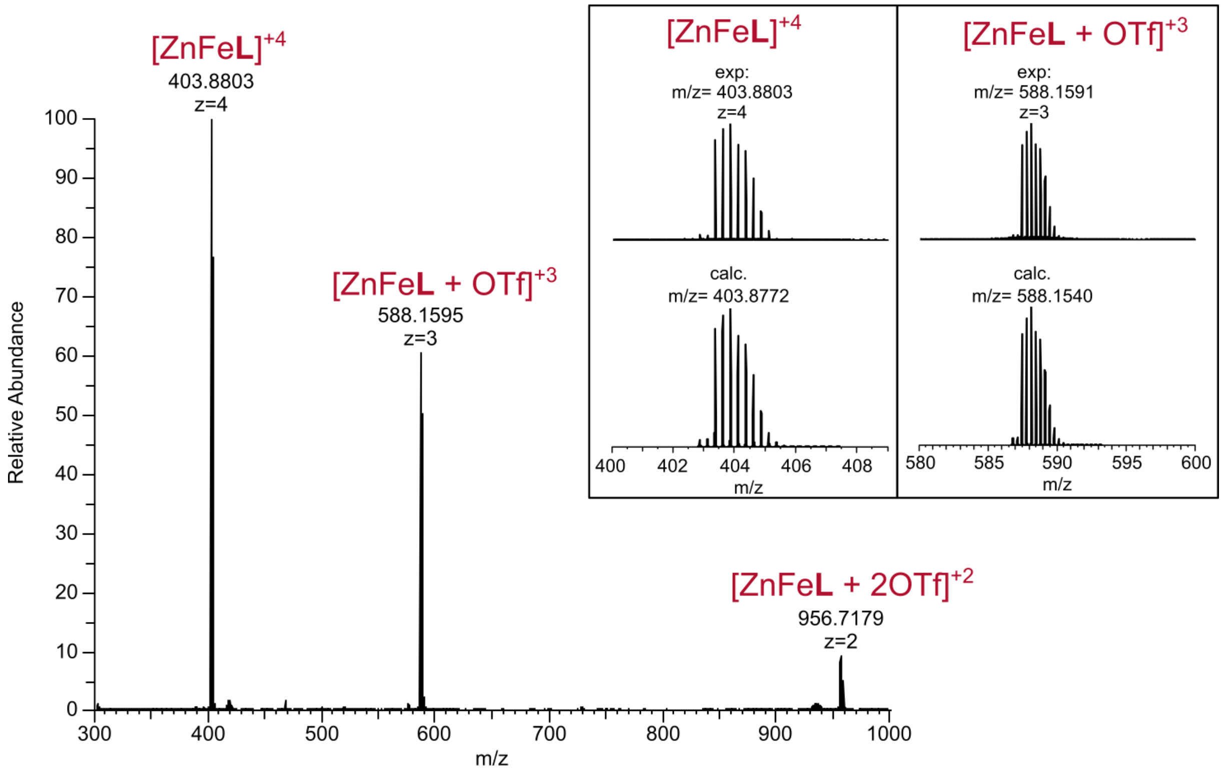

Figure S38. ESI^+ mass spectrum (CH_3CN) of FeZn**L**.

S4 Heterobimetallic self-sorting

S4.1 Experimental procedures

The self-sorting experiments were carried out as described in the general procedure from Section S3.2 with the samples being investigated by ^{1}H NMR, ESI$^+$ MS, and UV-vis without any further workup.

S4.1.1 ZnFe**L**

ZnFe**L** was synthesized according to the general procedure (Section S3.2) using $Zn(OTf)_2$ (0.39 mg, 1.07 µmol, 1.00 equiv.), $Fe(OTf)_2$ (0.38 mg, 1.07 µmol, 1.00 equiv.), aldehyde **1** (1.50 mg, 3.21 µmol, 3.00 equiv.), and TREN (0.16 mg, 1.07 µmol, 1.00 equiv.) with a reaction time of 1 day at a temperature of 70 °C. This experiment yields exclusively the desired ZnFe**L** helicate, as evidenced by ESI MS and ^{1}H NMR (Main text, Figure 2, bottom).

After precipitation the product was obtained as a dark red powder in 89% yield (2.54 mg, 0.95 µmol).

Analytical data of ZnFe**L** obtained via one-pot self-sorting is the same as for ZnFe**L** obtained from Zn_2**L** via Zn→Fe metal exchange (see Section S3.8).

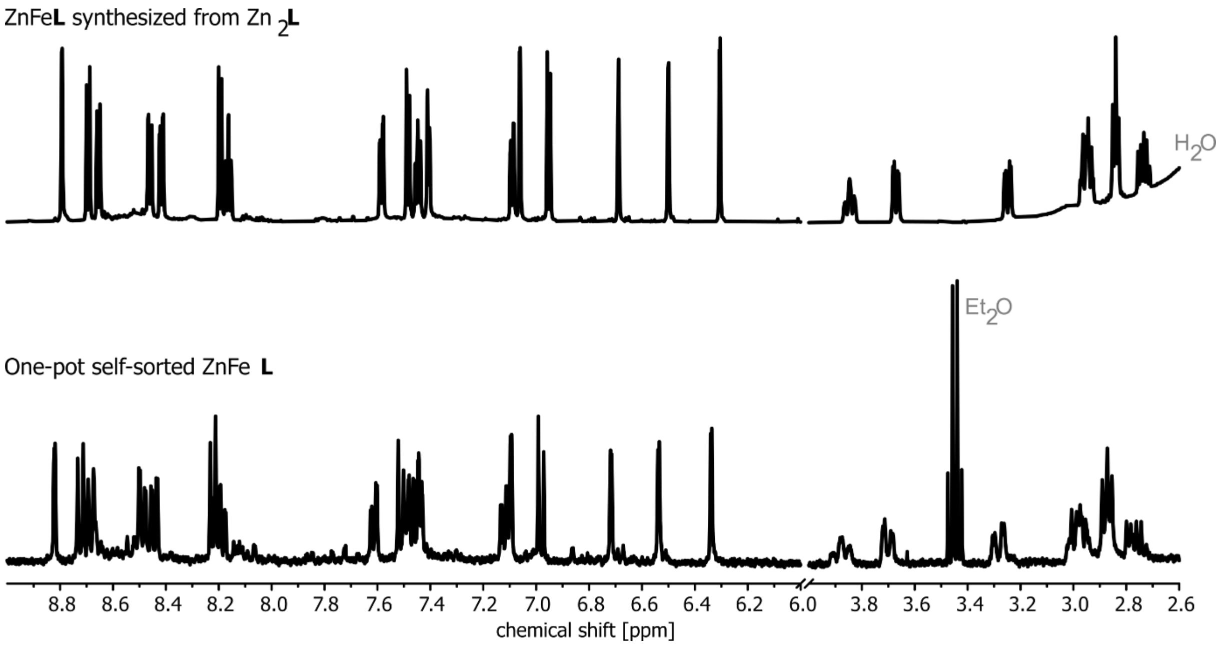

Figure S39. ^{1}H NMR spectra (CD_3CN, 298 K) of ZnFe**L** synthesized from Zn_2**L** via metal exchange (top, 700 MHz) and by one-pot self-sorting (bottom, 400 MHz).

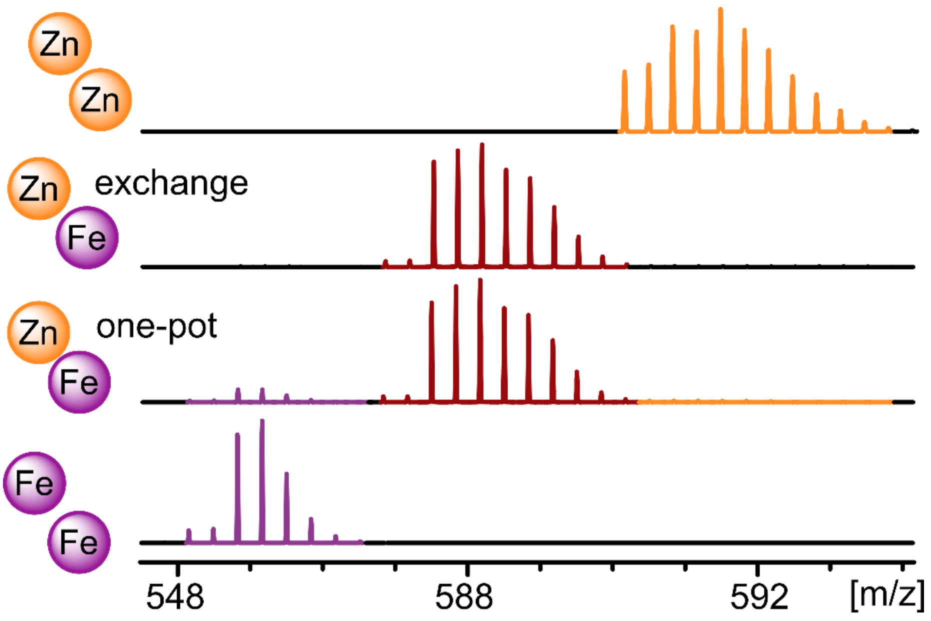

Figure S 40. ESI^{+} mass spectra (CH_3CN) of Zn_2**L**, Fe_2**L**, and FeZn**L** synthesized via metal exchange and by one-pot self-sorting.

S4.1.2 ZnCo**L**

ZnCo**L** was synthesized according to the general procedure (Section S3.2) using $Zn(OTf)_2$ (0.67 mg, 1.85 µmol, 1.00 equiv.), $Co(OTf)_2$ (0.66 mg, 1.85 µmol, 1.00 equiv.), aldehyde **1** (2.60 mg, 5.56 µmol, 3.00 equiv.), and TREN (0.27 mg, 1.85 µmol, 1.00 equiv.) with a reaction time of 1 day at a temperature of 70 °C.

This experiment yields exclusively the desired ZnCo**L** helicate, as evidenced by ESI MS and the absence of the characteristic broadened ^{1}H NMR signals typically observed for Co^{TREN} are absent (Section S4.4).

$[C_{96}H_{75}N_{19}CoZn](CF_3O_3S)_4$

^{1}H NMR (500 MHz, CD_3CN) δ [ppm] = 14.26 (H-c/f), 15.11 (H-c/f), 45.03 (H-b), 83.01 (H-d/e), 83.75 (H-d/e), 86.59 (H-a/g), 91.58 (H-a/g).

Due to the strong paramagnetism of the compound not all assignments could be made and not all signals could be observed.

HRMS (ESI^{+} Orbitrap): *m/z* (relative intensity) = 404.6277 (100%, [ZnCo**L**]$^{+4}$, calcd. 404.6267), 589.1548 (15%, [ZnCo**L** + OTf]$^{+3}$, calcd. 589.3150).

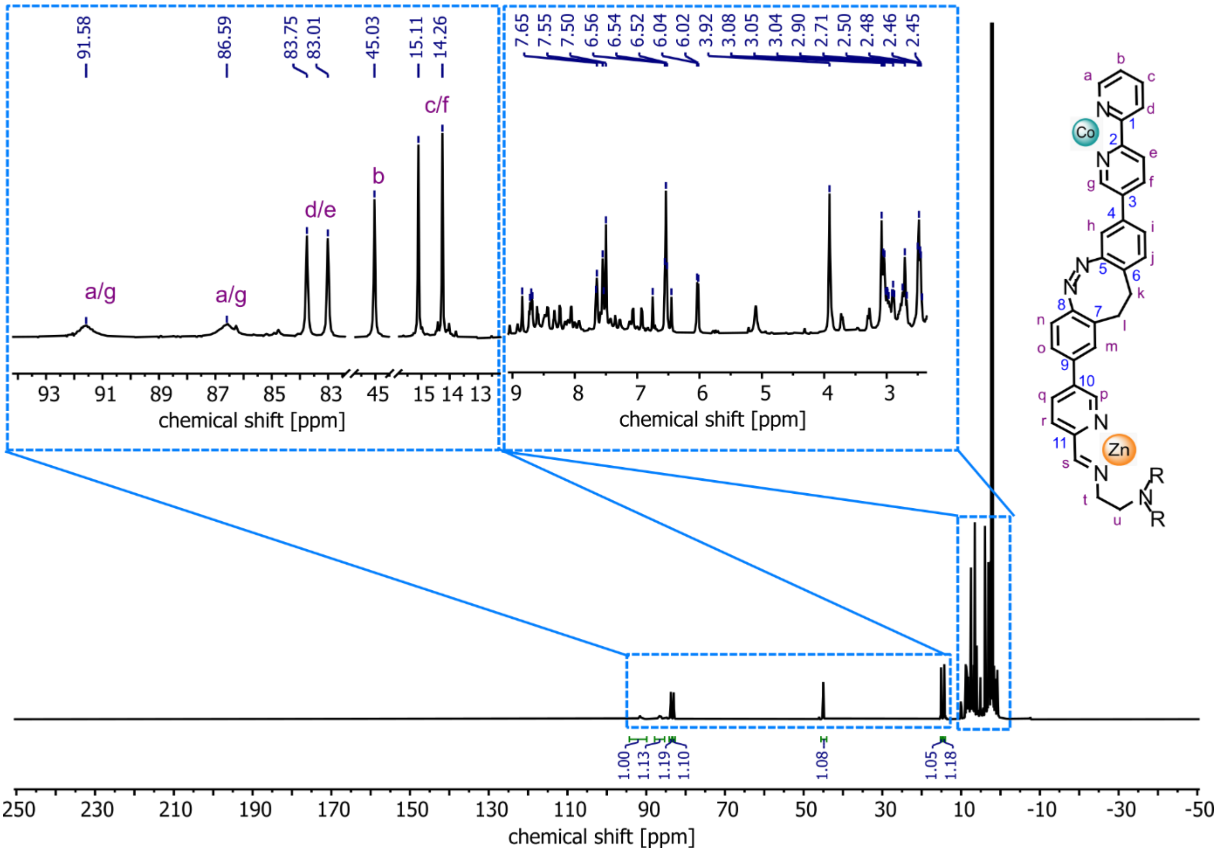

Figure S41. ^{1}H NMR spectrum (500 MHz, CD_3CN, 298 K) of ZnCo**L**.

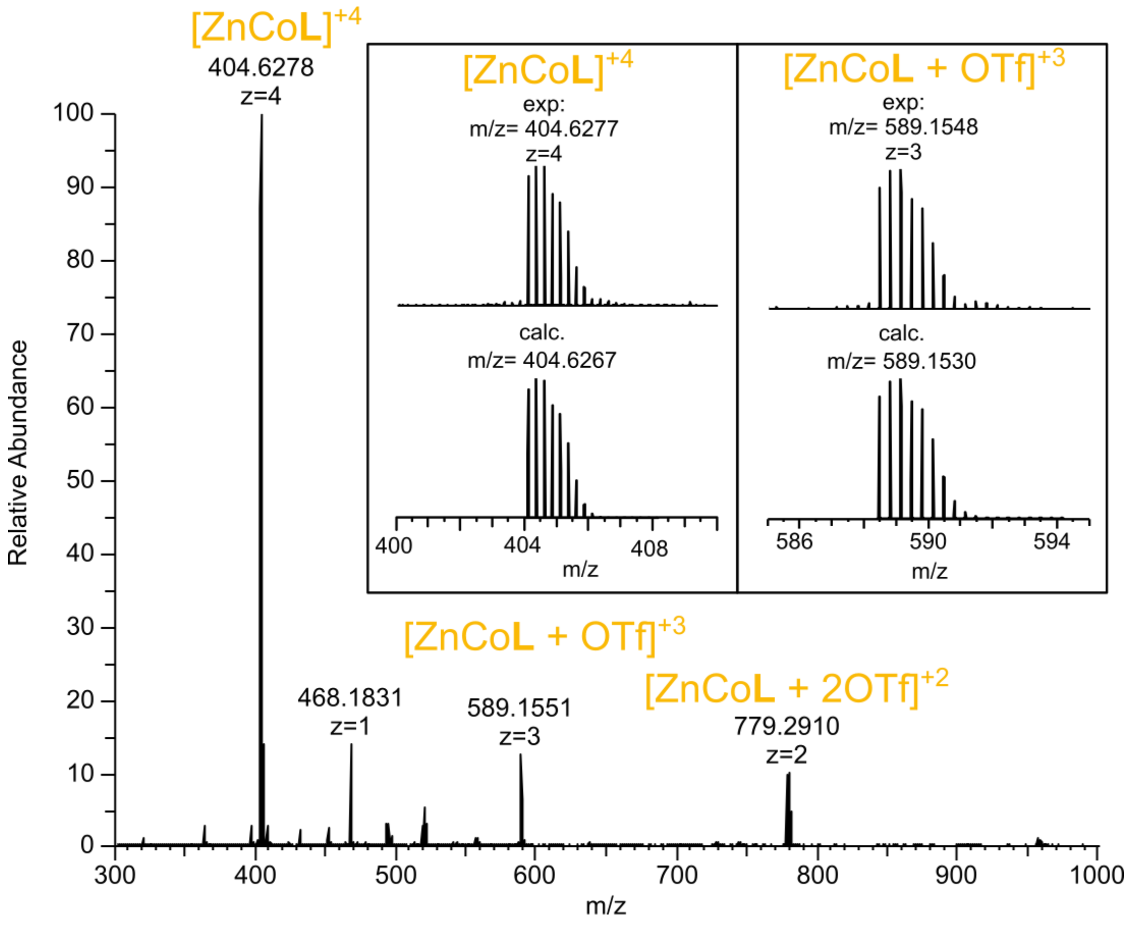

Figure S42. ESI^{+} mass spectrum (CH_3CN) of ZnCo**L**.

S4.1.3 CoFe**L**

CoFe**L** was synthesized according to the general procedure (Section S3.2) using $Co(OTf)_2$ (0.31 mg, 0.86 µmol, 1.00 equiv.), $Fe(OTf)_2$ (0.31 mg, 0.86 µmol, 1.00 equiv.), aldehyde **1** (1.22 mg, 2.60 µmol, 3.00 equiv.), and TREN (0.13 mg, 0.86 µmol, 1.00 equiv.) with a reaction time of 1 day at a temperature of 70 °C.

The product mixture contained minor amounts of Fe_2**L**, Co_2**L**, and FeCo**L**. For details see Section S4.3and Section S4.4.

$[C_{96}H_{75}N_{19}FeCo](CF_3O_3S)_4$

^{1}H NMR (500 MHz, CD_3CN) δ [ppm] = 10.14 (H-m), 48.75 (H-q), 113.74 (H-r), 171.75 (H-s), 183.48 (H-p).

Due to the strong paramagnetism of the compound not all assignments could be made and not all signals could be observed.

HRMS (ESI$^+$ Orbitrap): m/z (relative intensity) = 402.3817 (100%, [CoFe**L**]$^{+4}$, calcd. 402.3786), 586.1603 (35%, [CoFe**L** + OTf]$^{+3}$, calcd. 586.1558).

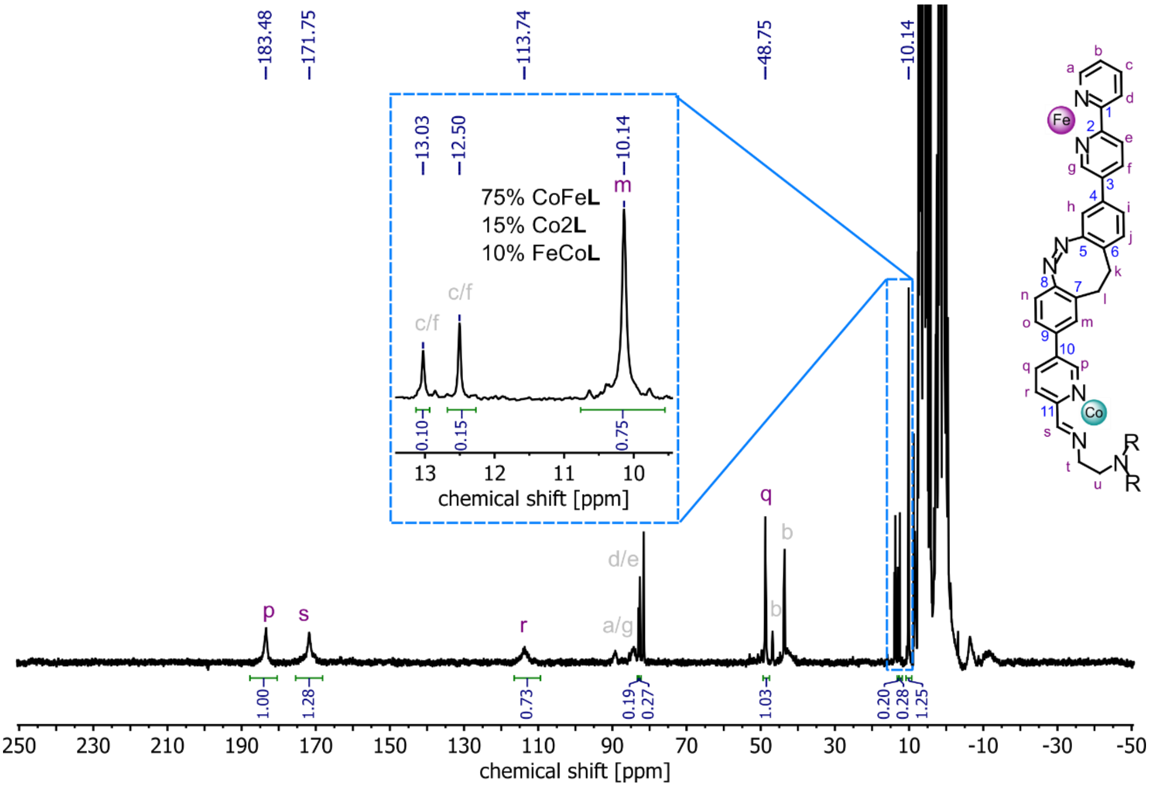

Figure S43. ^{1}H NMR spectrum (500 MHz, CD_3CN, 298 K) of CoFe**L**.

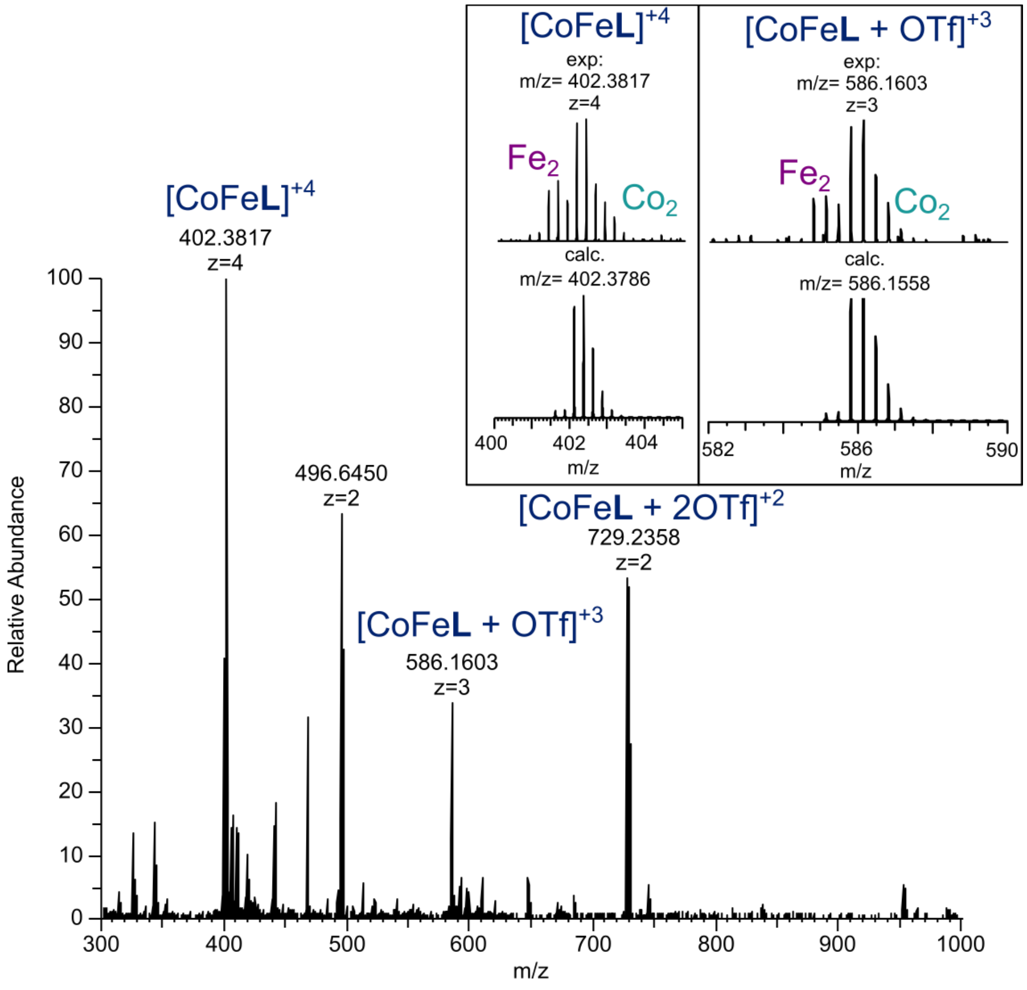

Figure S44. ESI^+ mass spectrum (CH_3CN) of CoFe**L**.

S4.2 Overview of one-pot self-sorting results

Table S3. Overview of the results of one-pot self-sorting experiments showing that the kinetically more labile metal ends up in the M^{TREN} binding site with the M^{bipy} binding site occupied by the metal forming stronger N−M ligand bonds.

	**Zn(II) + Fe(II)**	**Zn(II) + Co(II)**	**Co(II) + Fe(II)**
			(major product)
$\mathbf{M^{TREN}}$	Zn(II)	Zn(II)	Co(II)
	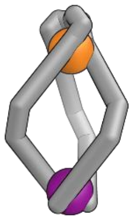	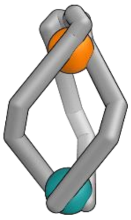	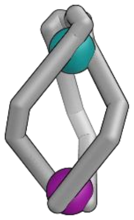
$\mathbf{M^{bipy}}$	Fe(II)	Co(II)	Fe(II)
			(major product)

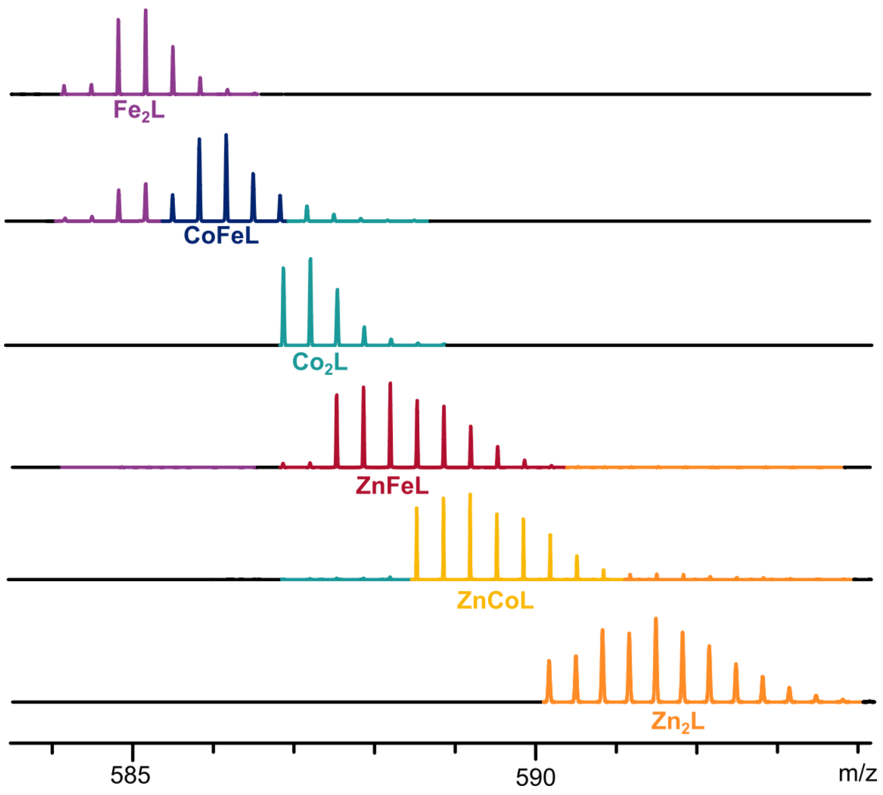

Figure S45. ESI$^+$ MS spectra (298 K, CH_3CN) of Fe_2**L**, CoFe**L**, Co_2**L**, ZnFe**L**, ZnCo**L**, and Zn_2**L**. The main product of the Co(II)/Fe(II) self-sorting experiment is the heterometallic CoFe**L** complex with the homometallic Co_2**L** and Fe_2**L** complexes being formed as minor side products. The Zn(II)/Co(II) and Zn(II)/Fe(II)self-sorting experiments selectively produced only the heterometallic ZnCo**L**/ZnFe**L** complexes.

S4.3 CoFe**L** – UV-vis

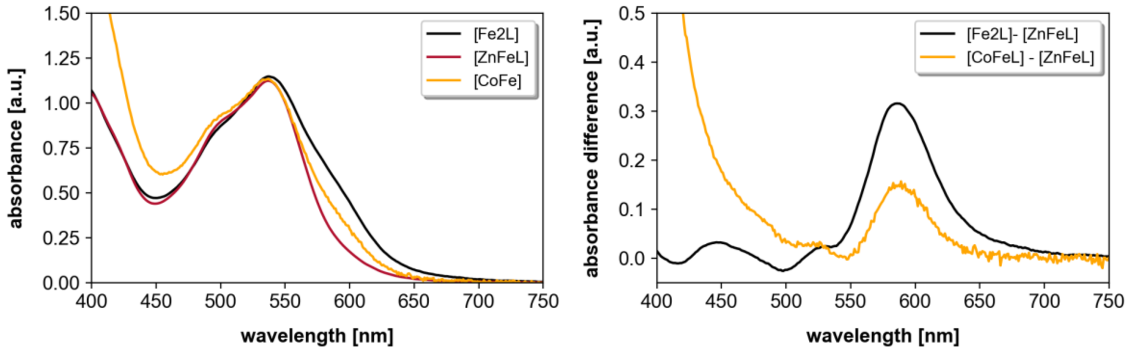

Figure S46. UV-vis spectra of Fe_2**L**, ZnFe**L** and CoFe**L** (CH_3CN, 0.06 mM) (*left*) and plot after subtracting the spectrum of ZnFe**L** from Fe_2**L** and CoFe**L** (*right*). Since Fe_2**L** contains Fe^{Bipy} and Fe^{TREN} in exactly the same amounts, the relative absorbances can be used as a benchmark for a 1:1 mixture of the two iron chromophores. Assuming the extinction coefficients of the Fe chromophores in the mixed complexes are similar to those in Fe_2**L**, it can be estimated that in the CoFe**L** mixture three quarters of all Fe(II) ions are in the bipy coordination site (Fe^{bipy}) with the remainder in the TREN coordination site (Fe^{TREN}), as the shoulder at 590 nm is about 1/3 as intense in CoFe**L** than it is counterpart in Fe_2**L**. This is just a rough estimate, indicating that CoFe**L** is the major product.

S4.4 ZnCoL and CoFeL – ^{1}H NMR

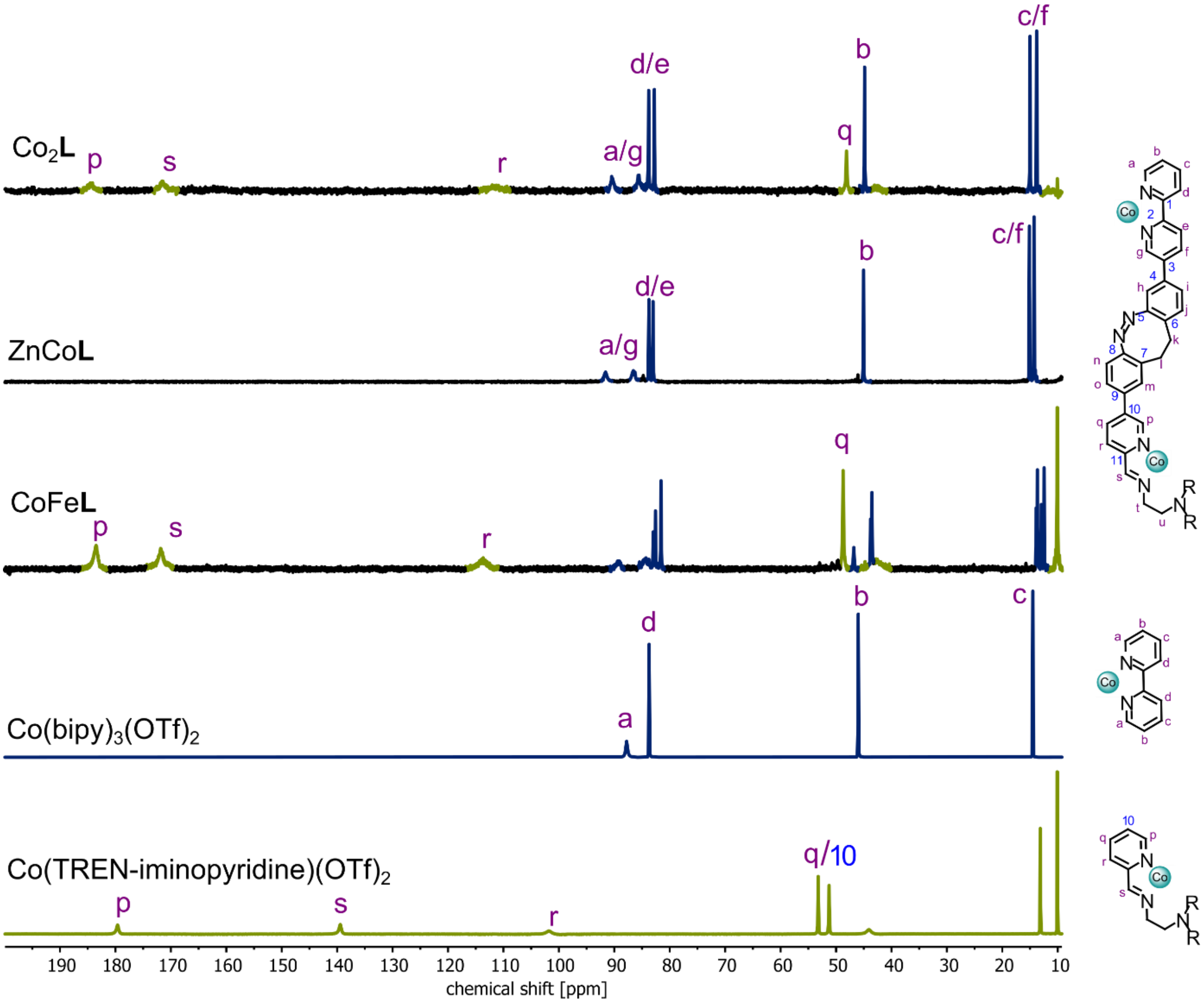

Figure S47. Wide-sweep ^{1}H NMR spectra (400 MHz, CD_3CN, 298 K) of Co_2**L**, ZnCo**L**, CoFe**L**, $Co(bipy)_3$, and Co^{TREN} (top to bottom). Signals referring to Co(II) ions in a bipy coordination environment (Co^{bipy}) are highlighted in blue, Co(II) ions in a TREN-pyridylimine corrdination evironment are highlighted in green (Co^{TREN}). For ZnCo**L**, no signals for Co^{TREN} can be observed, indicating successful self-sorting into $Zn^{TREN}Co^{bipy}$**L**. For CoFe**L**, the signal at δ = 48 ppm belonging to H-q in Co^{TREN} is much more intense than the neighbouring signal at δ = 45 ppm belonging to H-b Co^{bipy}. When comparing the relative intensities to the spectrum of Co_2**L**, this indicates Co^{TREN} having been formed preferentially over Co^{bipy} with an estimated ratio of around 3:1. This is in good agreement from the estimates made from the UV-vis data (Figure S46).

S5 UV-vis kinetics of the complexation reactions

The kinetics of complex formation were investigated using UV-vis spectroscopy, as the Fe(II) MLCT bands are readily observable. Initially, a stock solution of the ligand in acetonitrile was prepared at a concentration of either 0.4 or 0.2 mg/mL and added to a 1 mL screw-capped cuvette and positioned in the spectrometer at 25 °C.

Subsequently, a stock solution of TREN in methanol was added, using 3.5 or 7 µL of the solution. Following this, a stock solution of the metal salt(s) in acetonitrile was added to the mixture. The cuvette was then securely capped and thoroughly shaken to ensure complete homogenization of the solution before being returned to the spectrometer.

The sample was initially maintained at a constant temperature of 25 °C for 20 minutes. Subsequently, the temperature was raised to 65 °C to simulate the conditions used during helicate synthesis. Throughout the experiment, the progression of the reaction was monitored by measuring absorbance spectra at regular intervals. The data presented displays one spectrum captured every 30 minutes over a total duration of 5 hours.

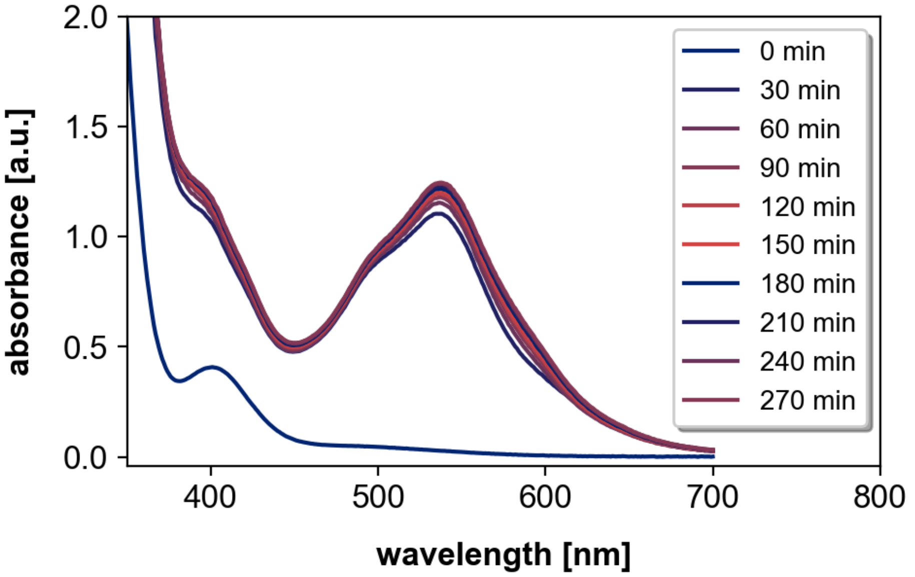

Figure S48. Fe_2**L** formation (0.4 mg/mL aldehyde **1**, 1.0 equiv. TREN, 2.0 equiv. $Fe(OTf)_2$) as followed by UV-vis at 25 °C to 65 °C with one scan every 30 minutes. Very fast formation of a band at 540 nm corresponding to Fe^{bipy} and slow formation of a second band at 590 nm, corresponding to Fe^{TREN}, were observed.

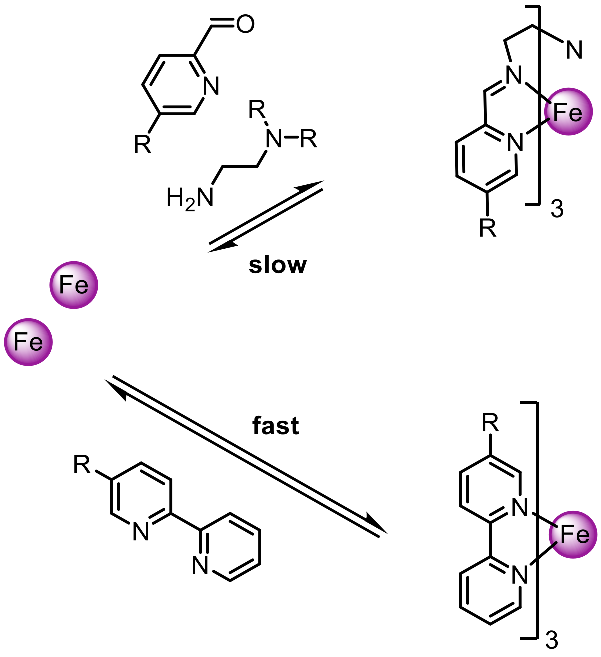

Figure S49. Mechanistic representation of the formation of Fe_2**L** based on the UV-vis experiment (Figure S48). The formation of Fe^{bipy} is rapid and therefore complete within the first 30 minutes, whereas Fe^{TREN} forms much slower over the course of multiple hours.

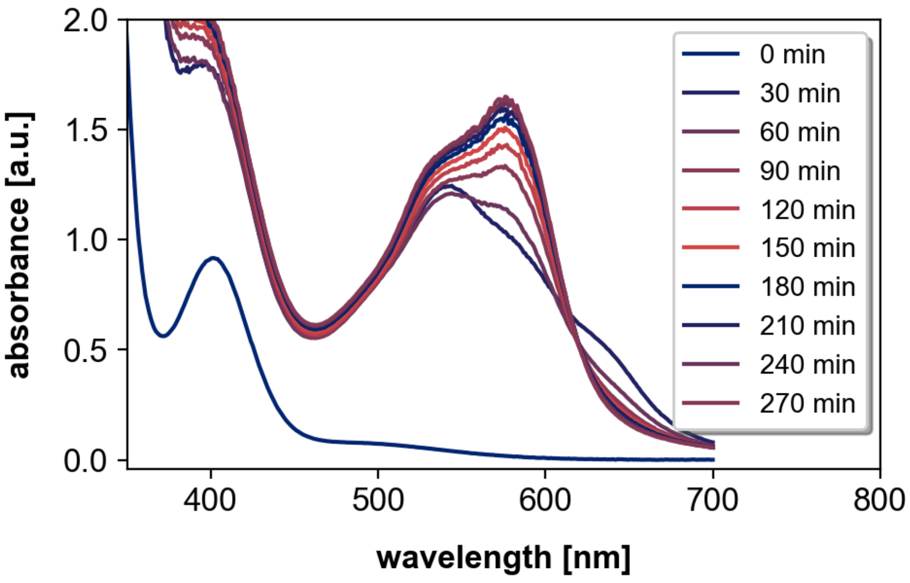

Figure S50. Fe**L** formation (0.2 mg/mL aldehyde **1**, 1.0 equiv. TREN, 1.0 equiv. $Fe(OTf)_2$) as followed by UV-vis at 25 °C to 65 °C with one scan every 30 minutes. Slow formation of a band at 590 nm (Fe^{TREN}), with transient absorbance bands visible at 540 nm (Fe^{bipy}) and 620 nm being observed.

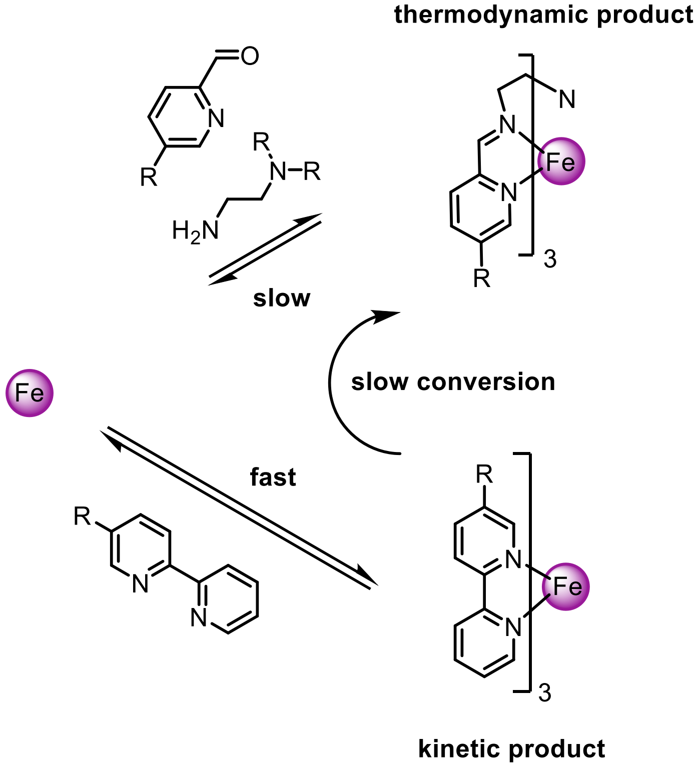

Figure S51. Mechanistic representation of the formation of Fe**L** based on the UV-vis experiment (Figure S50). The formation of Fe^{bipy} is fast, but over time the iminopyridine binding site is formed and the iron atom migrates to the Fe^{TREN} site, indicating that Fe^{Bipy} is a kinetic product, but Fe^{TREN} is the thermodynamic product.

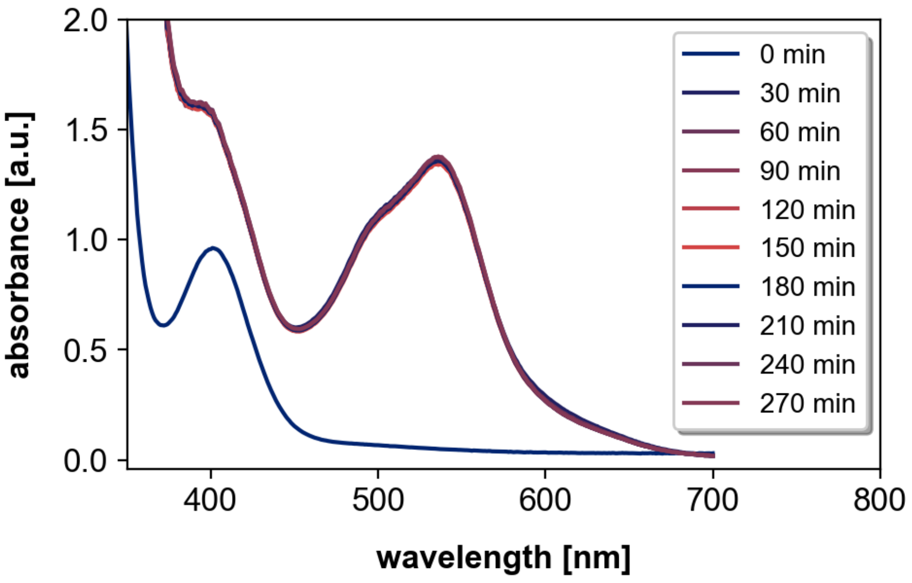

Figure S52. ZnFe**L** formation (0.4 mg/mL aldehyde **1**, 1.0 equiv. TREN, 1.0 equiv. $Fe(OTf)_2$, 1.0 equiv. $Zn(OTf)_2$) as followed by UV-vis at 25 °C to 65 °C with one scan every 30 minutes. Very fast formation of a band at 540 nm corresponding to Fe^{bipy} and no absorbance band at 590 nm (Fe^{TREN}) were observed.

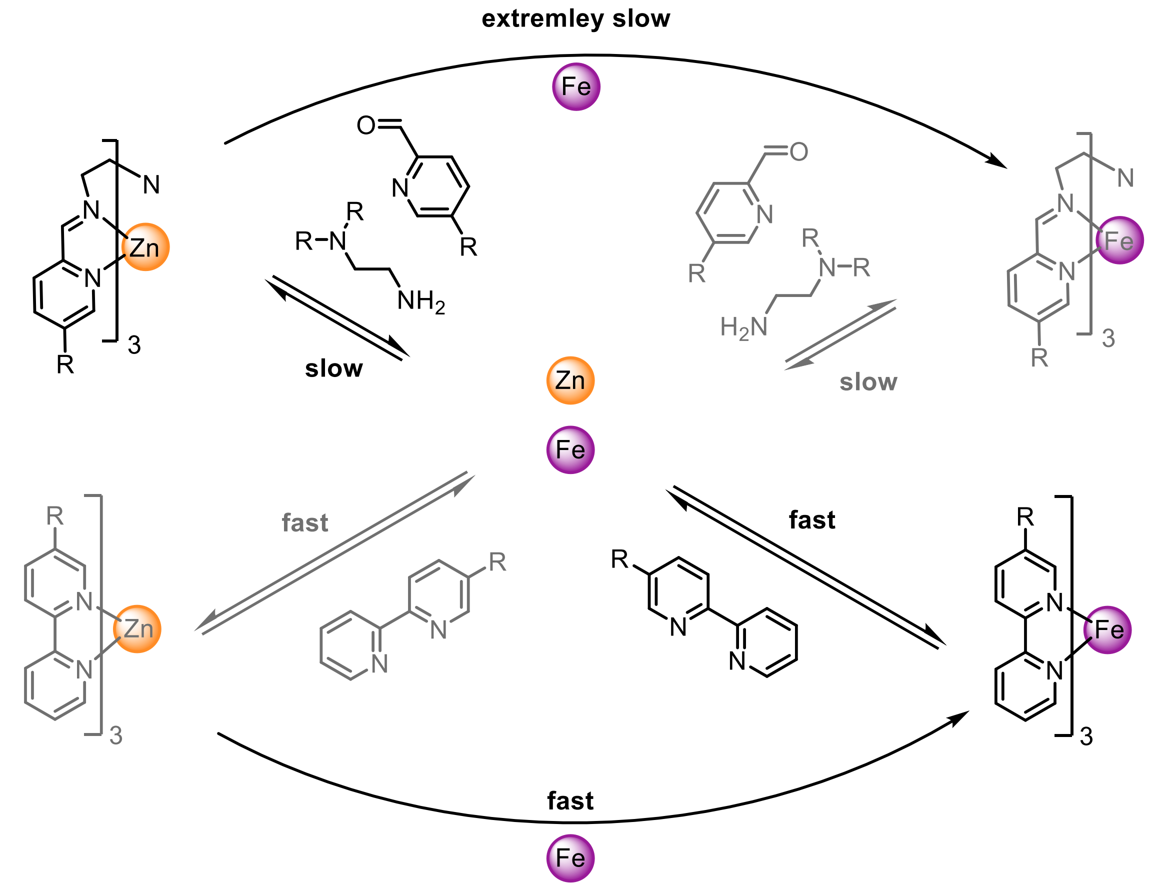

Figure S53. Mechanistic representation of the formation of ZnFe**L** based on the UV-vis experiment (Figure S52) The formation of the bipyridine binding site is fast. The stronger binding affinity of Fe(II) over Zn(II) results in the exclusive formation of Fe^{bipy} over Zn^{bipy}, leaving the uncoordinated and therefore more lewis acidic Zn(II) ions to template and catalyse the formation of the iminopyridine binding site, forming Zn^{TREN}. Replacing the Zn(II) ions in the Zn^{TREN} site with Fe(II) ions to form Fe^{TREN} is a thermodynamically downhill, but extremely slow process (5% over two months, see Section S9.4 for details).

S6 Structural characterization of the complexes

S6.1 A note on the helicate nomenclature used

The helicates are composed of five chiral building blocks: three ligand arms (Figure S54) and two octahedral metal coordination sites, resulting in a multitude of possible stereoisomers.

This multitude of potential stereoisomers raises the question of finding a clear, straightforward way to name all these stereoisomers. Since the aim of this work is not to establish the perfect nomenclature that encompasses all aspects of chirality but rather to visualise and explain the observed stereoisomers in an accessible manner for the reader, we have defined the following specific nomenclature for the helicates (Figure S55):

(i) The direction of view in the helicates is from the M^{TREN} to the M^{bipy} metal centre.
(ii) The helical chirality of the metal centres is defined as Δ and Λ for right- and left-handed helicity, respectively, when looking along this axis.
(iii) The directionality in the ligand-arm orientation is defined by the diazocine units, looking from the ethylene bridge to the azo bridge. The diazocine moieties can either point with their azo nitrogens clockwise or anticlockwise when looking along the M^{TREN}–M^{bipy} axis, which is denoted as P and M, respectively.

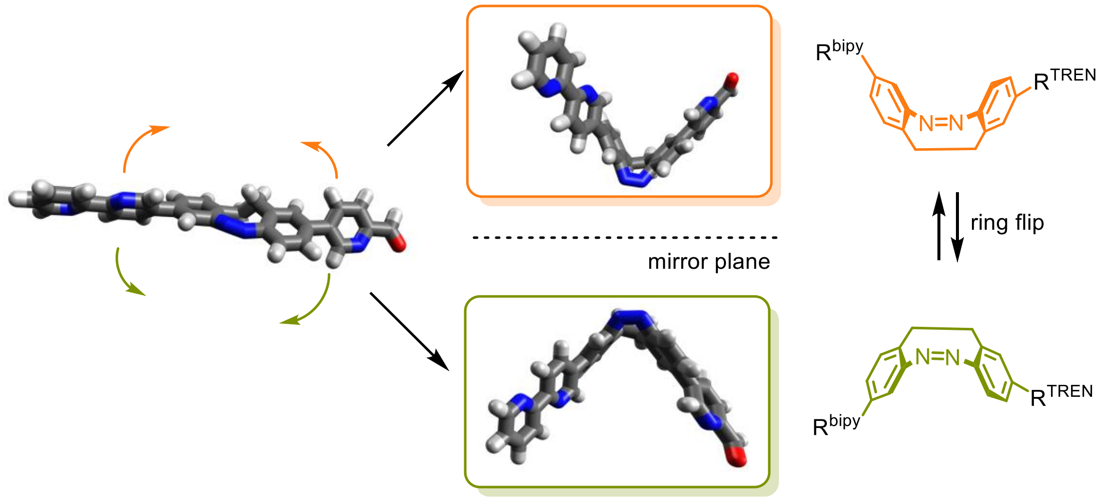

Figure S54. Chirality in the *Z* ground state of aldehyde **1**.

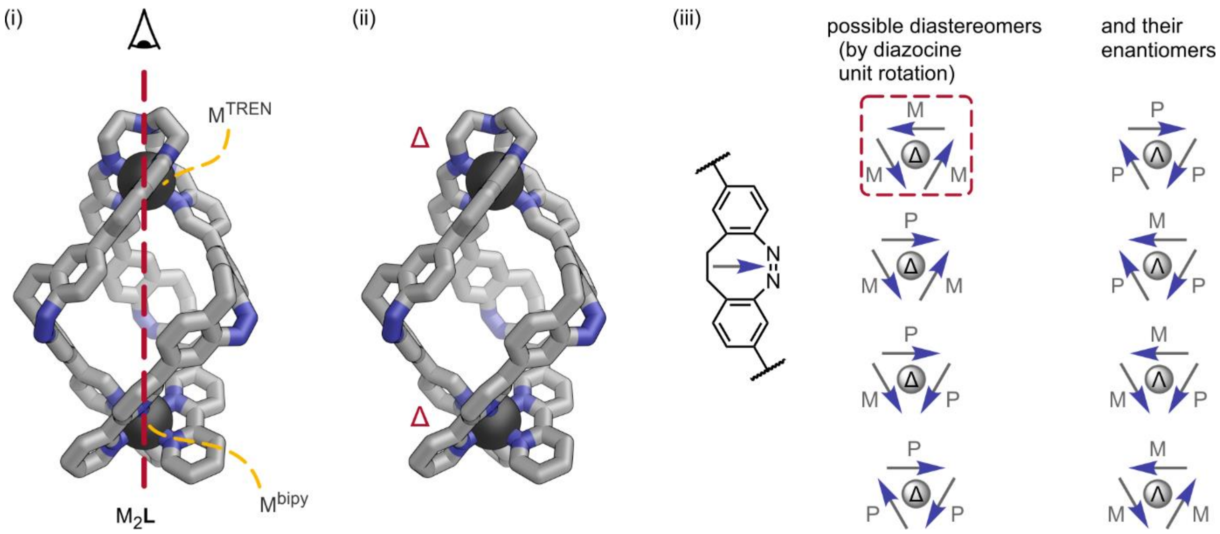

Figure S55. How to derive the nomenclature used in this work, following three steps: (i) The direction of view in the helicates is from the MTREN to the Mbipy metal centre. (ii) The helical chirality of the metal centres is defined as Δ and Λ for right- and left-handed helicity, respectively, when looking along this axis. (iii) The directionality in the ligand-arm orientation is defined by the diazocine units, looking from the ethylene bridge to the azo bridge. The diazocine moieties can either point with their azo nitrogens clockwise or anticlockwise when looking along the MTREN−Mbipy axis, which is denoted as P and M, respectively. Right: Possible diastereomers derived from different diazocine unit orientations within the helicates and their enantiomers. Cartoon representing the structure on the left is highlighted in red.

With two chiral metal centres and three chiral ligand arms, eight diastereomers (plus eight enantiomers) are possible:

(i) The two metal centres can be homochiral, resulting in a helicate structure, or heterochiral, resulting in a pseudo-mesocate.
(ii) The three ligand arms could exist with four different diazocine orientations: (MMM), (PMM), (PPM), and (PPP; Figure S55, right).

Thus, both the helicate and the pseudo-mesocate can have four distinct ligand-arm orientations, leading to eight possible diastereomers. Each of these exists as a racemic mixture of two enantiomers.

S6.2 X-ray crystallography

Suitable single crystals of Fe_2**L** and ZnFe**L** were grown by vapour diffusion. An acetonitrile solution of the respective helicate (approx. 2 mM, 0.3 mL) was filtered and added to a 2.0 mL GC vial. The GC vial was left open and placed in a 10 mL glass vial containing approx. 2 mL of 1,2-dimethoxyethane as the anti-solvent. The outer vial was closed with a snap lid and stored in a dark fridge at approx. 6 °C for five weeks.

The data collections were performed on a STOE Stadivari Eulerian 4-circle diffractometer using Cu-K_α radiation (λ = 1.54186 Å). The diffractometer was equipped with a low–temperature device (Cryostream 800er series, Oxford Cryosystems, 100(1) K). Intensities were measured by fine-slicing ω-scans and corrected for background, polarization and Lorentz effects. An absorption correction by scaling of reflection intensities with a subsequent spherical absorption correction was performed with STOE LANA programme.[5]

The structures were solved by intrinsic phasing methods[6] and refined anisotropically by the least-squares procedure implemented in the SHELX programme system.[7]

The hydrogen atoms were included isotropically using a riding model on the bound carbon atoms.

Some of the disordered triflate anions as well as some of the heavily disordered co-crystallised solvent molecules had to be modelled by using PLATON's squeeze methodology in order to account for their contributions to the calculated structure factors.[8]

Even if the final R-values of the structural model are not of outstanding quality, we consider the refined structural models of both samples to be valid and worth publishing. Due to the very high number of atoms in the asymmetric unit (high F(000) value) combined with small crystal size and poor scattering properties, ωR_2 values of about 40-45% are in our opinion not in the good, but still in the acceptable range.

CCDC numbers 2442413 (**Fe_2L**) and 2442414 (**ZnFeL**) contain the supplementary crystallographic data for this paper, which can be obtained free of charge from the Cambridge Crystallographic Data Centre via http://www.ccdc.cam.ac.uk/data_request/cif.

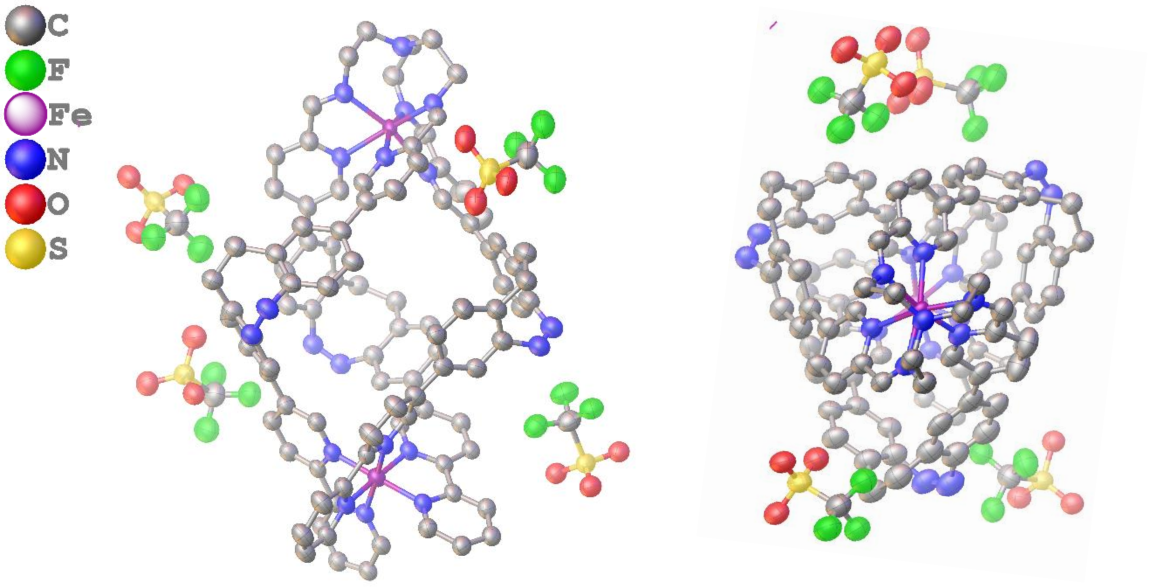

Figure S56. Asymmetric unit of helicate Fe_2**L** as observed in the crystal structure, plotted from two different viewing angles. Hydrogen atoms omitted for clarity. Displacement ellipsoids are drawn at 50% probability.

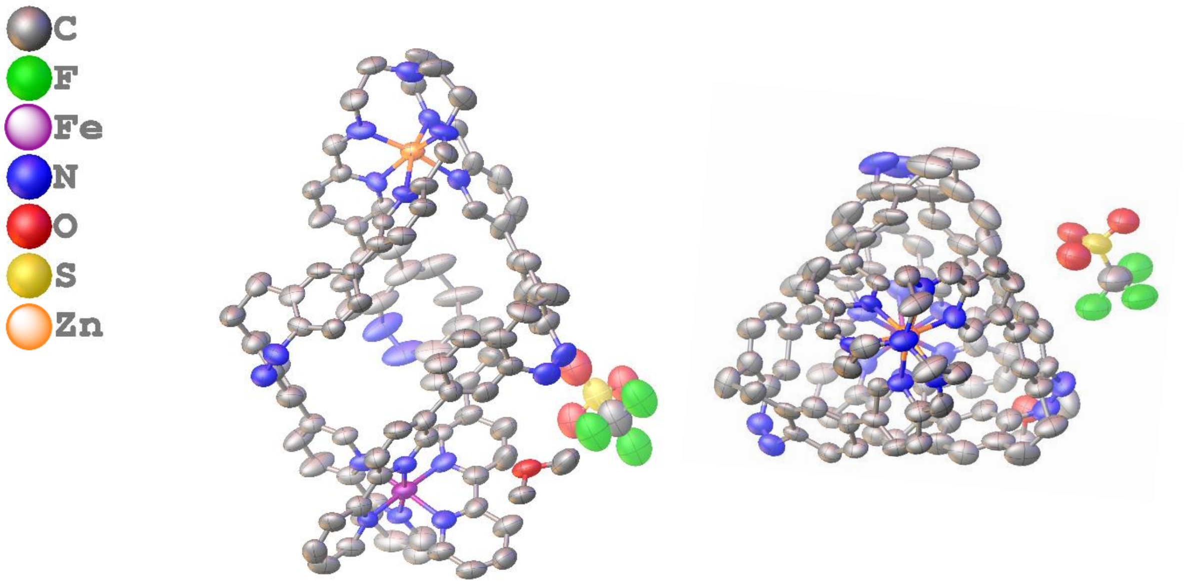

Figure S57. Asymmetric unit of helicate ZnFe**L** as observed in the crystal structure, plotted from two different viewing angles. Hydrogen atoms omitted for clarity. Displacement ellipsoids are drawn at 50% probability.

Table S4. Crystal data and refinement parameters for helicate Fe_2**L**.

Crystal Habitus	clear red plate
Device Type	STOE STADIVARI
Empirical formula	$C_{100}H_{75}F_{12}Fe_2N_{19}O_{12}S_4$
Moiety formula	$C_{96}H_{75}Fe_2N_{19}$, 4(CF_3O_3S)
Formula weight	2202.73
Temperature/K	100
Crystal system	triclinic
Space group	P-1
a/Å	13.868(6)
b/Å	13.993(8)
c/Å	27.609(12)
α/°	84.79(4)
β/°	88.59(4)
γ/°	75.32(4)
Volume/Å^3	5162(4)
Z	2
ρ_{calc}g/cm^3	1.417
μ/mm^{-1}	3.791
F(000)	2256.0
Crystal size/mm^3	0.1 × 0.1 × 0.03
Absorption correction	multi-scan
T_{min}; T_{max}	0.3511; 0.8080
Radiation	Cu Kα (λ = 1.54186)
2Θ range for data collection/°	9.218 to 146.854°
Completeness to theta	0.993
Index ranges	-16 ≤ h ≤ 17, -17 ≤ k ≤ 16, -13 ≤ l ≤ 32
Reflections collected	98477
Independent reflections	19469 [R_{int} = 0.3935, R_{sigma} = 0.2458]
Data/restraints/parameters	19469/1319/1342
Goodness-of-fit on F^2	1.019
Final R indexes [I>=2σ (I)]	R_1 = 0.1411, wR_2 = 0.3055
Final R indexes [all data]	R_1 = 0.3092, wR_2 = 0.4099
Largest diff. peak/hole / e Å^{-3}	1.11/-0.56
Crystal Habitus	clear red plate
Device Type	STOE STADIVARI
Empirical formula	$C_{100}H_{75}F_{12}Fe_2N_{19}O_{12}S_4$
Moiety formula	$C_{96}H_{75}Fe_2N_{19}$, 4(CF_3O_3S)

Table S5. Crystal data and refinement parameters for helicate ZnFe**L**.

Crystal Habitus	clear dark red block
Device Type	STOE STADIVARI
Empirical formula	$C_{102}H_{80}F_{12}FeN_{19}O_{13}S_4Zn$
Moiety formula	$C_{96}H_{75}FeN_{19}Zn$, CF_3O_3S, $0.5(C_4H_{10}O_2)$
Formula weight	1810.13
Temperature/K	100.15
Crystal system	triclinic
Space group	P-1
a/Å	13.2445(6)
b/Å	13.7646(8)
c/Å	29.9758(16)
α/°	87.839(5)
β/°	83.823(4)
γ/°	83.708(4)
Volume/Å^3	5398.4(5)
Z	2
ρ_{calc}g/cm^3	1.114
μ/mm^{-1}	2.008
F(000)	2314.0
Crystal size/mm^3	0.35 × 0.2 × 0.14
Absorption correction	multi-scan
T_{min}; T_{max}	0.2195; 0.8261
Radiation	Cu Kα (λ = 1.54186)
2Θ range for data collection/°	8.892 to 135.492°
Completeness to theta	0.990
Index ranges	-15 ≤ h ≤ 15, -7 ≤ k ≤ 16, -34 ≤ l ≤ 35
Reflections collected	100689
Independent reflections	19366 [R_{int} = 0.1942, R_{sigma} = 0.1081]
Data/restraints/parameters	19366/65/1154
Goodness-of-fit on F^2	1.403
Final R indexes [I>=2σ (I)]	R_1 = 0.1658, wR_2 = 0.4095
Final R indexes [all data]	R_1 = 0.2397, wR_2 = 0.4637
Largest diff. peak/hole / e Å^{-3}	1.36/-0.93
Crystal Habitus	clear dark red block
Device Type	STOE STADIVARI
Empirical formula	$C_{102}H_{80}F_{12}FeN_{19}O_{13}S_4Zn$
Moiety formula	$C_{96}H_{75}FeN_{19}Zn$, CF_3O_3S, $0.5(C_4H_{10}O_2)$

S6.3 Quantum chemical structure optimizations

S6.3.1 Structures of $Zn_2\mathbf{L}$ with unidirectional diazocine orientation within the ligand arms in the *Z* state

Input structures for four diastereomers with the two possible unidirectional diazocine orientations within the ligand arms (PPP and MMM, i.e. $Zn^{\Delta}Zn^{\Delta}\mathbf{L}^{MMM}$, $Zn^{\Delta}Zn^{\Delta}\mathbf{L}^{PPP}$, $Zn^{\Lambda}Zn^{\Delta}\mathbf{L}^{PPP}$, $Zn^{\Lambda}Zn^{\Delta}\mathbf{L}^{MMM}$) were generated using the stk python library (Version 2024.9.23.1),[9–11] which enabled the rapid creation of the initial molecular geometries. These structures were then preoptimized in Avogadro[12] (Version 1.2.0) using the Universal Force Field (UFF).[13] Following this, Grimme's CREST[14] algorithm at the GFN-FF[15] level was employed to perform a comprehensive exploration of the potential energy surface. The best structure identified from the conformer search was subsequently reoptimized on GFN2-xTB[16] level using the ALPB[17] model for implicit solvation in acetonitrile. The final structures were visualized using PyMol[18] (Version 3.1.0a OpenSource, Table S6).

Table S6. Overview GFN2-xTB optimized structures of the four diastereomers of $Zn_2\mathbf{L}$ containing only unidirectional diazocine orientation of the *Z*-ligand arms. The filenames for the structures are listed below with the files available as supporting information.

$Zn^{\Delta}Zn^{\Delta}\mathbf{L}^{MMM}$	$Zn^{\Delta}Zn^{\Delta}\mathbf{L}^{PPP}$	$Zn^{\Lambda}Zn^{\Delta}\mathbf{L}^{PPP}$	$Zn^{\Lambda}Zn^{\Delta}\mathbf{L}^{MMM}$
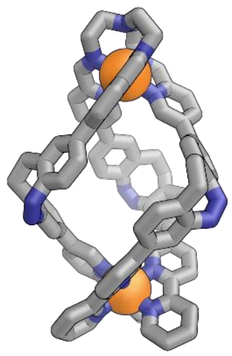	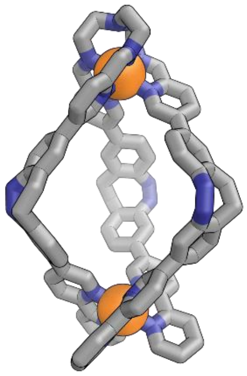	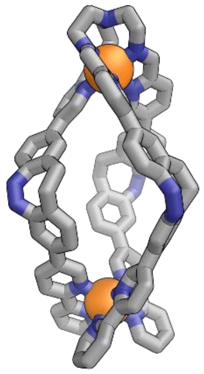	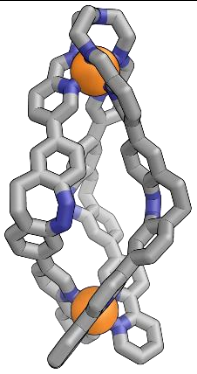
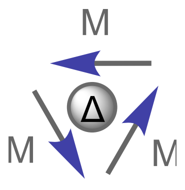	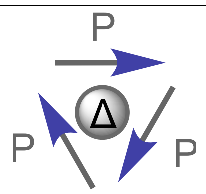	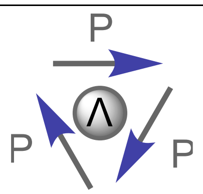	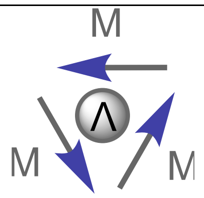
Zn2-Z-Helicate-1.xyz	Zn2-Z-Helicate-4.xyz	Zn2-Z-Mesocate-1.xyz	Zn2-Z-Mesocate-4.xyz

S6.3.2 Structures of Zn_2**L** with other ligand configurations

Input structures for structures containing more than one orientation of the Z-ligand arms or *E*-diazocines were generated using Avogadro[12] (Version 1.2.0) by manipulating the homochiral *Z*-diazocine Zn_2**L** structures listed in Table S6 and subsequent reoptimized on GFN2xTB[16] level using the ALPB[17] model for implicit solvation in acetonitrile. This gave rise to the mixed ligand chirality structures (Table S7) and the four all-E-Structures (Table S8). When an enantiomer of a structure was needed, the structure was mirrored using the "invert chirality" function built into Avogadro. The final structures were visualized using PyMol[18] (Version 3.1.0a OpenSource).

Table S7. Overview of GFN2-xTB optimized structures of the four diastereomers (atropisomers) for the $Zn^{\Delta}Zn^{\Delta}$ helicate. The filenames for the structures are listed below with the files available as supporting information.

$Zn^{\Delta}Zn^{\Delta}$**L**^{MMM}	$Zn^{\Delta}Zn^{\Delta}$**L**^{PMM}	$Zn^{\Delta}Zn^{\Delta}$**L**^{PPM}	$Zn^{\Delta}Zn^{\Delta}$**L**^{PPP}
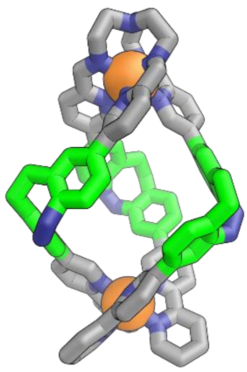	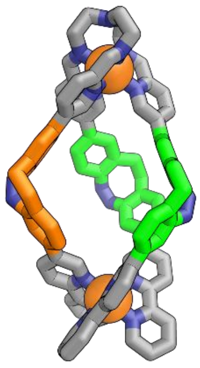	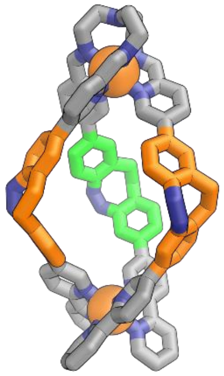	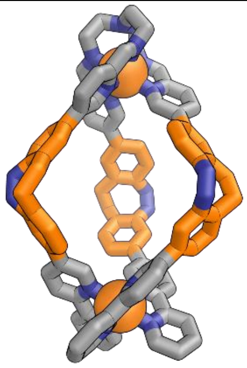
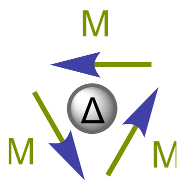	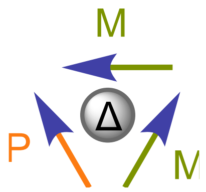	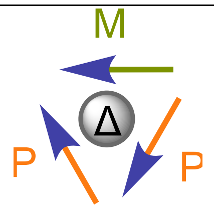	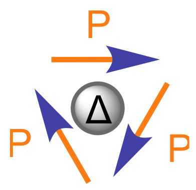
Zn2-Z-Helicate-1.xyz	Zn2-Z-Helicate-2.xyz	Zn2-Z-Helicate-3.xyz	Zn2-Z-Helicate-4.xyz

Table S8. Overview of GFN2-xTB optimized structures of six diastereomers of *E*-Zn_2**L**. The filenames for the structures are listed below with the files available as supporting information.

$Zn^{\Delta}Zn^{\Delta}\mathbf{L}^{PPP}$	$Zn^{\Delta}Zn^{\Delta}\mathbf{L}^{PPM}$	$Zn^{\Delta}Zn^{\Delta}\mathbf{L}^{PMM}$	$Zn^{\Delta}Zn^{\Delta}\mathbf{L}^{MMM}$	$Zn^{\Lambda}Zn^{\Delta}\mathbf{L}^{PPP}$	$Zn^{\Lambda}Zn^{\Delta}\mathbf{L}^{MMM}$
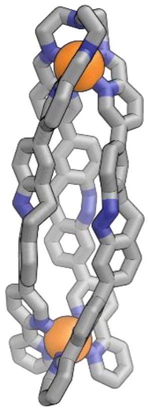	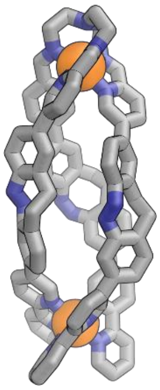	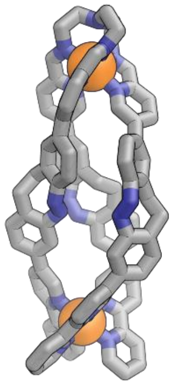	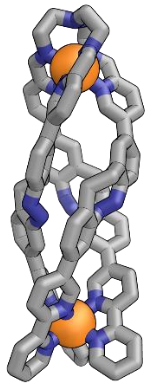	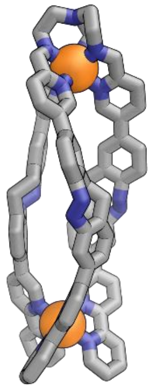	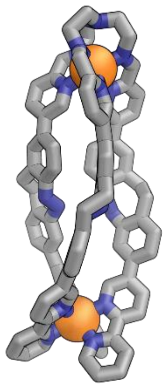
Zn2-E-Helicate-1.xyz	Zn2-E-Helicate-2.xyz	Zn2-E-Helicate-3.xyz	Zn2-E-Helicate-4.xyz	Zn2-E-Mesocate-1.xyz	Zn2-E-Mesocate-4.xyz

S6.3.3 Structures of complexes with metals other than zinc

Structures with metals other than zinc were generated by replacing the zinc atom with the respective element and reoptimized on GFN2-xTB[16] level using the ALPB[17] model for implicit solvation in acetonitrile. Complexes that contained iron atoms did not converge when using ALPB solvation and were optimized without implicit solvation. No significant structural changes were observed for iron and cobalt derivatives. The final structures were visualized using PyMol[18] (Version 3.1.0a OpenSource). Some examples are listed in (Table S9).

Table S9. Overview of GFN2-xTB optimized structures of four example diastereomers of ZnFe**L**. The filenames for the structures are listed below with the files for these and other diastereomers available as supporting information.

$Zn^{\Delta}Fe^{\Delta}\mathbf{L}^{MMM}$	$Zn^{\Delta}Fe^{\Delta}\mathbf{L}^{PPP}$	$Zn^{\Lambda}Fe^{\Delta}\mathbf{L}^{PPP}$	$Zn^{\Lambda}Fe^{\Delta}\mathbf{L}^{MMM}$
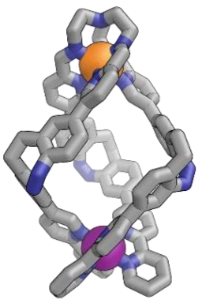	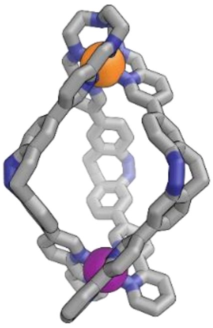	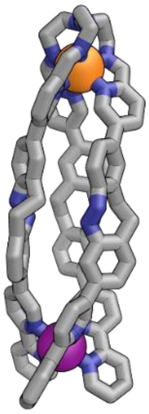	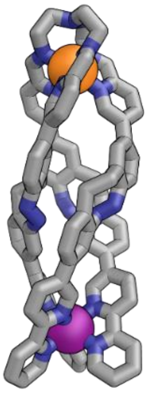
ZnFe-Z-Helicate-1.xyz	ZnFe-Z-Helicate-4.xyz	ZnFe-E-Helicate-1.xyz	ZnFe-E-Helicate-4.xyz

S6.3.4 Comment on energy calculations on metal-organic helicates

All structures were optimized using Grimme's GFN2-xTB[16] as this semi-empirical method shows a great cost-to-benefit ratio for large metallo-supramolecular systems. It is optimized to yield reasonable structures, vibrational frequencies, and noncovalent interactions, but higher-level DFT methods are necessary for accurate energies. Despite resulting in generally more accurate energies, energy calculations of large, porous, flexible, and charged molecules using high-level DFT methods will not necessarily result in accurate energies. Approximating these as static structures in single-molecule models without explicit solvation and anions will likely result in inaccurate results.[19] The complexity and high computational cost of these advanced calculations lead us not to pursue the calculation of accurate relative energies of the structures.

S6.4 Determination of solution stereochemistry of the helicates

As mentioned above, the helicates and pseudo-mesocates are composed of five chiral building blocks, three ligand arms and two octahedral metal coordination sites, resulting in a multitude of possible stereoisomers.

With two chiral metal centres and three possible ligand-arm orientations, eight diastereomers (plus eight enantiomers) are possible. Firstly, the two metal centres can be homochiral, leading to helicates, or heterochiral, resulting in pseudo-mesocates. Secondly, the three ligand arms could exist in four diazocine orientations: (MMM), (PMM), (PPM), and (PPP). Out of these, only the two homochiral arrangements (MMM) and (PPP) would lead to a single set of signals in the NMR spectrum (Table S10).

This leaves four possible structures (Table S11). Three of these structures can be ruled out by the findings from ^{1}H ROESY NMR experiments (Section S6.4.1) as the observed H−H through-space couplings are only possible in the $Zn^{\Delta}Fe^{\Delta}\mathbf{L}^{MMM}$ structure (and its enantiomer). This structure is the same as the one observed in the solid state, as confirmed by the single crystal X-ray structure (Section S6.2). This arrangement of the ligand arms could be confirmed for both $Zn_2\mathbf{L}$ (Figure S63) and $Fe_2\mathbf{L}$ (Figure S62).

Table S10. Computational structures for the four possible ligand configurations in $Zn^{\Delta}Fe^{\Delta}\mathbf{L}$ (for computational details see Section S6.3). Since just one set of signals is observed by NMR, structures containing heterochiral ligands can be ruled out for the ground state of the complexes.

$Zn^{\Delta}Zn^{\Delta}\mathbf{L}^{MMM}$	$Zn^{\Delta}Zn^{\Delta}\mathbf{L}^{PMM}$	$Zn^{\Delta}Zn^{\Delta}\mathbf{L}^{PPM}$	$Zn^{\Delta}Zn^{\Delta}\mathbf{L}^{PPP}$
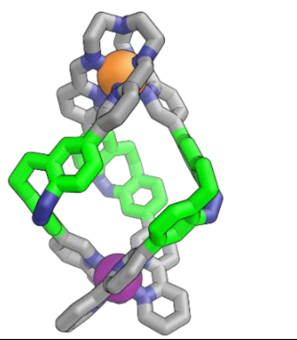	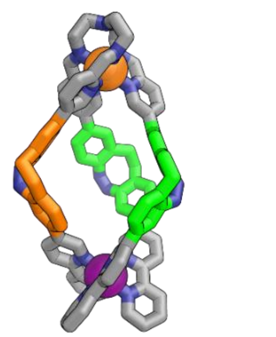	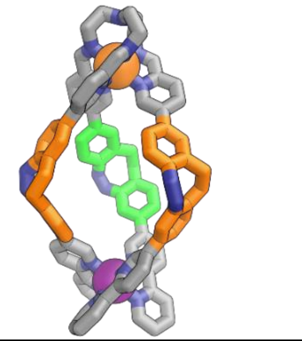	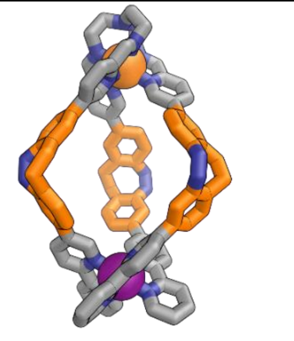
1 set of signals in NMR	3 sets of diastereotopic signals in NMR	3 sets of diastereotopic signals in NMR	1 set of signals in NMR

Table S11. Computational structures for the four remaining diastereomers of ZnFe**L** after ruling out mixed diazocine orientations within the ligand arms (Table S10) and ChemDraw representations of the ligand folding (for computational details see Section S6.3). Only $Zn^{\Delta}Fe^{\Delta}\mathbf{L}^{MMM}$ (and its enantiomer) show the correct folding that could give rise to the ^{1}H ROESY cross-peaks observed in Section S6.4. The same diastereomer was observed in the solid state (Section S6.2).

$Zn^{\Delta}Fe^{\Delta}\mathbf{L}^{MMM}$	$Zn^{\Delta}Fe^{\Delta}\mathbf{L}^{PPP}$	$Zn^{\Delta}Fe^{\Lambda}\mathbf{L}^{MMM}$	$Zn^{\Delta}Fe^{\Lambda}\mathbf{L}^{PPP}$
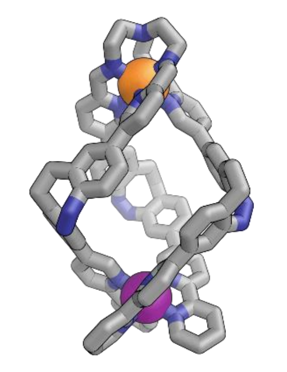 (+ enantiomer)	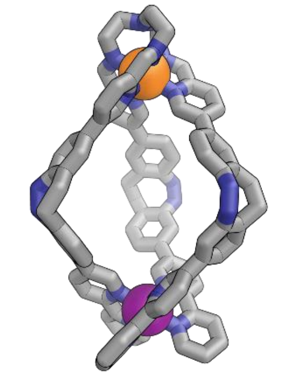 (+ enantiomer)	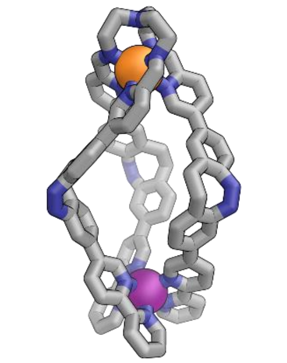 (+ enantiomer)	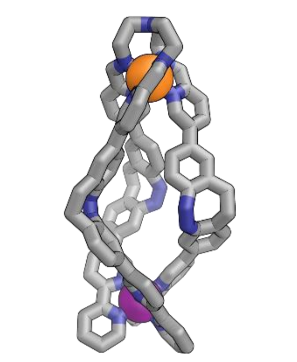 (+ enantiomer)
Ligand conformation in computational structure:			
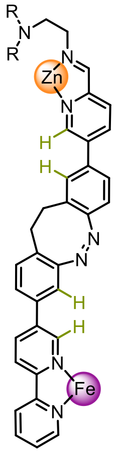	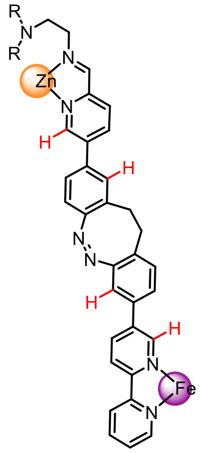	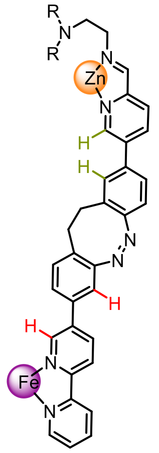	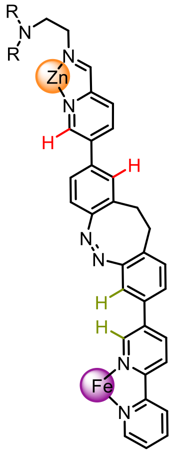
H-p and H-m are adjacent H-g and H-h are adjacent	H-p and H-m not adjacent H-g and H-h not adjacent	H-p and H-m are adjacent H-g and H-h not adjacent	H-p and H-m not adjacent H-g and H-h are adjacent

S6.4.1 1D ROESY NMR of ZnFeL

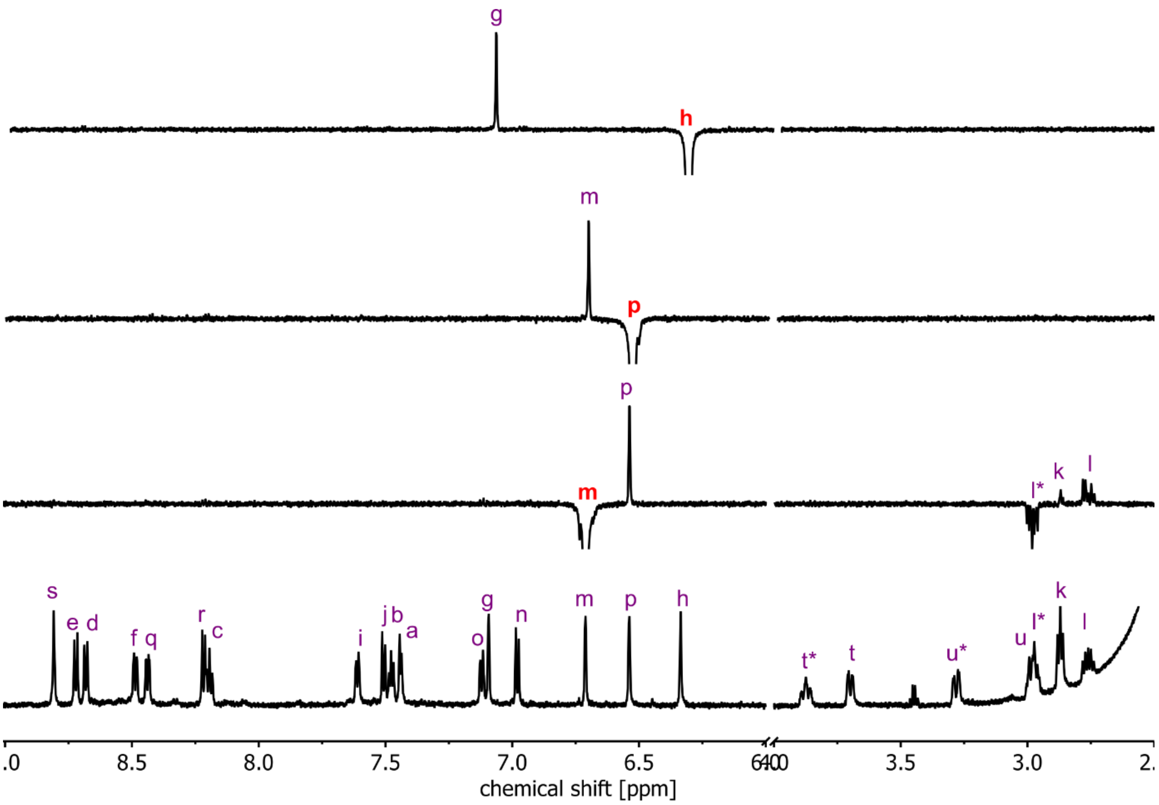

Figure S58. One dimensional ^{1}H ROESY spectra (700 MHz, CD_3CN, 1 mM, 298 K) of ZnFe**L** for signals H-h, H-p and H-m with results summarized in Figure S60 and Table S12.

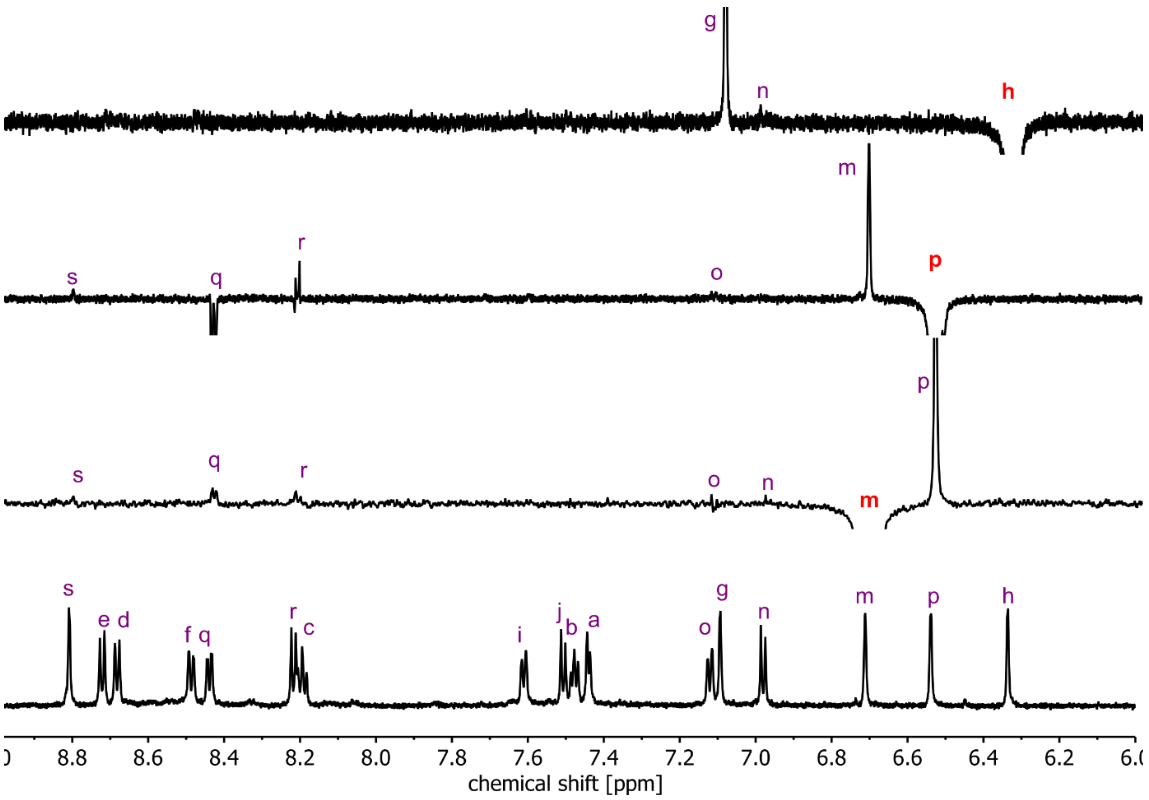

Figure S59. One dimensional ^{1}H ROESY spectra (700 MHz, CD_3CN, 1 mM, 298 K) of ZnFe**L** for signals H-h, H-p and H-m, measured with a small signal width (SW = 3 ppm) to observe just the aromatic region with results summarized in Figure S60 and Table S12.

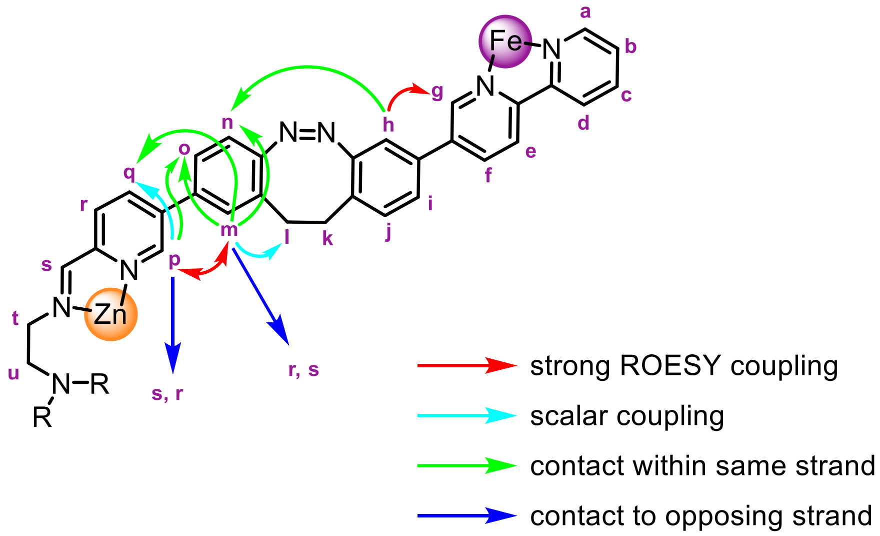

Figure S60. Contacts in ZnFe**L** according to one-dimensional ^{1}H ROESY spectra for signals H-h, H-p and H-m.

Table S12. List of observed ^{1}H ROESY couplings and measured distances in computational solution model of ZnFe**L**. The three intense cross peaks (marked red) can be used to determine the relative stereochemistry of ZnFe**L** (see Section S6.4). Some of the weak signals can likely be assigned to interactions between different ligand strands (distances marked red).

Protons	^{1}H ROESY cross peak	Distance [Å]
**h–g**	**strong**	2.2
**h–n**	weak	4.8
**m–p**	**strong**	2.3
**m–l**	weak	2.4/2.7
**m–q**	weak	4.8 (same strand)<br>4.8 (second ligand)
**m–r**	weak	5.9 (same strand)<br>4.7 (second ligand)
**m–s**	weak	7.0 (same strand)<br>5.0 (second ligand)
**m–o**	weak	4.3 (same strand)<br>4.7 (second ligand)
**m–n**	weak	4.9 (same strand)<br>6.7 (second ligand)
**p–m**	**strong**	2.3
**p–q**	weak	4.3 (same strand)<br>4.8 (second ligand)
**p–s**	weak	5.4 (same strand)<br>4.4 (second ligand)
**p–r**	weak	4.9 (same strand)<br>4.5 (second ligand)
**p–o**	weak	4.5 (same strand)<br>5.0 (second ligand)

S6.4.2 2D ROESY NMR of redissolved Fe2L single crystals

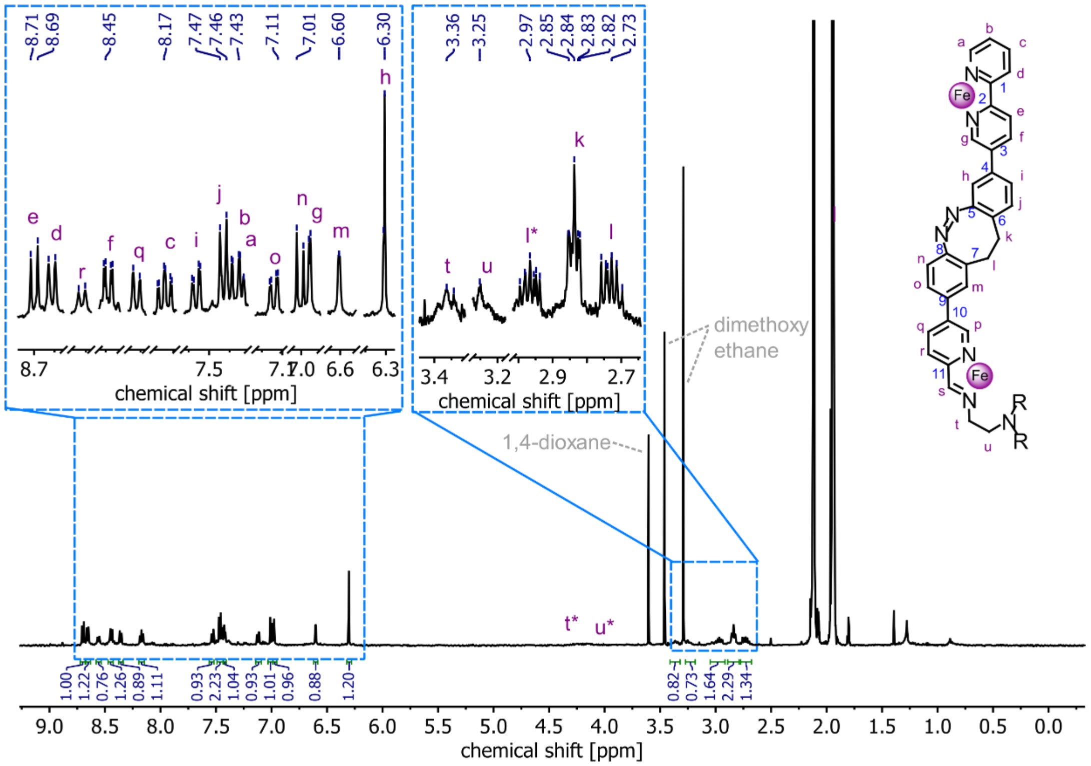

Figure S61. ^1H NMR spectrum (500 MHz, CD_3CN, 298 K) of redissolved single crystals of Fe_2**L** showing residual 1,2-dimethoxyethane and its decomposition product 1,4-dioxane that were used during crystallization. No differences to previous spectra are observed.

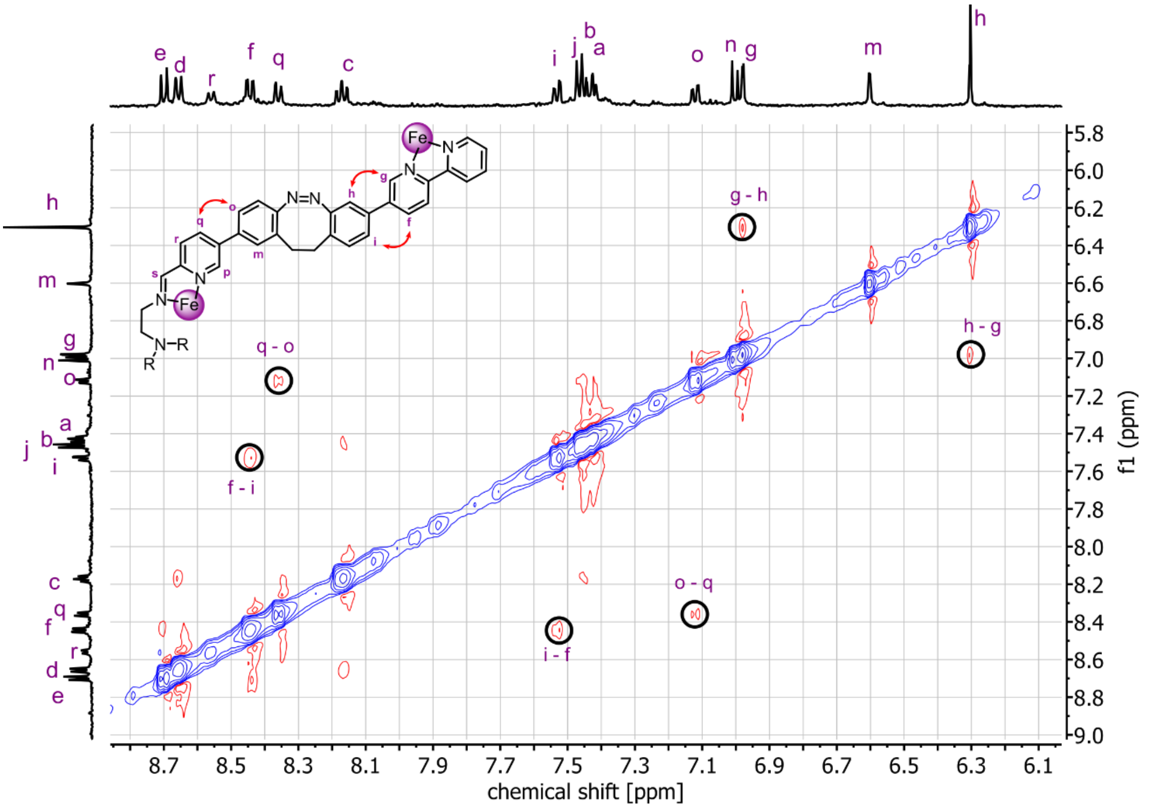

Figure S62. Partial $^1H,^1H$ ROESY NMR spectrum (500 MHz, CD_3CN, 298 K) of redissolved single crystals of Fe_2**L** showing through-space coupling between protons H-g and H-h, H-q and H-o, and H-f and H-i, as is expected for the diastereomer observed in the solid-state structure.

S6.4.3 2D ROESY NMR of Zn_2**L**

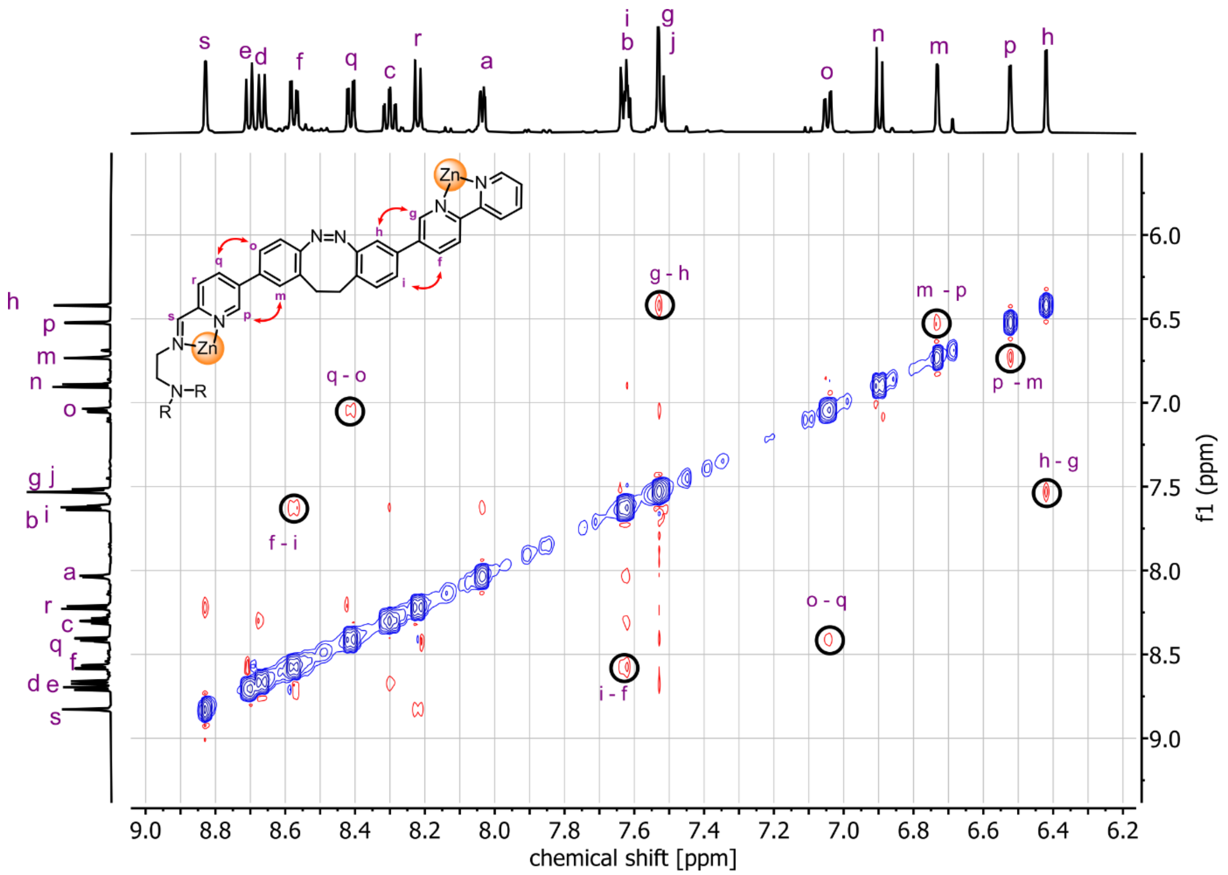

Figure S63. Partial ^{1}H,^{1}H ROESY NMR spectrum (500 MHz, CD3CN, 298 K) of Zn_2**L** showing through-space coupling between protons H-p and H-m, H-g and H-h, H-q and H-o, and H-f and H-i, as is expected for the diastereomer observed in the solid-state structures of Fe_2**L** and ZnFe**L**.

S7 Photochemical characterisation

S7.1 General procedures for the illumination of different types of samples

***Ex-situ* illumination (NMR and MS).** Portable, built-in-house light sources equipped with custom 3D-printed adapters were used to irradiate NMR and MS samples (Figure S60). The samples were promptly subjected to measurement, following irradiation to reduce thermal relaxation to a minimum.

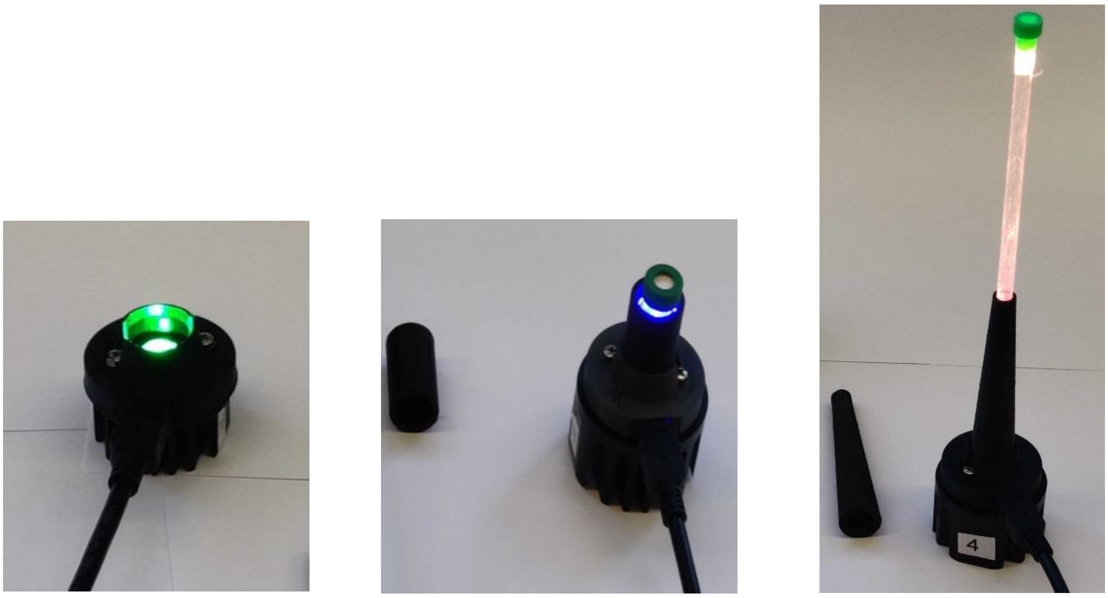

Figure S64. Pictures of the *ex-situ* illumination setup with USB-powered homemade light sources using commercial LED chips with 3D printed adaptors for 2 mL GC vials and 5 mm NMR-tubes.

***In-situ* illumination (NMR).** The NMR sample was fitted with an insert tube made from quartz glass and a quartz glass optical fibre was pushed down into the insert. The end of this fibre was non-terminated, and the exposed surface had been roughened to ensure even and omnidirectional illumination (Figure S61). This construction was lowered into the NMR device using an aluminium rod to avoid damage to the fibre. The other end of the optical fibre was connected to the light source so the sample could be irradiated inside the spectrometer.[20,21]

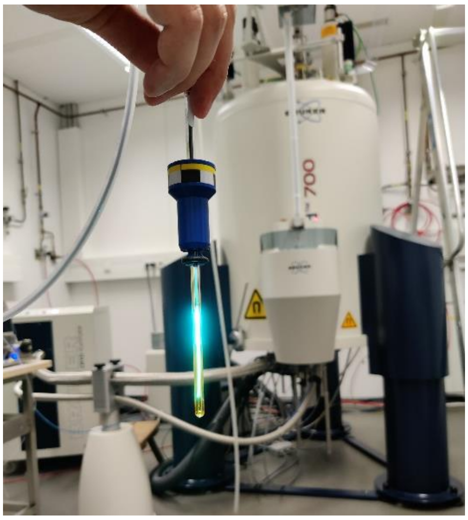

Figure S65. NMR sample with quartz glass insert and optical fibre for in-situ illumination during NMR experiments.

***In situ* illumination (UV-vis).** Samples were irradiated perpendicularly to the measurement axis within the UV-vis spectrometer using optical fibres connected to an LED light source (see Section S1; Figure S62). To facilitate the irradiation of the cuvettes perpendicularly to the spectrometer's measurement axis, a hole matching the diameter of the quartz glass fibre was drilled at the centre of the Teflon stopper used to seal the UV-vis cuvettes. This configuration enabled the connection of an optical fibre to the cuvette, allowing the irradiation of the samples from above.

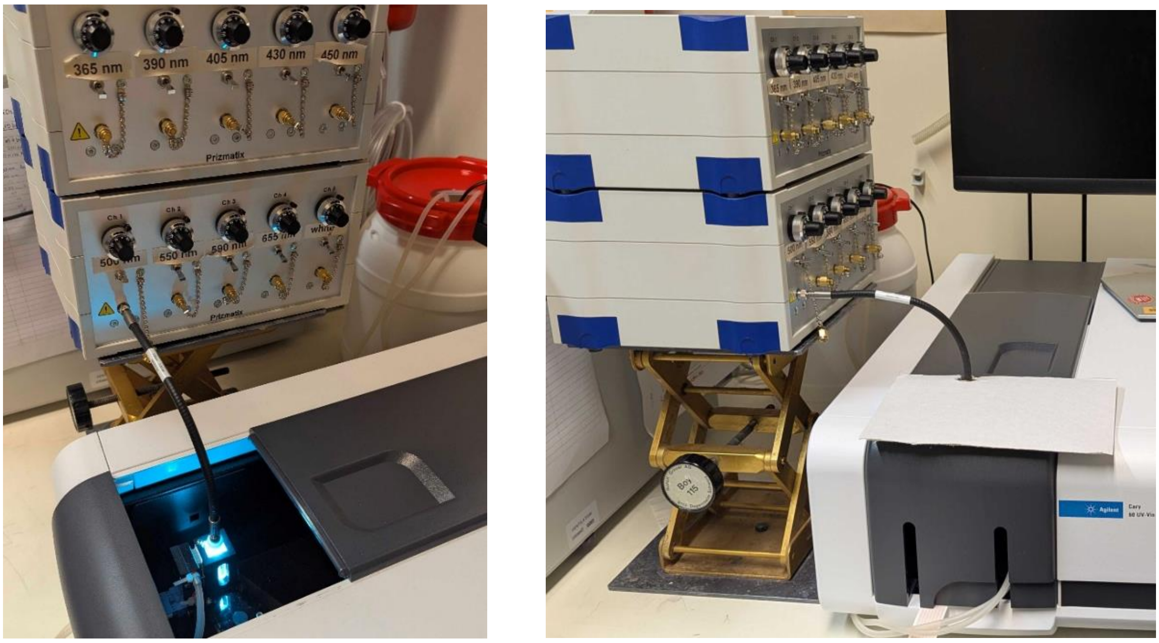

Figure S66. Pictures of the *in-situ* illumination setup showing fibre collimated LED-sources connected to the cuvette with open (left) and closed measurement chamber (right).

Determination of the photo stationary state (PSS). Photostationary states of the photoswitches were determined using ^{1}H NMR spectroscopy. For this purpose, two signals corresponding to the same proton were integrated, and the PSS was calculated based on the resulting integral ratios. To ensure the highest accuracy, well-separated and clearly resolved signals were selected for the analysis, as recommended in the literature.[22,23]

S7.2 Geometry changes of aldehyde **1** during photoswitching

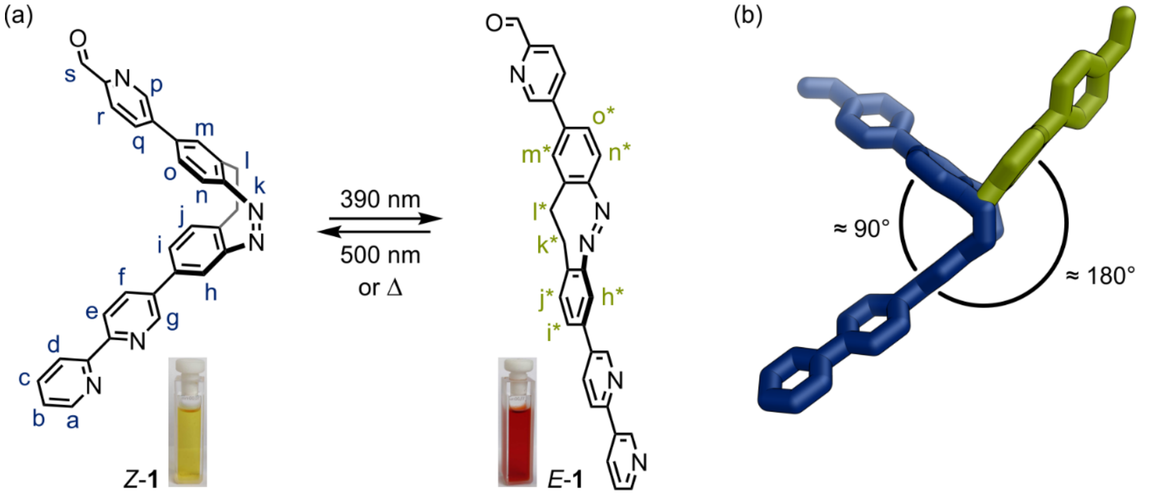

Figure S67. (a) ChemDraw representations of aldehyde **1** in its *E* and *Z*-states with photographs of an acetonitrile solution of **1** before and after irradiation with 405 nm light, showing a drastic colour change from yellow to red. (b) Three-dimensional representation of aldehyde **1** in its *E* and *Z*-states (green and blue, respectively), showcasing the difference in substituent orientation that changes from 90° in the *Z*-state to 180° in the *E*-state. The three-dimensional representations were modelled and pre-optimized using Avogardo[12] (Version 1.2.0) on UFF[13] level before final geometry optimisations were performed on GFN2-xTB[16] level (Version 6.6.0) and the resulting structures visualized using PyMol[18] (Version 3.1.0a OpenSource). The structures are available as supporting information.

S7.3 Photoswitching and thermal relaxation (UV-vis)

Table S13. Overview over photophysical properties of aldehyde **1** and helicates.

**structure**	$\lambda_{irr}(Z\rightarrow E)$ [nm]	$\lambda_{irr}(E\rightarrow Z)$ [nm]	$\tau_{1/2}$(298 K) [min]
**1**	390	500	165
Zn_2**L**	405	500	114
Co_2**L**	390	500	115
Fe_2**L**	405	500	68
ZnFe**L**	405	500	94

S7.3.1 Aldehyde subcomponent **1**

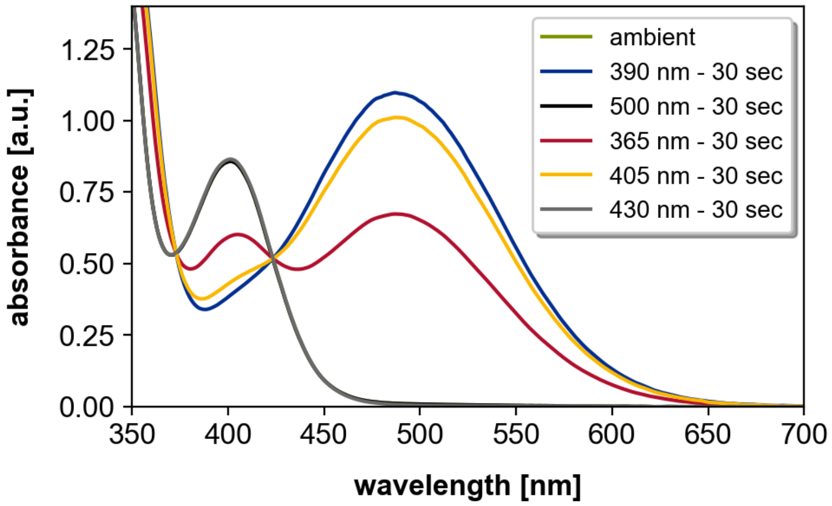

Figure S68. UV-vis spectra of aldehyde **1** (CH_3CN, 0.06 mM) before and after irradiation with different wavelengths.

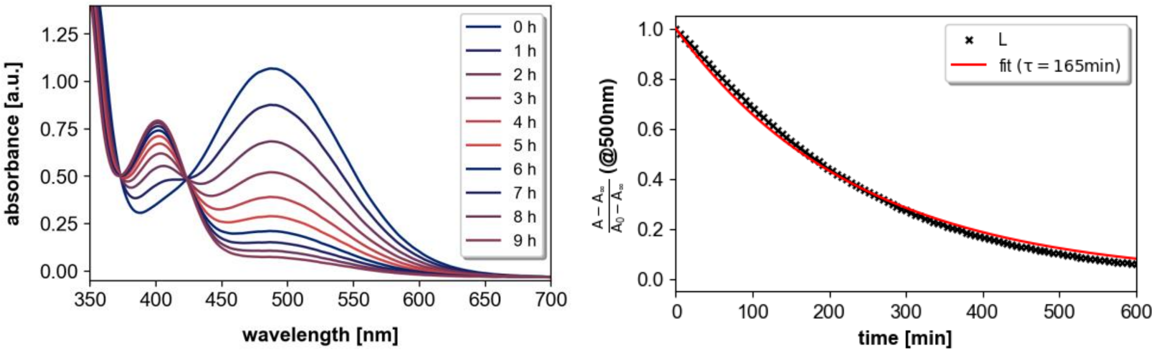

Figure S69. UV-vis spectra (left) and fit (right) of the thermal relaxation of aldehyde **1** (CH_3CN, 0.06 mM, 25 °C) after irradiation at 390 nm for 1 minute. Thermal half-life of **1** was determined to be $\tau_{½}$ = 165 min.

S7.3.2 Zn_2**L**

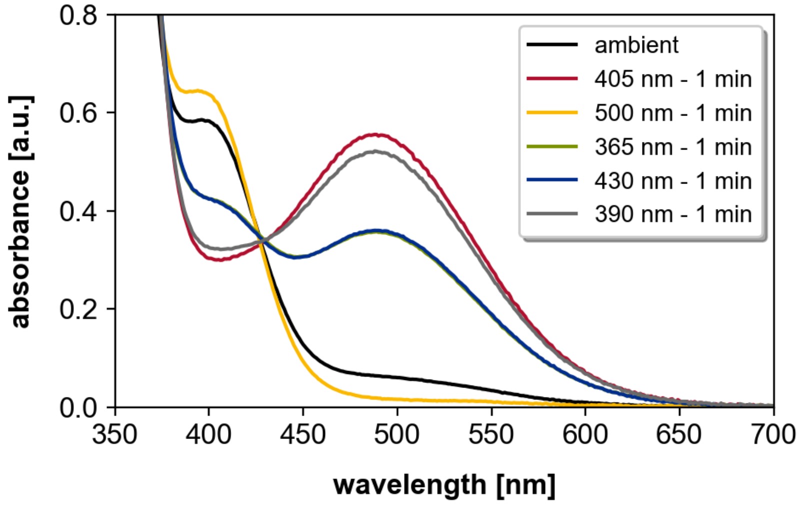

Figure S70. UV-vis spectra of Zn_2**L** (CH_3CN, 0.03 mM) before and after irradiation with different wavelengths.

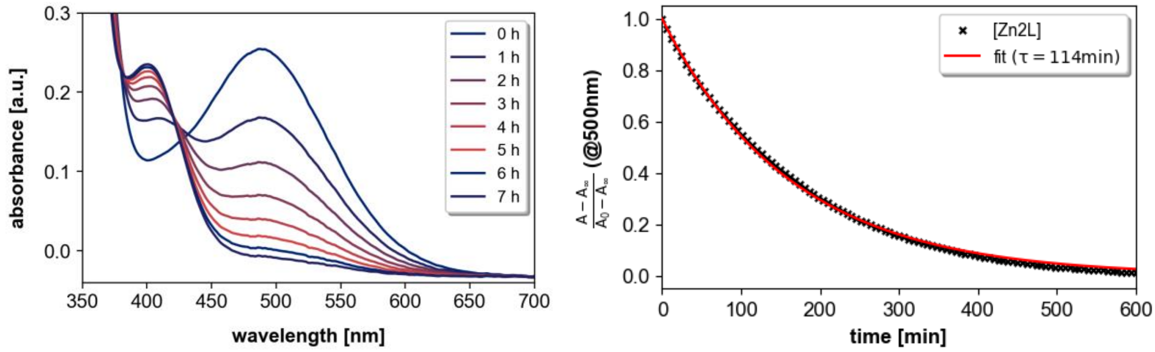

Figure S71. UV-vis spectra (left) and fit (right) of the thermal relaxation of Zn_2**L** (CH_3CN, 0.03 mM, 25 °C) after irradiation at 405 nm for 1 minute. Thermal half-life of Zn_2**L** was determined to be $\tau_{½}$ = 114 min.

S7.3.3 Co_2**L**

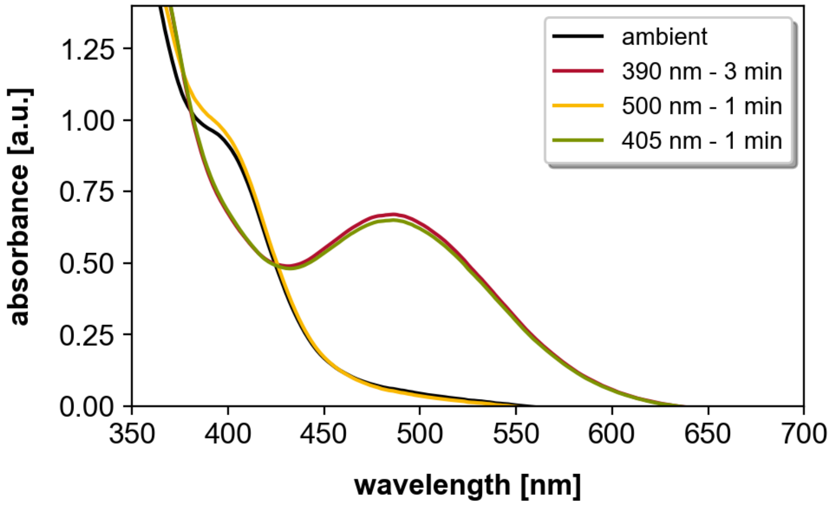

Figure S72. UV-vis spectra of Co_2**L** (CH_3CN, 0.03 mM) before and after irradiation with different wavelengths.

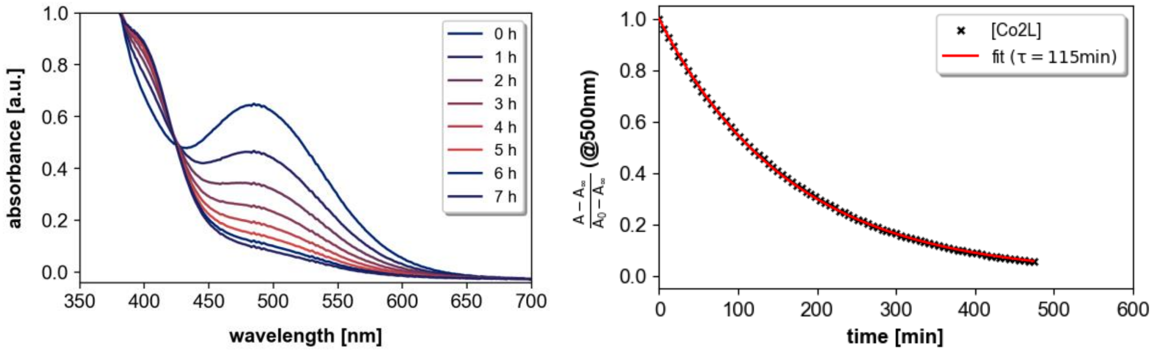

Figure S73. UV-vis spectra (left) and fit (right) of the thermal relaxation of Co_2**L** (CH_3CN, 0.03 mM, 25 °C) after irradiation at 405 nm for 1 minute. Thermal half-life of Co_2**L** was determined to be $\tau_{½}$ = 115 min.

S7.3.4 Fe_2**L**

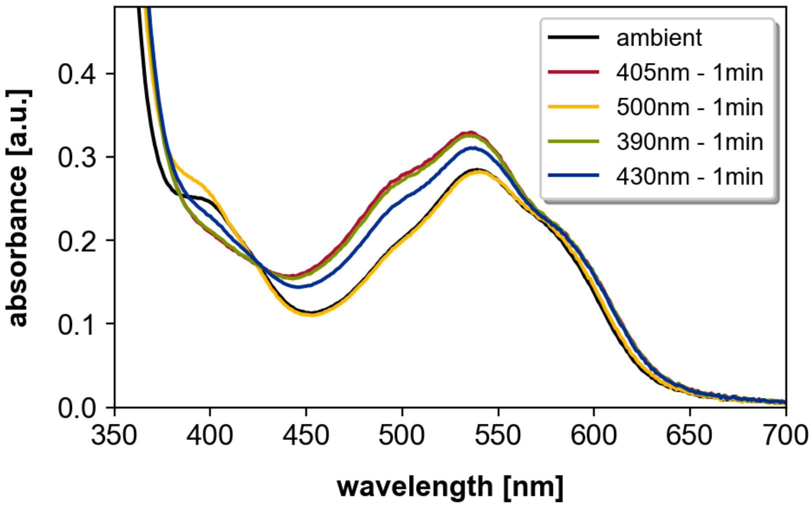

Figure S74. UV-vis spectra of Fe_2**L** (CH_3CN, 0.03 mM) before and after irradiation with different wavelengths.

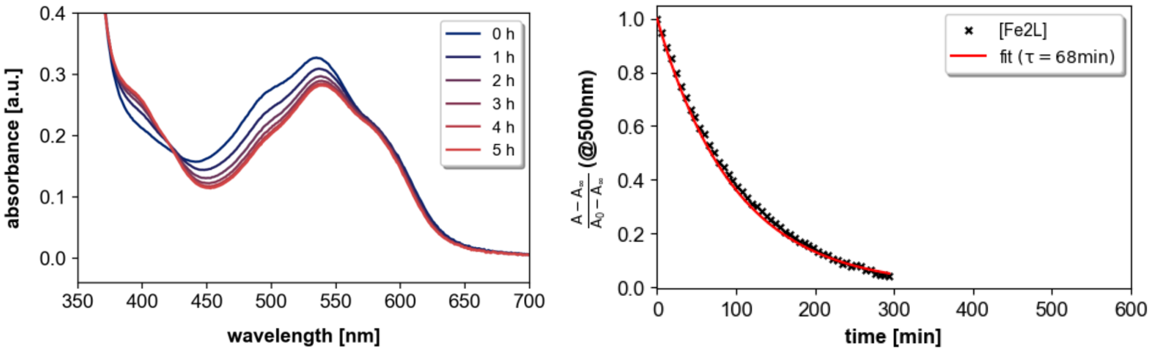

Figure S75. UV-vis spectra (left) and fit (right) of the thermal relaxation of Fe_2**L** (CH_3CN, 0.03 mM, 25 °C) after irradiation at 405 nm for 1 minute. Thermal half-life of Fe_2**L** was determined to be $\tau_{½}$ = 68 min.

S7.3.5 ZnFe**L**

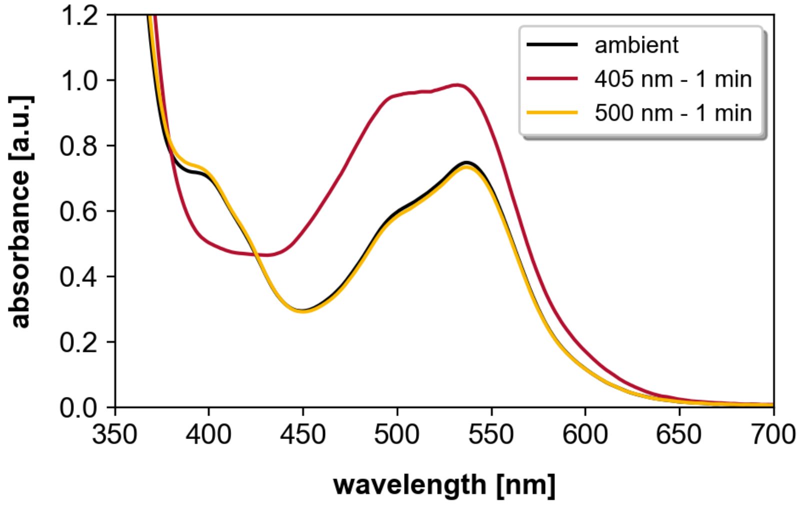

Figure S76. UV-vis spectra of ZnFe**L** (CH_3CN, 0.03 mM) before and after irradiation with different wavelengths.

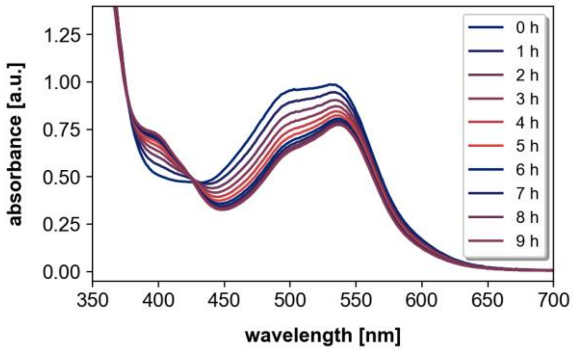

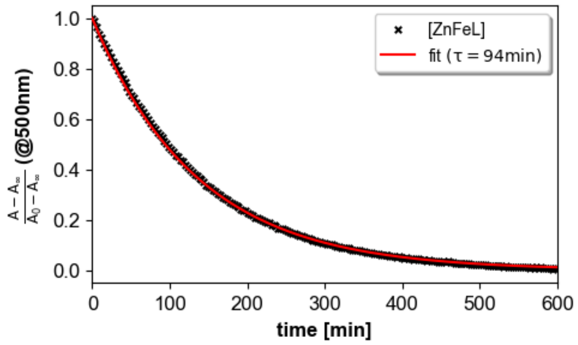

Figure S77. UV-vis spectra (left) and fit (right) of the thermal relaxation of ZnFe**L** (CH_3CN, 0.03 mM, 25 °C) after irradiation at 405 nm for 1 minute. Thermal half-life of ZnFe**L** was determined to be $\tau_{½}$ = 94 min.

S7.3.6 Photochemical fatigue of Zn_2**L**

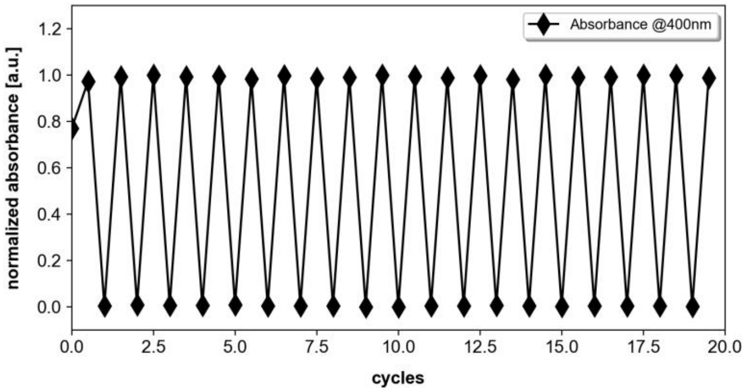

Figure S78. Absorbance of Zn_2**L** (0.03 mM, CH_3CN) at 400 nm after alternating irradiations at 405 nm and 500 nm demonstrating reversible photoisomerization cycles of Zn_2**L**.

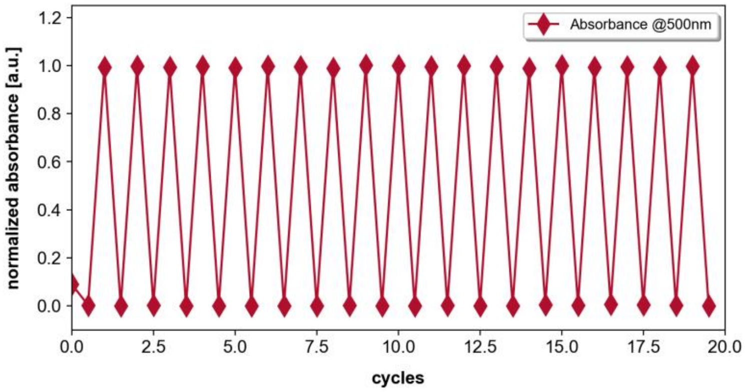

Figure S79. Absorbance of Zn_2**L** (0.03 mM, CH_3CN) at 500 nm after alternating irradiations at 405 nm and 500 nm demonstrating reversible photoisomerization cycles of Zn_2**L**.

S7.3.7 Difference in UV-vis spectra before and after photoswitching

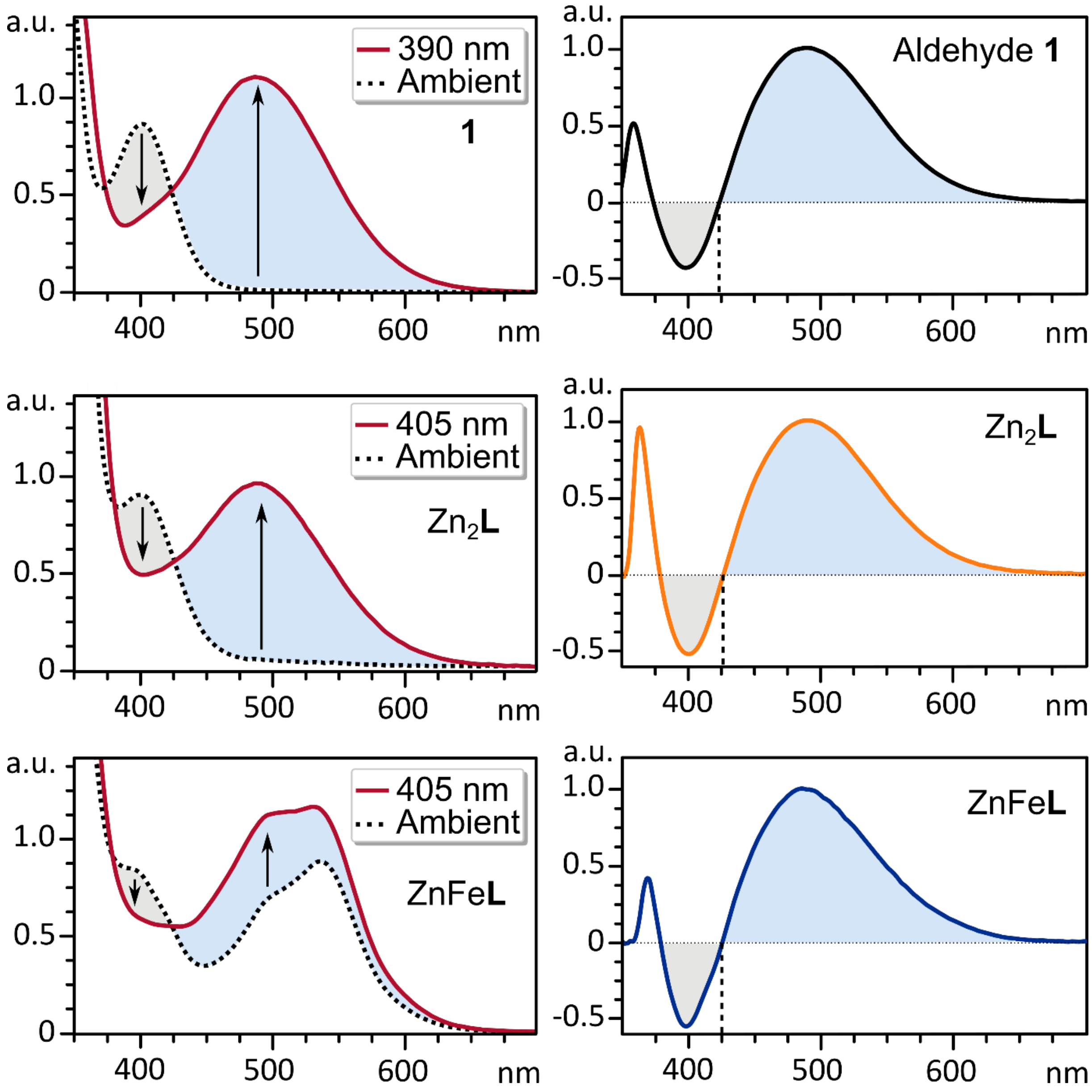

Figure S80. Comparison of the UV-vis spectra of under ambient conditions and at the photostationary state of aldehyde **1**, Zn_2**L**, and ZnFe**L** (left) as well as the normalized difference between the ambient spectrum and the spectrum at the PSS (right), indicating that all changes in the UV-vis spectra during switching arise from the diazocine chromophore.

S7.4 Photoswitching (^{1}H NMR)

S7.4.1 Photostationary states (*ex-situ* illumination)

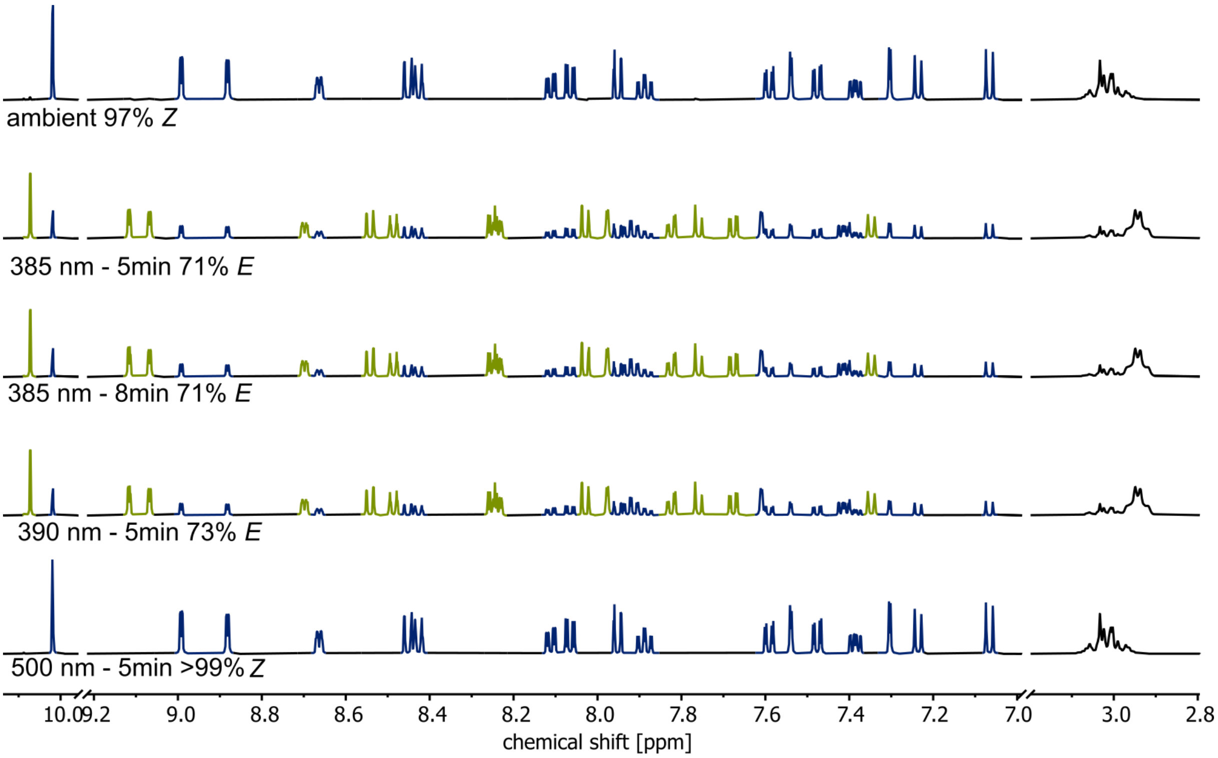

Figure S81. ^{1}H NMR spectra (500 MHz, CD_3CN, 1 mM, 298 K) of ligand precursor **1** before and after irradiation with 385 nm (5 min and 8 min), 390 nm (5 min), and 500 nm (5 min, top to bottom).

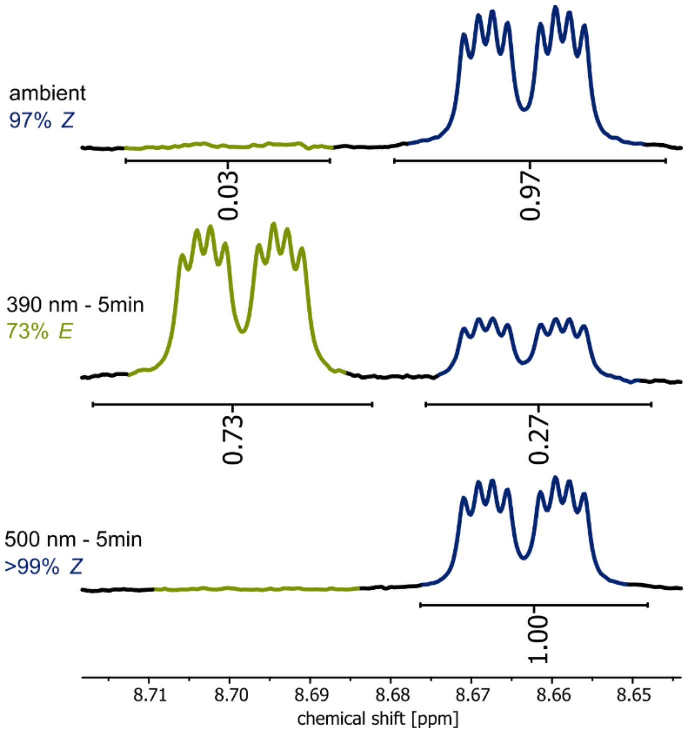

Figure S82. Partial ^{1}H NMR spectra (500 MHz, CD_3CN, 1 mM, 298 K) of ligand precursor **1** before and after irradiation. Integration of H-a proton signals indicates 3% *E*-**1** under ambient conditions (before irradiation) and 73% *E*-**1** after irradiation with 390 nm light for 5 minutes and <<1% *E*-**1** after irradiation with 500 nm light for 5 minutes.

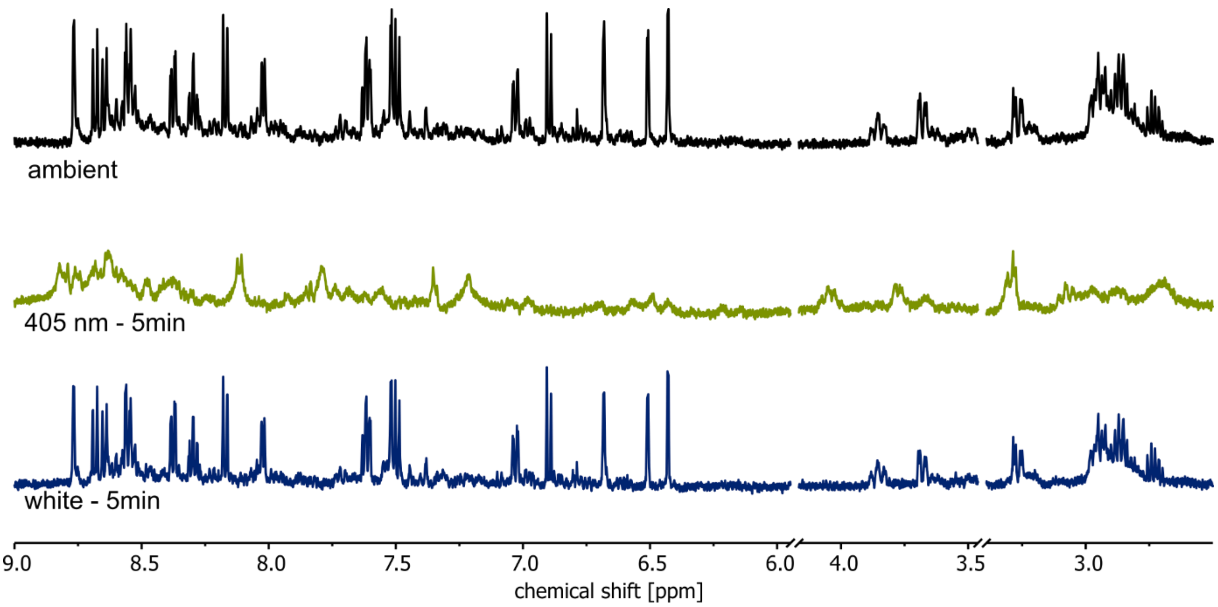

Figure S83. ^{1}H NMR spectra (500 MHz, CD_3CN, 1 mM, 298 K) of Zn_2**L** before and after irradiation with 405 nm (5 min) and white light (5 min, top to bottom).

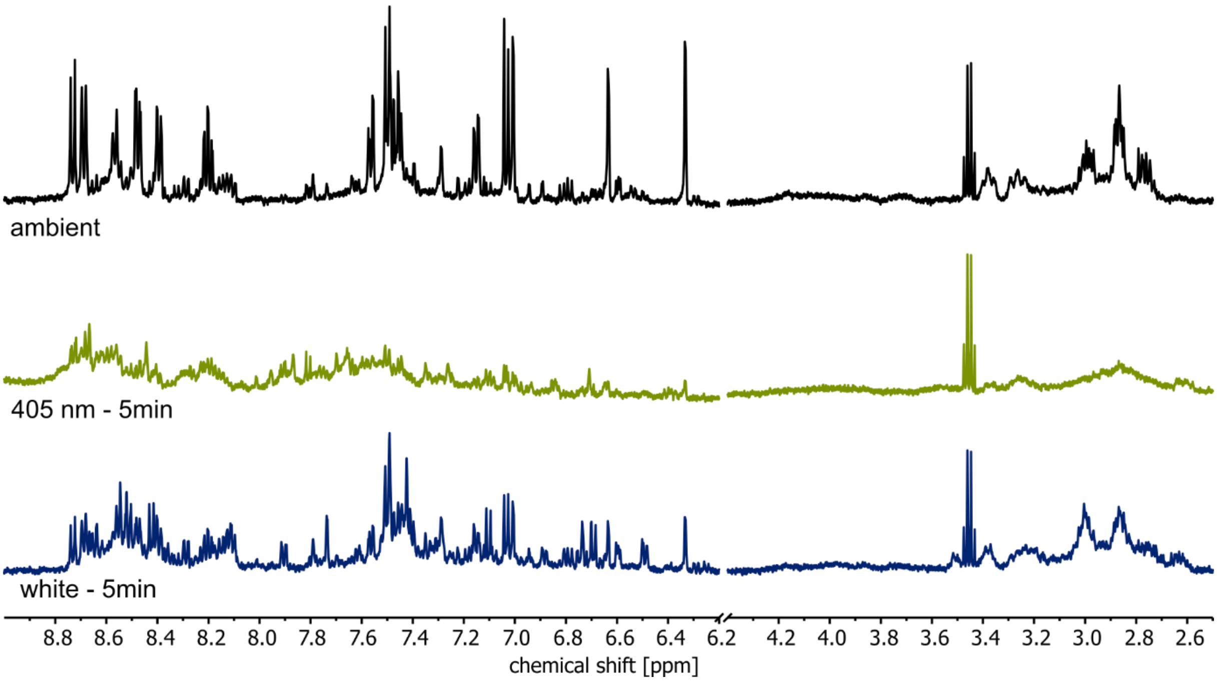

Figure S84. ^{1}H NMR spectra (500 MHz, CD_3CN, 1 mM, 298 K) of Fe_2**L** before and after irradiation with 405 nm (5 min) and white light (5 min, top to bottom).

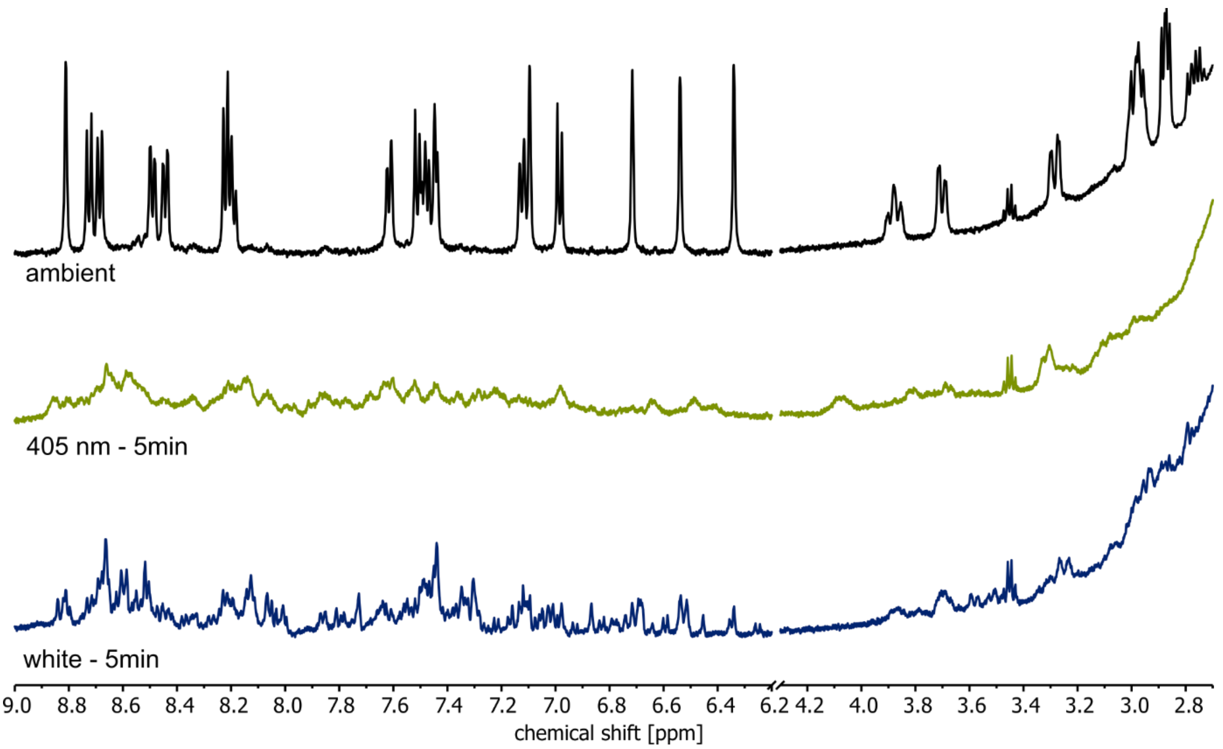

Figure S85. ^{1}H NMR spectra (500 MHz, CD_3CN, 1 mM, 298 K) of ZnFe**L** before and after irradiation with 405 nm (5 min) and white light (5 min, top to bottom).

S7.4.2 DOSY NMR of switched states

Table S14. Diffusion constants *D* and solvodynamic diameters *d* as determined by ^{1}H DOSY NMR experiments

structure	D [10^{-10} m^{2}s^{-1}]			d (=2r) [Å]		
	ambient	after 405 nm irr.	after 515 nm irr.	ambient	after 405 nm irr.	after 515 nm irr.
Zn_2**L**	5.402	5.450	–	20.7	20.5	–
ZnFe**L**	5.716	5.747	5.497	19.6	20.5	20.3

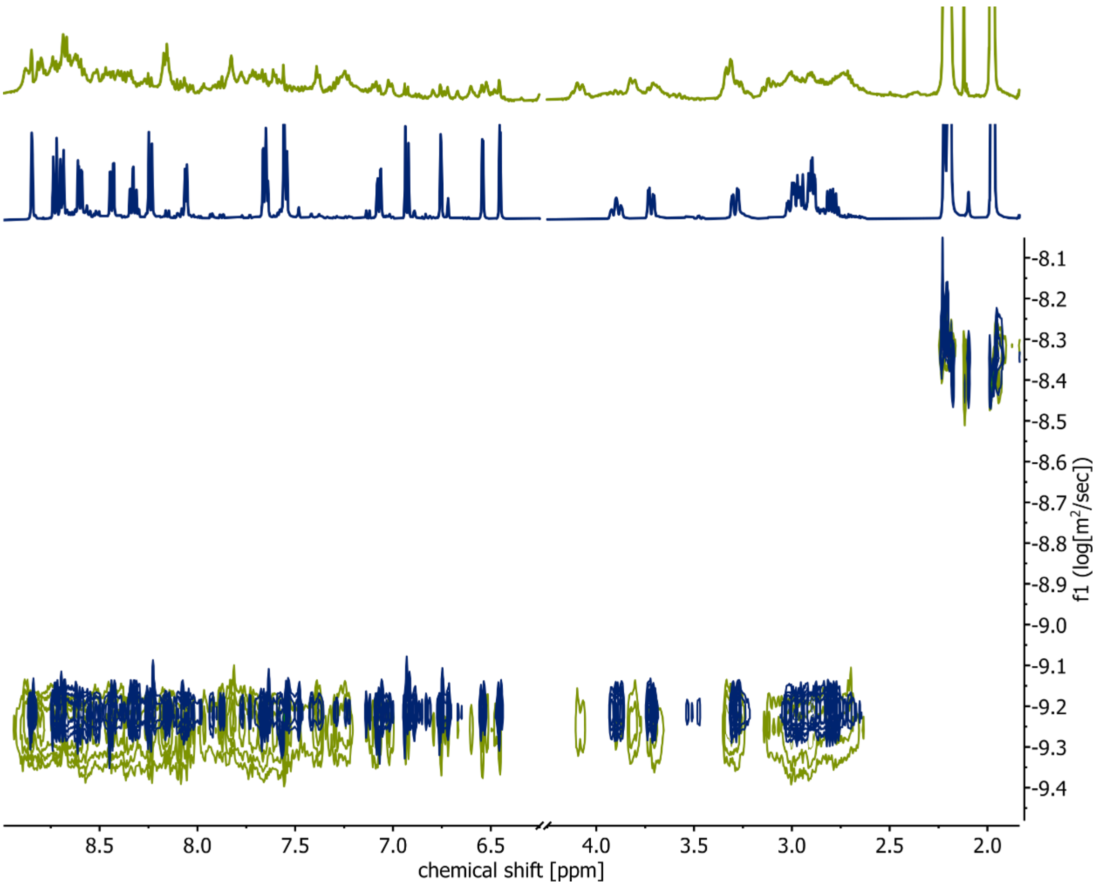

Figure S86. Two dimensional ^{1}H DOSY NMR spectra (500 MHz, CD_3CN, 8 mM, 298 K, D_{20} = 75 ms) of Zn_2**L** before (blue, $D_{amb.}$ = 5.402 · 10^{-10} m^{2}s^{-1}, $d_{amb.}$ = 20.7 Å) and after irradiation with 405 nm (green, D_{405} = 5.450 · 10^{-10} m^{2}s^{-1}, d_{405} = 20.5 Å).

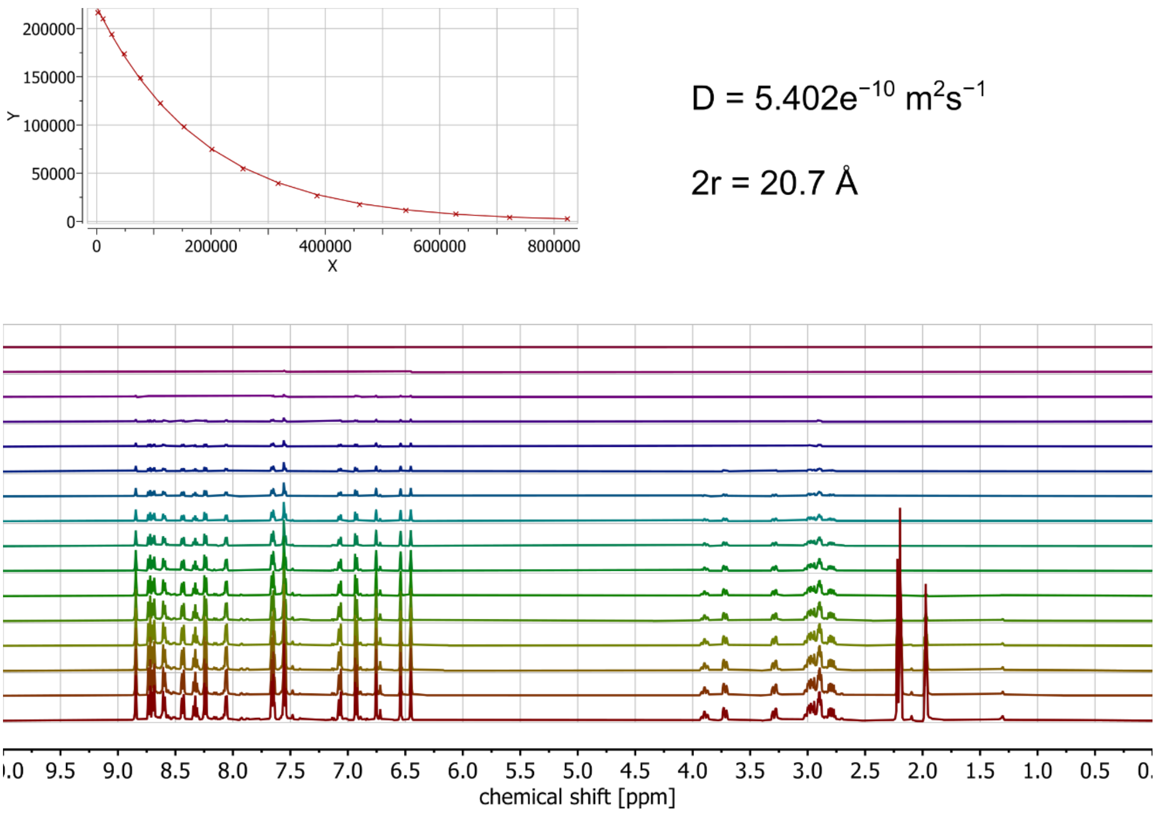

Figure S87. ^{1}H DOSY NMR spectra (500 MHz, CD_3CN, 8 mM, 298 K, D_{20} = 75 ms) of Zn_2**L** under ambient conditions with the diffusion parameter D fitted manually to afford $D_{amb.}$ = 5.402 · 10^{-10} m^2s^{-1} ($d_{amb.}$ = 20.7 Å).

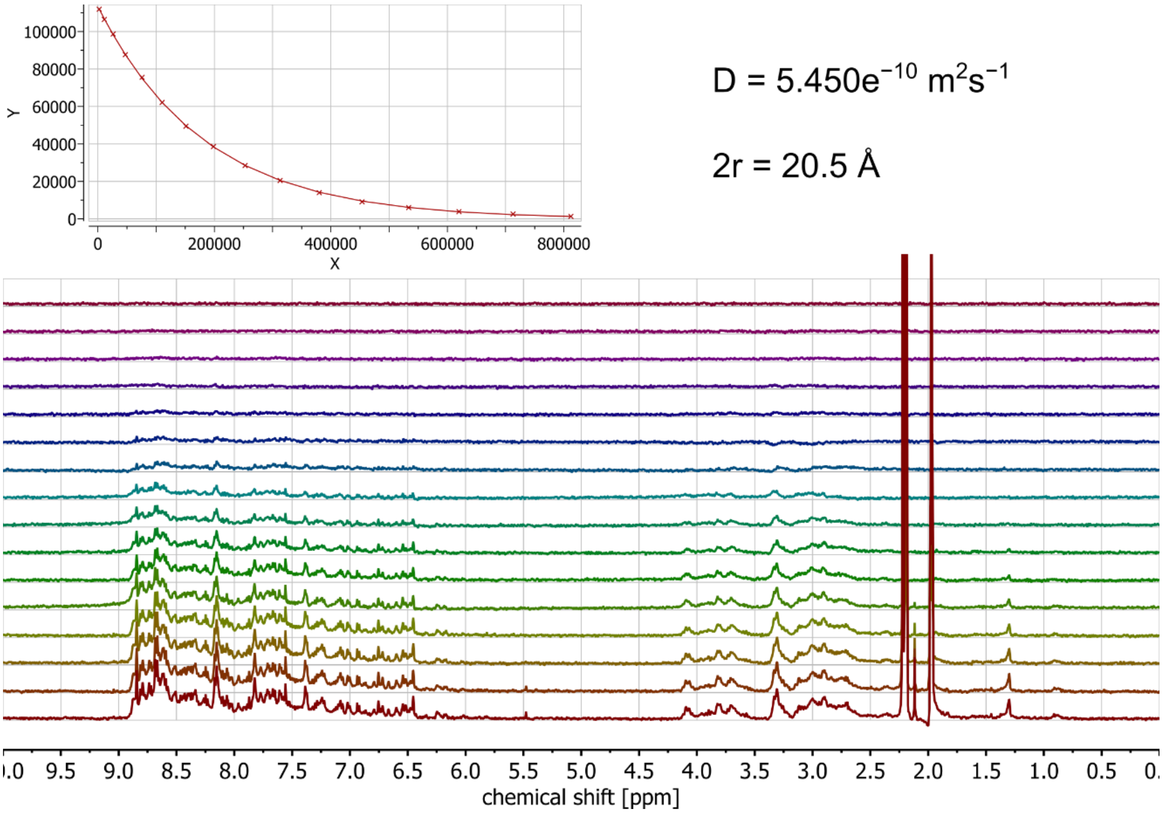

Figure S88. ^{1}H DOSY NMR spectra (500 MHz, CD_3CN, 8 mM, 298 K, D_{20} = 75 ms) of Zn_2**L** after *ex-situ* irradiation with 405 nm for 5 minutes with the diffusion parameter D fitted manually to afford D_{405} = 5.450 10^{-10} m^2s^{-1} (d_{405} = 20.5 Å).

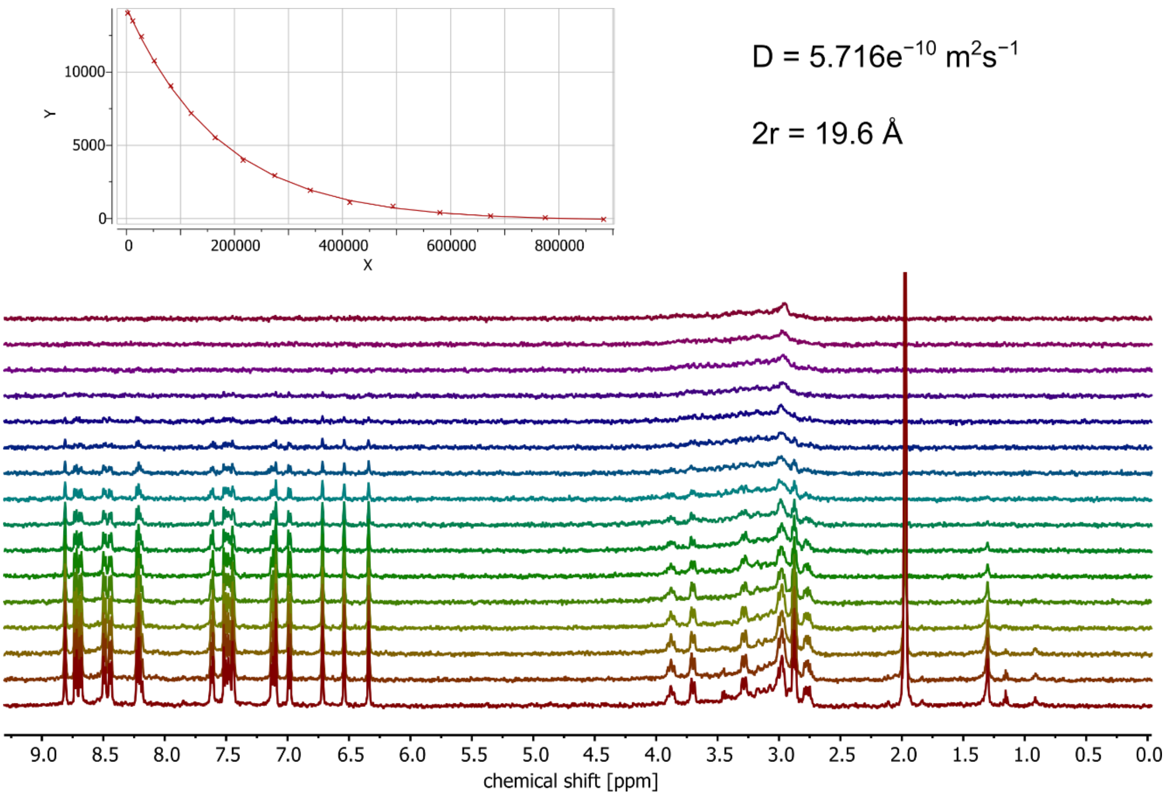

Figure S89. ^{1}H DOSY NMR spectra (500 MHz, CD_3CN, 1 mM, 298 K, D_{20} = 75 ms) of ZnFe**L** under ambient conditions. Due to the low concentration, the conversion to a 2D plot fails and the diffusion parameter D was fitted manually to afford $D_{amb.}$ = 5.716 · 10^{-10} m^2s^{-1} ($d_{amb.}$ = 19.6 Å).

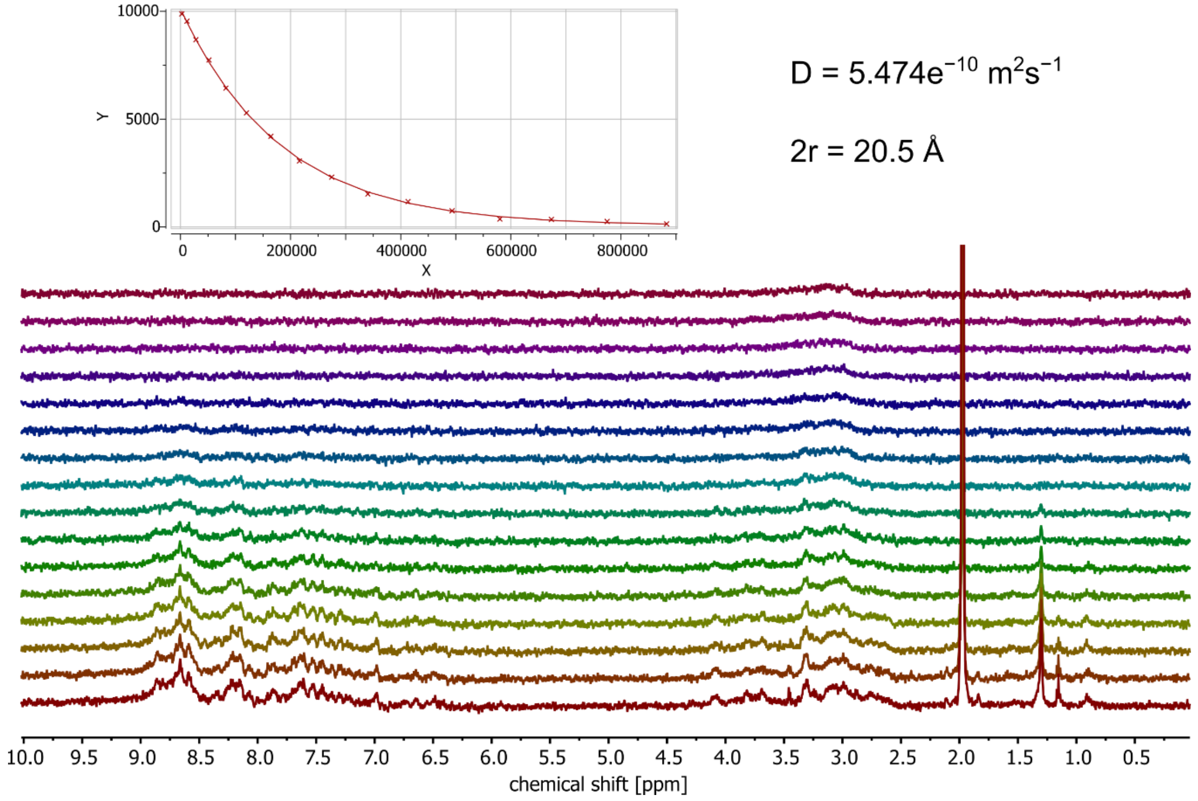

Figure S90. ^{1}H DOSY NMR spectra (500 MHz, CD_3CN, 1 mM, 298 K, D_{20} = 75 ms) of ZnFe**L** after irradiation with 405 nm for 5 minutes. Due to the low concentration, the conversion to a 2D plot fails and the diffusion parameter D was fitted manually to afford D_{405} = 5.474 · 10^{-10} m^2s^{-1} (d_{405} = 20.5 Å).

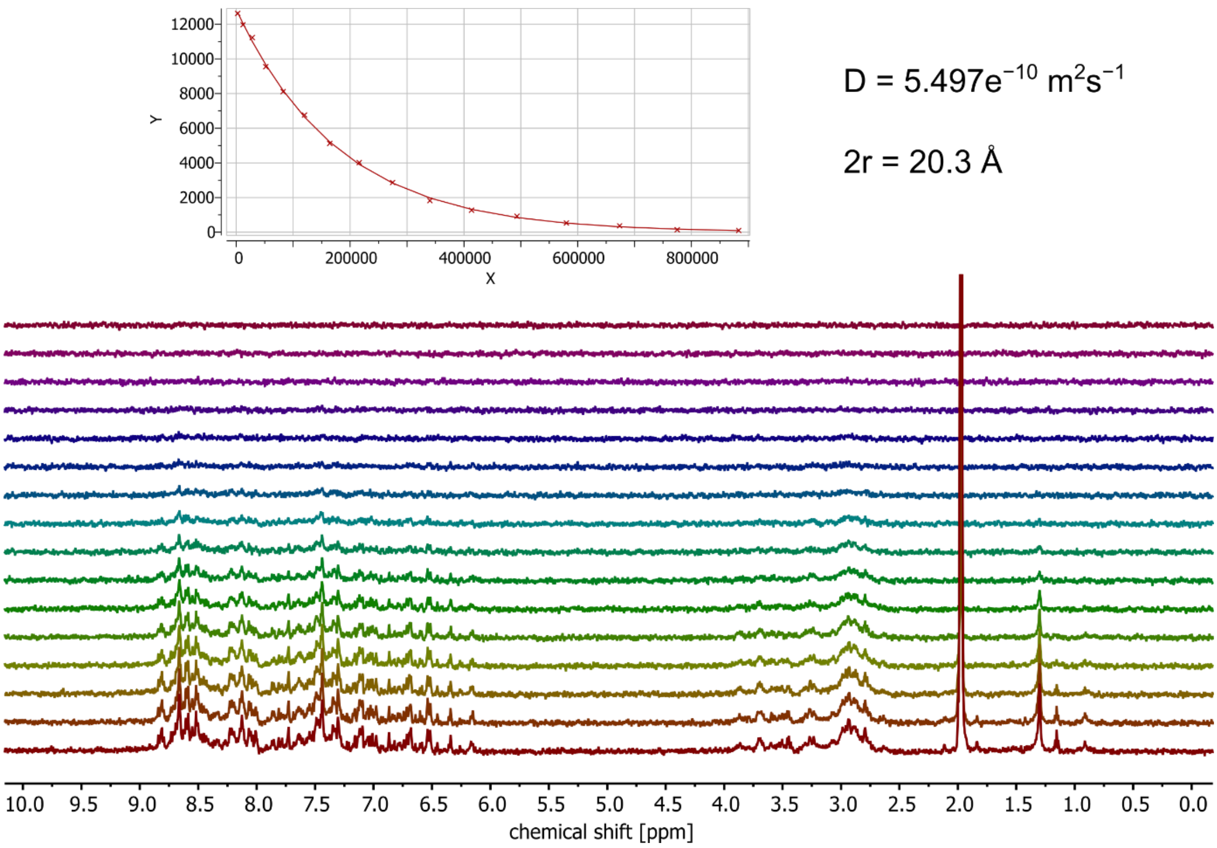

Figure S91. ^{1}H DOSY NMR spectra (500 MHz, CD_3CN, 1 mM, 298 K, D_{20} = 75 ms) of ZnFe**L** after *ex-situ* irradiation with 515 nm for 5 minutes. Due to the low concentration, the conversion to a 2D plot fails and the diffusion parameter *D* was fitted manually to afford $D_{515} = 5.497 \cdot 10^{-10}$ m^{2}s^{-1} (d_{515} = 20.3 Å).

S7.4.3 Photoswitching of ZnFe**L** followed by *in-situ* illumination NMR

To gain further insights into the kinetics of the photoisomerization processes within ZnFe**L** upon irradiation with 405 nm and white light, both processes were investigated via *in-situ* illumination of the samples inside the NMR spectrometer with spectra being recorded every 25 seconds (Figure S92 and Figure S93).

During 405 nm-light irradiation (Figure S92), the signals corresponding to ZnFe**L** disappeared following apparent first-order kinetics (Figure S92, bottom left, all traces). At the same time, at least three new species were formed (Figure S92, bottom right, purple (appears fast), teal (transient), yellow (appears slowly)). Two species appear immediately following apparent first-order kinetics (purple and teal, Figure S92, bottom right), with one of them disappearing again during the reaction (teal, Figure S92, bottom right). This disappearance coincides with the formation of the third species (yellow, Figure S92, bottom right), also following apparent first-order kinetics, indicating that the third species is formed out of the second species. Due to strong signal overlap, no further insights could be gained.

During irradiation of i-*E*-ZnFe**L** with white light (Figure S93), the signals corresponding to the two species formed in the first irradiation step (i-*E*-ZnFe**L**, compare Figure S92) disappear following apparent first-order kinetics (green, teal, Figure S93, bottom left). At the same time, at least two new species are formed (yellow, green, Figure S93, bottom right). One species appears slowly (yellow, bottom right), while the second species is the main product of the white-light irradiation and appears following apparent first-order kinetics (i-*Z*-ZnFe**L**; green, Figure S93, bottom right). Due to strong signal overlap, no further insights could be gained.

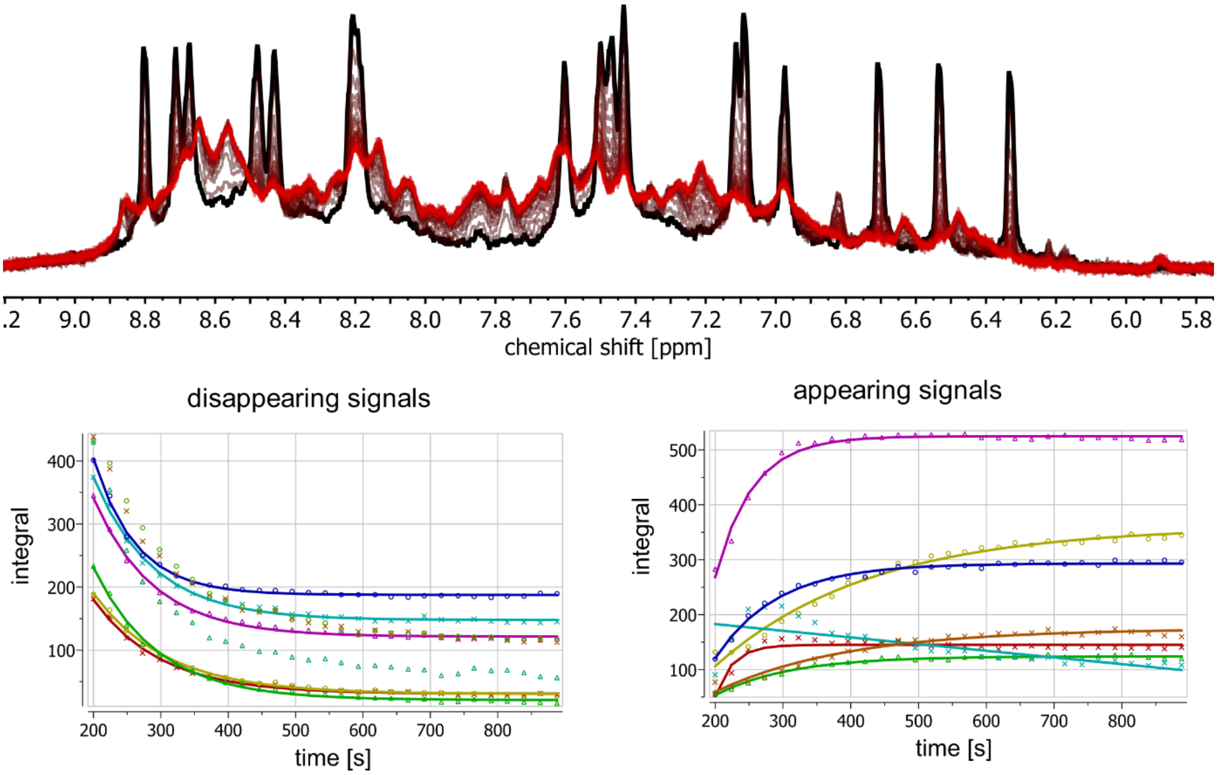

Figure S92. ^{1}H NMR spectra (700 MHz, CD_3CN, 1 mM, 298 K) of ZnFe**L** during *in-situ* irradiation with 405 nm (maroon to red, top) with spectra being measured every 25 seconds for a duration of 15 minutes. Bottom left: Plotted integrals of ZnFe**L** proton signals over time with data points fitted to first-order kinetics. Bottom right: Plotted integrals of appearing proton signals over time with data points fitted to first-order kinetics.

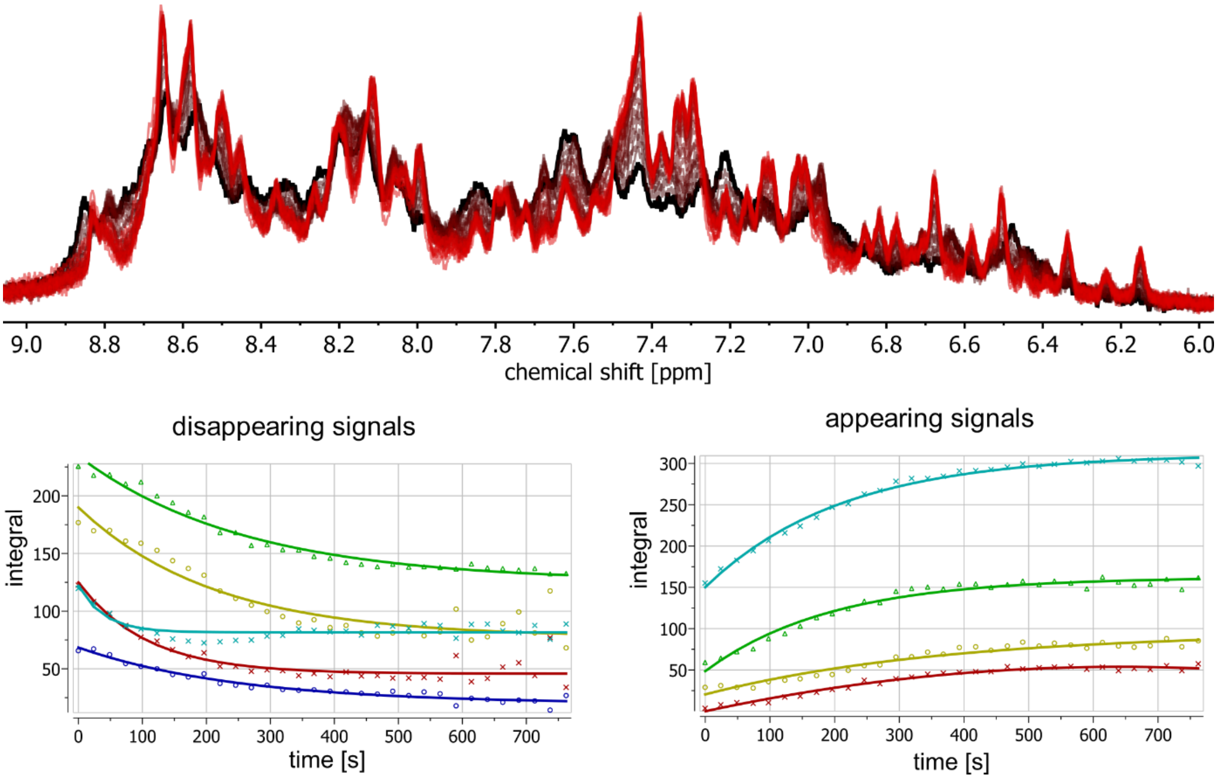

Figure S93. ^{1}H NMR spectra (700 MHz, CD_3CN, 1 mM, 298 K) of ZnFe**L** previously irradiated with 405 nm light following *in-situ* irradiation with white light (maroon to red, top) with a spectrum being recorded every 25 seconds for a duration of 13 minutes. Bottom left: Plotted integrals of i-*E*-ZnFe**L** proton signals over time with data points fitted to first-order kinetics. Bottom right: Plotted integrals of appearing proton signals over time with data points fitted to first-order kinetics.

S7.4.4 NMR kinetics of reforming ZnFe**L** from kinetically trapped state i-*Z*-ZnFe**L**

After the irradiation with 405 nm (Figure S92) and white light (Figure S93) the irradiation ceased and the dark state kinetics of the sample were observed (Figure S94). Over the course of 4.5 hours, only small changes were observed. One group of signals decreased in intensity (green, Figure S94, bottom left) while another group of signals increased in intensity (teal, Figure S94, bottom right), both following apparent first-order kinetics. No significant amount of the starting species ZnFe**L** is observed, and it is not the species that appears, indicating that a stable kinetic trap has been reached, and the mixture of isomers slowly converts into a more stable isomer, that is not the thermodynamic ground state.

In a separate experiment, a sample was consecutive irradiated *ex-situ* with 405 nm and white light and the relaxation was followed by high-temperature ^{1}H NMR at 65 °C (Figure S95). Due to the time required for shimming the magnetic field, the measurement started slightly delayed, and a significant amount of the thermodynamic ground state was already present when the measurement started (approx. 40%). All signals disappear and appear following apparent first-order kinetics (Figure S96).

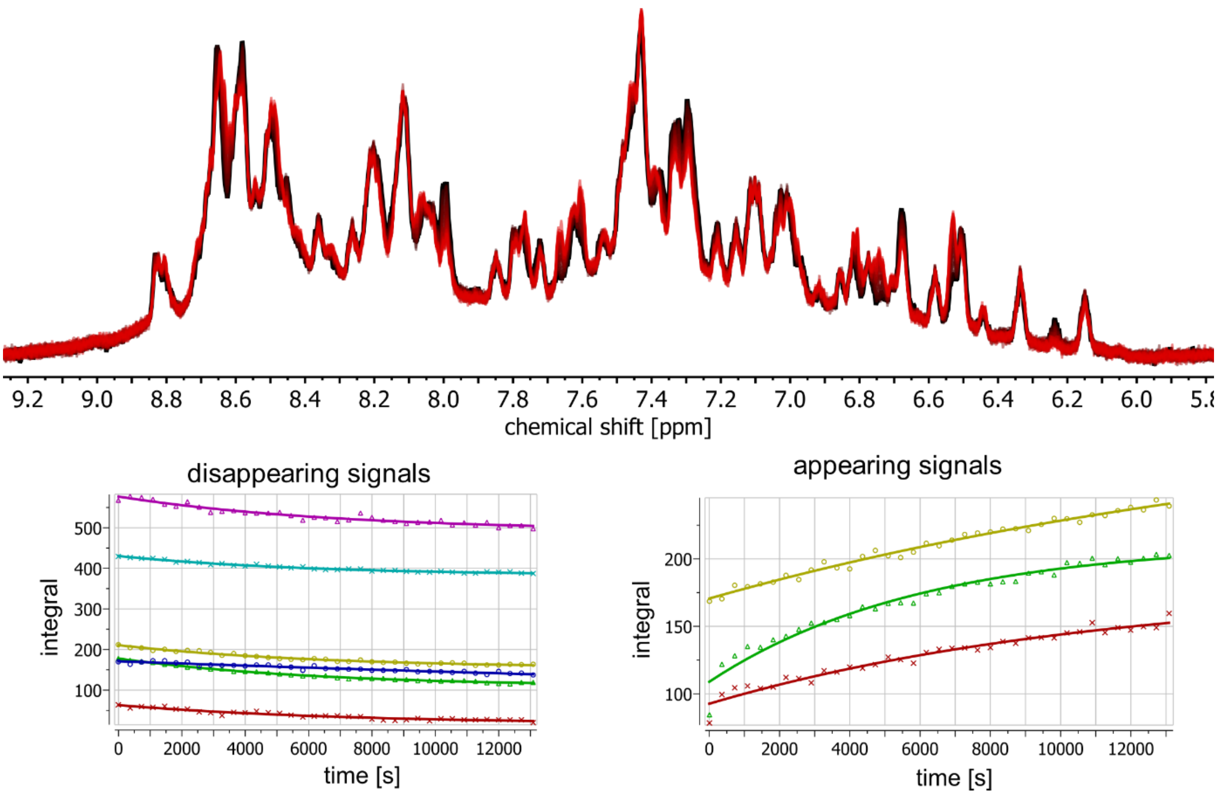

Figure S94. ^{1}H NMR spectra (700 MHz, CD_3CN, 1 mM, 298 K) of ZnFe**L** after consecutive 405 nm and white-light irradiation following thermal structure conversion at 298 K over 230 minutes with a spectrum recorded every 5 minutes (top, maroon to red). Bottom left: Plotted integrals of i-*Z*-ZnFe**L** proton signals over time with data points fitted to first-order kinetics. Bottom right: Plotted integrals of appearing proton signals over time with data points fitted to first-order kinetics. In the dark, a slow conversion of all signals into a second set of signals can be observed. This process follows apparent first-order kinetics.

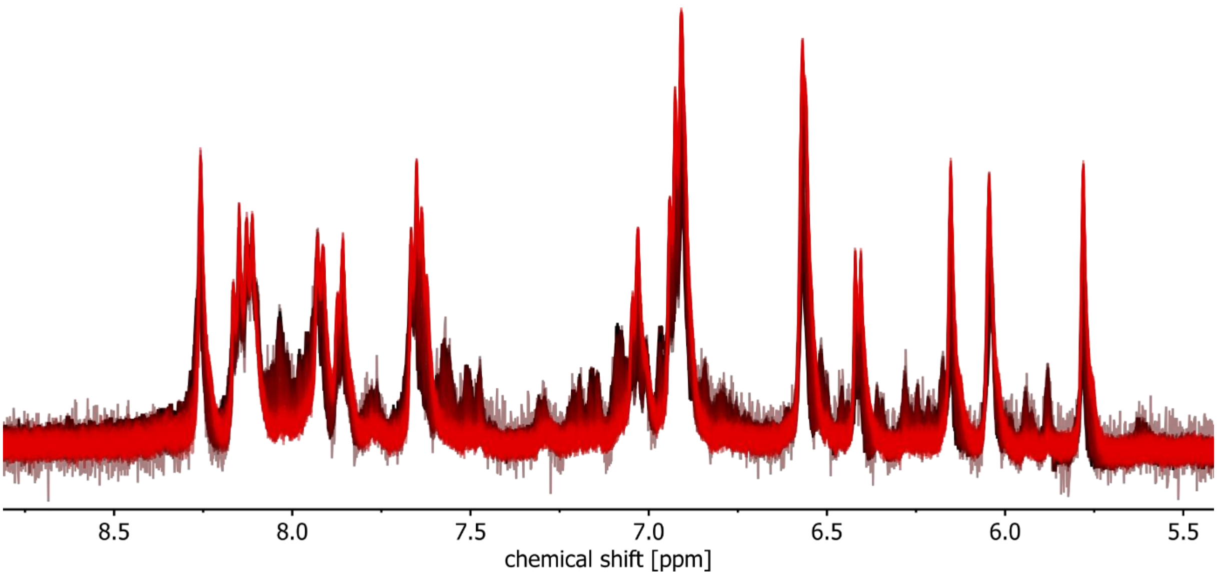

Figure S95. High-temperature ^{1}H NMR spectra (500 MHz, CD_3CN, 1 mM, 338 K) of ZnFe**L** after consecutive *ex-situ* irradiation with 405 nm and white light following conversion of i-*Z*-ZnFe**L** to ZnFe**L** over 4 hours with a spectrum being recorded every minute for the first hour and every 3 minutes after that (maroon to red). At 338 K, the conversion of the formerly kinetically trapped species into the ground state of ZnFe**L** can be observed to follow apparent first-order kinetics (Figure S96).

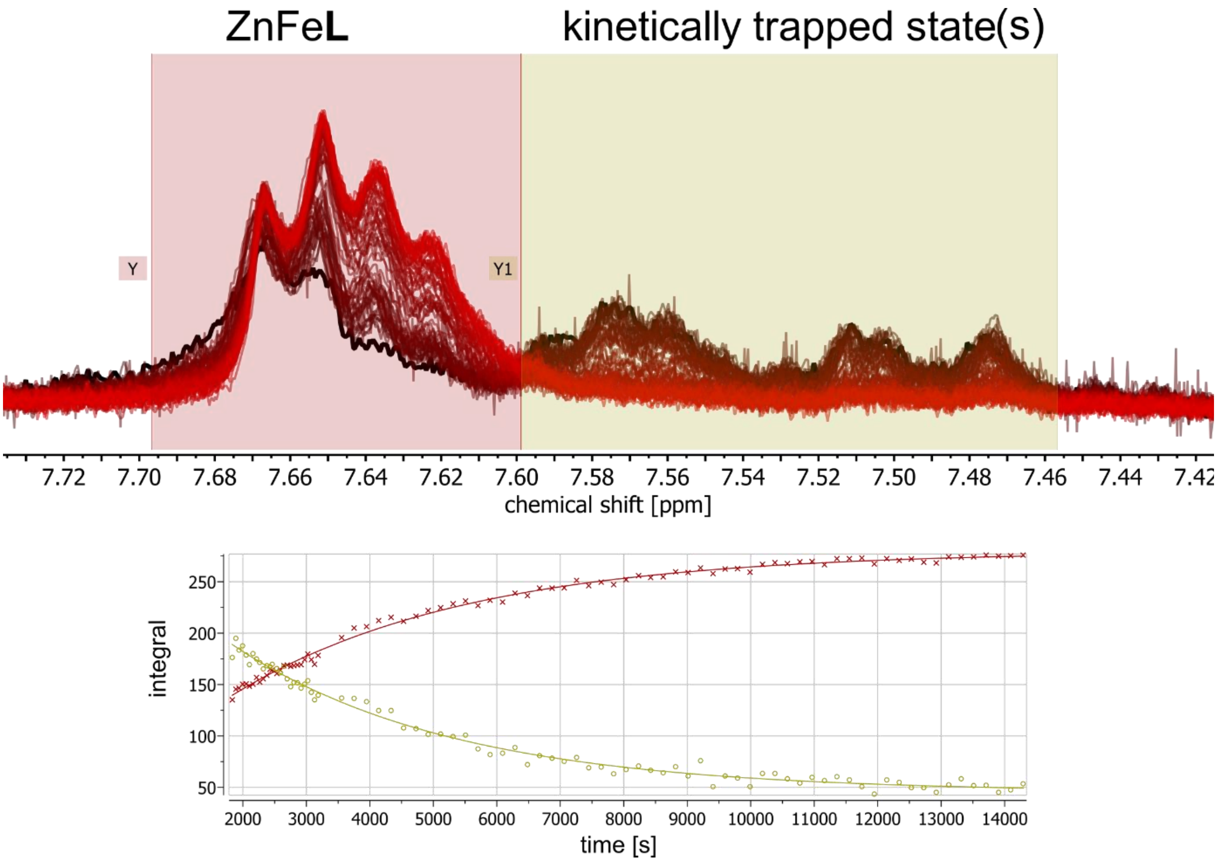

Figure S96. Partial high-temperature ^{1}H NMR spectra (500 MHz, CD_3CN, 1 mM, 338 K) of ZnFe**L** after consecutive *ex-situ* irradiation with 405 nm and white light following conversion of i-*E*-ZnFe**L** to ZnFe**L** over 4 hours with a spectrum being recorded every minute for the first hour and every 3 minutes after that (top, maroon to red, compare Figure S95). Bottom: Plotted integrals of i-*Z*-ZnFe**L** (yellow) and ZnFe**L** (red) proton signals over time with data points fitted to first-order kinetics. Formation of ZnFe**L** helicate follows apparent first-order kinetics with no intermediates being observed

S7.4.5 Low-temperature NMR of the switched states

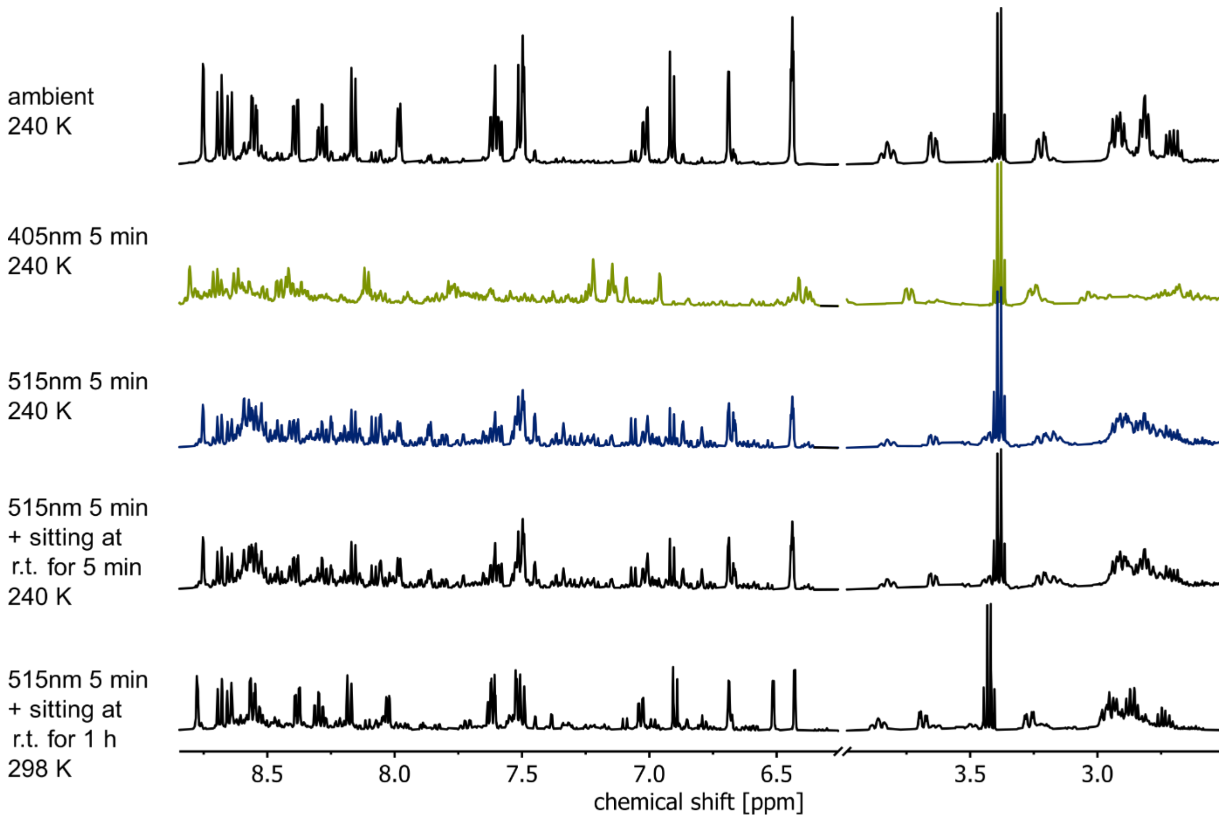

Figure S97. Variable-temperature ^{1}H NMR spectra (500 MHz, CD_3CN, 1 mM) of Zn_2**L** under ambient conditions (240 K), after *ex-situ* irradiation with 405 nm light for 5 min (240 K), after *ex-situ* 515 nm light irradiation for 5 min (240 K), and after leaving the irradiated sample at room temperature for 5 min and 1 hour (240 K and 298 K, respectively; top to bottom). Compared to switching experiments at room temperature (Figure S83), the spectrum after 405 nm irradiation shows many sharp and well-defined signals instead of broadened signals, indicating that a dynamic process was occurring at room temperature. Back-switching was fully reversible at room temperature. In contrast, a spectrum similar to that of kinetically trapped ZnFe**L** is observed after back-switching at low temperatures. Warming the sample up to room temperature reforms the initial spectrum for Zn_2**L**. This indicates that the M_2**L** complexes undergo an isomerization during the photoswitching process, resulting in a kinetically trapped state. Reforming the initial state likely involves dissociation of bipyridine ligands, as the much more labile zinc complex already reforms at a much lower temperature (5 min at 25°C as compared to 5 hours at 65 °C for ZnFe**L**).

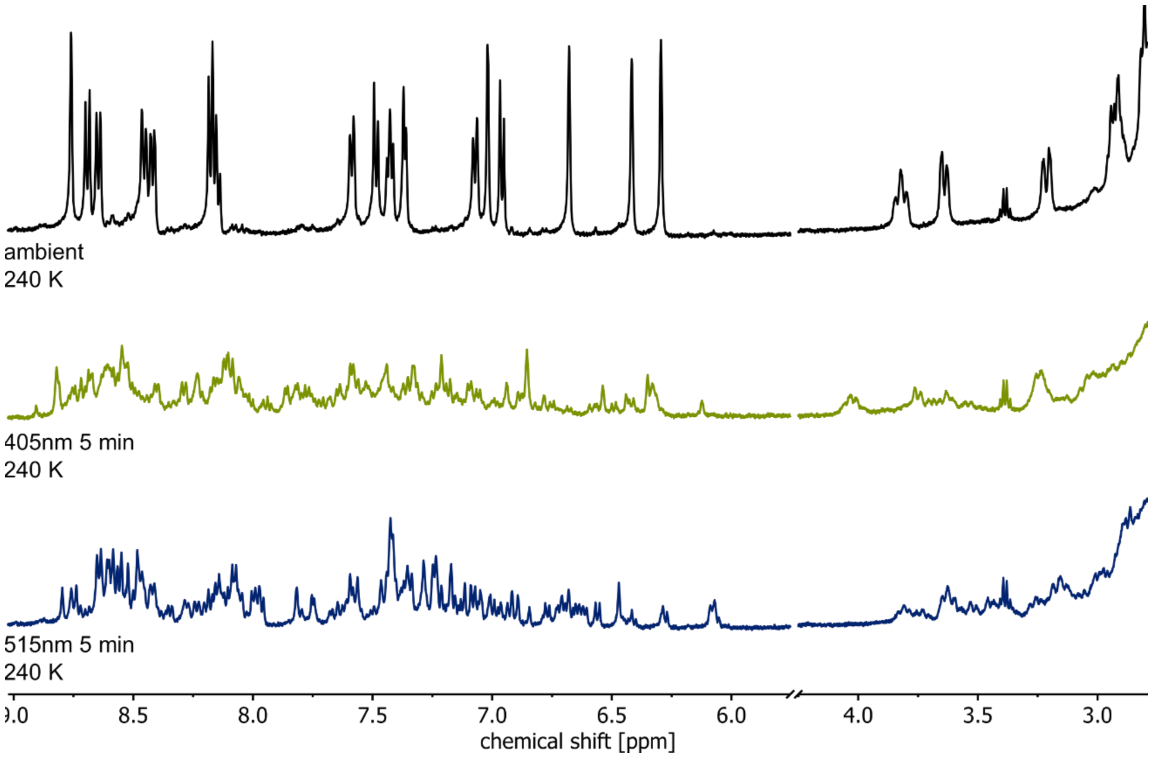

Figure S98. Low temperature ^{1}H NMR spectra (500 MHz, CD_3CN, 1 mM, 240 K) of ZnFe**L** under ambient conditions, after irradiation with 405 nm light for 5 min, and after 515 nm light irradiation for 5 min (top to bottom). Compared to switching experiments at room temperature (Figure S85), the spectrum after 405 nm irradiation shows many sharp and well-defined signals instead of broadened signals, indicating that a dynamic process was occurring at room temperature. The spectrum obtained after back-switching looks very similar to the one observed at room temperature, indicating that the irreversible transformation of the complex to the kinetically trapped state during photoswitching remains fast at low temperatures.

S7.5 Photoswitching of helicates investigated by ESI$^+$ MS

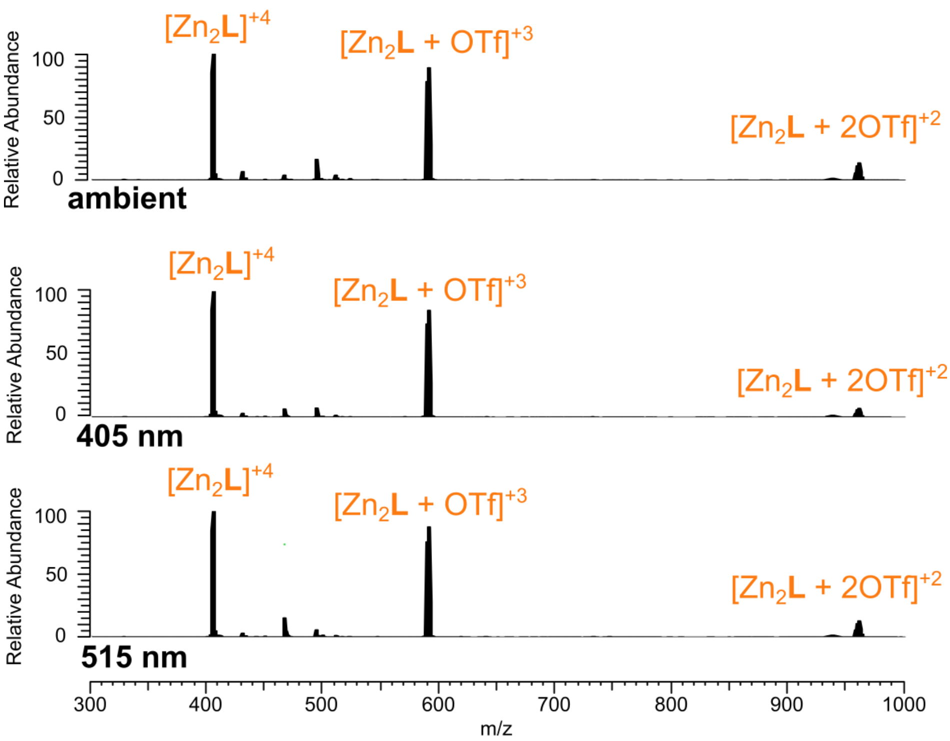

Figure S99. ESI$^+$ mass spectra (CH_3CN, 0.1 mM) of Zn_2**L** under ambient conditions and after irradiation with 405 nm and 515 nm light, respectively (top to bottom). After irradiation, no new peaks are observed, but the relative intensity of the [Zn_2**L** + 2OTf]$^{2+}$ peak decreases slightly after irradiating with 405 nm and recovers when switching back with 515 nm.

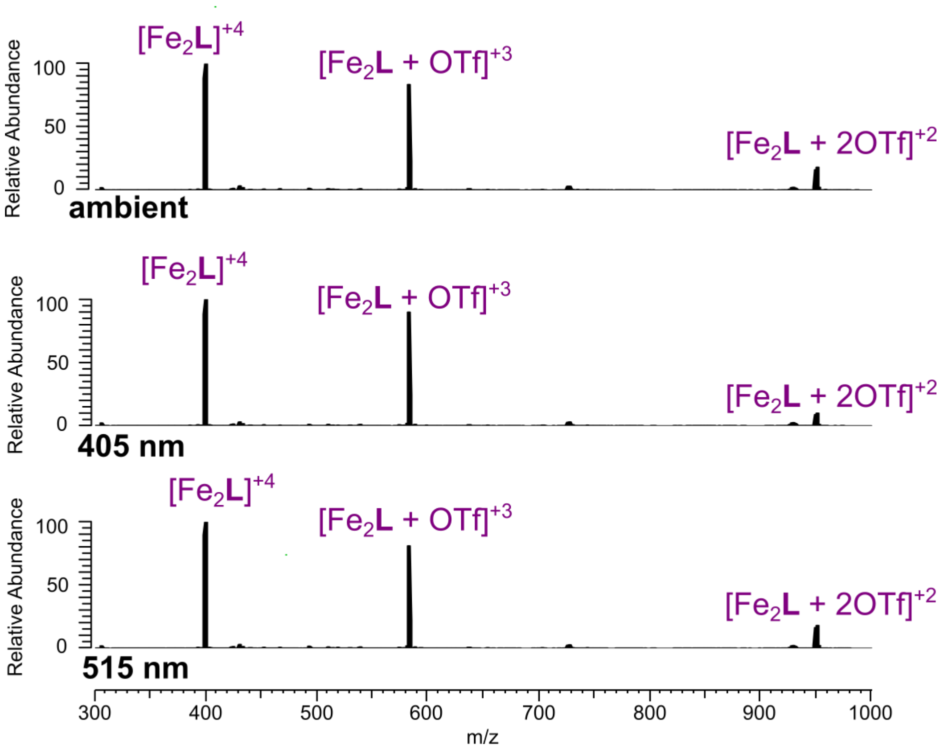

Figure S100. ESI$^+$ mass spectra (CH_3CN, 0.1 mM) of Fe_2**L** under ambient conditions and after irradiation with 405 nm and 515 nm light, respectively (top to bottom). No new peaks are observed after irradiation but the relative intensity of the [Fe_2**L** + 2OTf]$^{2+}$ peak decreases slightly after irradiating with 405 nm and recovers when switching back with 515 nm.

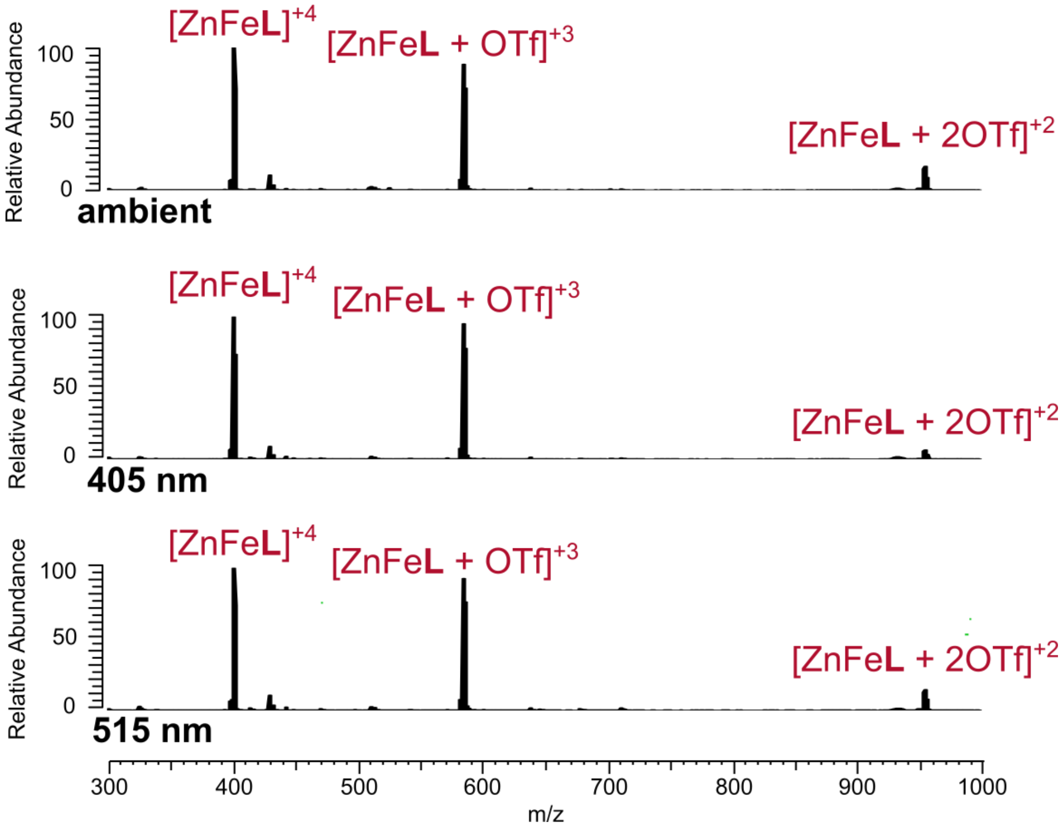

Figure S101. ESI(+) mass spectra (CH_3CN, 0.1 mM) of ZnFe**L** under ambient conditions and after irradiation with 405 nm and 515 nm light, respectively (top to bottom). No new peaks are observed after irradiation but the relative intensity of the [ZnFe**L** + 2OTf]$^{2+}$ peak decreases slightly after irradiating with 405 nm and recovers when switching back with 515 nm.

S7.6 Possible isomerisations observed during photoswitching

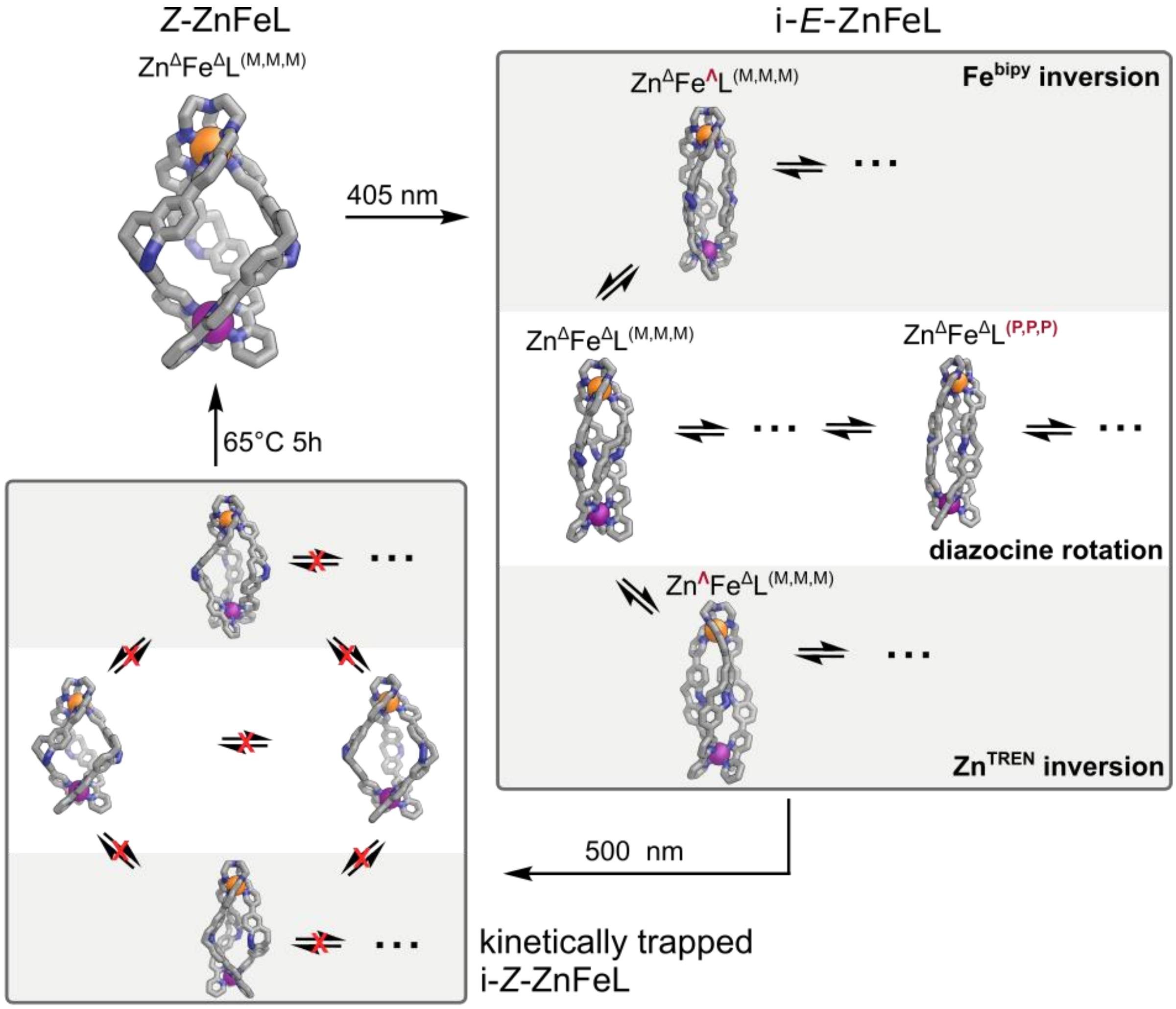

Figure S102. Potential isomerisations of the helicate during photoswitching, using ZnFe**L** as an example. Mass spectrometry (see section S7.5) and DOSY NMR (see section S7.4.2) indicate that both approximate size and molecular formula are retained, but in-situ illuminated NMR spectroscopy (see section S7.4.3) indicates that photoswitching with 405 nm forces an isomerisation reaction, producing multiple products. Back-switching with 500 nm/white light brings the photoswitches back into the *Z* state, and the isomerisation of the helical structure is kinetically trapped. Reforming the initial structure requires heating with the temperature and time of heating strongly depending on the metal in the bipyridine binding site, indicating that breaking of the M−bipy bonds is the rate determining step for the reformation of the initial helicate. Potential isomerisations could occur at the two metal centres that could invert their stereochemistry or by rotation of the diazocines around sigma bonds which should exhibit a much lower barrier in the *E* state than in the *Z* state, where the inversion of the N=N double bond would be required.

S8 Irradiation of ZnFe**L** with full spectrum light for long periods

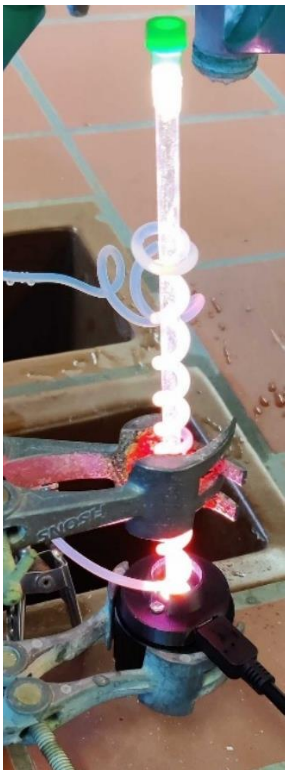

Figure S103. Experimental setup for the long-term irradiation of ZnFe**L** with white light. An NMR sample (1 mM, CD_3CN) was placed on the white LED and flexible tubing was wrapped around the NMR tube. Cold water (approximately 15 °C) was circulated through the tubing to prevent the sample from overheating during the prolonged irradiation time.

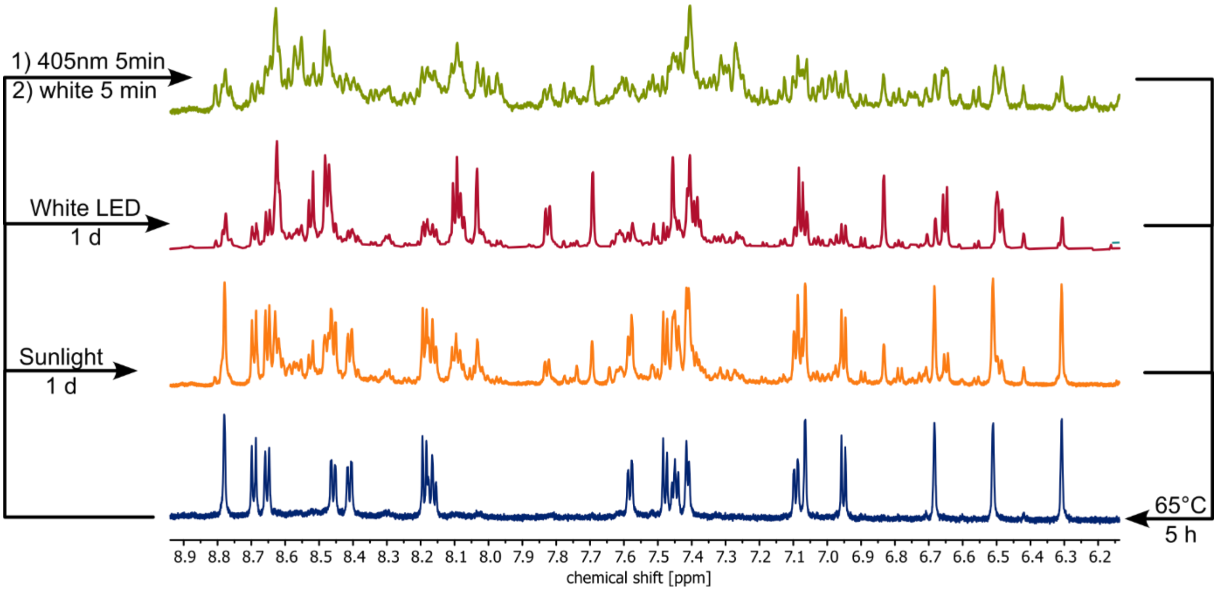

Figure S104. Partial ^{1}H NMR spectra (700 MHz, CD_3CN, 1 mM, 298 K) of ZnFe**L** before and after irradiation with sunlight for one winter day, a white LED for 24 hours, and consecutive irradiation with 1) 405 nm for 5 minutes and 2) white light for 5 minutes. Despite visible light being unable to accumulate meta-stable *E*-diazocines, a drastic photoinduced change is observed.

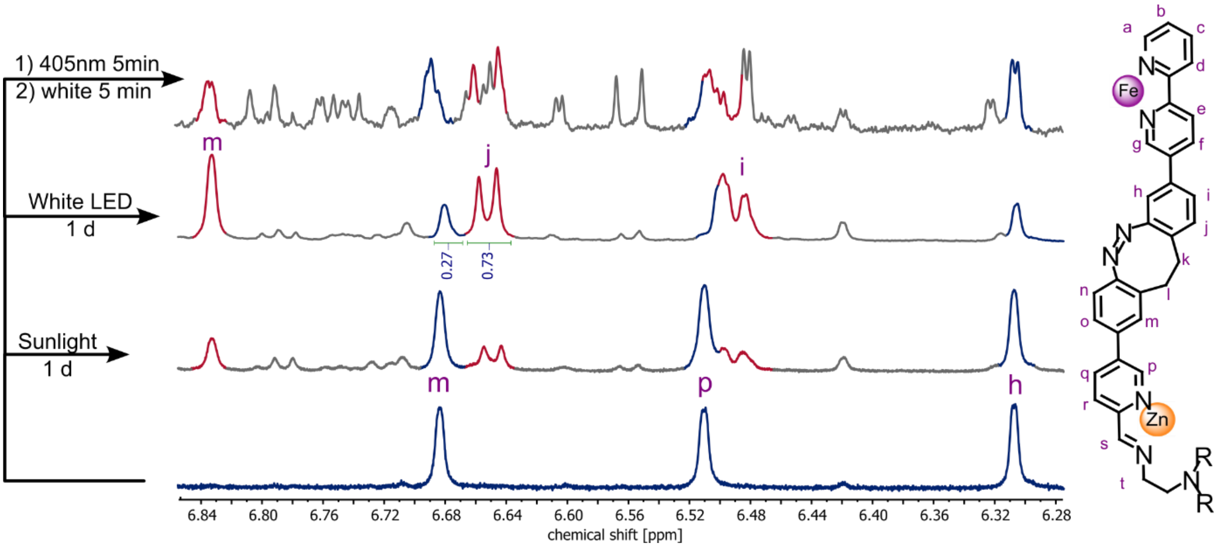

Figure S105. Partial ^{1}H NMR spectra (700 MHz, CD_3CN, 1 mM, 298 K) of ZnFe**L** before and after irradiation with sunlight for one winter day, a white LED for 24 hours, and consecutive irradiation with 1) 405 nm for 5 minutes and 2) white light for 5 minutes. The ground state structure and the new structure are highlighted in blue and red, respectively.

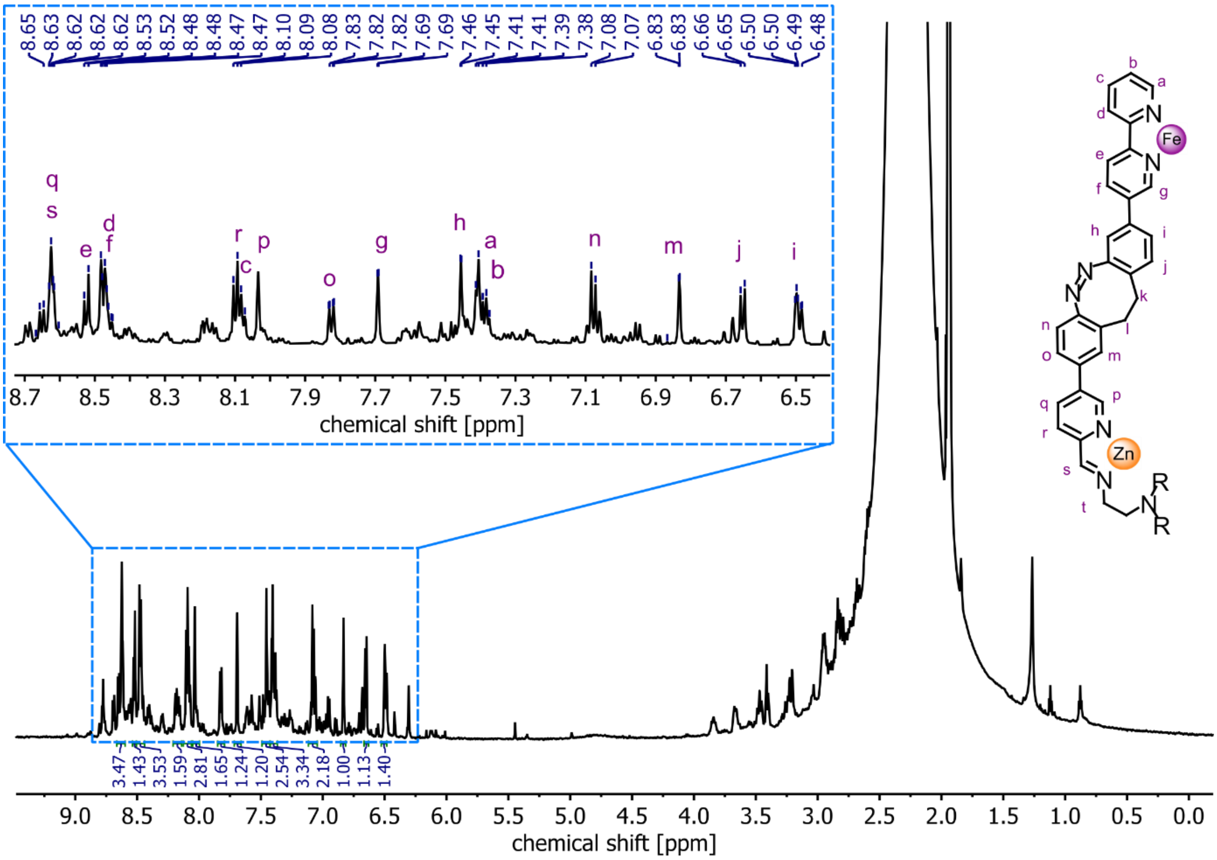

Figure S106. ^{1}H NMR spectrum (700 MHz, CD_3CN, 298 K) of Fe_2**L** after 24 hours white light irradiation.

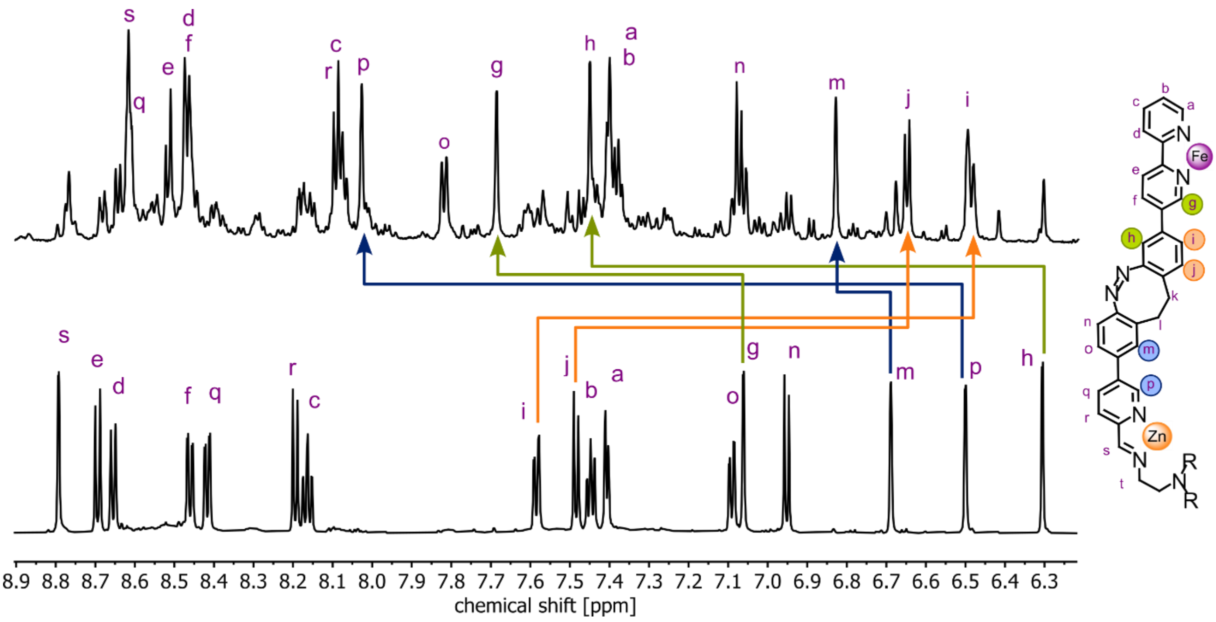

Figure S107. Partial ^{1}H NMR spectra (700 MHz, CD_3CN, 1 mM, 298 K) of ZnFe**L** before (bottom) and after white light irradiation for 24 hours (top) with arrows indicating the changes for highlighted proton signals.

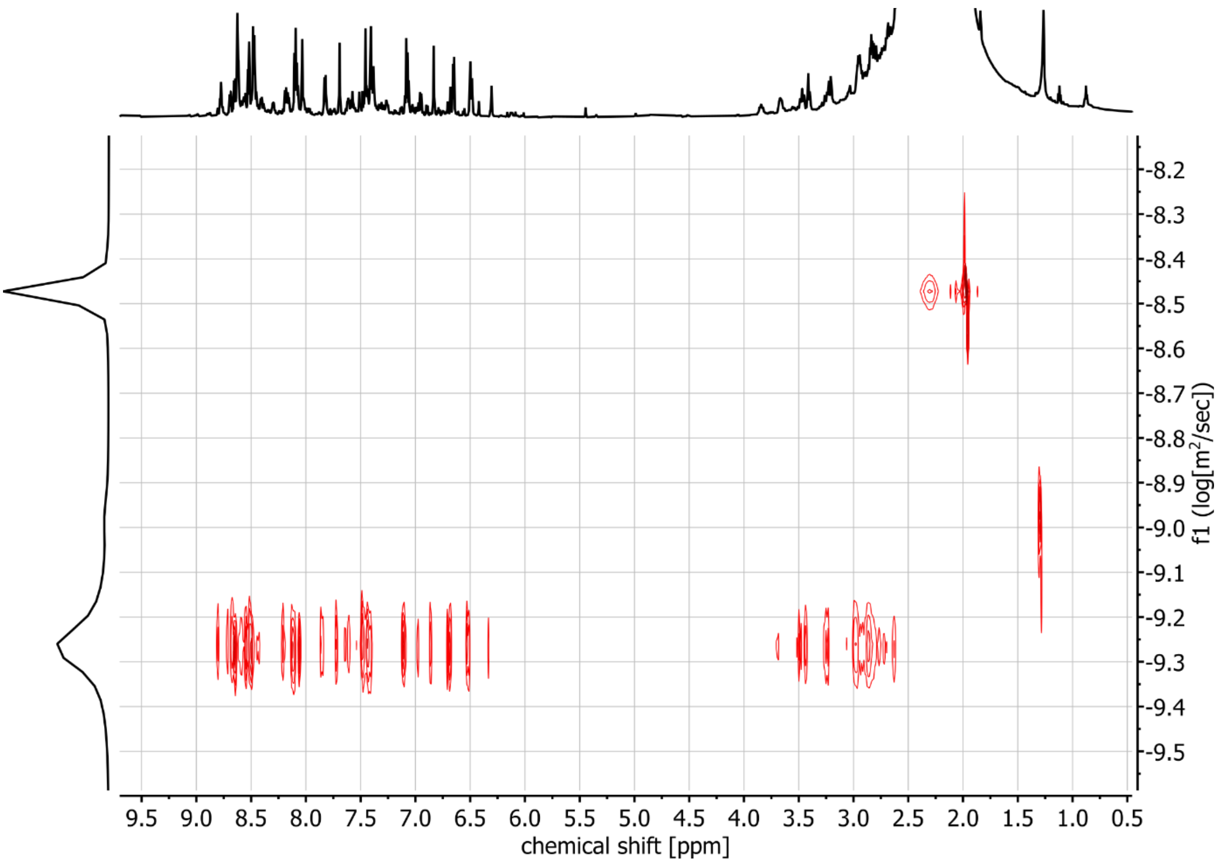

Figure S108. ^{1}H DOSY spectrum (700 MHz, CD_3CN, 1 mM, 298 K, D_{20} = 50 ms) of ZnFe**L** after 24 hours white light irradiation.

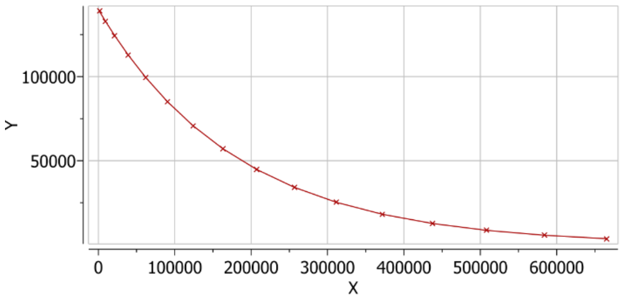

D = 5.500e^{-10} m^{2}s^{-1}

2r = 20.3 Å

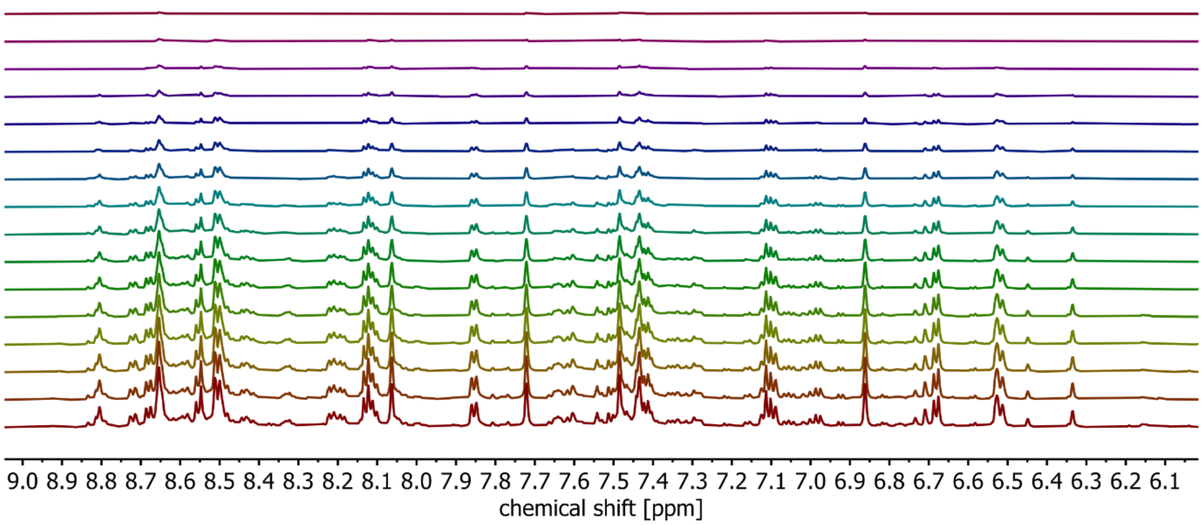

Figure S109. ^{1}H DOSY spectrum (700 MHz, CD_3CN, 1 mM, 298 K, D_{20} = 50 ms) of ZnFe**L** after 24 hours white light irradiation.

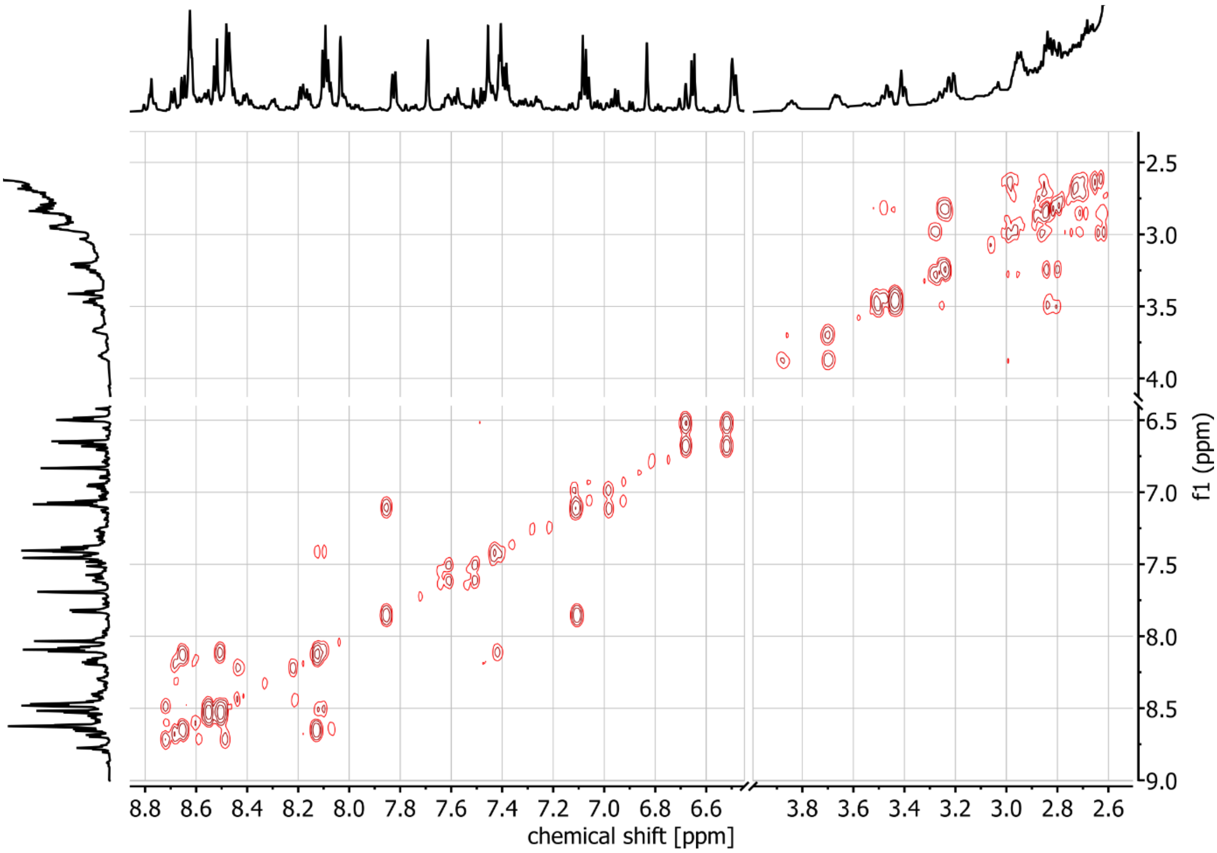

Figure S110. $^{1}H,^{1}H$ COSY spectrum (700 MHz, CD_3CN, 298 K) of ZnFe**L** after 24 hours white light irradiation.

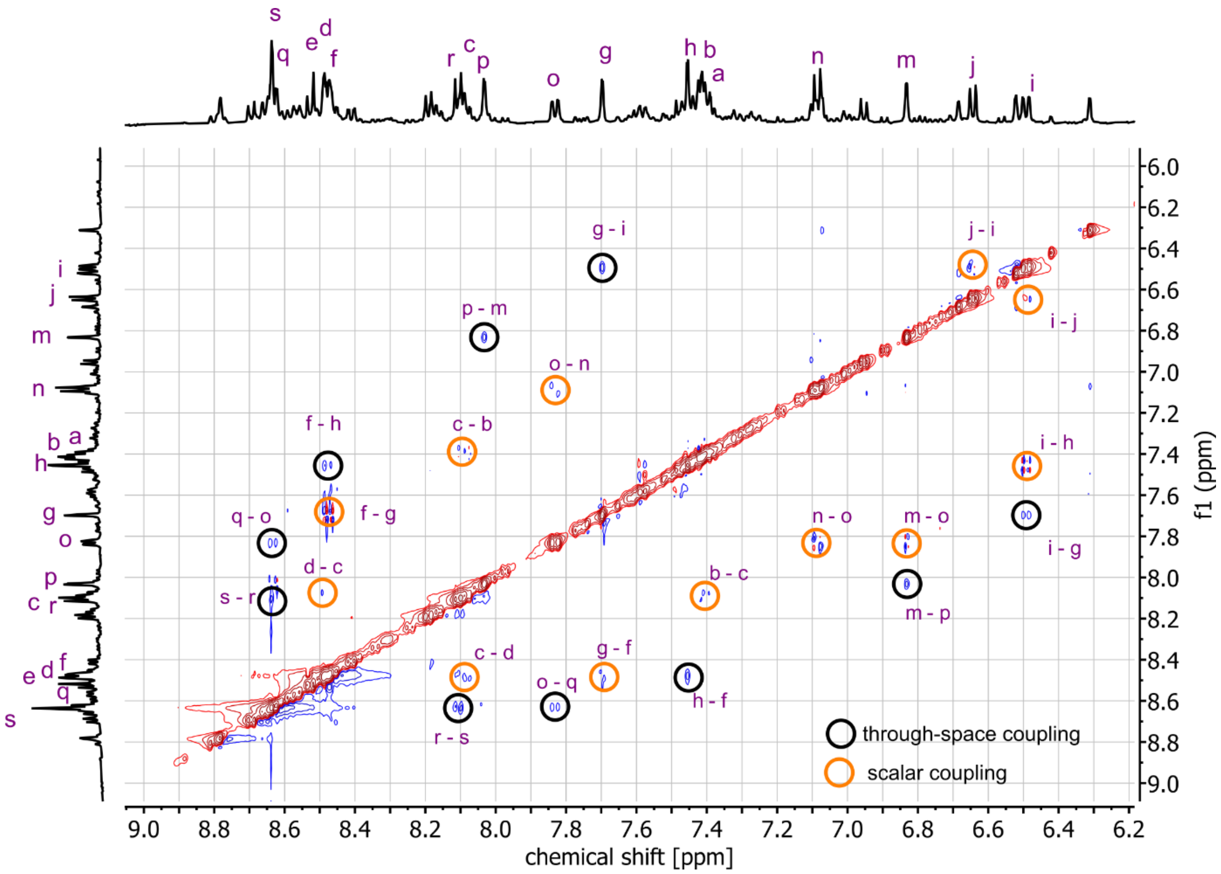

Figure S111. $^{1}H,^{1}H$ ROESY spectrum (500 MHz, CD_3CN, 298 K) of ZnFe**L** after 24 hours white light irradiation.

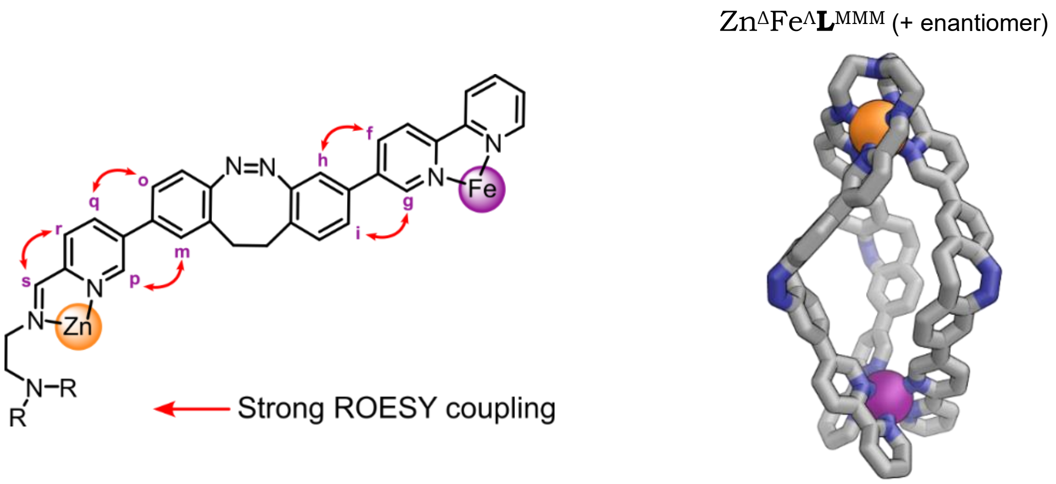

Figure S112. Through space interactions observed by ROESY NMR (Figure S111) show that white light irradiation results in the Zn$^{\Delta}$Fe$^{\Lambda}$**L**^{MMM} diastereomer (and its enantiomer), based on neighbouring hydrogen atoms in the computational structures (Table S15).

Table S15. Computational structures for the four remaining diastereomers of ZnFe**L** after ruling out mixed chirality ligands (Table S10) and ChemDraw representations of the ligand folding (for computational details see Section S6.3). Only Zn$^{\Delta}$Fe$^{\Lambda}$**L**^{MMM} (and its enantiomer) show the correct folding that could give rise to the ^{1}H ROESY cross-peaks observed in Figure S111.

Zn$^{\Delta}$Fe$^{\Delta}$**L**^{MMM}	Zn$^{\Delta}$Fe$^{\Delta}$**L**^{PPP}	Zn$^{\Delta}$Fe$^{\Lambda}$**L**^{MMM}	Zn$^{\Delta}$Fe$^{\Lambda}$**L**^{PPP}
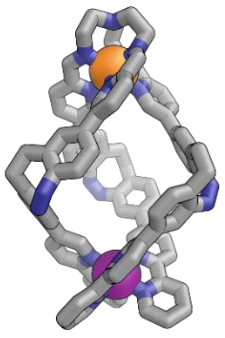<br>(+ enantiomer)	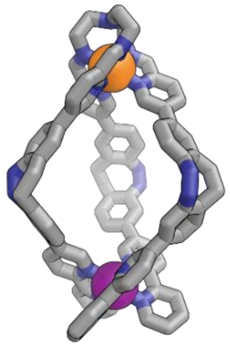<br>(+ enantiomer)	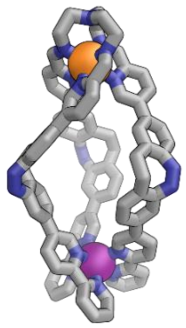<br>(+ enantiomer)	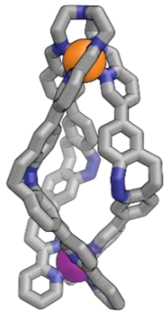<br>(+ enantiomer)
Ligand conformation in computational structure:			
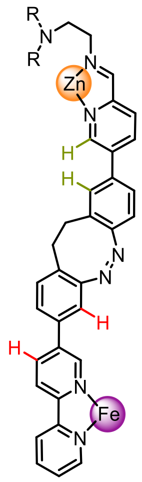	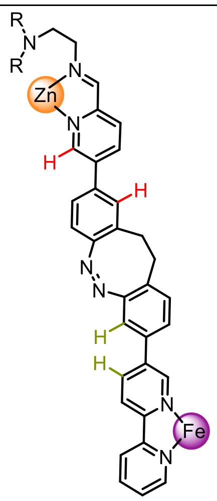	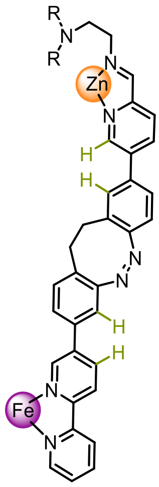	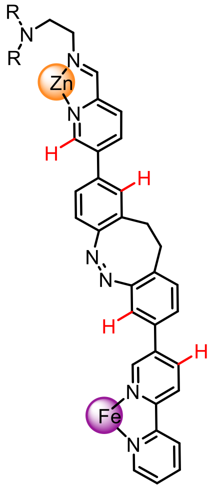
H-p and H-m are adjacent<br>H-g and H-i are not adjacent	H-p and H-m not adjacent<br>H-g and H-i are not adjacent	H-p and H-m are adjacent<br>H-g and H-i are adjacent	H-p and H-m not adjacent<br>H-g and H-i are not adjacent

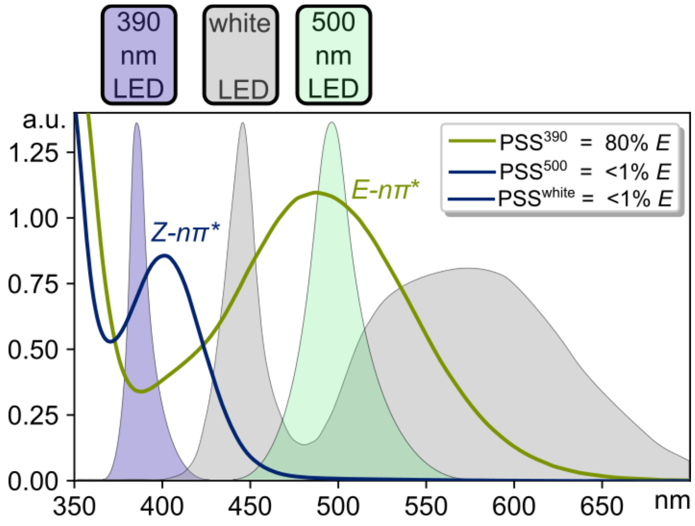

Figure S113. UV-vis spectrum of the diazocine chromophore (0.1 mM, CH_3CN) at the photostationary states after irradiation with 390 nm, 500 nm, and white light with an overlay of the normalized emission spectra of the LEDs used for irradiation, showing a small overlap of the white LED's emission spectrum with the *Z*-nπ* band and a large overlap with the *E*-nπ* band. This means that at the photostationary state of the white LED, the diazocine is constantly being switched into the *E*-state, but back switching is much faster due to the better spectral overlap, resulting in no significant accumulation of *E*-diazocine and a photostationary state identical to PSS^{500}.

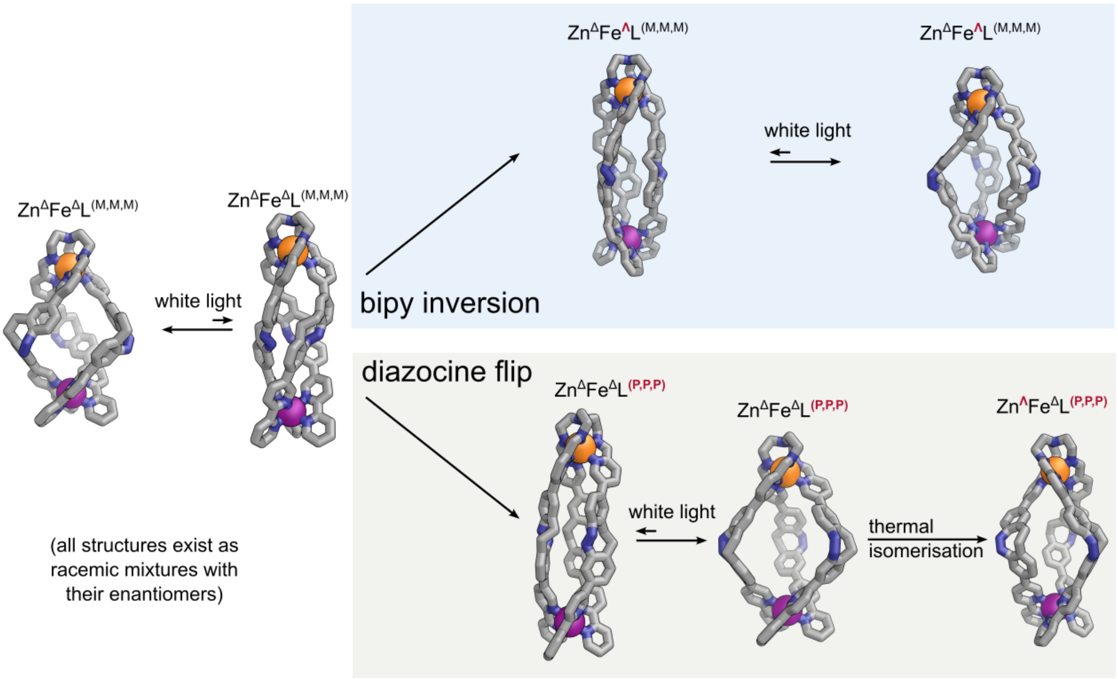

Figure S114. Postulated isomerisation pathways for the white light induced isomerisation of ZnFe**L** occurring at room temperature. Experimental data suggests the conversion of $Zn^{\Delta}Fe^{\Delta}$**L**^{MMM} (and its enantiomer) into $Zn^{\Delta}Fe^{\Lambda}$**L**^{MMM} (and its enantiomer).

$Zn^{\Delta}Fe^{\Delta}L^{MMM}$ to $Zn^{\Delta}Fe^{\Lambda}L^{MMM}$ isomerization may occur either by the inversion of the stereochemistry at the Fe^{bipy} centre in the switched *E*-helicate or by the rotation of the diazocine moieties in the *E*-helicate, followed by a stereoinversion at the Zn^{TREN} centre. The bipy-inversion path is supported by the observation that this binding site is destabilised in the *E*-state (Section S9), which would also accelerate Fe^{bipy} stereo isomerisation. However, the ligand dissociation/association potentially involved in the inversion of the Fe^{bipy} moiety seems to occur on a slower timescale spanning multiple hours at elevated temperatures (Section S9). The diazocine-flip Zn^{TREN}-inversion pathway is supported by the observation that isomerisation occurs within minutes, even at ambient temperature (Figure S85 and Figure S98), indicating that only low-energy barriers must be overcome in the process. Rotation around two single bonds, necessary for flipping the *E*-diazocine moieties, does not present a high energetic barrier, and the relatively labile coordinative bonds of Zn(II) do not create a significant barrier for Zn^{TREN} stereoisomerization. Additionally, the diazocine-flip mechanism may generate mixed chirality structures, potentially accounting for the complex spectra observed following the 405nm/white light switching. ^{1}H NMR data of Fe_2**L** (Figure S19) show strongly broadened signals for hydrogen atoms near the Fe^{TREN} centre, suggesting that dynamic processes may be occurring at room

temperature and that the M^{TREN} moiety might be relatively flexible, allowing for possible chiral inversion at room temperature. Comparing the two potentially isomerised metal centres, Zn^{TREN} presents a stronger coordination pocket but is a much more labile metal compared to Fe^{bipy}. The strongly distorted coordination sphere surrounding Zn^{TREN}, with a seventh nitrogen in close proximity to the Zn(II) cation in the crystal structure (Section S6.2), might further suggest the possibility of chirality inversion at room temperature. While these considerations may hint at the diazocine-flip Zn^{TREN}-inversion mechanism, there is no definitive experimental evidence for either reaction pathway, and the precise nature of the isomer conversion mechanism remains unclear.

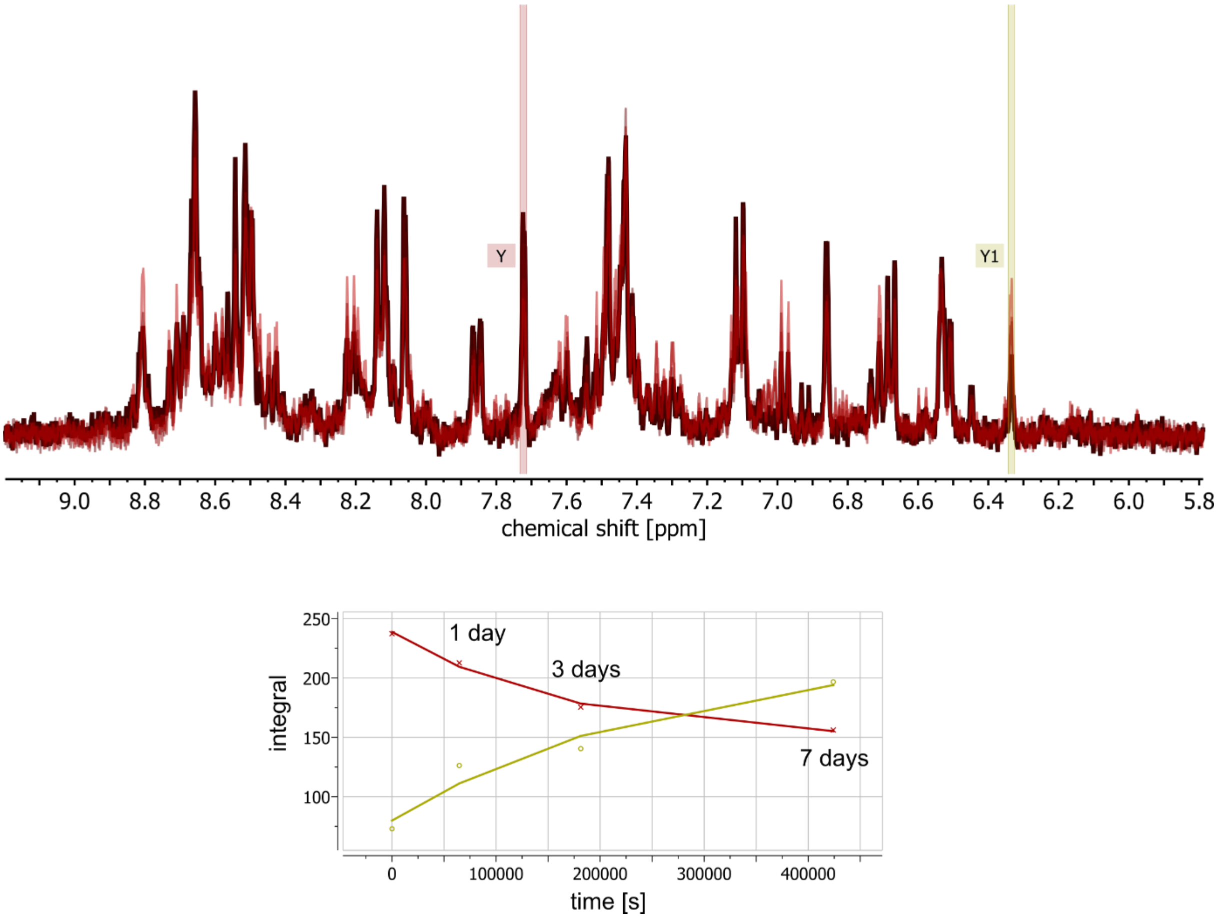

Figure S115. Thermal back-isomerisation of ZnFe**L** after 24 hours of white light irradiation as followed by ^{1}H NMR spectrometry (400 MHz, CD_3CN, 1 mM, 298 K). Bottom: Plotted integrals of meta-stable diastereomer of ZnFe**L** (red, Y) and stable isomer ZnFe**L** (yellow, Y1) proton signals over time with data points fitted to first-order kinetics. Reforming of lowest energy isomer ZnFe**L** helicate follows apparent first-order kinetics with no intermediates being observed.

S9 Metal Exchange

S9.1 Thermal relaxation of ZnFe**L** at 65 °C for reference

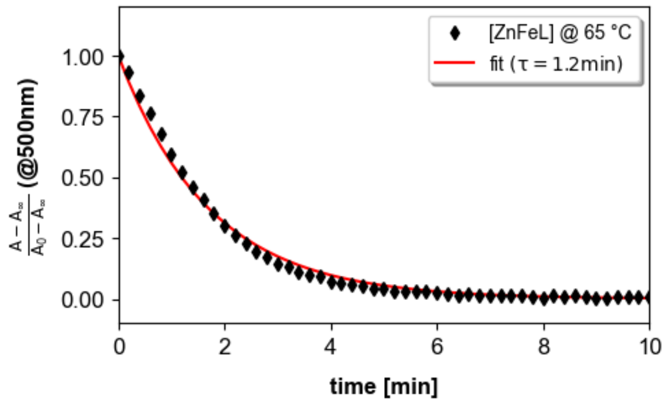

Figure S116. UV-vis kinetics and fit of the thermal relaxation of ZnFe**L** (CH_3CN, 0.06 mM, 65 °C) after irradiation at 405 nm for 1 minute. The thermal half-life of ZnFe**L** was determined to be $\tau_{½}$ = 1.2 min at 65 °C (compare Figure S117).

S9.2 Metal-exchange kinetics by UV-Vis

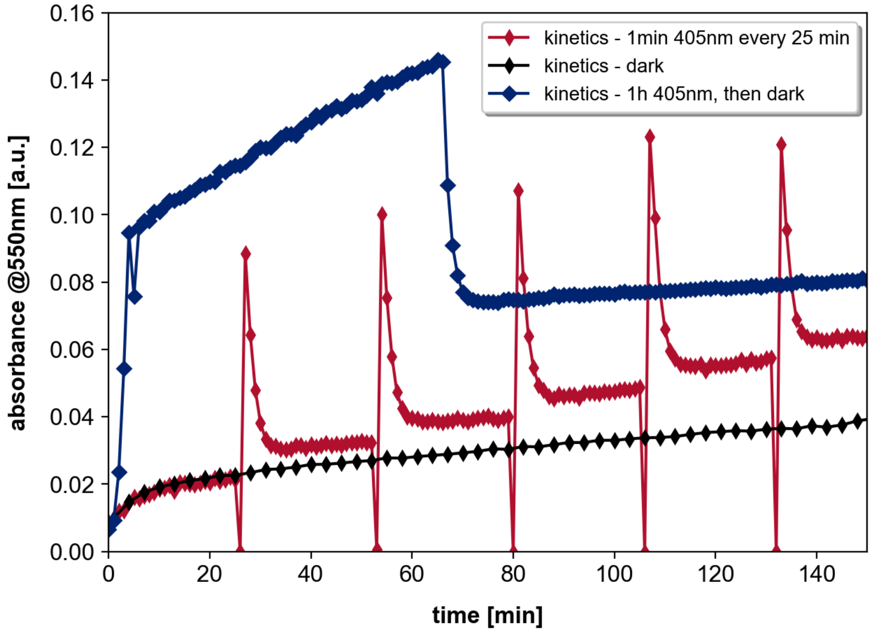

Figure S117. UV-vis kinetics of the metal exchange of Zn_2**L** to ZnFe**L**, following the formation of Fe^{bipy} by the appearance of its MLCT band at 550 nm. A stock solution of Zn_2**L** (CH_3CN, 0.06 mM) was cooled to 6°C and 1.5 equiv. of $Fe(OTf)_2$ were added. Three aliquots were consecutively taken from this mixture and transferred to a UV-vis cuvette, which was then immediately placed into the UV-vis spectrometer that had been preheated to 65°C. The first sample was measured in the dark (black), the second sample was irradiated at 405 nm for 1 minute every 25 minutes (red), and the last sample was continuously irradiated for 1 hour (blue).

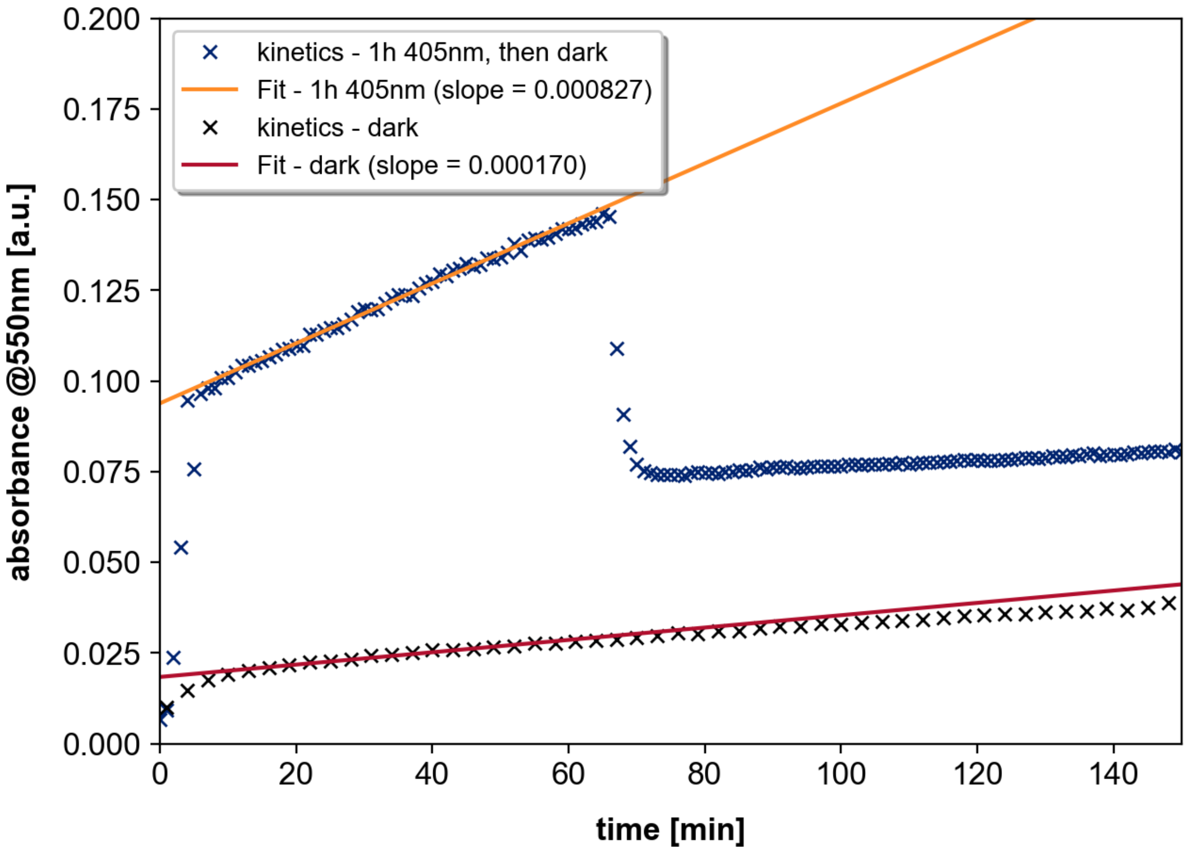

Figure S118. Fitted linear regressions to the experimental UV-vis data Zn_2**L** to ZnFe**L** metal exchange experiment (Figure S117, 1 h 405 nm irradiation, then 1h dark). The linear regression was performed using the data from t = 10 min to t = 60 min. The fit for the irradiated rate yielded a slope of 0.000827. In contrast, the reaction without irradiation had a slope of 0.000170, indicating that irradiation accelerates the metal exchange reaction by a factor of approximately 5.

S9.3 Metal-exchange kinetics by NMR

The kinetics of the Zn_2**L** to ZnFe**L** metal exchange reaction were investigated using high-temperature ^{1}H NMR spectroscopy (Figure S119, 700 MHz, CD3CN, 1 mM, 338 K). Besides the signals belonging to the starting material and the product, a third group of signals appeared and disappeared again (Figure S120). The experimental data suggests that the reaction kinetics did not follow first-order rate laws and that a transient intermediate or side product, tentatively assigned to a "Zn_2Fe**L**" state (Figure S122), was formed. This state seems unable to directly form the ZnFe**L** product and may be considered an unproductive resting state. The kinetics modeling program COPASI (version 4.44)[24] was used to fit this two-equilibria, three-state model (Figure S121) to the data obtained from the NMR experiments (Figure S122 and Table S16). The results of this fit seem reasonable and can explain the rapid disappearance of the starting material, as both the product and resting state are formed through equilibrium reactions. The overall equilibrium lays on the side of the desired ZnFe**L**, which appears to be the thermodynamically most stable species. Over time, the slow backreaction of "Zn_2Fe**L**" to Zn_2**L** pulls the mixture into the thermodynamic minimum, resulting in the predominant formation ZnFe**L**. The exact structure or nature of the "Zn_2Fe**L**" species could not be ascertained from the available data.

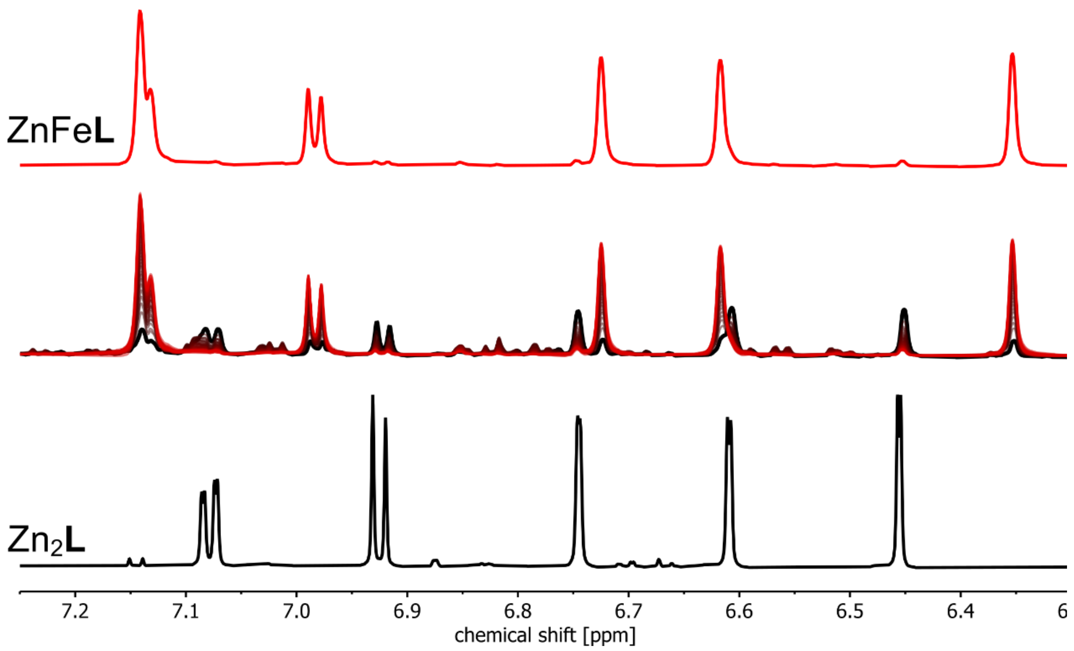

Figure S119. High-temperature ^{1}H NMR spectra (700 MHz, CD_3CN, 1 mM, 338 K) of ZnFe**L**, Zn_2**L** after the addition of 1.5 equiv. $Fe(OTf)_2$ with spectra being recorded every 5 minutes for 5.5 hours (maroon to red), and Zn_2**L** (top to bottom).

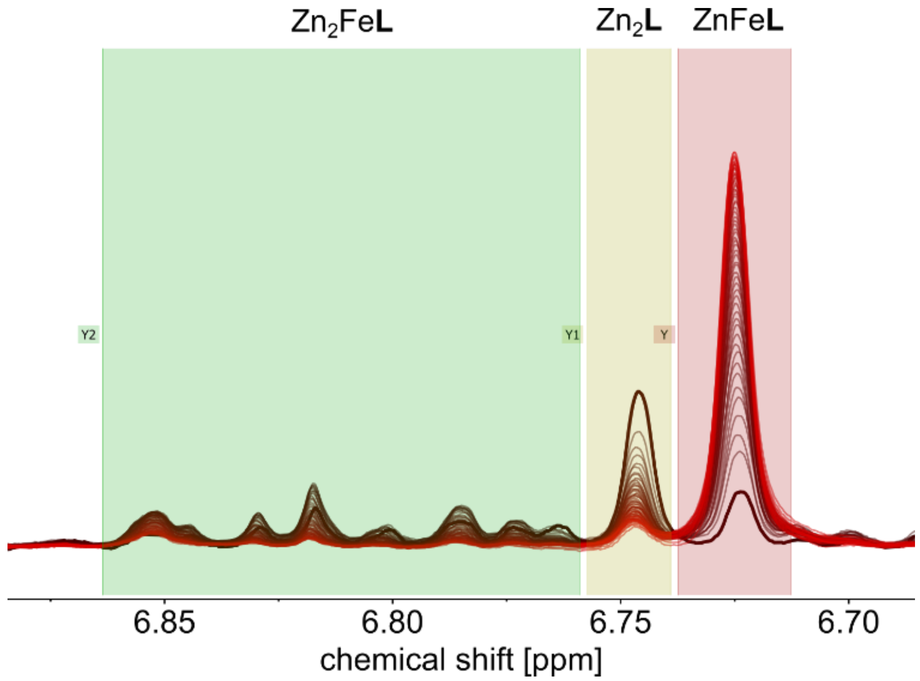

Figure S120. Partial high-temperature ^{1}H NMR spectra (700 MHz, CD_3CN, 1 mM, 338 K) of Zn_2**L** after the addition of 1.5 equiv. $Fe(OTf)_2$. Coloured panels show the signals assigned to different species for the evaluation of the kinetics of the metal exchange process.

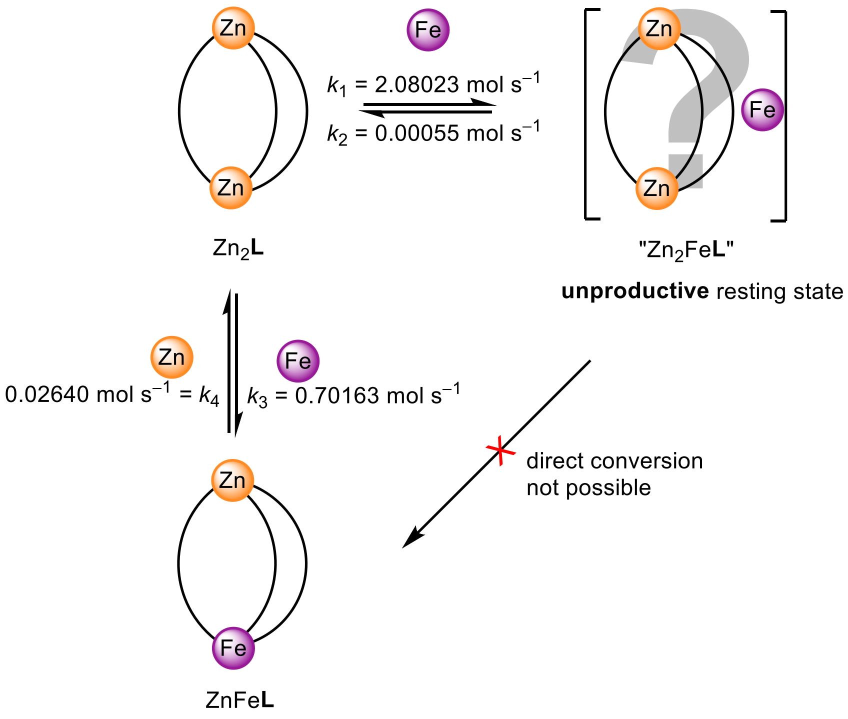

Figure S121. Kinetics model used for fitting of the NMR data (Figure S120). At first glance, the experimental data indicated that the reaction kinetics did not follow first-order rate laws and that a transient intermediate or side product was formed. Since this only forms after the addition of Fe(II) ions, the additional NMR signals were tentatively assigned to a "Zn_2Fe**L**" state. Kinetic modelling indicated that this state was unable to directly form the ZnFe**L** product and can be regarded as an unproductive resting state.

Table S16. Parameter estimation results for the kinetic model (Figure S121) using the genetic algorithm built into the kinetics modelling software COPASI.[24]

Parameter	Lower Bound	Upper Bound	Value [mol s^{-1}]	Std. Deviation
k_1	1.00E-06	10000	2.08023	0.050440
k_2	1.00E-06	10000	0.00055	0.000013
k_3	1.00E-06	10000	0.70163	0.008606
k_4	1.00E-06	10000	0.02640	0.001452

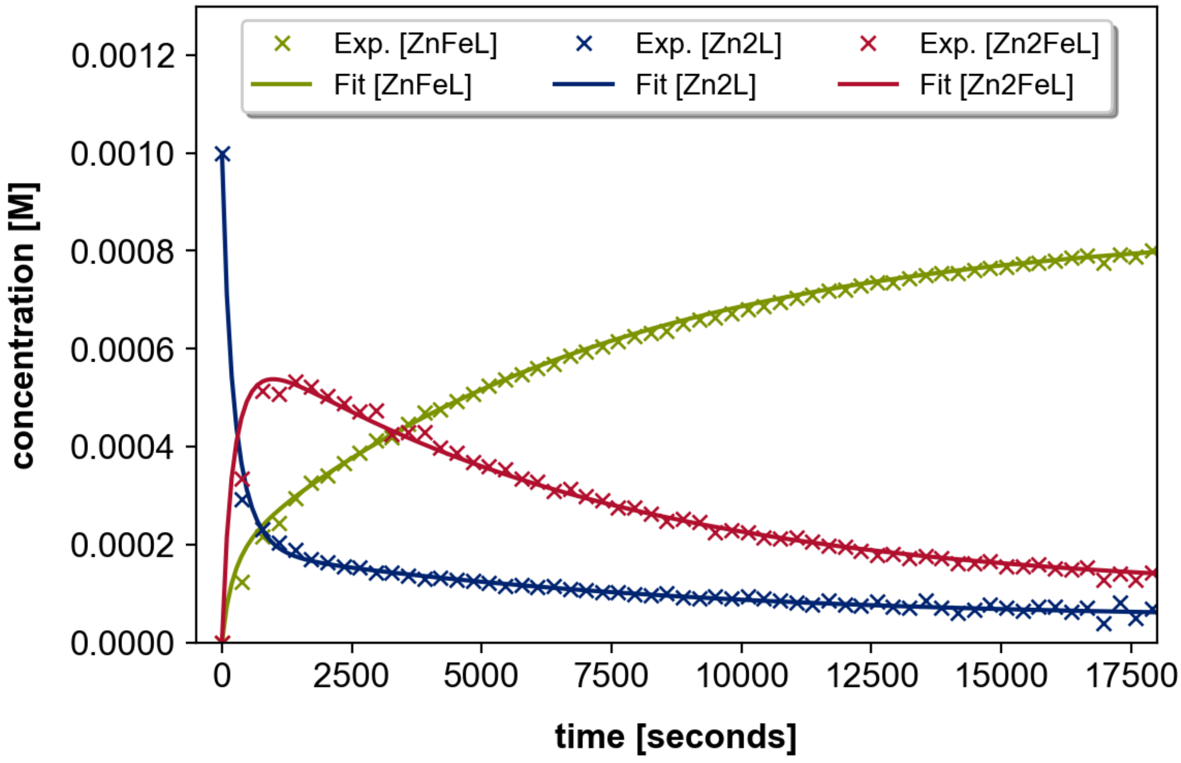

Figure S122. Kinetic model (Figure S121) fitted to experimental data (Figure S120) using the COPASI program.[24]

S9.4 Stability of ZnFeL and metal scrambling

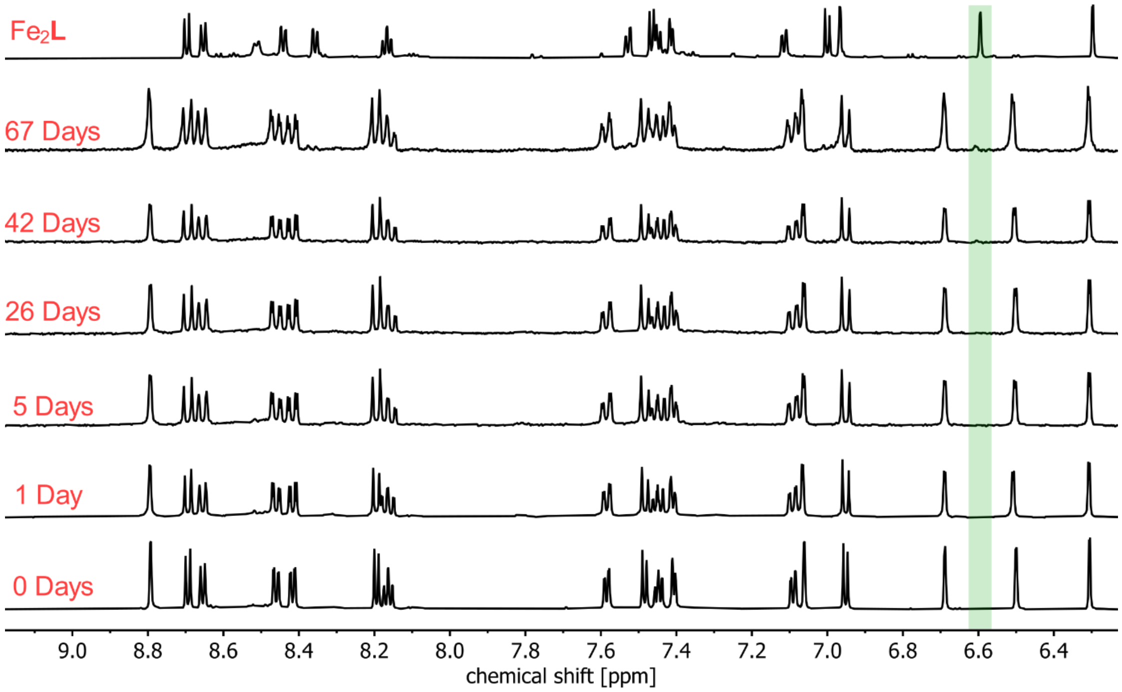

Figure S123. Partial ^{1}H NMR spectra (400 MHz, CD_3CN, 1 mM, 298 K) of ZnFe**L** during stability tests. The sample was synthesised using the metal exchange reaction of Zn_2**L** with 1.5 equiv. $Fe(OTf)_2$. The sample was heated to 65 °C, with measurements taken periodically after 0, 1, 5, 26, 42, and 62 days (bottom to top). The very slow emergence of signals belonging to Fe_2**L** (green box) due to metal exchange at the Zn^{TREN} site was observed. No signals corresponding to Zn_2**L** are observed, indicating that traces of free Fe(II) ions remaining from the synthesis were involved in the exchange, and the Zn(II) ions ended up as solvated ions in solution rather than in any complex.

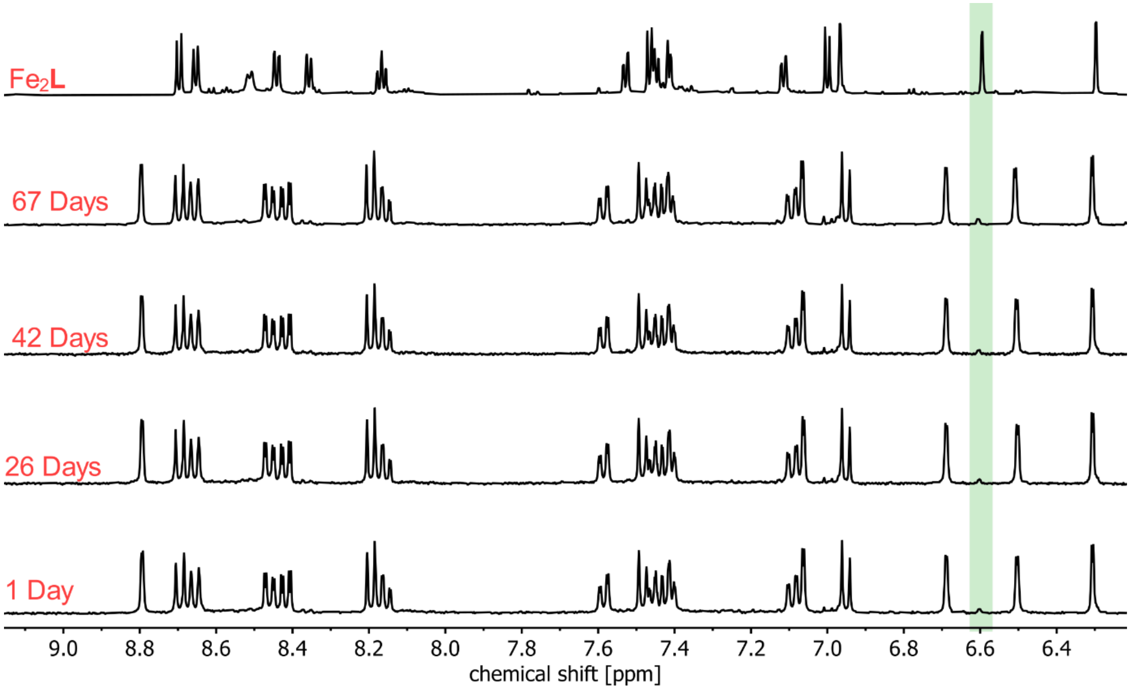

Figure S124. Partial ^{1}H NMR spectra (400 MHz, CD_3CN, 1 mM, 298 K) of ZnFe**L** (derived from the one-pot reaction, therefore containing 6% Fe_2**L**) during stability tests. 3.0 equiv. of $Fe(OTf)_2$ were added to the sample and it was heated to 65 °C, with measurements taken periodically after 1, 26, 42, and 62 days (bottom to top). The very slow increase of signals belonging to Fe_2**L** (green box) due to metal exchange was observed. No signals corresponding to Zn_2**L** are observed, indicating that excess Fe(II) ions were involved in the exchange, and the Zn(II) ions ended up as solvated ions in solution rather than in any complex.

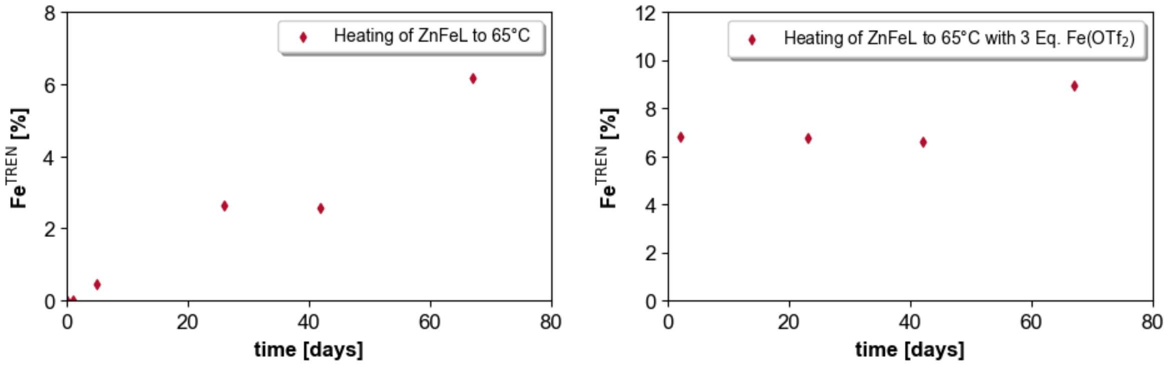

Figure S125. Plot of the percentage of TREN coordination sites occupied by Fe(II) (Fe^{TREN}) in % over time, derived from the signals in the green boxes from Figure S123 (left) and Figure S124 (right).

S10 Dilution experiment for Zn_2L

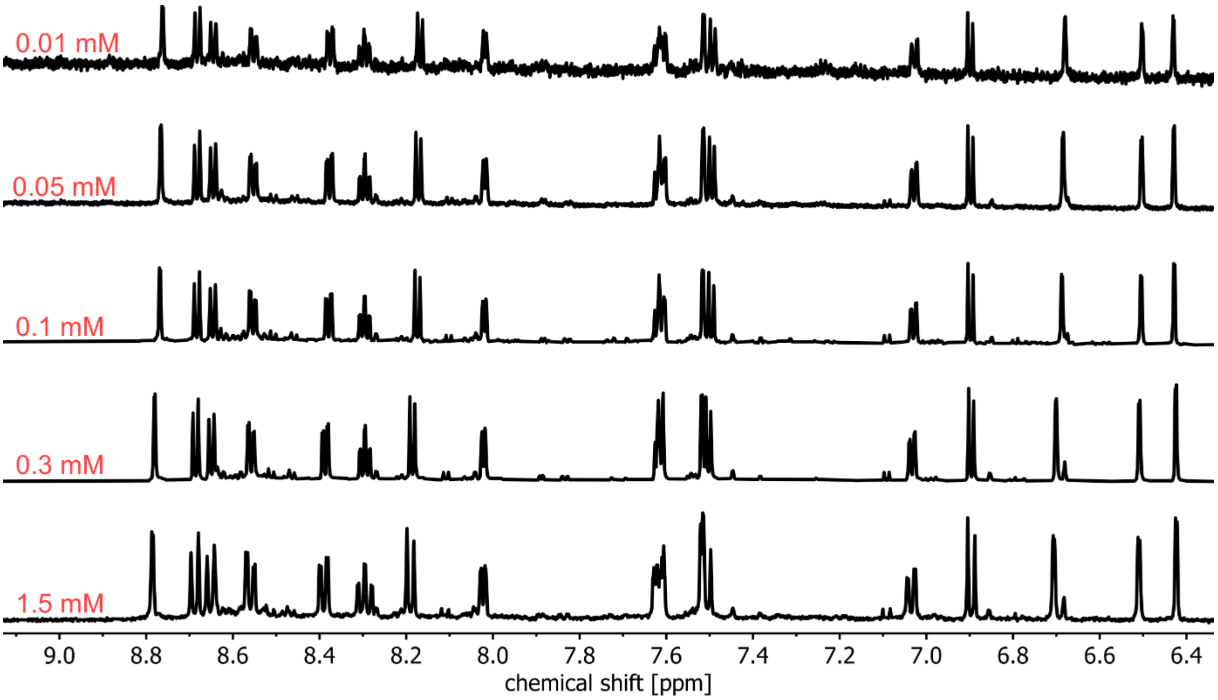

Figure S126. ^{1}H NMR spectra (700 MHz, CD_3CN, 298 K, up to 128 scans) of Zn_2**L** at concentrations from 0.01 mM to 1.5 mM (top to bottom) showing no observable disassembly upon dilution of the samples, because no signals corresponding to the free ligand or any other species could be observed.

S11 Literature

[1] M. J. Notheis, V. Sahiti, V. Prangenberg, J. S. Kruse, L. von Krbek, *Synlett* **2025**, DOI 10.1055/a-2567-1399.

[2] M. Kieffer, B. S. Pilgrim, T. K. Ronson, D. A. Roberts, M. Aleksanyan, J. R. Nitschke, *J. Am. Chem. Soc.* **2016**, *138*, 6813–6821.

[3] D. A. Roberts, B. S. Pilgrim, J. D. Cooper, T. K. Ronson, S. Zarra, J. R. Nitschke, *J. Am. Chem. Soc.* **2015**, *137*, 10068–10071.

[4] N. Oka, T. Yamada, H. Sajiki, S. Akai, T. Ikawa, *Org. Lett.* **2022**, *24*, 3510–3514.

[5] X-Area LANA 2.7.9.0 (STOE&Cie, **2022**.)

[6] G. M. Sheldrick, *Acta Crystallogr. Sect. C: Struct. Chem.* **2015**, *71*, 3–8.

[7] G. M. Sheldrick, *Acta Crystallogr. Sect. A* **2015**, *71*, 3–8.

[8] A. L. Spek, *Acta Crystallogr. Sect. C* **2015**, *71*, 9–18.

[9] L. Turcani, A. Tarzia, F. T. Szczypiński, K. E. Jelfs, *J. Chem. Phys.* **2021**, *154*, 214102.

[10] L. Turcani, A. Tarzia, *Stk*, https://github.com/lukasturcani/stk.

[11] A. Tarzia, K. E. Jelfs, *Chem. Commun.* **2022**, *58*, 3717–3730.

[12] M. D. Hanwell, D. E. Curtis, D. C. Lonie, T. Vandermeersch, E. Zurek, G. R. Hutchison, *J. Cheminformatics* **2012**, *4*, 17.

[13] A. K. Rappe, C. J. Casewit, K. S. Colwell, W. A. Goddard, W. M. Skiff, *J. Am. Chem. Soc.* **1992**, *114*, 10024–10035.

[14] P. Pracht, S. Grimme, C. Bannwarth, F. Bohle, S. Ehlert, G. Feldmann, J. Gorges, M. Müller, T. Neudecker, C. Plett, S. Spicher, P. Steinbach, P. A. Wesołowski, F. Zeller, *J. Chem. Phys.* **2024**, *160*, 114110.

[15] S. Spicher, S. Grimme, *Angew. Chem. Int. Ed.* **2020**, *59*, 15665–15673.

[16] C. Bannwarth, S. Ehlert, S. Grimme, *J. Chem. Theory Comput.* **2019**, *15*, 1652–1671.

[17] S. Ehlert, M. Stahn, S. Spicher, S. Grimme, *J. Chem. Theory Comput.* **2021**, *17*, 4250–4261.

[18] L. Schrödinger, *The PyMOL Molecular Graphics System, Version~1.8*, **n.d.**

[19] A. Tarzia, W. Shan, V. Posligua, C. J. T. Cox, L. Male, B. D. Egleston, R. L. Greenaway, K. E. Jelfs, J. E. M. Lewis, *Chem. A Eur. J.* **2025**, *31*, e202403336.

[20] C. Feldmeier, H. Bartling, E. Riedle, R. M. Gschwind, *J. Magn. Reson.* **2013**, *232*, 39–44.

[21] Y. Ji, D. A. DiRocco, J. Kind, C. M. Thiele, R. M. Gschwind, M. Reibarkh, *ChemPhotoChem* **2019**, *3*, 984–992.

[22] S. Ghosh, C. Eschen, N. Eleya, A. Staubitz, *J. Org. Chem.* **2022**, *88*, 3372–3377.

[23] M. S. Maier, K. Hüll, M. Reynders, B. S. Matsuura, P. Leippe, T. Ko, L. Schäffer, D. Trauner, *J. Am. Chem. Soc.* **2019**, *141*, 17295–17304.

[24] S. Hoops, S. Sahle, R. Gauges, C. Lee, J. Pahle, N. Simus, M. Singhal, L. Xu, P. Mendes, U. Kummer, *Bioinformatics* **2006**, *22*, 3067–3074.
